# Supplementary material for: Interpreting the pervasive observation of U-shaped Site Frequency Spectra
Source: PLoS Genet. 2023 Mar 23;19(3):e1010677. doi: 10.1371/journal.pgen.1010677 (PMC10072462; doi:10.1371/journal.pgen.1010677)
Supplement: S1 Appendix — (PDF) [file pgen.1010677.s001.pdf]

Appendix A Appendix

A.1 A short primer on Kingman and multiple-merger coalescent models

The genealogy of a sample in many generation-by-generation reproduction models, e.g. the Wright-Fisher or Moran Model, can be approximated by a random tree with random, real-valued branch length. For this primer, we only consider one-locus models and ignore recombination, the general idea stays similar for models featuring recombination, but becomes more challenging mathematically.

Let us consider a reproduction model, for instance a Cannings model [Can74]. A Cannings model assumes that offspring sizes in each generation have the same distribution, but are independent between generation. Within a generation, a (standard) Cannings model assumes that the sum of offspring sizes equals the number of parents, let's say N. This introduces a dependency between offspring sizes of different parents, but they are still have identical distributions by assuming that offspring sizes are exchangeable [HS76]. The discrete genealogy model, a discrete n-coalescent, simply is the (random) tree obtained from tracing back the genealogy of a sample (of size n) taken from some generation. If one now allows bigger and bigger population sizes N, but fixes sample size n, the genealogy trees grow in height (it needs longer to find the Most Recent Common Ancestor, MRCA, that is the root of the tree). However, looking at the right time scale (1 unit of time equalling many generations), the rescaled discrete n-coalescent is very similar to a random tree whose time is measured in continuous time units and not generations. All our coalescent models with fixed population size N are constructed this way.

The coalescent tree we end up with after time rescaling for large population sizes ($N \to \infty$) will often be a Kingman coalescent, if the variance of offspring sizes (and higher moments) stay below some threshold. It is true even for N very large [Kin82]. Note that, for all Canning models, the expected offspring sizes always equal 1, since sizes are identically distributed and sum up to population size N. However, when the variance of offspring is large, multiple merger coalescent (MMC) will appear as the proper coalescent tree model (mathematically, a limit process under mild further assumption [MS01]). Both types of trees (Kingman and MMC trees) are depicted in Figure A.1. Speaking in term of offspring sizes, unbounded variance means that offspring sizes above any threshold must appear with a large enough probability so that the variance is pushed above the threshold. As discussed in the introduction, a variety of biological properties may lead to such (highly) skewed offspring sizes.

Different coalescent models may lead to substantially different patterns of genetic diversity. Generally, genetic diversity results from mutation events occurring on the coalescent tree, which appear randomly with a constant rate across branches (a homogeneous Poisson process). We assume that mutations will all target a different site in the sequence, a model known as the infinite sites model. Thus, each mutation occurring on a branch connecting to a subtree of k leaves is a mutation inherited by k of the n sampled sequences and thus a polymorphic sites of frequency k/n. We are also assuming that the mutation-generating Poisson process is independent from the genealogy-generating coalescent process. This means that, in the models considered here, mutations are assumed to be neutral and thus do not alter the genealogy's shape. However, in the selection-associated MMCs (e.g. the Bolthausen-Sznitman coalescent and its variants [Sch17] and the Durrett-Schweinsberg coalescent [SD05]), selection acting on short enough timescales and on variants that are then fixed are already included in the genealogy model.

The difference in patterns of genetic diversity between different coalescent models are due to two mechanisms: changed branch length distributions and changed distribution of how many lineages merge in a coalescent event. Both interact to produce patterns of genetic diversity and may show complex interactions. Thus, the full information on differences in genetic diversity between coalescent models may only be fully understood by either extensive computer simulation or mathematical analysis. Still, trends can be understood heuristically.

Multiple merger coalescents show U-shaped SFS, that is an excess of low frequency and high frequency variants (which are at the extreme derived and ancestral singletons). We will now provide some heuristics for why the latter two appear (and on which parameter their strength depends) using known results for large n; precise results for expected site frequency spectra for finite n are available e.g. from [SKS16].

Derived singletons (polymorphisms of frequency $1/n$) correspond to mutations that occurred on external branches, connecting a node to a leaf (marked in bold in Figure A.1). The expected relative number of derived singletons is proportional to the ratio of the sum of external branches to the sum of all branches (total tree length). For the Kingman and the Beta trees, many branches coalesce close to the leaves, while this is not true for Psi-coalescents [Pit99] (explaining the excess of derived singletons for Psi-trees). However, external branches for Beta trees are still are considerably relatively longer than for the Kingman trees. They represent on the order of $1/\log(n)$ for Kingman trees, while they are on the order of $n^{2-\alpha}/\log(n)$ for Beta trees (with $1 < \alpha < 2$). Results for different coalescents were obtained by different groups, see [DFSJY13], [GIM14] for a collection of results including references. Thus, we expect a considerably higher fraction of derived singletons among all mutations for MMC trees.

On the other side of the SFS, ancestral singletons (polymorphisms of frequency $(n-1)/n$) correspond to mutations occurring on the branch that connect to the subtree of size $n-1$ (marked in dashed in Figure A.1) [SKS16, Lemma 2]. This tree is highly imbalanced for the MRCA node (having 1 vs $(n-1)$ leaves on both sides). Observing a tree with such a branch is unlikely under the Kingman coalescent, $\mathbb{P} = 2/(n-1)$ but not that uncommon under MMC trees [EF18]. Common unbalanced MMC trees, points towards relatively more ancestral singletons on MMC models.

In addition to the references of the main text, we also point towards [BW21] featuring several recent reviews on aspects of MMC, modelling and inference from a mathematical perspective. For an overview of biological properties leading to MMC genealogies, in addition to our pointers in the introduction, see e.g. [TL14], [JPS$^+$19], [Eld20].

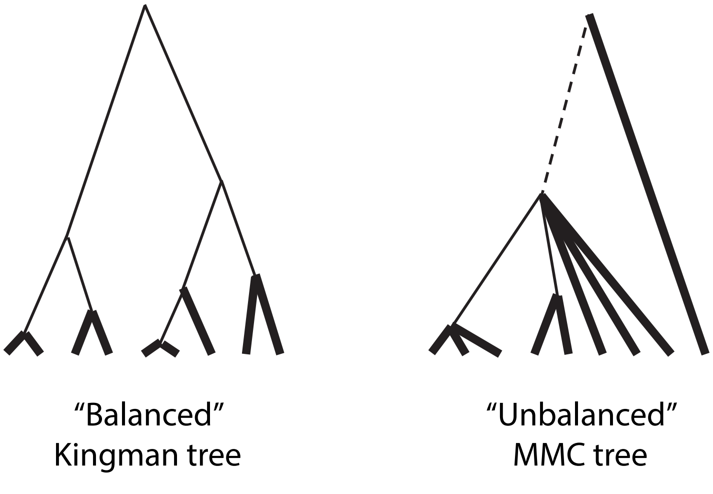

Figure A.1: Illustrative pictures of typical Kingman (left) and MMC (right) coalescent trees. External branches, on which derived singletons can occur, are in bold. In a Kingman tree, they represent a fraction of the order of $1/\log(n)$ of the sum of all branches, whereas they represent a fraction $n^{\alpha-2}/\log(n)$ for a Beta-coalescent. The branch on which ancestral singletons can occur is dashed. This branch is unlikely to exist under the Kingman tree, $\mathbb{P} = 2/(n-1)$, whereas it is more common in MMC trees. The relative inflation of both rare derived and rare ancestral alleles in MMC models creates the U-shape of the SFS.

A.2 Reproduction models linked to MMC and time scalings

The coalescent approximations from the main text are the coalescent limits for population size $N \to \infty$ (with changed time-scale) of genealogical trees given some reproduction model. We focus on Cannings models [Can74] of reproduction, which are discrete-generation models, usually with fixed population size, and exchangeable offspring numbers between individuals. This is a standard model choice, see e.g. [Sag99], and the modified Moran models present in the Methods are Cannings models. Different reproduction models can lead to the same coalescent limit, e.g. the Wright-Fisher and Moran model both lead to Kingman's coalescent. If the coalescent limit is identical for two constant population size reproduction models (and the number of generations to form one coalescent time unit is of order N^{η}), we can describe the limit as in Eq. (1) for both models. Thus, adding population size changes can still lead to a difference in coalescent limit via changing the power η of the population size ratio ν. For instance, $\eta = 2$ for the standard Moran model but $\eta = 1$ for the Wright-Fisher model (Λ the point mass in 0 in both cases). In the case of exponential growth (on the coalescent time scale), we see that the factor influenced by η in Eq. (1) equals $\nu(t)^{-\eta} = \exp(\eta g t)$. This means that we can still interpret parameters assuming one reproduction model (model 1) leading to the coalescent (with scale parameter $\eta = x_1$) under the assumption of an alternative reproduction model (model 2 with scale parameter $\eta = x_2$) by simply re-scaling the exponential growth parameter g from model 1 as $g' = g\frac{\eta_1}{\eta_2}$. For instance, a growth rate of $2g$ in the Wright-Fisher model corresponds to a growth rate of g in the Moran model.

For our two MMC models, this also means that we could analyze the models based on alternative reproduction models. For instance, we set $\gamma = 1.5$ for the discrete reproduction model leading to the Psi-coalescent, but we could also choose any other $1 < \gamma < 2$. For the Beta-coalescent, there is indeed a very appealing alternative reproduction model due to Schweinsberg [Sch03]. This alternative model assumes, for $1 < \alpha < 2$, that each individual at each generation independently produces a number of offspring following a power law distribution with tail parameter α (infinite variance), and that the next generation (the individuals surviving long enough to reproduce) is sampled from these offspring. In this model, one unit of coalescent time corresponds to an order of $N^{\alpha-1}$ generations. As discussed above, if $\alpha > 1$, we can interpret any growth rate g when seeing the Beta-coalescent as the genealogy model based on the modified Moran model with growth rate $g' = \left(1 + \frac{1}{\alpha-1}\right) g$ under Schweinsberg's model.

A.3 Properties of the reproduction model underlying the Beta-coalescent

The modified Moran model with distribution given in Section 2.1 leading to the Beta($2-\alpha$,α)-coalescent was introduced in [HM13] and the properties of U have been additionally analyzed in [IM02]. U, or more precisely U_N since it depends on N, is distributed as the number of lineages merged at the first merger in a Beta($2-\alpha$,α)-coalescent starting with sample size N. Since when increasing the sample size, the first merger can only include more lineages, $U_N \leq U_M$ holds for $M \geq N$ (we can assume that, with increasing sample sizes, coalescent events just add branches to the tree from smaller sample sizes, see [Pit99]). This then also holds for the expected values, so $E(U_N) \leq E(U_M) \leq E(U_\infty) = \frac{\alpha}{\alpha-1}$, where U_∞ is the limit of U for $N \to \infty$. See [HM13, p.9] for the existence of the limit, whose properties including its mean are described on the cited page combined with [IM02, p.226], including its infinite variance. See Table A for some properties of U_N for different N and α, computed from the definition of U_N and the listed properties of its limit.

A.4 Mathematical derivation of the pseudolikelihood function Eq. (2)

We follow the derivation from [EBBF15, Eq. 11]. We want to compute the likelihood of seeing the observed unfolded SFS, noted $\xi = (\xi_1, \ldots, \xi_{n-1})$ under a given coalescent (here a Beta-n-coalescent or a Psi-n-

N	α	$E(U_N)$	$\sqrt{Var(U_N)}$	$P(U \leq x_{min}) \geq 0.99$
5000	1.1	6.54	46.87	62
10000	1.1	6.83	64.24	62
25000	1.1	7.20	97.26	62
∞	1.1	11.0	-	62
5000	1.5	2.96	9.39	15
10000	1.5	2.97	11.27	15
25000	1.5	2.98	14.29	15
∞	1.5	3.00	-	15
5000	1.9	2.11	1.39	4
10000	1.9	2.11	1.50	4
25000	1.9	2.11	1.64	4
∞	1.9	2.11	-	4

Table A: Properties of U_N for the modified Moran model underlying the Beta-coalescents. x_{min}: Minimal integer x such that $P(U \leq x) \geq 0.99$.

coalescent with exponential growth, but the derivation works for any coalescent model). Let $s_2 = \sum_{i=1}^{n-1} \xi_i$ be the number of observed segregating sites that can be oriented using an outgroup and thus that are included in the unfolded SFS. The index 2 of s_2 denotes that even with the outgroup there are 2 alleles. We assume the fixed-s approach, e.g. we assume that the distribution of the SFS is given by placing s mutations at random on the genealogical tree. Under the fixed-s assumption, the probability of observing the SFS is given by the multinomial distribution

$$P(\xi = (\xi_1, \ldots, \xi_{n-1})) = \mathbb{E}\left[\frac{s_2!}{\xi_1! \cdots \xi_{n-1}!} \prod_{i=1}^{n-1} \left(\frac{T_i}{T_{tot}}\right)^{\xi_i}\right], \tag{5}$$

since a segregating site has mutant allele frequency i if it lands on a branch that supports i leaves (T_i is the sum of lengths of branches supporting i leaves, $T_{tot} = \sum_{i=1}^{n-1} T_i$ is the total length of the genealogy). Under further assumptions of independence of the different fractions $\frac{T_i}{T_{tot}}$ of the total branch length and approximating $E\left(\frac{T_i}{T_{tot}}\right) \approx \frac{E(T_i)}{E(T_{tot})}$, we have a further approximation

$$P(\xi) = \frac{s_2!}{\xi_1! \cdots \xi_{n-1}!} \prod_{i=1}^{n-1} \left(\frac{E(T_i)}{E(T_{tot})}\right)^{\xi_i}, \tag{6}$$

Next, we consider the addition of a misorientation probability, e, describing the switch of ancestral and derived states. Eq. (6) constitutes a multinomial distribution, which can be interpreted as throwing s balls into compartments 1,. . . ,$n-1$, where compartment i is hit with probability $\frac{E(T_i)}{E(T_{tot})}$. Misorienting the allele in this interpretation means that a ball that originally lands in compartment i is placed in compartment $n-i$ instead. If this happens with probability e, a ball consequently lands in compartment i with probability $(1-e)\frac{E(T_i)}{E(T_{tot})} + e\frac{E(T_{n-i})}{E(T_{tot})}$. So the probability to observe a specific SFS when ancestral and derived types can be confused is

$$P(\xi) = \frac{s_2!}{\xi_1! \cdots \xi_{n-1}!} \prod_{i=1}^{n-1} \left(\frac{(1-e)E(T_i) + eE(T_{n-i})}{E(T_{tot})}\right)^{\xi_i}. \tag{7}$$

Simulations showed that inferring parameters via a pseudolikelihood approach based on Eq. 7 tends to overestimate e to fit the U-shape. To counteract this, we couple this equation with an alternative estimation of

e by using polymorphic sites discarded in the process of polarizing the SFS due to having a third allele in the outgroup. As described in [Lap17, Section 4.2] or [BD03, p. 1620], these sites carry information about e. Let $S_T = S_2 + S_3$ be the total number of bi- and tri-allelelic SNPs in the sample, where S_2 is the (random) number of sites where the outgroup does not show a third allele not observed in the sample (left and central trees in Figure A.2) and S_3 the number of sites where it does (right trees in Figure A.2). Note that s_2 is the observed outcome of S_2, the total sum of the observed SFS. Consider a polymorphic site in the sample

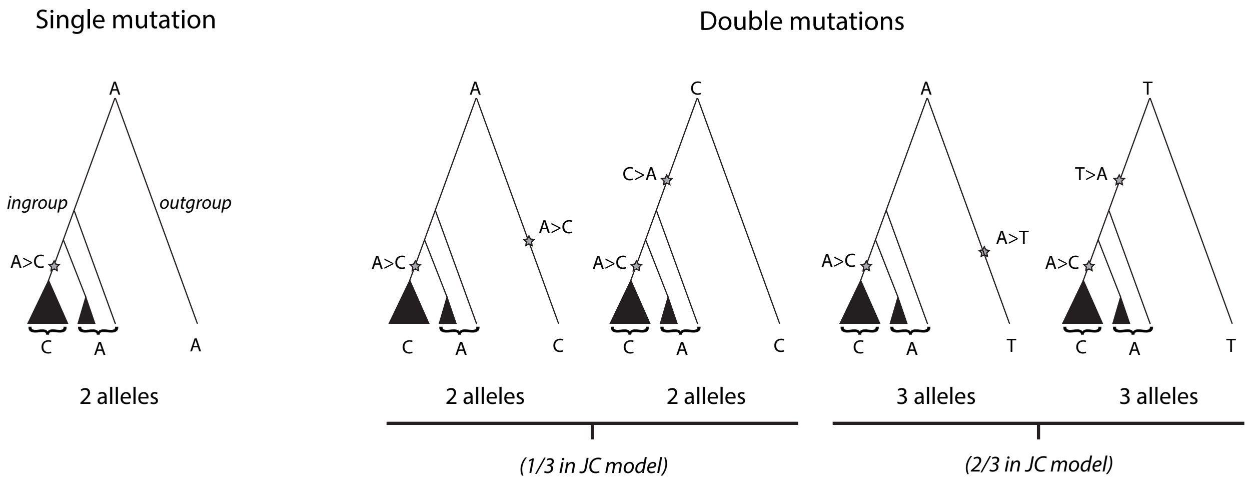

Figure A.2: Sketch of trees with mutations to illustrate how tri-allelic sites relates to the probability of misorientation. Left and central trees have one of their variant equal to the outgroup (counted in s_2) whereas the left trees have 3 differents alleles (counted in s_3). Under a Jukes and Cantor setting, the expected number of misoriented variants (central trees) equals half the number of tri-allelic sites.

(ingroup). If one of the alleles is the same allele as the sole allele present in the outgroup, there was either a single mutation (most likely for a closely related outgroup) or two mutations, the second one masking the effect of the first (central trees of Figure A.2). In the Jukes-Cantor model where all mutations are equally likely, if the probability of having a specific (second) mutation $X > Y$ masking the effect of the first is p (central trees in Figure A.2, then the probabilties of observing 2 alelles is p and the probability of observing 3 alleles is $2p$ (right trees of Figure A.2). We emphasise that while we assume an infinite sites model within the sample, we allow reverse mutations here due to the considerably longer branch lengths to the outgroup. Following this, we can compute the probability $P(S_3 = s_3|S_T = s_2 + s_3)$ that we observe exactly s_3 sites which are biallelic within the sample but have a third allele for the outgroup. This is simply binomial sampling from S_T biallelic sites with success probability $2p$. We can also express this probability in terms of the misorientation probability e. Let $v \in \xi$ be the event that a biallelic site (variant) can be polarized via outgroup (displaying one allele equal to the outgroup allele) and $mis(v)$ the event that the ancestral state of v is misidentified. The probability that a site of the SFS can be polarized is $1 - 2p$. We have

$$e = P(mis(v)|v \in \xi) = \frac{P(mis(v), v \in \xi)}{P(v \in \xi)} = \frac{p}{1-2p}, \tag{8}$$

and thus equivalently $p = \frac{e}{1+2e}$. This leads to

$$P(S_3 = s_3|S_T = s_2 + s_3) = \binom{s_T}{s_3}\left(\frac{2e}{1+2e}\right)^{s_3}\left(\frac{1}{1+2e}\right)^{s_2}. \tag{9}$$

We now assume a composite likelihood, multiplying Eqs. (7) and (9). Conditional on observing $s_2 + s_3$ segregating sites from which s_2 can be polarized via outgroup and form the SFS, the pseudo-likelihood of

observing a specific SFS is given by Eq. (2).

Remark A.1. *Following the discussion leading to Eq.* (9), S_2 *given* S_T *is binomially distributed with* S_T *draws with success probability* $1-2p=(1+2e)^{-1}$. *For our simulation approach, we want to set* S_T *to a reasonable value based on given values of* S_2 *and the mis-classification probability* e. *We define the most reasonable value* s_T *of* S_T*, given* $S_2=s_2$ *and* e*, as the value maximising* $P(S_2=s_2|S_T=s_T)$*, i.e. the value of* S_T *leading to the highest likelihood of observing* s_2. *These probabilities are given by the aforementioned binomial distribution. Thus, the maximal value is reached for* $\hat{S_T}=\lfloor(1+2e)s\rfloor$. *To see this, observe that*

$$\frac{P(S_2=s_2|S_T=s_T+1)}{P(S_2=s_2|S_T=s_T)}=\frac{s_T+1}{s_T+1-s_2}\frac{2e}{1+2e}\geq 1\Leftrightarrow S_T\leq s_2(1+2e)-1.$$

In words, as long as S_T *is smaller or equal than* $s_2(1+2e)-1$, *increasing it by +1 increases the likelihood, while as soon as it is bigger than this value, increasing it further will decrease the likelihood.*
We will use this estimate to simulate a reasonable s_3. *If we simulate a SFS with* s_2 *mutations, and we flip each mutation in it with frequency* e *from class* i *to* $n-i$*, we then simulate* s_3 *as a binomial draw from* $\hat{S}_T=\lfloor(1+2e)s\rfloor$ *Bernoulli r.v.'s with success probability* $\frac{2e}{1+2e}$. *This is denoted as the* $\hat{S}_T$ *approach.*

A.4.1 Taking into account transition-transversion bias

The approach in the section above assumes a potentially oversimplified Jukes-Cantor setting, where all mutations from one nucleotide to the other on the phylogenetic scale happen with equal rate. We assess how the approach changes if we assume, at least, different probabilities for transitions ($A\leftrightarrow G$ and $C\leftrightarrow T$) and transversions to appear in the sample (all other mutation patterns) [Kim80]. We define the mutation probability for a transition as p and set the probability for a transversion as κp, for $\kappa>0$. Eq. 8 then splits into two versions, one for transitions (ts) within sample ($\xi^{(ts)}$) and one for transversions (tv) within sample ($\xi^{(tv)}$). For a transition mutation within the sample, a third nucleotide only appears in the outgroup if there is one of the two transversions on the branch to the outgroup (probability $2\kappa p$ compared to p for the compensating transition). For transversions within the sample, this happens either if there is the possible transition mutation away from the ancestral state of the sample or the other transversion (probability $(\kappa+1)p$ compared to κp for a compensating transversion). This leads to the following equations

$$e_{ts}=P(mis(v)|v\in\xi^{(ts)})=\frac{P(mis(v),v\in\xi^{(ts)})}{P(v\in\xi^{(ts)})}=\frac{p}{1-2\kappa p}\Leftrightarrow p=\frac{e_{ts}}{1+2\kappa e_{ts}},\tag{10}$$

$$e_{tv}=P(mis(v)|v\in\xi^{(tv)})=\frac{P(mis(v),v\in\xi^{(tv)})}{P(v\in\xi^{(tv)})}=\frac{\kappa p}{1-(1+\kappa)p}\Leftrightarrow p=\frac{e_{tv}}{\kappa+(1+\kappa)e_{tv}},\tag{11}$$

which coincide and equal Eq. 8 for $\kappa=1$. Since we have two equations for p, we can express e.g. e_{ts} in terms of e_{tv} and κ or e_{tv} in terms of the other two parameters:

$$e_{ts}=\frac{e_{tv}}{\kappa+(1-\kappa)e_{tv}},\quad e_{tv}=\frac{\kappa e_{ts}}{1+(\kappa-1)e_{ts}},\quad \kappa=\frac{e_{tv}(1-e_{ts})}{e_{ts}(1-e_{tv})}\tag{12}$$

Analogously to the previous section, we can use these two equations to express the binomial probabilities that we see a certain number of sites with an additional, third nucleotide among all sites showing a transition respectively a transversion mutation within the sample. We will first state these equations in terms of the probability p to observe a transition on the phylogenetic path between sample and outgroup. For this, let $s_2^{(ts)}$, $s_3^{(ts)}$ be the sites observed showing a within-sample transition, where the former counts sites which show one of the two nucleotides observed in the outgroup and the latter a third, different nucleotide. Analogously,

we introduce $s_2^{(tv)}$, $s_3^{(tv)}$ for within-sample transversions. Recall that for a further nucleotide observed in the outgroup at a site with a within-sample transition, we need one of two possible transversion mutations.

$$P(S_3^{(ts)} = s_3^{(ts)} | S_T^{(ts)} = s_2^{(ts)} + s_3^{(ts)}) = \binom{s_T^{(ts)}}{s_3^{(ts)}} (2\kappa p)^{s_3^{(ts)}} (1 - 2\kappa p)^{s^{(ts)_2}}. \tag{13}$$

On the other hand, for a third nucleotide observed in the outgroup at a site showing a in-sample transversion, we need either a specific transition or a specific transversion.

$$P(S_3^{(tv)} = s_3^{(ts)} | S_T^{(tv)}) = \binom{s_T^{(tv)}}{s_3^{(tv)}} ((1+\kappa)p)^{s_3^{(tv)}} (1 - (1+\kappa)p)^{s_2^{(tv)}}. \tag{14}$$

Since, under the assumptions made here, the probability for the misidentification of the ancestral allele does depend on whether the within-sample segregation observed is due to a transition or to a transversion mutation, we also need to replace Eq. (7) by two separate equations for the SFS entries that are transversions within sample and those who are transitions (with analogous notation as introduced above)

$$P(\xi^{(ts)}) = \frac{s_2^{(ts)}!}{\xi_1^{(ts)}! \cdots \xi_{n-1}^{(ts)}!} \prod_{i=1}^{n-1} \left(\frac{(1-e_{ts})E(T_i) + e_{ts}E(T_{n-i})}{E(T_{tot})} \right)^{\xi_i^{(ts)}}, \tag{15}$$

$$P(\xi^{(tv)}) = \frac{s_2^{(tv)}!}{\xi_1^{(tv)}! \cdots \xi_{n-1}^{(tv)}!} \prod_{i=1}^{n-1} \left(\frac{(1-e_{tv})E(T_i) + e_{tv}E(T_{n-i})}{E(T_{tot})} \right)^{\xi_i^{(tv)}}, \tag{16}$$

To now get an analogous formulation of the pseudolikelihood function Eq. (2), we can multiply the four equations Eq. (13)- Eq. (16). Parameters to estimate can be reduced to the coalescent parameter and the growth parameter (via the expected values) plus two out of e_{ts}, e_{tv}, κ by plugging in p from Eq.'s (10) and (11) and using Eq. (12). Here below, we express it using e_{ts} and κ while replacing κ by its straightforward estimator $\hat{\kappa} = s_T^{(tv)}/2s_T^{(ts)}$. We thus optimise like before for three parameters: coalescent parameter α/Ψ, growth parameter g and transition misorientation probability e_{ts}:

$$\begin{aligned} &P(\xi, s_3) = \quad (17)\\ &\frac{s_2^{(ts)}!}{\xi_1^{(ts)}! \cdots \xi_{n-1}^{(ts)}!} \prod_{i=1}^{n-1} \left(\frac{(1-e_{ts})E(T_i) + e_{ts}E(T_{n-i})}{E(T_{tot})} \right)^{\xi_i^{(ts)}} \cdot \binom{s_T^{(ts)}}{s_3^{(ts)}} (2\hat{\kappa}\frac{e_{ts}}{1+2\hat{\kappa}e_{ts}})^{s_3^{(ts)}} (1 - 2\hat{\kappa}\frac{e_{ts}}{1+2\hat{\kappa}e_{ts}})^{s_2^{(ts)}} \cdot \\ &\cdot \frac{s_2^{(tv)}!}{\xi_1^{(tv)}! \cdots \xi_{n-1}^{(tv)}!} \prod_{i=1}^{n-1} \left(\frac{(1 - \frac{\hat{\kappa}e_{ts}}{1+(\hat{\kappa}-1)e_{ts}})E(T_i) + \frac{\hat{\kappa}e_{ts}}{1+(\hat{\kappa}-1)e_{ts}}E(T_{n-i})}{E(T_{tot})} \right)^{\xi_i^{(tv)}} \\ &\cdot \binom{s_T^{(tv)}}{s_3^{(tv)}} ((1+\hat{\kappa})\frac{e_{ts}}{1+2\hat{\kappa}e_{ts}})^{s_3^{(tv)}} (1 - (1+\hat{\kappa})\frac{e_{ts}}{1+2\hat{\kappa}e_{ts}})^{s_2^{(tv)}}. \end{aligned}$$

Alternatively, one could e.g. express the formula in terms of e_{ts} and e_{tv} and optimise over four parameters α/Ψ, g and these two misorientation probabilities.

Results for model selection and parameter estimation based on Eq. (17) are shown in Tables E and F. When κ is set to 1 in Eq. (17), only the binomial coefficients differ compared to Eq. (2). This matters during optimisation solely for comparing parameter sets with $e = 0$ (resp. $e_{ts} = 0$) with parameter sets with $e > 0$ ($e_{ts} > 0$), because for no misorientation error the binomial coefficients (and the whole binomial contributions) are not taking into account when evaluating the pseudolikelihoods. Hence optimisation under both equations is equivalent apart from these comparisons. Thus, we mostly have identical optimisation, but some small discrepancies between Table D and Tables E, F.

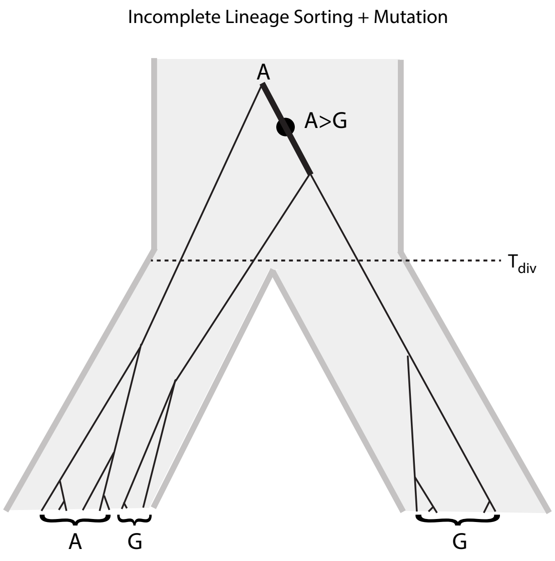

Figure A.3: Sketch of an ancestral polymorphism that causes an orientation errors. Both ILS and a mutation (here A to G) in one "problematic" branch (marked here in bold) must occur jointly.

A.5 Estimating the impact of ancestral polymorphism

To assess whether ancestral polymorphisms (Incomplete Lineage Sorting, ILS plus specific mutation events) may affect our estimation of orientation errors (Figure A.3), we compare estimated diversity *between* in- and outgroups (π_b) to estimated *within*-group diversity (π_w). We use a moment-based method to estimate T_{div}, the time of divergence between the two species of interest (ingroup and outgroup), assuming an instantaneous split and a constant coalescent rate N_e^{-1}. As we used genome-wide polymorphisms, moment estimators are likely to be unbiased and to have a low variance, as many pseudo-independent loci are averaged. Noting t time in generations and T when expressed in N_e generations, we have:

$$\begin{aligned} \mathbb{E}[\pi_w] &= 2 \cdot t_2 \cdot \mu \\ \mathbb{E}[\pi_b] &= 2 \cdot (t_2 + t_{div}) \cdot \mu \\ \frac{\mathbb{E}[\pi_b]}{\mathbb{E}[\pi_w]} - 1 &\approx \frac{t_{div}}{t_2} = T_{div}, \end{aligned} \tag{18}$$

where t_2 is the coalescence time of two sequences from the same group (in- or outgroup).
Thus, one can estimate the time (in coalescent time units) to the ancestral species using the moment estimator:

$$\hat{T}_{div} = \frac{\pi_b}{\pi_w} - 1$$

and from there compute the probability that the whole coalescent tree from the ingroup has a T_{MRCA} smaller than the estimated $\hat{T}_{div}$ using the full distribution of T_{MRCA}: $\mathbb{P}(ILS) = \mathbb{P}(T_{MRCA} > \hat{T}_{div})$. As our main interest is to check whether the presence of ILS may produce a spurious MMC signal although the biological properties would lead to a Kingman-based genealogy, we compare the estimate T_{div} with T_{MRCA} under a Kingman coalescent. We use the standard coalescent without exponential growth, since adding exponential growth shortens T_{MRCA} and we thus compare to the "worst case". The results are shown in Table H. To compute the probability for $T_{MRCA} > T_{div}$, we use that for sample size n, T_{MRCA} is a

sum of independent exponentially distributed random variables with rates $\binom{n}{2}, \binom{n-1}{2}, \ldots, 1$ and thus hypo-exponentially distributed with these parameters. We then take sample size as the maximum of sizes of in- and outgroup and compute $P(T_{MRCA} > T_{div})$ with the R package `sdprisk`.
From this conservative estimate of the genome that could have experienced ILS, only a small fraction of the polymorphic sites will results from a mutation in the "problematic" ancestral branch (see Figure A.3). As a comparison, for an average Kingman tree of $n = 50$ leaves, only a fraction $1/2a_n \approx 0.11$ (where $a_n = \sum_{i=1}^{n-1} i^{-1}$) of mutations will occur one of the branch connecting the MRCA. We note that ILS trees are typically longer than average, this number is quite conservative. Therefore, it is safe to assume that the fraction of sites that potentially will be mis-oriented due to ILS and mutations is at least one order of magnitude lower than the computed $\mathbb{P}(ILS)$.

A.6 Cramér's V as a goodness-of-fit measure

Our assumptions leading to Equations (6), (7) can be interpreted that each variant observed for the SFS is sampled from a multinomial distribution from the 'true' allele frequency spectrum. In the following, we denote the multinomial approximation of the SFS entry frequencies, the 'true' spectrum, by $(p_1, \ldots, p_{n-1})$. Since assuming sampling from a multinomial distribution is also the statistical model behind the χ^2 goodness-of-fit test, we chose the effect size measure Cramér's V [Cra16, ch. 21] of this test, defined as

$$V = \sqrt{\sum_{i=1}^{n-1} \frac{(o_i - p_i)^2}{p_i(n-2)}},$$

to quantify the lack of goodness of fit (o_i is the observed frequency of mutations with frequency i/n among all mutations). This measure can be interpreted as a dimensionless version of the χ^2 test statistic, since the mutation counts do not enter, just the mutation frequencies and the additional factor $n - 2$ corrects for unequal sample sizes.

A.7 Assessing estimation errors

A.7.1 Simulation and inference setup

As a rough approximation of a genome, we simulated 100 independent loci (ignoring the fine structure of weakly physically linked loci and long range LD, see Appendix A.10). This means that the genealogical trees of the loci are independent and follow the same tree distribution, e.g. realisations of a Beta coalescent with exponential growth with rate g and coalescent parameter α. The mutations on each tree are independent of all other trees (and mutations on them) and given by a Poisson process with rate $\frac{\theta}{2}$. We assumed three different sample sizes $n = 20$, $n = 25$ and $n = 100$. For each locus, we set the mutation rate so that on average 50 mutations appear, *i.e.* we set $\theta = 100/E[T_{tot}]$ (generalized Watterson estimate), where T_{tot} is the sum of all branch lengths of the locus' genealogy. Mutations are interpreted under the infinite-sites model, resulting in simulated SNP sequences (ancestral vs. derived type). For each SNP, we then flip ancestral and derived allele with probability e. We simulate 500 SNP sequences as described above for each combination of coalescent parameter α or Ψ, growth rate g and misorientation probability e from the following two sets (the first set has α, Ψ and g on the inference grid, the second uses off-grid values).

- Set 1: equidistant $\alpha \in \{1, 1.05, 1.1, \ldots, 2\}$ and $\Psi \in \{0.05, 0.1, 0.2, 0.3, 0.4, 0.5, 0.6, 0.7, 0.8, 0.9\}$
  Set 2: $\alpha \in \{1.025, 1.325\}$, $\Psi \in \{0.025, 0.075\}$ (additionally $\Psi = 0.005$ for $n = 25$)

- Set1: $g \in \{0, 0.5, 1, 10\}$, Set 2: $g \in \{0.25, 2.25, 11.25\}$

- Set 1: $e \in \{0, 0.01, 0.05, 0.1\}$ (essentially on grid), Set 2: $e \in \{0, 0.015, 0.045, 0.095\}$

To infer via Eq. (2), we also need the total number of segregating sites $s + s_{\neq}$, adding the number of segregating positions not included in the SFS due to not being able to polarize them. For this, we use the $\hat{S}_0$ approach described in Remark A.1.

A.7.2 Parameter and model selection accuracy

First, for $n = 20$ and $n = 100$, we estimate parameters using Eq. 2 using the same coalescent model (Beta or Psi) on equidistant grids with $\alpha \in \{1, 1.05, 1.1, \ldots, 2\}$ or $\Psi \in \{0, 0.5, 0.1, \ldots, 1\}$, $g \in \{0, 0.05, \ldots, 25\}$, $e \in \{0.001, 0.011, \ldots, 0.201\}$. For this, we only used the on-grid values (Set 1). Results are shown in Figures 1, A.4 − A.6,A.10 − A.21.
Second, we assess the error of our model selection approach based on approximated Bayes factors for $n \in \{20, 25, 100\}$. For this, we fixed different values of Ψ and α from Set 1 and Set 2 including $\alpha = 2$.

We then picked 2,000 simulations at random from all parameter combinations with this fixed coalescent parameter (as described above) and performed model selection via Bayes factors as described in the method described in the main document (section 2.2). The maximum was taken on the same equidistant grids as for the parameter estimation. Expanded results are provided in Tables B and C.

A.7.3 Parameter estimation accuracy - results

For inferring parameters under the Beta-coalescent or the Psi-coalescent, Figures 1, A.4–A.6 show the error distribution of all three parameters for $n \in \{20, 100\}$ across all simulation parameter choices. While g cannot be estimated precisely in some cases, e, Ψ and, to a lesser degree, α, can generally be estimated rather well, especially if sample size $n = 100$.

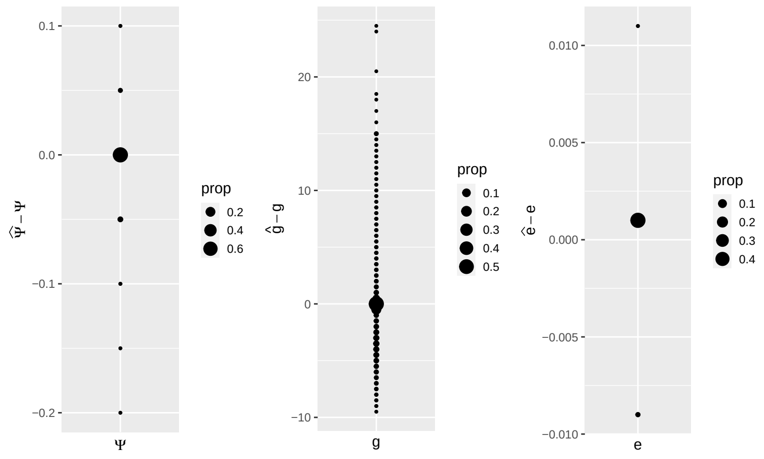

Figure A.4: Error for estimating parameters for Psi-coalescents ($n = 100$, with growth and misclassification) across all simulation scenarios

These errors distribute over the different parameter settings as shown in Figures A.10 − A.21. Most notably, large errors when estimating growth rates only happen if the growth rate is also large. For Psi-coalescents, we see that choosing Ψ between grid points are still mostly captured by the adjacent Ψ grid points.

sample size	true model	grid	Fraction model inferred as			
			Kingman	Beta	Psi	MMC
$n = 20$	$\alpha = 2$	yes	**0.77**	0.22	0.00	0.00
	$\alpha = 1.9$	yes	0.37	**0.58**	0.02	0.02
	$\alpha = 1.8$	yes	0.06	**0.79**	0.09	0.08
	$\alpha = 1.625$	no	0	**0.82**	0.09	0.08
	$\alpha = 1.025$	no		**0.99**	0.01	0
	$\alpha = 1$	yes		**0.99**	0	0
	$\Psi = 0.025$	no	0.14	**0.59**	0.15	0.12
	$\Psi = 0.05$	yes	0.01	0.17	**0.70**	0.11
	$\Psi = 0.075$	no		0.12	**0.82**	0.06
	$\Psi = 0.1$	yes		0.04	**0.94**	0.02
$n = 25$	$\alpha = 2$	yes	**0.79**	0.21		
	$\alpha = 1.9$	yes	0.34	**0.66**	0	0
	$\alpha = 1.8$	yes	0.02	**0.91**	0.04	0.03
	$\alpha = 1.625$	no		**0.9**	0.06	0.05
	$\alpha = 1.025$	no		**1**		0
	$\alpha = 1$	yes		**1**		0
	$\Psi = 0.005$	no	**0.55**	0.45		
	$\Psi = 0.025$	no	0.05	**0.72**	0.14	0.09
	$\Psi = 0.05$	yes	0	0.12	**0.82**	0.06
	$\Psi = 0.075$	no		0.06	**0.91**	0.03
	$\Psi = 0.1$	yes		0.02	**0.98**	0
$n = 100$	$\alpha = 2$	yes	**0.87**	0.13		
	$\alpha = 1.9$	yes	0.12	**0.88**		
	$\alpha = 1.8$	yes		**1**		
	$\alpha = 1.625$	no		**1**		
	$\alpha = 1.025$	no		**1**		
	$\alpha = 1$	yes		**1**		
	$\Psi = 0.025$	no		**0.92**	0.06	0.02
	$\Psi = 0.05$	yes			**1**	
	$\Psi = 0.075$	no			**1**	
	$\Psi = 0.1$	yes			**1**	

Table B: Model selection via two-step Bayes factor criterion. Based on 2,000 simulations for each true model assuming 100 loci with 50 observed mutations. For each simulation, the coalescent parameter is fixed and the growth parameter g and the allele misorientation rate e are randomly chosen ($g \in [0, 11.25]$, $e \in [0, 0.1]$). The column grid shows whether the parameters used for simulation were included in the inference grid. The maximum of each row is marked in bold. For details on both simulations and inference parameters see Appendix A.7. Fractions are rounded to two digits.

A.8 Visual correlations between the estimated parameters

We provide a graphical view of the correlation between the parameters inferred between the Kingman coalescent and the Beta-coalescent (Figure A.7) or between both MMC models (Figure A.8).

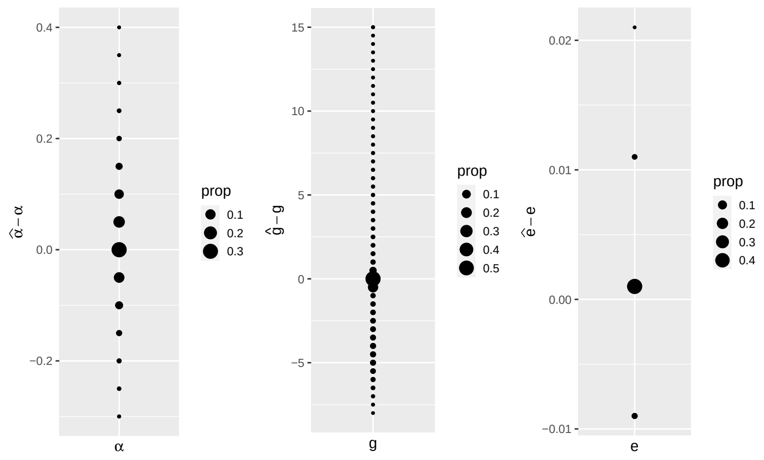

Figure A.5: Error for estimating parameters for Beta-coalescents ($n = 20$, with growth and misclassification) across all simulation scenarios

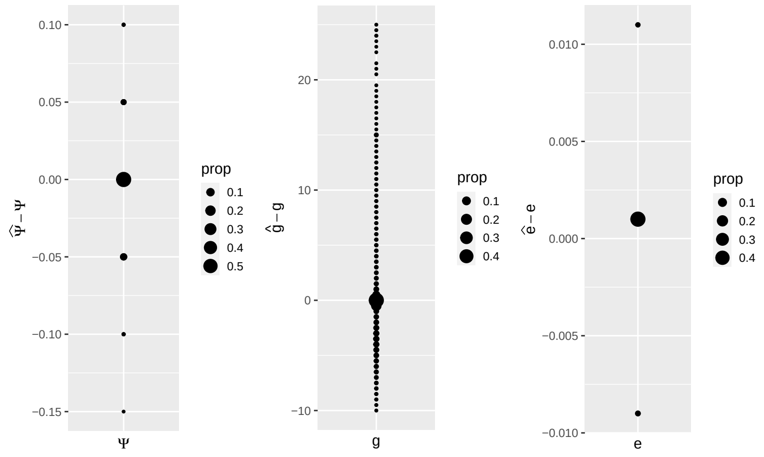

Figure A.6: Error for estimating parameters for Psi-coalescents ($n = 20$, with growth and misclassification) across all simulation scenarios

sample size	$n = 20$	$n = 100$
$\alpha = 1.75$	0.01	
$\alpha = 1.8$	0.04	
$\alpha = 1.85$	0.22	0.04
$\alpha = 1.9$	0.37	0.53
$\alpha = 1.95$	0.34	0.43
$\Psi = 0.05$	0.02	

Table C: Fractions of estimated parameters of model-misidentified coalescent simulations with $\alpha = 2$. If the two-step Bayes factor model inference recorded "MMC", the Beta parameter is reported.

A.9 Correction for GC-bias

We use the approach from [PATE18] and consider the subset of SNPs corresponding to $A \leftrightarrow T$ and $G \leftrightarrow C$ substitutions, which are not affected by biased gene conversion. We overlaid these neutralized SFS to the

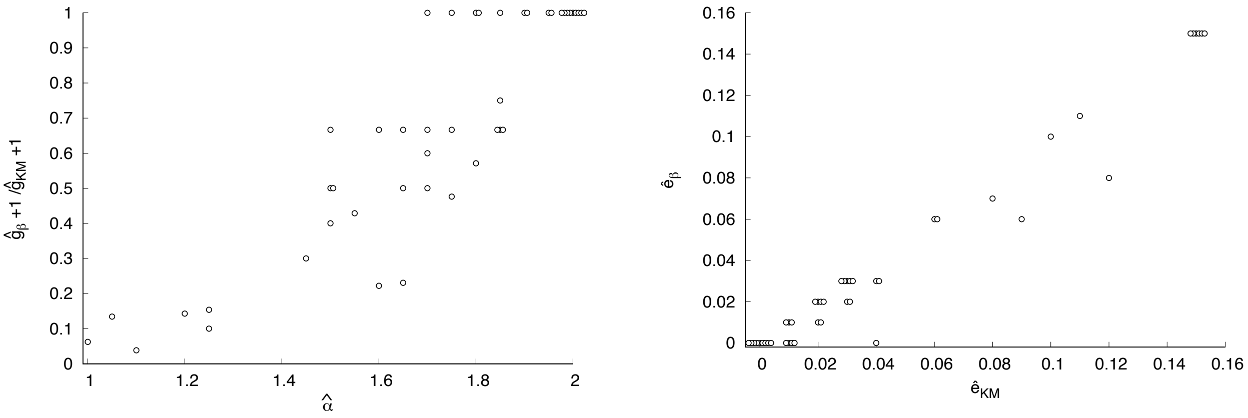

(a) Kingman model and Beta-coalescent model $\hat{g}+1$ ratio in function of $\hat{\alpha}$.

(b) Kingman model $\hat{e}$ in function of the corresponding Beta-coalescent allele misorientation parameter.

Figure A.7: Comparison of parameters between Kingman model and Beta-coalescent model.

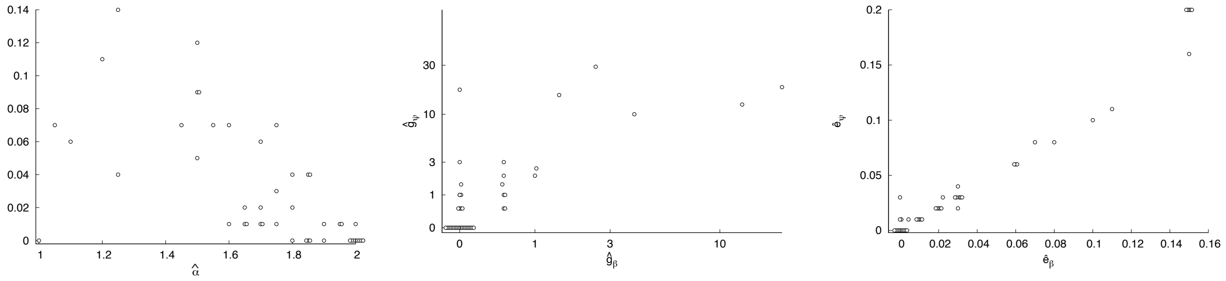

(a) α or Ψ

(b) g

(c) e

Figure A.8: Comparison of Beta-coalescent (x-axis) and Psi-coalescent (y-axis) parameters inferred from each SFS.

observed SFS and the predictions of the fitted models in supplementary files S1 Figs, S2 Figs.

A.10 Non-independence of unlinked loci under multiple merger genealogies

Here, we address the issue that physically unlinked loci in multiple merger genealogies still have dependent genetic diversity. For Λ-coalescents, which all our coalescent models are, the issue can be easily understood within the approximate multi-unlinked-locus model from the appendix of [Kos18]. In this model, multiple mergers result from large families appearing in a short amount of evolutionary time (see also a more thorough explanation in [MGF20]), so these families affect not only one, but all loci. Due to the model definition of MMC, each ancestral lineage can join one of such events with the same probability. Thus, if this probability is high, there will be a merger of similar size at each or nearly each locus in the genome, introducing a dependency between loci. The strength of this dependency should be correlated to the probability with which an ancestral lineage merges, following Remark 1 in [Kos18]. This probability x is generated by a Poisson process whose rate is proportional to $x^{-2}\Lambda(dx)$, where Λ is the associated measure of the coalescent (a Beta distribution or the point mass in Ψ for our model classes). This probability is rather small for Beta coalescents, but high for high Ψ values, see also Figure A.22.

A.11 Population structure scans

We performed two simple checks for population structure: PCA and `find.clusters` from the R package `adegenet` [JA11]. For PCA, we coded alleles as 0 and 1, imputed missing data as the mean allele at the site, to then perform a double-centered PCA: PCA, as implemented in `adegenet`, was performed on the SNP matrix after subtracting row means and column means (and adding the overall mean), see [GJQP+19, p.20]. The approach behind `find.clusters` is to first perform a standard PCA and then group individuals by running the k-means clustering algorithm on the principal component coordinates for different numbers of clusters. Based on the goodness-of-fit criterion BIC, we chose the 'optimal' k as the smallest value of k that is visibly a local minimum (essentially the elbow criterion). For large data sets of more than 1 million SNPs, we performed the analysis with a reduced data set by filtering down the number of SNPs by only retaining each xth SNP where $x = \frac{\#\ SNPs}{1000000}$, rounded to the lower integer. Results are shown in the supplementary files S3 Figs, S4 Figs (PCA results plotted, coloured according to DAPC clustering for 'optimal' k, BICs from DAPC) and Table H. For diploids, PCA and `find.clusters` results were not qualitatively affected by performing them on either haplotypes or diploid genotypes. For *D. melanogaster*, we performed the population structure scan separately on Chromosome 2L, 2R, 3L, 3R (with filtering down as described above, see supplementary file S5 Figs). For the human data, we omitted the X and Y chromosomes.

A.12 Nucleotide diversity across the genome

We recorded the (sample) mean and standard deviation of the per-site nucleotide diversity in non-overlapping windows of 15,000 sites along the genome (resp. the sequenced part of it). For computation, we used the R package `pegas` [Par10] and `vcftools` [DAA+11] (for haploid data presented as vcf files, we used J. Dutheil's fork of `vcftools` `https://github.com/jydu/vcftools`). For the human data, we omitted the X and Y chromosomes and for *D. melanogaster*, we used chromosomes 2L, 2R, 3L, 3R.
Results are shown in Table I and Figure A.23.

A.13 Effect of non-extreme demography on the SFS

The expected SFS $(E(S_1), \ldots, E(S_{n-1}))$ for a given genealogy tree (conditional on waiting times and topology) is a linear function of the waiting times C_k for the next coalescence event of the sample genealogy if k ancestral lineages are present, with coefficients $kP_{n,k}(i)$ dependent on the topology, where $P_{n,k}(i)$ is the probability that a random branch at level k has i descendants in the sample ([Fu95; FLW+17; SW08]). For a sample from a population with panmictic, neutral dynamics and finite variance in offspring number, corresponding to a Kingman coalescent where time is rescaled by a deterministic strictly monotonic function, all tree topologies are equally probable and independent of waiting times. The expected coefficients are given by $\mathrm{E}[kP_{n,k}(i)] = k\binom{n-i-1}{k-2}/\binom{n-1}{k-1}$. It should be noted that the assumption of monotonic time change ensures that the genealogy stays bifurcating: extreme changes in population size violate this and may lead to multiple merger genealogies.

The SFS for a large population described by the time-rescaled Kingman coalescent can be obtained as the large sample limit $n \to \infty$ of the above spectrum [FKR+18]. For large n, the probability that a random lineage at level k takes a fraction f of the descendants is $\mathrm{E}[P_{n,k}(fn)] \to (k-1)(1-f)^{k-2}df$. Hence the continuous expected SFS is given by equation (4), which depends on the expected population-level waiting times $c_k = \mathrm{E}[C_k] > 0$ for $k = 2 \ldots \infty$. The positivity of all coefficients in this expansion implies that for a finite expected TMRCA $\sum_{k=2}^{\infty} T_k < \infty$, the expected SFS for populations with non-extreme demography is an absolutely monotonic function of $1-f$ in $[0,1)$, and therefore a completely monotonic function of the frequency f in $(0,1]$. This is the case for all non-extreme demographies with bounded past population size,

since all of them have finite expected TMRCA. For small sample sizes n, see [SW08, Appendix B] for a similar argument explaining that demographic changes on top of the Kingman coalescent will still be monotonic.

A.14 Further figures and tables

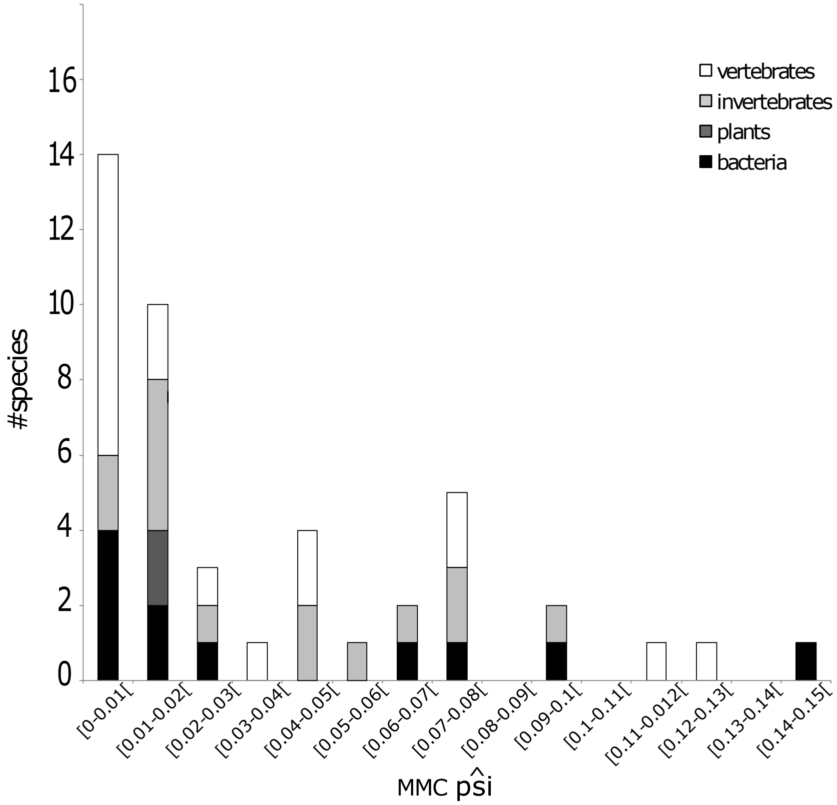

Figure A.9: Distribution of psi parameter in function of the order of the species (white: vertebrates, light grey: invertebrates, dark grey: plants, black: bacteria).

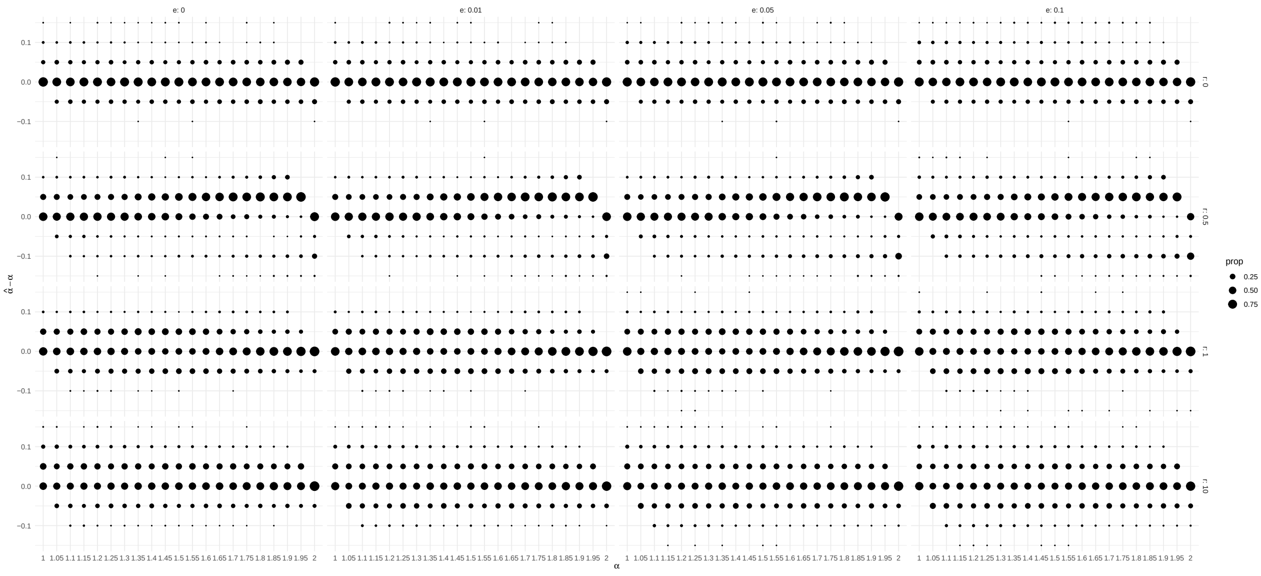

Figure A.10: Error for estimating coalescent parameter α for Beta-coalescents with growth and misclassification ($n = 100$). Growth rate is denoted by g.

Table D: Parameters Estimations

Species	g_{KM}	e_{KM}	V_{KM}	α	g_{Beta}	e_{Beta}	V_{Beta}	Ψ	g_{Ψ}	e_{Ψ}	V_{Ψ}	Model
Acinetobacter baumannii	0	0.1	0.064	1.8	0	0.1	0.059	0.02	0	0.1	0.061	Beta
Aptenodytes patagonicus	24	0.01	0.071	1.25	1.5	0	0.047	0.04	15.5	0.01	0.063	Beta
Arabidopsis thaliana	3.5	0.08	0.019	1.6	0	0.07	0.010	0.01	1	0.08	0.017	Beta
Armadillidium vulgare	0	0.03	0.089	1.7	0	0.03	0.069	0.06	0	0.02	0.083	Beta
Artemia franciscana	0.5	0.03	0.067	1.65	0	0.03	0.055	0.02	0.5	0.03	0.065	Beta
Athene cunicularia	2.5	0.03	0.040	1.8	1	0.03	0.037	0	2.5	0.03	0.040	Beta
Bacillus subtilis	5.5	0.15	0.085	1.25	0	0.15	0.079	0.14	0	0.2	0.062	Ψ
Caenorhabditis brenneri	1.5	0.09	0.094	1.5	0	0.06	0.086	0.09	0	0.06	0.105	Beta
Caenorhabditis elegans	0	0.06	0.142	2	0	0.06	0.1422	0	0	0.06	0.1422	KM
Chlamydia trachomatis	0	0.11	0.105	2	0	0.11	0.105	0	0	0.11	0.105	KM
Ciona intestinalis A	0	0.03	0.099	1.9	0	0.03	0.096	0.01	0	0.03	0.053	Beta
Ciona intestinalis B	0.5	0.03	0.135	1.6	0	0.02	0.071	0.07	0	0.02	0.078	Beta
Clostridium difficile	15	0.15	0.214	1	0	0.15	0.221	0	17.5	0.2	0.214	KM
Corvus cornix	1	0.01	0.032	1.85	0.5	0	0.024	0	1	0.01	0.032	Beta
Coturnix japonica	4	0.02	0.048	1.45	0.5	0.01	0.020	0.07	1.5	0.01	0.044	Beta
Culex pipiens	2.5	0.02	0.069	1.55	0.5	0.01	0.057	0.07	1	0.01	0.063	Beta
Drosophila melanogaster	5.5	0.02	0.019	1.65	0.5	0.02	0.005	0.01	3	0.02	0.017	Beta
Egretta garzetta	0	0.02	0.055	1.75	0	0.02	0.037	0.07	0	0.02	0.039	Beta
Emys orbicularis	0.5	0	0.068	1.85	0	0	0.060	0.04	0	0	0.059	KM
Escherichia coli	0	0.06	0.054	2	0	0.06	0.054	0	0	0.06	0.054	KM
Ficedula albicollis	0.5	0.01	0.029	2	0.5	0.01	0.029	0.01	0.5	0.01	0.028	Ψ
Gorilla gorilla gorilla	0	0	0.042	1.9	0	0	0.040	0	0	0	0.042	Beta
Halictus scabiosae	0	0.01	0.069	1.85	0	0.01	0.064	0.04	0	0.01	0.062	MMC
Helicobacter pilori	1	0.15	0.052	1.65	0	0.15	0.060	0.01	1	0.2	0.050	Ψ
Homo sapiens	0.5	0.01	0.010	1.85	0	0	0.011	0	0.5	0.01	0.010	Beta
Klebsiella pneumoniae	18.5	0.15	0.122	2	18.5	0.15	0.126	0	18.5	0.16	0.122	KM
Lepus granatensis	0.5	0.04	0.102	1.5	0	0.03	0.069	0.12	0	0.03	0.066	MMC
Melitaea cinxia	1.5	0.04	0.061	1.7	0.5	0.03	0.059	0.01	2	0.04	0.061	Beta
Messor barbarus	0.5	0	0.069	2	0.5	0	0.069	0	0.5	0	0.069	KM
Mycobacterium tubercolosis	25	0.01	0.118	1.05	2.5	0	0.090	0.07	29	0	0.126	Beta
Nipponia nippon	0	0.03	0.160	2	0	0.03	0.160	0	0	0.03	0.160	KM
Ostrea edulis	0	0.02	0.052	1.8	0	0.02	0.044	0.04	0	0.02	0.042	MMC
Pan paniscus	2	0	0.068	1.85	1	0	0.056	0	2	0	0.068	Beta
Pan troglodytes ellioti	0.5	0	0.052	1.7	0	0	0.028	0.02	0.5	0	0.045	Beta
Parus major	0.5	0.01	0.031	1.75	0	0.01	0.010	0.03	0	0.01	0.022	Beta
Parus caeruleus	6	0.04	0.062	1.2	0	0	0.037	0.11	1.5	0.03	0.031	MMC
Passer domesticus	0	0	0.022	2	0	0	0.022	0	0	0	0.022	KM
Phylloscopus trochilus	12.5	0	0.022	2	12.5	0	0.022	0	12.5	0	0.022	KM
Physa acuta	1	0.03	0.068	1.5	0	0.02	0.035	0.05	0.5	0.03	0.055	Beta
Pseudomonas aeruginosa	25	0.15	0.073	1.1	0	0.15	0.063	0.06	3	0.2	0.050	Ψ
Sepia officinalis	0	0.02	0.091	1.95	0	0.02	0.090	0.01	0	0.02	0.090	KM
Staphylococcus aureus	1	0.15	0.054	1.7	0	0.15	0.059	0.01	1	0.2	0.055	Ψ
Streptococcus pneumoniae	1	0.12	0.103	1.5	0	0.08	0.099	0.09	0	0.08	0.102	Beta
Taeniopygia guttata	9.5	0	0.034	1.75	4	0	0.019	0.01	10	0	0.030	Beta
Zea mays	0	0	0.033	1.95	0	0	0.031	0.01	0	0	0.030	Ψ

Table E: Parameters Estimations using Eq. (17). First line per data set: Using plugin estimate for κ. Second line: $\kappa = 1$, corresponds to main text analysis

Species	κ	α	g_{Beta}	$e_{ts,\text{Beta}}$	$e_{tv,\text{Beta}}$	Ψ	g_{Ψ}	$e_{ts,\Psi}$	$e_{tv,\Psi}$	Model
Acinetobacter baumannii	0.164	1.8	0	0.15	0.028	0.02	0	0.2	0.039	Ψ
Acinetobacter baumannii	1	1.8	0	0.1	0.1	0.02	0	0.1	0.1	Beta
Aptenodytes patagonicus	0.217	1.45	4	0.02	0.004	0.04	16.5	0.02	0.004	Beta
Aptenodytes patagonicus	1	1.25	1.5	0	0	0.04	15.5	0.01	0.01	Beta
Arabidopsis thaliana	0.438	1.6	0	0.11	0.051	0.01	1	0.12	0.056	Beta
Arabidopsis thaliana	1	1.6	0	0.07	0.07	0.01	1	0.08	0.08	Beta
Armadillidium vulgare	0.27	1.7	0	0.07	0.02	0.06	0	0.06	0.017	Beta
Armadillidium vulgare	1	1.7	0	0.03	0.03	0.06	0	0.02	0.02	Beta
Artemia franciscana	0.212	2	1	0.09	0.021	0	1	0.09	0.021	KM
Artemia franciscana	1	1.65	0	0	0	0.02	0.5	0.03	0.03	Beta
Athene cunicularia	0.307	1.95	2.5	0.06	0.019	0	3	0.06	0.019	Beta
Athene cunicularia	1	1.8	1	0.03	0.03	0	2.5	0.03	0.03	Beta
Bacillus subtilis	0.211	1.25	0	0.15	0.036	0.14	0	0.2	0.05	Ψ
Bacillus subtilis	1	1.25	0	0.15	0.15	0.14	0	0.2	0.2	Ψ
Caenorhabditis brenneri	0.106	1.5	0	0.15	0.018	0	2	0.2	0.026	Ψ
Caenorhabditis brenneri	1	1.5	0	0.06	0.06	0.09	0	0.06	0.06	Beta
Caenorhabditis elegans	0.294	2	0	0.1	0.032	0	0	0.1	0.032	KM
Caenorhabditis elegans	1	2	0	0.04	0.04	0	0	0.04	0.04	KM
Chlamydia trachomatis	0.098	2	0	0.15	0.017	0	0	0.2	0.024	KM
Chlamydia trachomatis	1	2	0	0.11	0.11	0	0	0.11	0.11	KM
Ciona intestinalis A	0.206	1.9	0	0.1	0.022	0.01	0	0.1	0.022	Beta
Ciona intestinalis A	1	1.9	0	0.03	0.03	0.01	0	0.03	0.03	Beta
Ciona intestinalis B	0.2	1.6	0	0.06	0.013	0.03	0.5	0.08	0.017	Beta
Ciona intestinalis B	1	1.6	0	0.02	0.02	0.07	0	0.02	0.02	Beta
Clostridium difficile	0.082	1.2	0	0	0	0.2	0	0.2	0.02	MMC
Clostridium difficile	1	1	0	0.15	0.15	0	17.5	0.2	0.2	MMC
Corvus cornix	0.225	2	1	0.02	0.005	0	1	0.02	0.005	KM
Corvus cornix	1	1.85	0.5	0	0	0	1	0.01	0.01	Beta
Coturnix japonica	0.245	1.5	0.5	0.04	0.01	0.05	2.5	0.05	0.013	Beta
Coturnix japonica	1	1.3	0	0	0	0.06	1.5	0.02	0.02	Beta
Culex pipiens	0.156	1.7	1	0.04	0.006	0.04	2	0.05	0.008	Beta
Culex pipiens	1	1.55	0.5	0	0	0.05	1.5	0.02	0.02	Beta
Drosophila melanogaster	0.421	1.65	0.5	0.03	0.013	0.01	3	0.04	0.017	Beta
Drosophila melanogaster	1	1.65	0.5	0.02	0.02	0.01	3	0.02	0.02	Beta
Egretta garzetta	0.187	1.75	0	0.07	0.014	0.07	0	0.07	0.014	Beta
Egretta garzetta	1	1.75	0	0.02	0.02	0.07	0	0.02	0.02	Beta
Emys orbicularis	0.127	1.85	0	0	0	0.04	0	0	0	KM
Emys orbicularis	1	1.85	0	0	0	0.04	0	0	0	KM
Escherichia coli	0.156	2	0	0.15	0.027	0	0	0.18	0.033	KM
Escherichia coli	1	2	0	0.06	0.06	0	0	0.06	0.06	KM
Ficedula albicollis	0.267	2	0.5	0.03	0.008	0	0.5	0.03	0.008	KM
Ficedula albicollis	1	1.85	0	0	0	0.01	0.5	0.01	0.01	Beta
Gorilla gorilla gorilla	0.206	1.9	0	0	0	0	0.5	0.02	0.004	Beta
Gorilla gorilla gorilla	1	1.9	0	0	0	0	0	0	0	Beta
Halictus scabiosae	0.127	1.85	0	0.05	0.007	0.03	0	0.05	0.007	MMC
Halictus scabiosae	1	1.85	0	0	0	0.04	0	0	0	MMC
Helicobacter pilori	0.044	1.6	0	0	0	0.05	0	0	0	Beta
Helicobacter pilori	1	1.65	0	0.15	0.15	0.01	1	0.2	0.2	Ψ

Table F: Parameters Estimations using Eq. (17). First line per data set: Using plugin estimate for κ. Second line: $\kappa = 1$, corresponds to main text analysis. *: run on a subset of contigs due to computational constraints, including the majority of SNPs

Species	κ	α	g_{Beta}	$e_{ts,\text{Beta}}$	$e_{tv,\text{Beta}}$	Ψ	g_{Ψ}	$e_{ts,\Psi}$	$e_{tv,\Psi}$	Model
Homo sapiens (YRI)	0.235	1.85	0	0.01	0.002	0	0.5	0.02	0.005	Beta
Homo sapiens (YRI)	1	1.85	0	0	0	0	0.5	0.01	0.01	Beta
Klebsiella pneumoniae	0.175	1.4	0	0.15	0.03	0	18.5	0.2	0.042	Beta
Klebsiella pneumoniae	1	2	18.5	0.15	0.15	0	18.5	0.16	0.16	KM
Lepus granatensis	0.117	1.5	0	0.07	0.009	0.11	0	0.06	0.007	MMC
Lepus granatensis	1	1.55	0	0	0	0.12	0	0	0	Ψ
Melitaea cinxia	0.169	2	1.5	0.09	0.016	0	1.5	0.09	0.016	KM
Melitaea cinxia	1	1.55	0	0	0	0.03	1	0.03	0.03	Beta
Messor barbarus	0.046	2	0.5	0	0	0	0.5	0	0	KM
Messor barbarus	1	2	0.5	0	0	0	0.5	0	0	KM
Mycobacterium tubercolosis	0.624	1.05	2.5	0	0	0.07	30	0.01	0.006	Beta
Mycobacterium tubercolosis	1	1.05	2.5	0	0	0.07	29	0	0	Beta
Nipponia nippon	0.222	2	0	0.09	0.021	0	0	0.09	0.021	KM
Nipponia nippon	0.222	2	0	0.09	0.021	0	0	0.09	0.021	KM
Ostrea edulis	0.18	1.85	0	0.06	0.011	0.04	0	0.06	0.011	MMC
Ostrea edulis	1	1.8	0	0	0	0.05	0	0	0	MMC
Pan paniscus	0.227	2	2	0.01	0.002	0	2	0.01	0.002	KM
Pan paniscus	1	1.85	1	0	0	0	1.5	0	0	Beta
Pan troglodytes ellioti	0.232	1.7	0	0	0	0.02	0.5	0	0	Beta
Pan troglodytes ellioti	1	1.7	0	0	0	0.02	0.5	0	0	Beta
Parus major	0.26	1.75	0	0.02	0.005	0.01	0.5	0.02	0.005	Beta
Parus major	1	1.75	0	0.01	0.01	0.03	0	0	0	Beta
Parus caeruleus	0.201	1.2	0	0	0	0.11	1.5	0.04	0.008	MMC
Parus caeruleus	1	1.2	0	0	0	0.13	1	0.01	0.01	Beta
Passer domesticus	0.293	2	0	0	0	0	0	0	0	KM
Passer domesticus	1	2	0	0	0	0	0	0	0	KM
Phylloscopus trochilus	0.242	2	8.5	0	0	0	8.5	0	0	KM
Phylloscopus trochilus	1	2	8.5	0	0	0	8.5	0	0	KM
Physa acuta	0.242	1.5	0	0.05	0.013	0.05	0.5	0.07	0.018	Beta
Physa acuta	1	1.5	0	0.02	0.02	0.05	0.5	0.03	0.03	Beta
Pseudomonas aeruginosa	0.191	1.1	0	0.15	0.033	0.06	3	0.2	0.046	Ψ
Pseudomonas aeruginosa	1	1.1	0	0.15	0.15	0.06	3	0.2	0.2	Ψ
Sepia officinalis	0.153	1.95	0	0.09	0.015	0.01	0	0.09	0.015	KM
Sepia officinalis	1	1.95	0	0.02	0.02	0.01	0	0.02	0.02	KM
Staphylococcus aureus	0.237	1.65	0	0.15	0.04	0.01	0.5	0.2	0.056	Ψ
Staphylococcus aureus	1	1.7	0	0.15	0.15	0.01	1	0.2	0.2	Ψ
Streptococcus pneumoniae	0.174	1.5	0	0.15	0.03	0.09	0	0.2	0.042	Ψ
Streptococcus pneumoniae	1	1.5	0	0.08	0.08	0.09	0	0.08	0.08	Beta
*Taeniopygia guttata**	0.283	1.75	4.5	0	0	0	12.5	0.01	0.003	Beta
*Taeniopygia guttata**	1	1.75	4.5	0	0	0.01	11.5	0	0	Beta
Zea mays	0.388	1.95	0	0	0	0.01	0	0	0	Ψ
Zea mays	1	1.95	0	0	0	0.01	0	0	0	Ψ

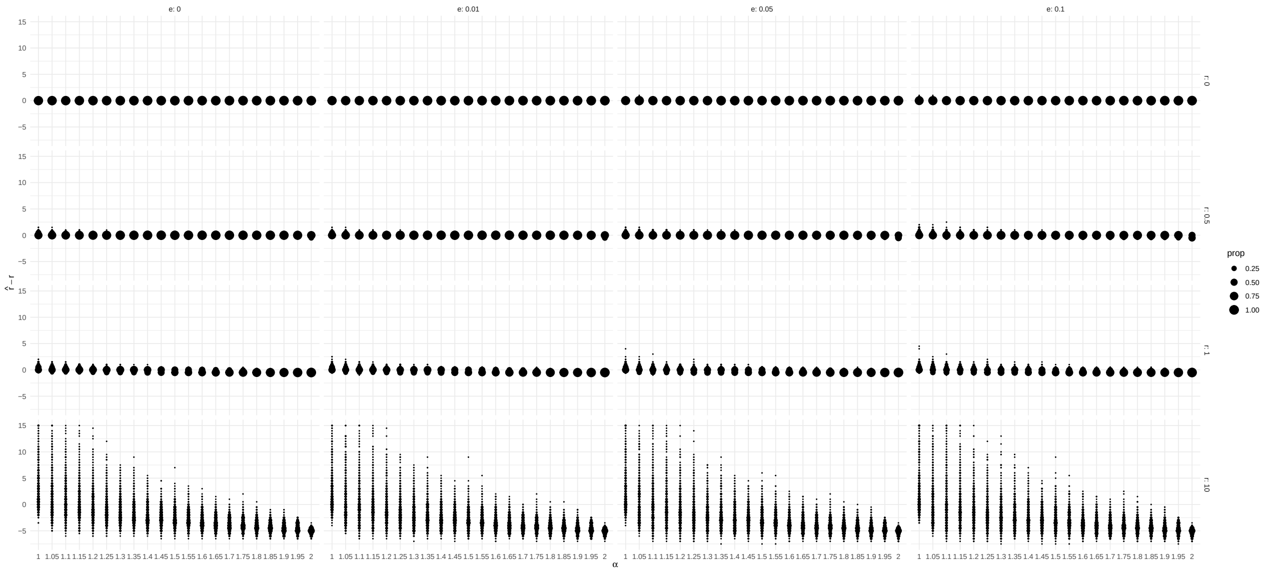

Figure A.11: Error for estimating growth rate g for Beta-coalescents with growth and misclassification ($n = 100$)

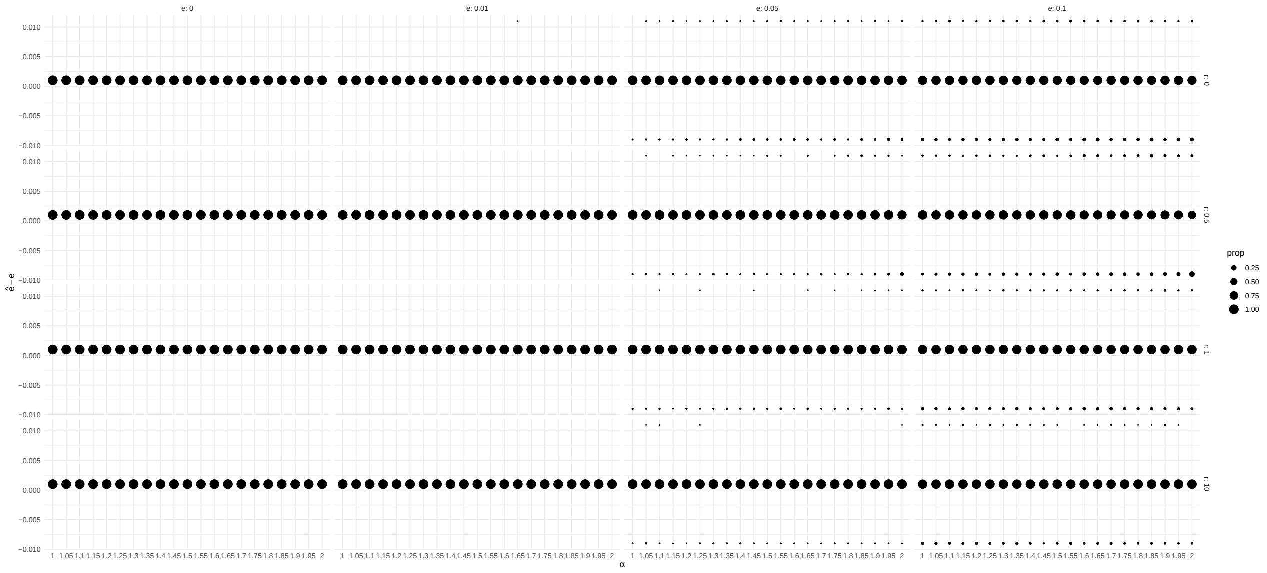

Figure A.12: Error for estimating misorientation rate e for Beta-coalescents with growth and misclassification ($n = 100$). Growth rate is denoted by g.

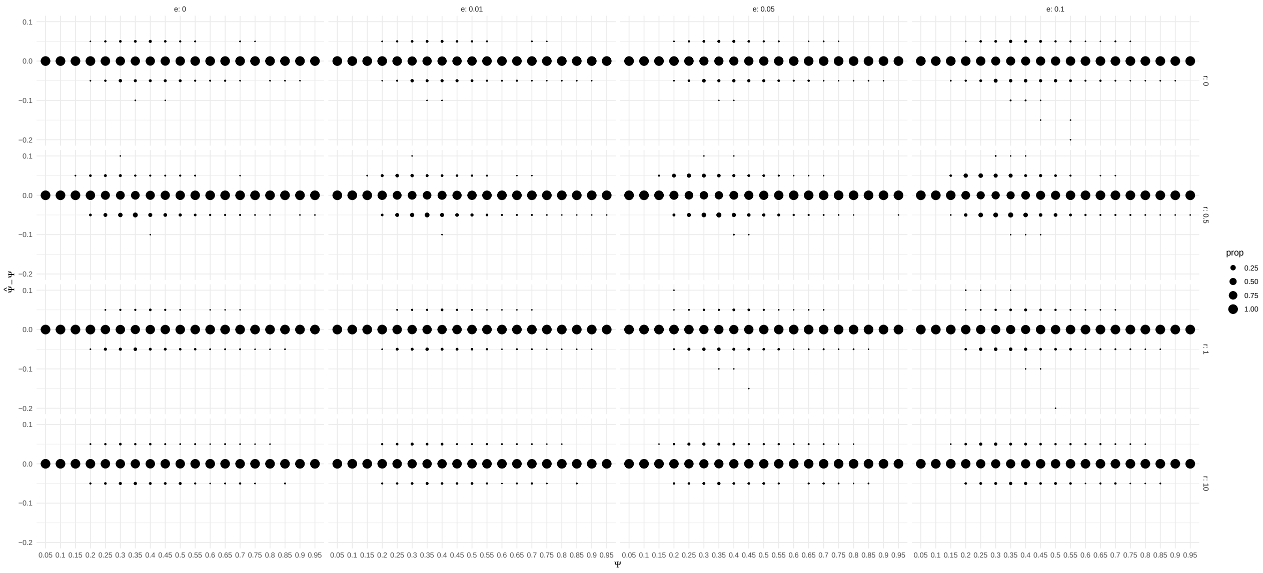

Figure A.13: Error for estimating coalescent parameter Ψ for Psi-coalescents with growth and misclassification ($n = 100$). Growth rate is denoted by g.

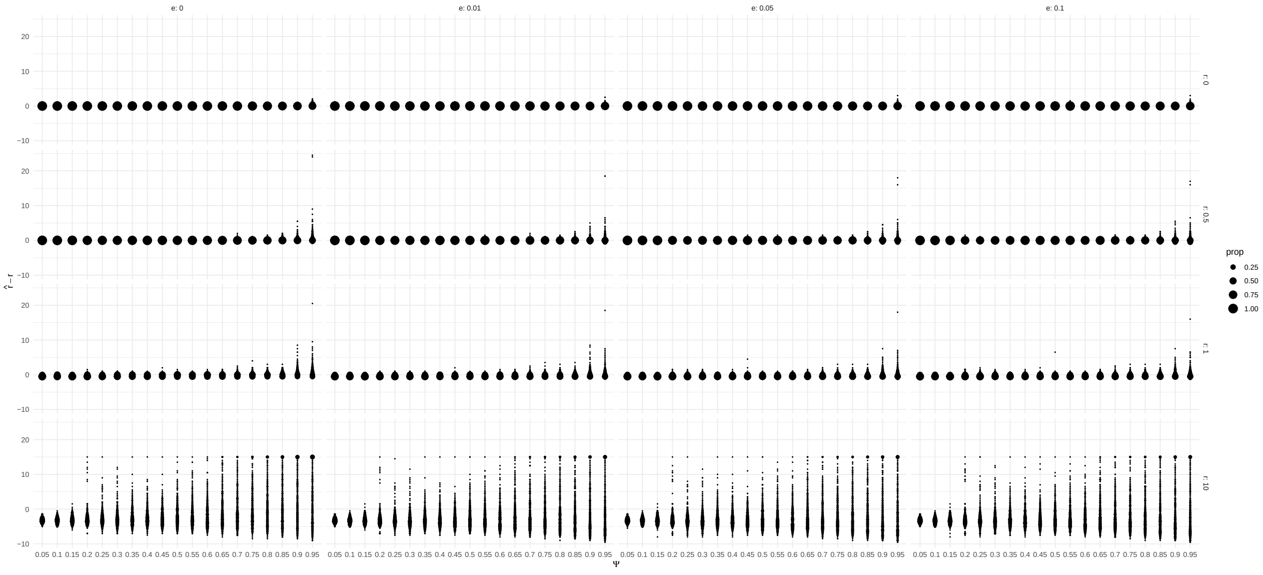

Figure A.14: Error for estimating growth rate g for Psi-coalescents with growth and misclassification ($n = 100$).

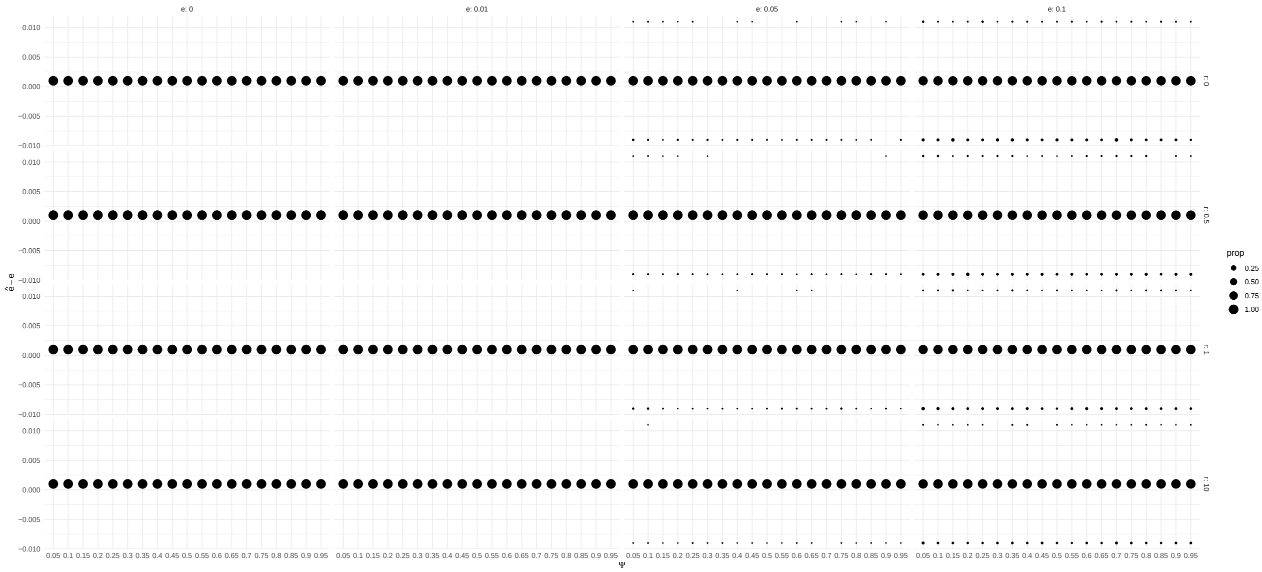

Figure A.15: Error for estimating misorientation rate e for Psi-coalescents with growth and misclassification ($n = 100$). Growth rate is denoted by g.

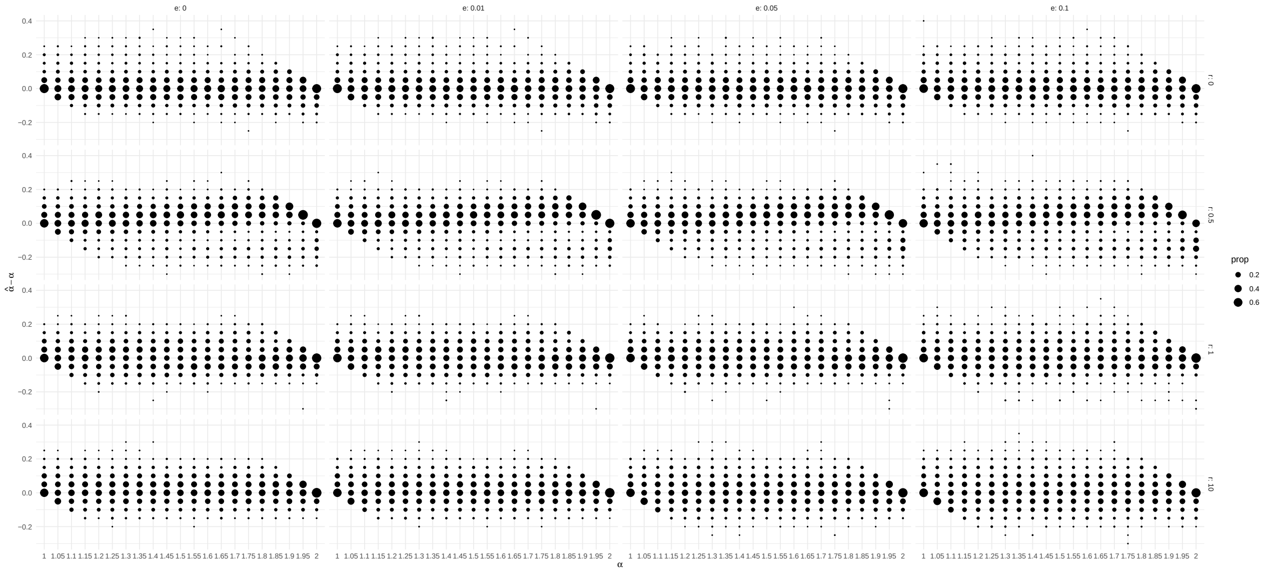

Figure A.16: Error for estimating coalescent parameter α for Beta coalescents with growth and misclassification ($n = 20$). Growth rate is denoted by g.

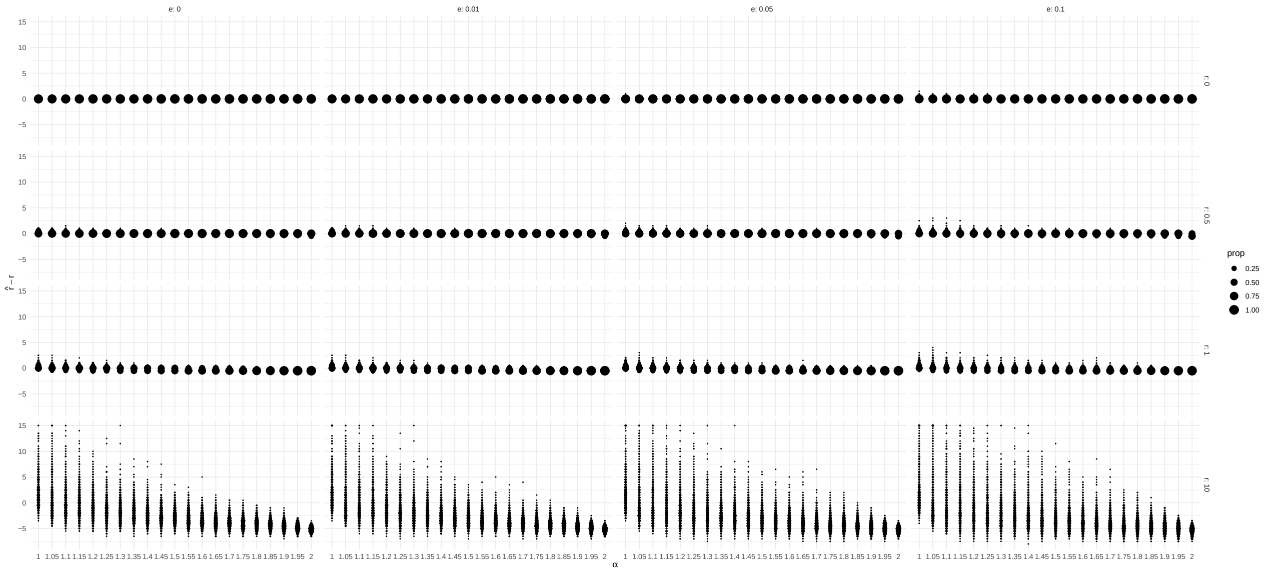

Figure A.17: Error for estimating growth rate g for Beta-coalescents with growth and misclassification ($n = 20$)

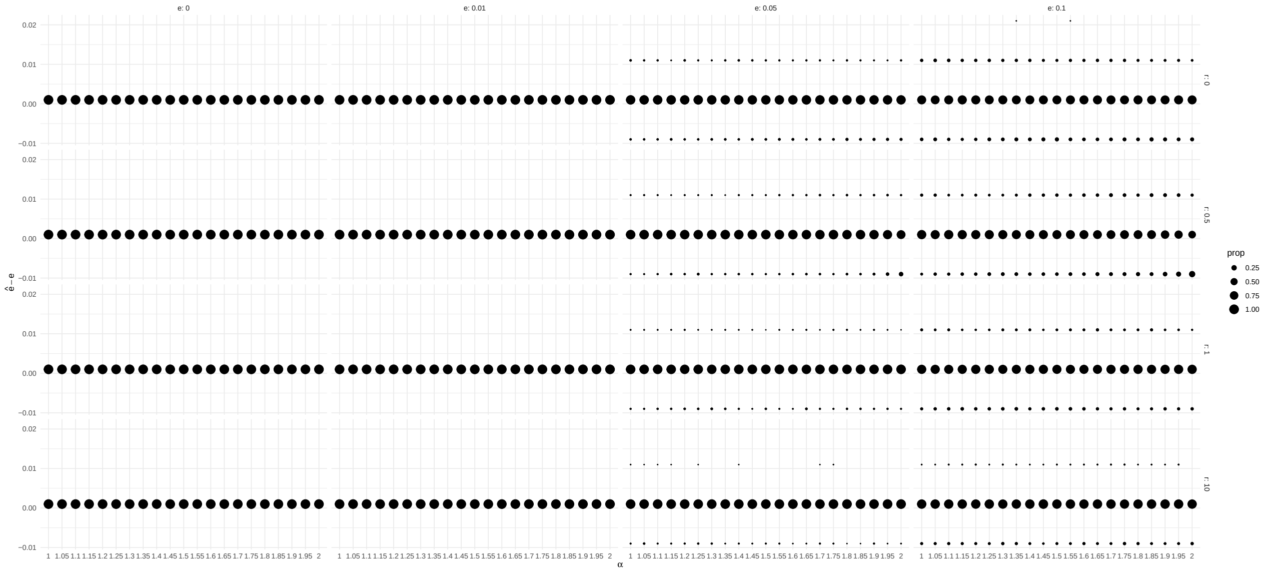

Figure A.18: Error for estimating misorientation rate e for Beta-coalescents with growth and misclassification ($n = 20$). Growth rate is denoted by g.

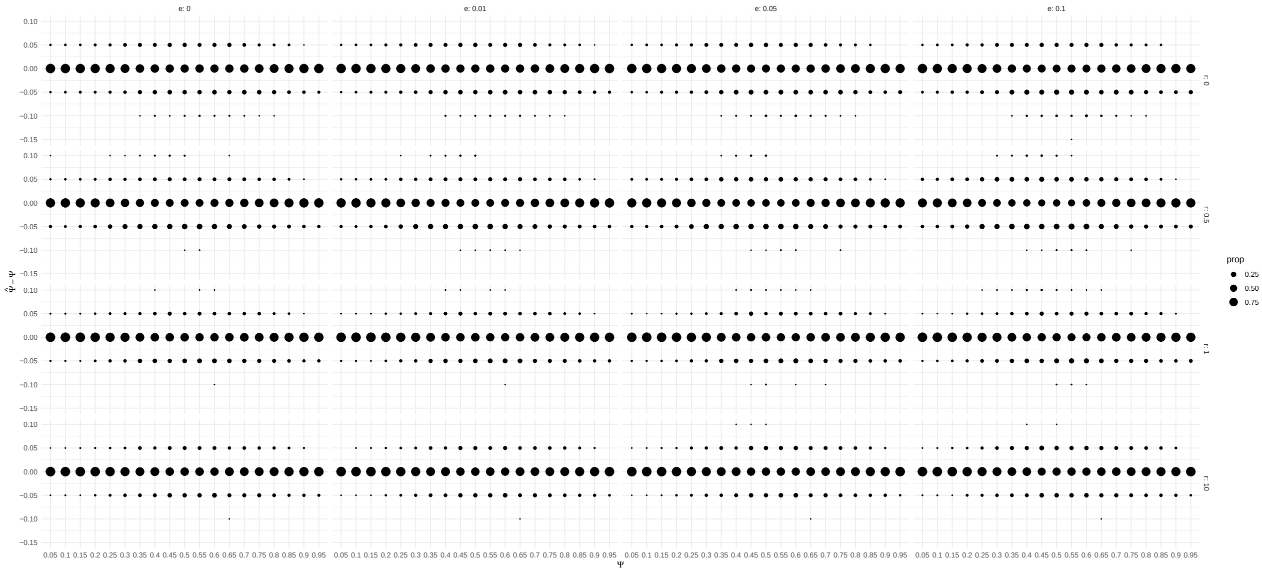

Figure A.19: Error for estimating coalescent parameter Ψ for Psi-coalescents with growth and misclassification ($n = 20$). Growth rate is denoted by g.

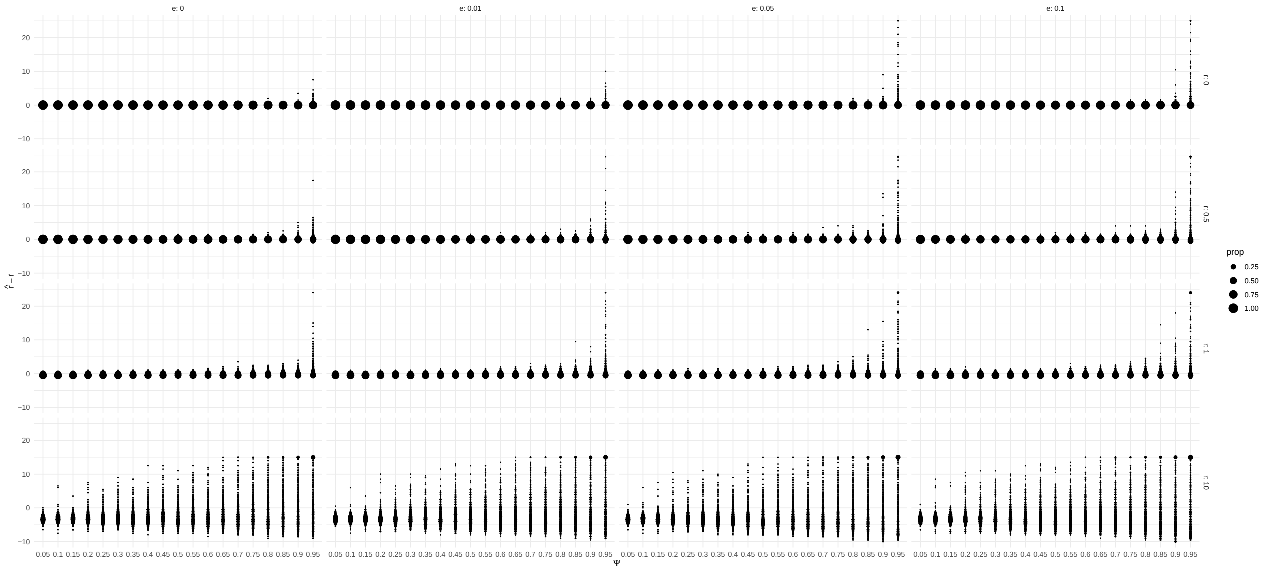

Figure A.20: Error for estimating growth rate g for Psi-coalescents with growth and misclassification ($n = 20$).

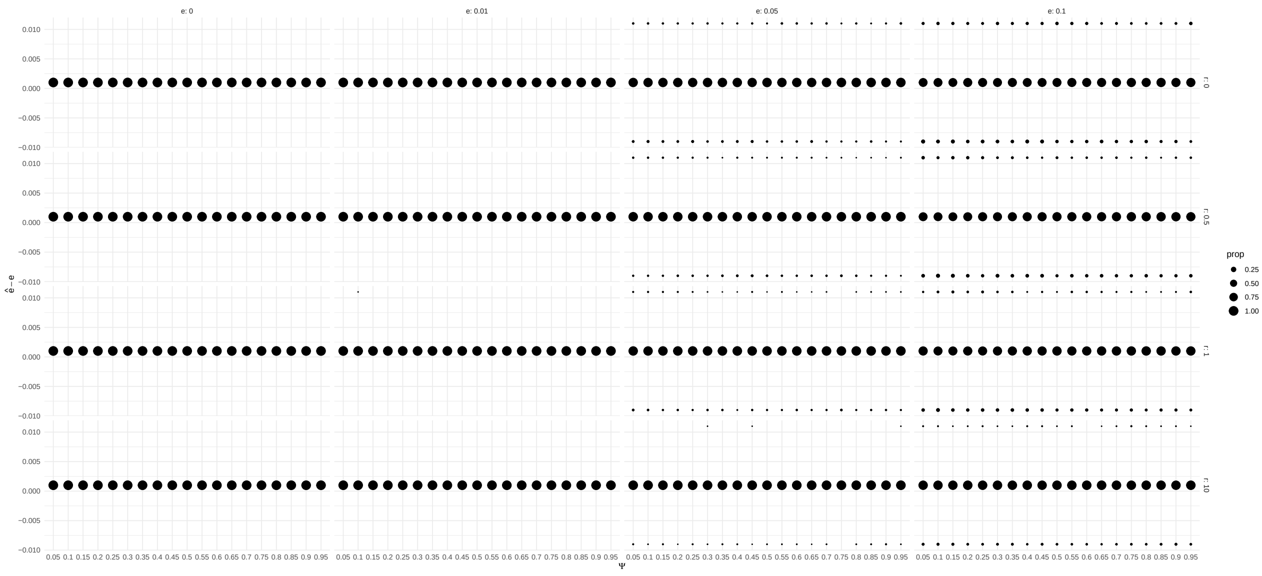

Figure A.21: Error for estimating misorientation rate e for Psi-coalescents with growth and misclassification ($n = 20$). Growth rate is denoted by g.

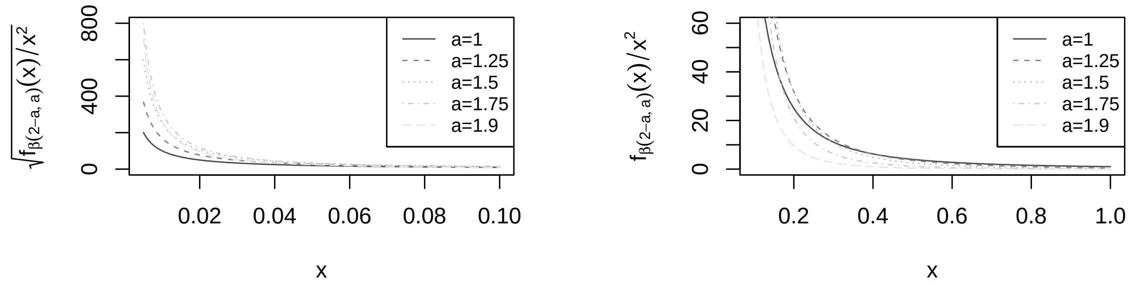

(a) (Improper) distribution of mergP x close to 0

(b) (Improper) distribution of bigger mergP x

Figure A.22: Distribution of merger rates of Beta-coalescents: Each lineage merges with merger probability x (abbeviated as mergP), where x is chosen with rate $x^{-2} * \Lambda(dx)$, where Λ is a Beta distribution with parameters $2 - a$ and a. Mergers only are realized if at least two lineages merge. The figures depict the corresponding (improper) density $x^{-2} * f_\beta(2 - a, a)$, where f_β is the density of the Beta distribution used. The detailed (Poisson) construction can be found in [Pit99].

Table G: Data set information

Species	Outgroup	n	Outgroup size	Polarized SNPs	$\neq$ outgroup	Diallelic# outgroup	Source	Outgroup source (or same as sample)
Acinetobacter baumannii	*A. nosocomialis*	79	1	78175	6006		[RdSB+18]*	NCBI RefSeq• - Nov 2016
Aptenodytes patagonicus	*A. forsteri*	20	2	1278	12	32	[RGB+14]+	[FBRG14]
Arabidopsis thaliana	*A. lyrata*	345	1	10322757	1023148	398365	[ABAB+16]	Public directory∘
Armadillidium vulgare	*A. nasatum*	20	4	23323	745		[RGB+14]+	same
Artemia franciscana	*A. sinica*	20	4	5548	247		[RGB+14]+	same
Athene cunicularia	*Strix occidentalis*	40	2	11268203	383702	68196	[MKB+18]⋆	[HHW+17]
Bacillus subtilis	*B. atrophaeus*	38	1	105523	29934		[RdSB+18]*	NCBI RefSeq• - Nov 2016
Caenorhabditis brenneri	*Caenorhabditis sp. 10*	20	4	1339	106		[RGB+14]+	same
Caenorhabditis elegans (Orsay)	*C. elegans* ECA396 ECA723 ECA744	573	3	165	5	22	[RZL+18]	same
Chlamydia trachomatis	*C. muridarum*	59	1	9924	1694		[RdSB+18]*	NCBI RefSeq• - Nov 2016
Ciona intestinalis A	*C. intestinalis B*	20	20	1491	63	377	[RGB+14]+	same
Ciona intestinalis B	*C. intestinalis A*	20	20	2186	67	139	[RGB+14]+	same
Clostridium difficile	*Anaerococcus prevotii*	11	1	192	49		[RdSB+18]*	NCBI RefSeq• - Nov 2016
Corvus cornix	*C. monedula*	38	20	7551159	49479	269096	[PVB+14]⋆	[VBP+16; PVB+14]
Coturnix japonica	*Gallus varius*	20	14	5061864	87069	220450	[WZH+18]⋆	[UKMF+16]
Culex pipiens	*C. torrentium*	20	4	5442	106		[RGB+14]+	same
Drosophila melanogaster	*D. simulans*	196	1	4662706	151138		[LCC+15]	[SK16]
Egretta garzetta	*Pelecanus crispus*	10	2	9318499	361539	10242	[LLC+14]⋆	[ZLL+14]
Emys orbicularis	*Trachemys scripta*	20	4	515	14		[RGB+14]+	same
Escherichia coli	*E. fergusonii*	62	1	84222	6903		NCBI RefSeq• used in [LBL+16]	same
Ficedula albicollis	*F. hypoleuca*	24	2	14697230	269430	229260	[ESB+12; KHM+16; SMQE16; BNK+15]⋆	[BNK+15]
Gorilla gorilla	ancestral allele call from [PMSK+13]	54	Inferred	9878547	42	569321	[PMSK+13]	same
Halictus scabiosae	*H simplex*	22	2	712	10		[RGB+14]+	[Gal16]
Helicobacter pylori	*H. felis*	70	1	27498	8235		[RdSB+18]*	NCBI RefSeq• - Nov 2016
Homo sapiens (Yoruba)	ancestral allele call from [Con15]	216	Inferred	19441528	105146		[Con15]	same
Klebsiella pneumoniae	*K. varicola*	156	1	203601	375		[RdSB+18]*	NCBI RefSeq• - Nov 2016
Lepus granatensis	*L americanus*	20	2	769	31	1	[RGB+14]+	[GMFG+13]
Melitaea cinxia	*M didyma*	18		1695	101		[RGB+14]+	same
Messor barbarus	*M structor*	20	8	9651	50		[RGB+14]+	[Gal16]
Mycobacterium tubercolosis	MYCN001 - MYCN005	33	2	7142	13	78	NCBI RefSeq• - Nov 2013	same
Nipponia nippon	*Pelecanus crispus*	16	2	1140694	44153	2034	[LLC+14]⋆	[ZLL+14]
Ostrea edulis	*O. chilensis*	20	4	939	28		[RGB+14]+	same
Pan paniscus	ancestral allele call from [PMSK+13]	26	Inferred	6293657	63	284527	[PMSK+13]	same
Pan troglodytes ellioti	ancestral allele call from [PMSK+13]	20	Inferred	10009190	44	459884	[PMSK+13]	same
Parus caeruleus	*P. major*	20	2	866	51	19	[RGB+14]+	[FBRG14]
Parus major	*Cyanistes caeruleus*	54	2	14174305	143760	126876	[QTH+15; LGS+16]⋆	[MKTK16]
Passer domesticus	*P. montanus*	16	2	18501992	90623	633399	[RTE+18; ETT+17; RET+18]⋆	[ETT+17]
Phylloscopus trochilus	*P. tristis*	24	40	33401127	8605	6092936	[LLL+17]⋆	[TKS+17]
Physa acuta	*P. gyrina*	18	4	4286	176		[RGB+14]+	same
Pseudomonas aeruginosa	*P. knackmussii*	86	1	90258	17208		[RdSB+18]*	NCBI RefSeq• - Nov 2016
Sepia officinalis	*Sepiella japonica*	18	2	1740	52		[RGB+14]+	[Gal16]
Staphylococcus aureus	*S. epidermis*	152	1	30052	8694		[RdSB+18]*	NCBI RefSeq• - Nov 2016
Streptococcus pneumoniae	*S. mitis*	32	1	49917	2468		[RdSB+18]*	NCBI RefSeq• - Nov 2016
Taeniopygia guttata	*Poephila acuticauda*	38	40	53263038	118506	4346767	[SLS+15]⋆	same
Zea mays	*Tripsacum dactyloides*	66	1	520310	214398	1	[BMHR+17]	same

*Core genomes were computed and aligned as is [RdSB+18], we added to the set of a species genome one further genome from the closest species (to be able to orient the changes).

number of positions where the outgroup was diallelic (such positions are excluded, since we cannot set the ancestral allele to the outgroup allele then)

⋆ `https://github.com/harvardinformatics/shortRead_mapping_variantCalling` +SFS were extracted from transcriptomes as described in [RGB+14].

• NCBI RefSeq `ftp://ftp.ncbi.nih.gov/genomes/` [TCF+14]

∘ `pipeline.lbl.gov/data/araTha04_Araly1/`

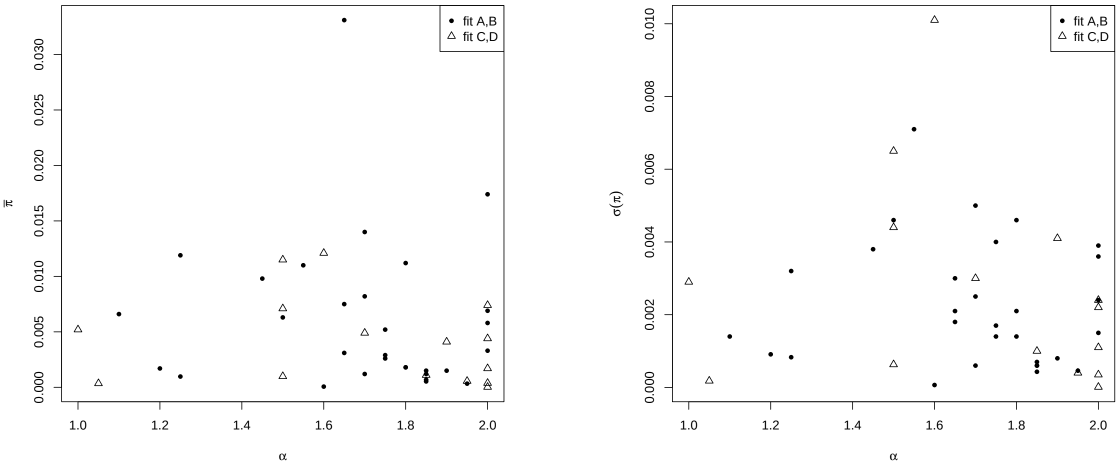

Figure A.23: Comparison of estimated α parameter $\hat{\alpha}$ (x-axis) and mean $\bar{\pi}$ (standard deviation $\sigma(\pi)$) of windowed nucleotide diversity π (y-axis). See Sect. A.12) for details.

Table H: Population structure inference, nucleotide diversity π (per site in 15 kb windows), $\hat{T}_{div}$ between in- and outgroup (18) and $\mathbb{P}_{ILS} := P(T_{MRCA} > \hat{T}_{div})$ (rounded to 10^{-4}, ANC: reconstructed ancestral sequence as outgroup, T_{div} not estimated). Model fitted, grade of fit, and biological order repeated from Table 2. Number of clusters: k inferred by BIC criterion for subsequent k-means clustering. PCA eye-test: Visual inspectation of PCA plot. Clear structure: "yes" if PCA eye-test=yes and DAPC cluster=1, "no" if PCA eye-test=no and DAPC cluster>1, otherwise "?".

Species (clade)	best model, grade	PCA eye-test	DAPC clusters	clear structure	mean π (s.d. π) $\times 10^{-3}$	$\hat{T}_{div}$	$\mathbb{P}(ILS)$
Aptenodytes patagonicus (V)	Beta, B	unclear	5	?	1.0 (0.8)	4.40	0.033
Athene cunicularia (V)	Beta, B	yes	1	?	1.8 (1.4)	24.81	0
Corvus cornix (V)	Beta, A	no	1	no	1.5 (0.7)	6.39	0.005
Coturnix japonica (V)	Beta, A	no	1	no	9.8 (3.8)	3.46	0.085
Egretta garzetta (V)	Beta, B	no	4	?	2.6 (1.7)	18.89	0
Emys orbicularis (V)	KM, C	unclear	2	?	1.1 (1.0)	10.90	10^{-4}
Ficedula albicollis (V)	Ψ, A	no	1	no	3.3 (1.5)	6.28	0.005
Gorilla gorilla (V)	Beta, B	no	1	no	1.5 (0.8)	ANC	ANC
Homo sapiens (Yoruba) (V)	Beta, A	no	1	no	1.2 (0.6)	ANC	ANC
Lepus granatensis (V)	MMC, C	yes	2	yes	1.0 (0.6)	9.83	10^{-4}
Nipponia nippon (V)	KM, D	unclear	7	?	0.4 (0.3)	146.25	0
Pan paniscus (V)	Beta, B	no	1	no	0.7 (0.4)	ANC	ANC
Pan troglodytes ellioti (V)	Beta, A	unclear	1	?	1.2 (0.6)	ANC	ANC
Parus caeruleus (V)	MMC, B	unclear	1	?	1.7 (0.9)	11.34	0
Parus maior (V)	Beta, A	no	1	no	2.9 (1.4)	5.3	0.014
Passer domesticus (V)	KM, A	no	1	no	5.8 (2.4)	1.35	0.63
Phylloscopus trochilus (V)	KM, A	no	1	no	6.9 (3.6)	0.745	0.95
Taeniopygia guttata (V)	Beta, A	no	1	no	5.2 (4.0)	0.812	0.93
Armadillidium vulgare (I)	Beta, C	no	1	no	4.9 (3.0)	5.17	0.016
Artemia franciscana (I)	Beta, B	no	1	no	3.1 (1.8)	14.40	0
Caenorhabditis brenneri (I)	Beta, C	no	1	no	7.1 (4.4)	12.27	0
Caenorhabditis elegans (I)	KM, D	no	10	?	0.02 (0.01)	13671	0
Ciona intestinalis A (I)	Beta, C	unclear	3	?	4.1 (4.1)	10.83	10^{-4}
Ciona intestinalis B (I)	Beta, C	unclear	9	?	12.1 (10.1)	2.79	0.17
Culex pipiens (I)	Beta, B	unclear	2	?	11 (7.1)	2.04	0.343
Drosophila melanogaster (I)	Beta, A	yes	2	yes	7.5 (3)	140	0
Halictus scabiosae (I)	MMC, B	unclear	2	?	0.5 (0.6)	36.30	0
Melitaea cinxia (I)	Beta, B	unclear	3	?	13.5 (5.3)	6.57	0.004
Messor barbarus (I)	KM, C	unclear	2	?	1.7 (1.1)	8.86	$4 \cdot 10^{-4}$
Ostrea edulis (I)	MMC, B	yes	2	yes	1.8 (2.1)	14.62	0
Physa acuta (I)	Beta, B	no	1	no	6.3 (4.6)	4.86	0.021
Sepia officinalis (I)	KM, C	no	2	?	0.6 (0.4)	31.47	0
Zea mays (P)	Ψ, A	unclear	3	?	0.3 (0.5)	65.28	0
Arabidopsis thaliana (P)	Beta, A	yes	1	?	0.06 (0.07)	1.256	0.74
Acinetobacter baumannii (B)	Beta, B	yes	14	yes	11.2 (4.6)	6.59	0.004
Bacillus subtilis (B)	Ψ, B	unclear	5	?	11.9 (3.2)	0.812	0.927
Chlamydia trachomatis (B)	KM, D	yes	8	yes	4.4 (2.2)	3.67	0.074
Clostridium difficile (B)	KM, D	unclear	5	?	5.2 (2.9)	102.4	0
Escherichia coli (B)	KM, B	yes	17	yes	17.4 (3.9)	-0.41	1
Helicobacter pilori (B)	Ψ, B	yes	2	yes	33.1 (2.1)	4.55	0.03
Klebsiella pneumoniae (B)	KM, D	unclear	9	?	7.4 (2.4)	5.71	0.01
Mycobacterium tubercolosis (B)	Beta, C	yes	12	yes	0.4 (0.2)	(-0.44)	(1)
Pseudomonas aeruginosa (B)	Ψ, B	yes	9	yes	6.6 (1.4)	27.9	0
Staphylococcus aureus (B)	Ψ, B	yes	15	yes	8.2 (2.5)	34.71	0
Streptococcus pneumoniae (B)	Beta, C	yes	2	yes	11.5 (6.5)	-0.5	1

x	$\rho(\hat{\alpha}, x)$ all data sets	$\rho(\alpha, x)$ data sets with fits A,B
$\bar{\pi}$	-.11	-.09
$\sigma(\pi)$	-.14	-.07

Table I: Correlation coefficient ρ of estimated α parameter $\hat{\alpha}$ and mean $\bar{\pi}$ (standard deviation $\sigma(\pi)$) of windowed nucleotide diversity π (Sect. A.12)

References

[ABAB$^+$16] Carlos Alonso-Blanco, Jorge Andrade, Claude Becker, Felix Bemm, Joy Bergelson, Karsten M. Borgwardt, Jun Cao, Eunyoung Chae, Todd M. Dezwaan, Wei Ding, Joseph R. Ecker, Moises Exposito-Alonso, Ashley Farlow, Joffrey Fitz, Xiangchao Gan, Dominik G. Grimm, Angela M. Hancock, Stefan R. Henz, Svante Holm, Matthew Horton, Mike Jarsulic, Randall A. Kerstetter, Arthur Korte, Pamela Korte, Christa Lanz, Cheng-Ruei Lee, Dazhe Meng, Todd P. Michael, Richard Mott, Ni Wayan Muliyati, Thomas Nägele, Matthias Nagler, Viktoria Nizhynska, Magnus Nordborg, Polina Yu. Novikova, F. Xavier Picó, Alexander Platzer, Fernando A. Rabanal, Alex Rodriguez, Beth A. Rowan, Patrice A. Salomé, Karl J. Schmid, Robert J. Schmitz, Ümit Seren, Felice Gianluca Sperone, Mitchell Sudkamp, Hannes Svardal, Matt M. Tanzer, Donald Todd, Samuel L. Volchenboum, Congmao Wang, George Wang, Xi Wang, Wolfram Weckwerth, Detlef Weigel, and Xuefeng Zhou. 1,135 genomes reveal the global pattern of polymorphism in arabidopsis thaliana. *Cell*, 166(2):481–491, 2016.

[BD03] Emmanuelle Baudry and Frantz Depaulis. Effect of misoriented sites on neutrality tests with outgroup. *Genetics*, 165(3):1619–1622, 2003.

[BMHR$^+$17] Jean-Tristan Brandenburg, Tristan Mary-Huard, Guillem Rigaill, Sarah J. Hearne, Hélène Corti, Johann Joets, Clémentine Vitte, Alain Charcosset, Stéphane D. Nicolas, and Maud I. Tenaillon. Independent introductions and admixtures have contributed to adaptation of european maize and its american counterparts. *PLOS Genetics*, 13(3):e1006666, Mar 2017.

[BNK$^+$15] Reto Burri, Alexander Nater, Takeshi Kawakami, Carina F Mugal, Pall I Olason, Linnea Smeds, Alexander Suh, Ludovic Dutoit, Stanislav Bureš, Laszlo Z Garamszegi, et al. Linked selection and recombination rate variation drive the evolution of the genomic landscape of differentiation across the speciation continuum of ficedula flycatchers. *Genome research*, 25(11):1656–1665, 2015.

[BW21] Ellen Baake and Anton Wakolbinger. *Probabilistic Structures in Evolution*. EMS Press, 2021.

[Can74] C. Cannings. The latent roots of certain markov chains arising in genetics: A new approach, i. haploid models. *Advances in Applied Probability*, 6(2):260–290, 1974.

[Con15] The 1000 Genomes Project Consortium. A global reference for human genetic variation. *Nature*, 526(7571):68–74, 2015.

[Cra16] Harald Cramér. *Mathematical Methods of Statistics (PMS-9), Volume 9*. Princeton university press, 2016.

[DAA$^+$11] Petr Danecek, Adam Auton, Goncalo Abecasis, Cornelis A Albers, Eric Banks, Mark A DePristo, Robert E Handsaker, Gerton Lunter, Gabor T Marth, Stephen T Sherry, et al. The variant call format and vcftools. *Bioinformatics*, 27(15):2156–2158, 2011.

[DFSJY13] Jean-Stéphane Dhersin, Fabian Freund, Arno Siri-Jégousse, and Linglong Yuan. On the length of an external branch in the beta-coalescent. *Stochastic Processes and their Applications*, 123(5):1691–1715, 2013.

[EBBF15] Bjarki Eldon, Matthias Birkner, Jochen Blath, and Fabian Freund. Can the site-frequency spectrum distinguish exponential population growth from multiple-merger coalescents? *Genetics*, 199(3):841–856, 2015.

[EF18] Bjarki Eldon and Fabian Freund. Genealogical properties of subsamples in highly fecund populations. *Journal of Statistical Physics*, 172(1):175–207, 2018.

[Eld20] Bjarki Eldon. Evolutionary genomics of high fecundity. *Annual Review of Genetics*, 54, 2020.

[ESB+12] Hans Ellegren, Linnea Smeds, Reto Burri, Pall I Olason, Niclas Backström, Takeshi Kawakami, Axel Künstner, Hannu Mäkinen, Krystyna Nadachowska-Brzyska, Anna Qvarnström, et al. The genomic landscape of species divergence in ficedula flycatchers. *Nature*, 491(7426):756–760, 2012.

[ETT+17] Tore O Elgvin, Cassandra N Trier, Ole K Tørresen, Ingerid J Hagen, Sigbjørn Lien, Alexander J Nederbragt, Mark Ravinet, Henrik Jensen, and Glenn-Peter Sætre. The genomic mosaicism of hybrid speciation. *Science advances*, 3(6):e1602996, 2017.

[FBRG14] Emeric Figuet, Marion Ballenghien, Jonathan Romiguier, and Nicolas Galtier. Biased Gene Conversion and GC-Content Evolution in the Coding Sequences of Reptiles and Vertebrates. *Genome Biology and Evolution*, 7(1):240–250, 12 2014.

[FKR+18] Luca Ferretti, Alexander Klassmann, Emanuele Raineri, Sebastian E Ramos-Onsins, Thomas Wiehe, and Guillaume Achaz. The neutral frequency spectrum of linked sites. *Theoretical population biology*, 123:70–79, 2018.

[FLW+17] Luca Ferretti, Alice Ledda, Thomas Wiehe, Guillaume Achaz, and Sebastian E Ramos-Onsins. Decomposing the site frequency spectrum: the impact of tree topology on neutrality tests. *Genetics*, 207(1):229–240, 2017.

[Fu95] Yun-Xin Fu. Statistical properties of segregating sites. *Theoretical population biology*, 48(2):172–197, 1995.

[Gal16] Nicolas Galtier. Adaptive protein evolution in animals and the effective population size hypothesis. *PLOS Genetics*, 12(1):e1005774, jan 2016.

[GIM14] Alexander Gnedin, Alexander Iksanov, and Alexander Marynych. λ-coalescents: a survey. *Journal of Applied Probability*, 51(A):23–40, 2014.

[GJQP+19] Hugh G Gauch Jr, Sheng Qian, Hans-Peter Piepho, Linda Zhou, and Rui Chen. Consequences of PCA graphs, SNP codings, and PCA variants for elucidating population structure. *PloS one*, 14(6):e0218306, 2019.

[GMFG+13] Philippe Gayral, José Melo-Ferreira, Sylvain Glémin, Nicolas Bierne, Miguel Carneiro, Benoit Nabholz, Joao M. Lourenco, Paulo C. Alves, Marion Ballenghien, Nicolas Faivre, Khalid Belkhir, Vincent Cahais, Etienne Loire, Aurélien Bernard, and Nicolas Galtier. Reference-free population genomics from next-generation transcriptome data and the vertebrate–invertebrate gap. *PLOS Genetics*, 9(4):1–15, 04 2013.

[HHW+17] Zachary R Hanna, James B Henderson, Jeffrey D Wall, Christopher A Emerling, Jérôme Fuchs, Charles Runckel, David P Mindell, Rauri CK Bowie, Joseph L DeRisi, and John P Dumbacher. Northern spotted owl (strix occidentalis caurina) genome: divergence with the barred owl (strix varia) and characterization of light-associated genes. *Genome biology and evolution*, 9(10):2522–2545, 2017.

[HM13] Thierry Huillet and Martin Möhle. On the extended moran model and its relation to coalescents with multiple collisions. *Theoretical population biology*, 87:5–14, 2013.

[HS76] David Heath and William Sudderth. De finetti's theorem on exchangeable variables. *The American Statistician*, 30(4):188–189, 1976.

[IM02] Alex Iksanov and Martin Möhle. On the number of jumps of random walks with a barrier. *Advances in Applied Probability*, 40(01):206–228, 2002.

[JA11] Thibaut Jombart and Ismaïl Ahmed. adegenet 1.3-1: new tools for the analysis of genome-wide snp data. *Bioinformatics*, 27(21):3070–3071, 2011.

[JPS+19] Jeffrey D. Jensen, Bret A. Payseur, Wolfgang Stephan, Charles F. Aquadro, Michael Lynch, Deborah Charlesworth, and Brian Charlesworth. The importance of the neutral theory in 1968 and 50 years on: A response to kern and hahn 2018. *Evolution*, 73(1):111–114, 2019.

[KHM+16] Marty Kardos, Arild Husby, S Eryn McFarlane, Anna Qvarnström, and Hans Ellegren. Whole-genome resequencing of extreme phenotypes in collared flycatchers highlights the difficulty of detecting quantitative trait loci in natural populations. *Molecular Ecology Resources*, 16(3):727–741, 2016.

[Kim80] Motoo Kimura. A simple method for estimating evolutionary rates of base substitutions through comparative studies of nucleotide sequences. *Journal of molecular evolution*, 16(2):111–120, 1980.

[Kin82] J.F.C. Kingman. The coalescent. *Stochastic Processes and their Applications*, 13(3):235–248, Sep 1982.

[Kos18] Jere Koskela. Multi-locus data distinguishes between population growth and multiple merger coalescents. *Statistical applications in genetics and molecular biology*, 17(3), 2018.

[Lap17] Marguerite Lapierre. Extensions du modèle standard neutre pertinentes pour l'analyse de la diversité génétique. Université Pierre et Marie Curie-Paris VI, 2017.

[LBL+16] Marguerite Lapierre, Camille Blin, Amaury Lambert, Guillaume Achaz, and Eduardo PC Rocha. The impact of selection, gene conversion, and biased sampling on the assessment of microbial demography. *Molecular biology and evolution*, 33(7):1711–1725, 2016.

[LCC+15] Justin B Lack, Charis M Cardeno, Marc W Crepeau, William Taylor, Russell B Corbett-Detig, Kristian A Stevens, Charles H Langley, and John E Pool. The Drosophila Genome Nexus: A Population Genomic Resource of 623 Drosophila melanogaster Genomes, Including 197 from a Single Ancestral Range Population. *Genetics*, 199(4):1229–1241, 01 2015.

[LGS+16] Veronika N Laine, Toni I Gossmann, Kyle M Schachtschneider, Colin J Garroway, Ole Madsen, Koen JF Verhoeven, Victor De Jager, Hendrik-Jan Megens, Wesley C Warren, Patrick Minx, et al. Evolutionary signals of selection on cognition from the great tit genome and methylome. *Nature communications*, 7(1):1–9, 2016.

[LLC+14] Shengbin Li, BO Li, Cheng Cheng, Zijun Xiong, Qingbo Liu, Jianghua Lai, Hannah V Carey, Qiong Zhang, Haibo Zheng, Shuguang Wei, et al. Genomic signatures of near-extinction and rebirth of the crested ibis and other endangered bird species. *Genome biology*, 15(12):1–17, 2014.

[LLL+17] Max Lundberg, Miriam Liedvogel, Keith Larson, Hanna Sigeman, Mats Grahn, Anthony Wright, Susanne Åkesson, and Staffan Bensch. Genetic differences between willow warbler migratory phenotypes are few and cluster in large haplotype blocks. *Evolution Letters*, 1(3):155–168, 2017.

[MGF20] Fabrizio Menardo, Sébastien Gagneux, and Fabian Freund. Multiple Merger Genealogies in Outbreaks of Mycobacterium tuberculosis. *Molecular Biology and Evolution*, 38(1):290–306, 07 2020.

[MKB+18] Jakob C Mueller, Heiner Kuhl, Stefan Boerno, Jose L Tella, Martina Carrete, and Bart Kempenaers. Evolution of genomic variation in the burrowing owl in response to recent colonization of urban areas. *Proceedings of the Royal Society B: Biological Sciences*, 285(1878):20180206, 2018.

[MKTK16] Jakob C Mueller, Heiner Kuhl, Bernd Timmermann, and Bart Kempenaers. Characterization of the genome and transcriptome of the blue tit c yanistes caeruleus: polymorphisms, sex-biased expression and selection signals. *Molecular ecology resources*, 16(2):549–561, 2016.

[MS01] Martin Möhle and Serik Sagitov. A classification of coalescent processes for haploid exchangeable population models. *The Annals of Probability*, 29(4):1547–1562, 2001.

[Par10] Emmanuel Paradis. pegas: an r package for population genetics with an integrated–modular approach. *Bioinformatics*, 26(3):419–420, 2010.

[PATE18] Fanny Pouyet, Simon Aeschbacher, Alexandre Thiéry, and Laurent Excoffier. Background selection and biased gene conversion affect more than 95% of the human genome and bias demographic inferences. *Elife*, 7:e36317, 2018.

[Pit99] Jim Pitman. Coalescents with multiple collisions. *Annals of Probability*, 27(4):1870–1902, 1999.

[PMSK+13] Javier Prado-Martinez, Peter H. Sudmant, Jeffrey M. Kidd, Heng Li, Joanna L. Kelley, Belen Lorente-Galdos, Krishna R. Veeramah, August E. Woerner, Timothy D. O'Connor, Gabriel Santpere, Alexander Cagan, Christoph Theunert, Ferran Casals, Hafid Laayouni, Kasper Munch, Asger Hobolth, Anders E. Halager, Maika Malig, Jessica Hernandez-Rodriguez, Irene Hernando-Herraez, Kay Prüfer, Marc Pybus, Laurel Johnstone, Michael Lachmann, Can Alkan, Dorina Twigg, Natalia Petit, Carl Baker, Fereydoun Hormozdiari, Marcos Fernandez-Callejo, Marc Dabad, Michael L. Wilson, Laurie Stevison, Cristina Camprubí, Tiago Carvalho, Aurora Ruiz-Herrera, Laura Vives, Marta Mele, Teresa Abello, Ivanela Kondova, Ronald E. Bontrop, Anne Pusey, Felix Lankester, John A. Kiyang, Richard A. Bergl, Elizabeth Lonsdorf, Simon Myers, Mario Ventura, Pascal Gagneux, David Comas, Hans Siegismund, Julie Blanc, Lidia Agueda-Calpena, Marta Gut, Lucinda Fulton, Sarah A. Tishkoff, James C. Mullikin, Richard K. Wilson, Ivo G. Gut, Mary Katherine Gonder, Oliver A. Ryder, Beatrice H. Hahn, Arcadi Navarro, Joshua M. Akey, Jaume Bertranpetit, David Reich, Thomas Mailund, Mikkel H. Schierup, Christina Hvilsom, Aida M. Andrés, Jeffrey D. Wall, Carlos D. Bustamante, Michael F. Hammer, Evan E. Eichler, and Tomas Marques-Bonet. Great ape genetic diversity and population history. *Nature*, 499(7459):471–475, 2013.

[PVB+14] Jelmer W Poelstra, Nagarjun Vijay, Christen M Bossu, Henrik Lantz, Bettina Ryll, Inge Müller, Vittorio Baglione, Per Unneberg, Martin Wikelski, Manfred G Grabherr, et al. The

genomic landscape underlying phenotypic integrity in the face of gene flow in crows. *Science*, 344(6190):1410–1414, 2014.

[QTH+15] Yanhua Qu, Shilin Tian, Naijian Han, Hongwei Zhao, Bin Gao, Jun Fu, Yalin Cheng, Gang Song, Per GP Ericson, Yong E Zhang, et al. Genetic responses to seasonal variation in altitudinal stress: whole-genome resequencing of great tit in eastern himalayas. *Scientific Reports*, 5(1):1–10, 2015.

[RdSB+18] Olaya Rendueles, Jorge A. Moura de Sousa, Aude Bernheim, Marie Touchon, and Eduardo P. C. Rocha. Genetic exchanges are more frequent in bacteria encoding capsules. *PLOS Genetics*, 14(12):1–25, 12 2018.

[RET+18] Mark Ravinet, Tore Oldeide Elgvin, Cassandra Trier, Mansour Aliabadian, Andrey Gavrilov, and Glenn-Peter Sætre. Signatures of human-commensalism in the house sparrow genome. *Proceedings of the Royal Society B*, 285(1884):20181246, 2018.

[RGB+14] J. Romiguier, P. Gayral, M. Ballenghien, A. Bernard, V. Cahais, A. Chenuil, Y. Chiari, R. Dernat, L. Duret, N. Faivre, and et al. Comparative population genomics in animals uncovers the determinants of genetic diversity. *Nature*, 515(7526):261–263, Aug 2014.

[RTE+18] Anna Runemark, Cassandra N Trier, Fabrice Eroukhmanoff, Jo S Hermansen, Michael Matschiner, Mark Ravinet, Tore O Elgvin, and Glenn-Peter Sætre. Variation and constraints in hybrid genome formation. *Nature Ecology & Evolution*, 2(3):549–556, 2018.

[RZL+18] Aurélien Richaud, Gaotian Zhang, Daehan Lee, Junho Lee, and Marie-Anne Félix. The local coexistence pattern of selfing genotypes in caenorhabditis elegans natural metapopulations. *Genetics*, 208(2):807–821, 2018.

[Sag99] Serik Sagitov. The general coalescent with asynchronous mergers of ancestral lines. *Journal of Applied Probability*, 36(4):1116–1125, 1999.

[Sch03] Jason Schweinsberg. Coalescent processes obtained from supercritical Galton–Watson processes. *Stochastic Proc. Appl.*, 106(1):107–139, 2003.

[Sch17] Jason Schweinsberg. Rigorous results for a population model with selection ii: genealogy of the population. *Electronic Journal of Probability*, 22, 2017.

[SD05] Jason Schweinsberg and Rick Durrett. Random partitions approximating the coalescence of lineages during a selective sweep. *The Annals of Applied Probability*, 15(3):1591 – 1651, 2005.

[SK16] Craig E. Stanley and Rob J. Kulathinal. Genomic signatures of domestication on neurogenetic genes in drosophila melanogaster. *BMC Evolutionary Biology*, 16(1):6, 2016.

[SKS16] Jeffrey P. Spence, John A. Kamm, and Yun S. Song. The site frequency spectrum for general coalescents. *Genetics*, 202(4):1549–1561, 2016.

[SLS+15] Sonal Singhal, Ellen M Leffler, Keerthi Sannareddy, Isaac Turner, Oliver Venn, Daniel M Hooper, Alva I Strand, Qiye Li, Brian Raney, Christopher N Balakrishnan, et al. Stable recombination hotspots in birds. *Science*, 350(6263):928–932, 2015.

[SMQE16] Linnéa Smeds, Carina F Mugal, Anna Qvarnström, and Hans Ellegren. High-resolution mapping of crossover and non-crossover recombination events by whole-genome re-sequencing of an avian pedigree. *PLoS genetics*, 12(5):e1006044, 2016.

[SW08] Ori Sargsyan and John Wakeley. A coalescent process with simultaneous multiple mergers for approximating the gene genealogies of many marine organisms. *Theoretical Population Biology*, 74(1):104–114, Aug 2008.

[TCF+14] Tatiana Tatusova, Stacy Ciufo, Scott Federhen, Boris Fedorov, Richard McVeigh, Kathleen O'Neill, Igor Tolstoy, and Leonid Zaslavsky. Update on RefSeq microbial genomes resources. *Nucleic Acids Research*, 43(D1):D599–D605, 12 2014.

[TKS+17] Venkat Talla, Faheema Kalsoom, Daria Shipilina, Irina Marova, and Niclas Backström. Heterogeneous patterns of genetic diversity and differentiation in european and siberian chiffchaff (phylloscopus collybita abietinus/p. tristis). *G3: Genes, Genomes, Genetics*, 7(12):3983–3998, 2017.

[TL14] Aurelien Tellier and Christophe Lemaire. Coalescence 2.0: a multiple branching of recent theoretical developments and their applications. *Molecular ecology*, 23(11):2637–2652, 2014.

[UKMF+16] Maria Ulfah, Ryouka Kawahara-Miki, Achmad Farajalllah, Muladno Muladno, Ben Dorshorst, Alison Martin, and Tomohiro Kono. Genetic features of red and green junglefowls and relationship with indonesian native chickens sumatera and kedu hitam. *BMC genomics*, 17(1):1–9, 2016.

[VBP+16] Nagarjun Vijay, Christen M Bossu, Jelmer W Poelstra, Matthias H Weissensteiner, Alexander Suh, Alexey P Kryukov, and Jochen BW Wolf. Evolution of heterogeneous genome differentiation across multiple contact zones in a crow species complex. *Nature communications*, 7(1):13195, 2016.

[WZH+18] Yan Wu, Yaolei Zhang, Zhuocheng Hou, Guangyi Fan, Jinsong Pi, Shuai Sun, Jiang Chen, Huaqiao Liu, Xiao Du, Jie Shen, et al. Population genomic data reveal genes related to important traits of quail. *GigaScience*, 7(5):giy049, 2018.

[ZLL+14] Guojie Zhang, Cai Li, Qiye Li, Bo Li, Denis M Larkin, Chul Lee, Jay F Storz, Agostinho Antunes, Matthew J Greenwold, Robert W Meredith, et al. Comparative genomics reveals insights into avian genome evolution and adaptation. *Science*, 346(6215):1311–1320, 2014.
