# Supplementary material for: Interpreting the pervasive observation of U-shaped Site Frequency Spectra
Source: PLoS Genet. 2023 Mar 23;19(3):e1010677. doi: 10.1371/journal.pgen.1010677 (PMC10072462; doi:10.1371/journal.pgen.1010677)
Supplement: S1 Fig — (PDF) [file pgen.1010677.s002.pdf]

Supplementary Figures - Site Frequency Spectra vs. Best Fitting Expectations

Supplementary material for
"Interpreting the pervasive observation of U-shaped
Site Frequency Spectra"

The following plots show, for each data set analysed,

- the observed site frequency spectrum (SFS) scaled by the total number of mutations
- the non-GC-biased proportion of the observed SFS, scaled by its total number of mutations)
- the expected SFS under the best fitting parameters
  - among all Kingman-coalescent-based model choices
  - among all Beta-coalescent-based model choices
  - among all Psi-coalescent-based model choices

  scaled by the total expected number of mutations with these best fitting parameters

Solid lines in the plot show the best fitting model(s) across the three types of coalescent models (Kingman, Beta, Psi), while dotted lines indicate inferior fit (as derived by the testing procedure using Eq. 3 in the main manuscript).

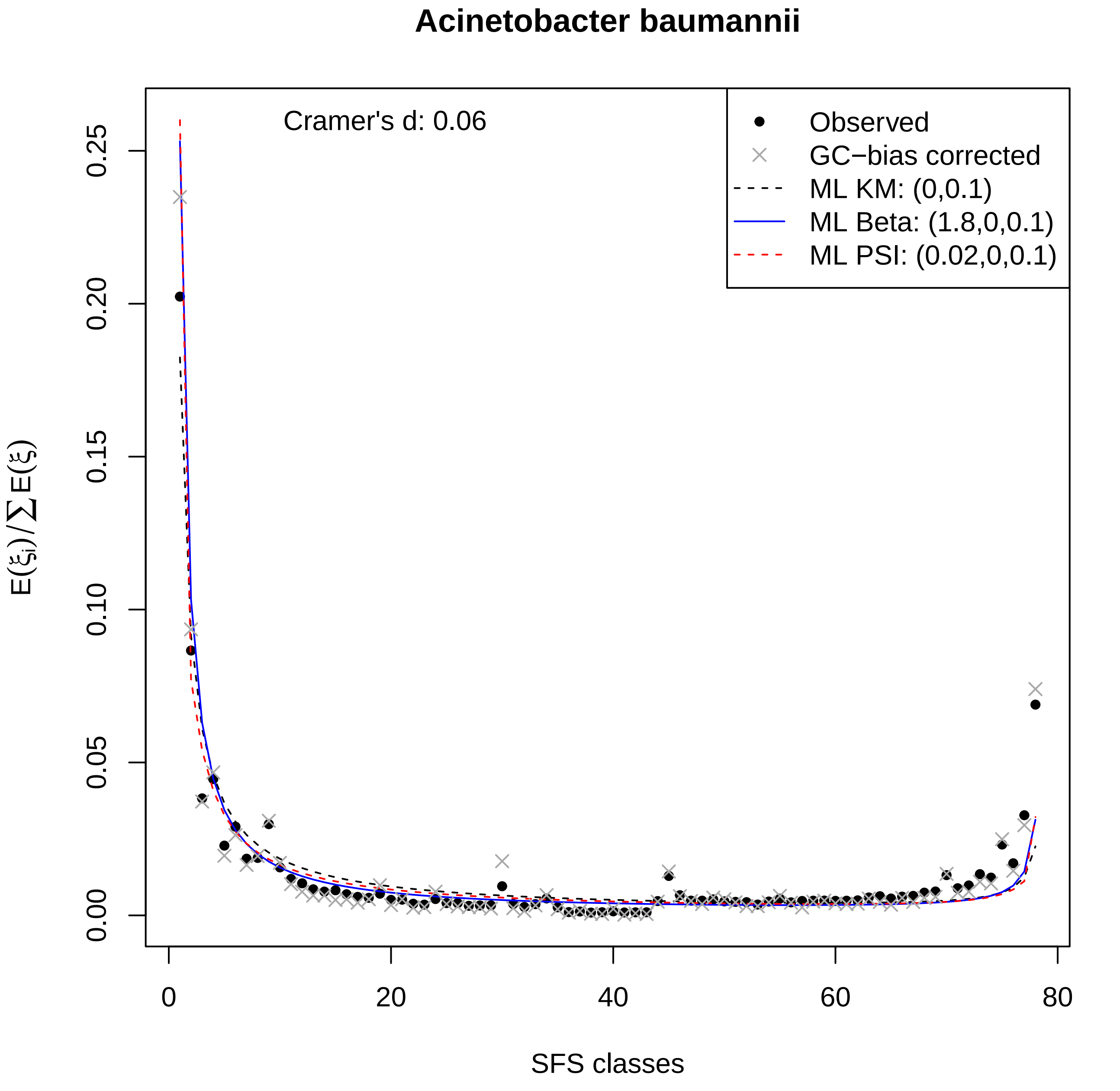

Acinetobacter baumannii
Cramer's d: 0.06
Observed
GC−bias corrected
ML KM: (0,0.1)
ML Beta: (1.8,0,0.1)
ML PSI: (0.02,0,0.1)
0.25
0.20
0.15
0.10
0.05
0.00
0
20
40
60
80
SFS classes
$E(\xi_i)/\sum E(\xi)$

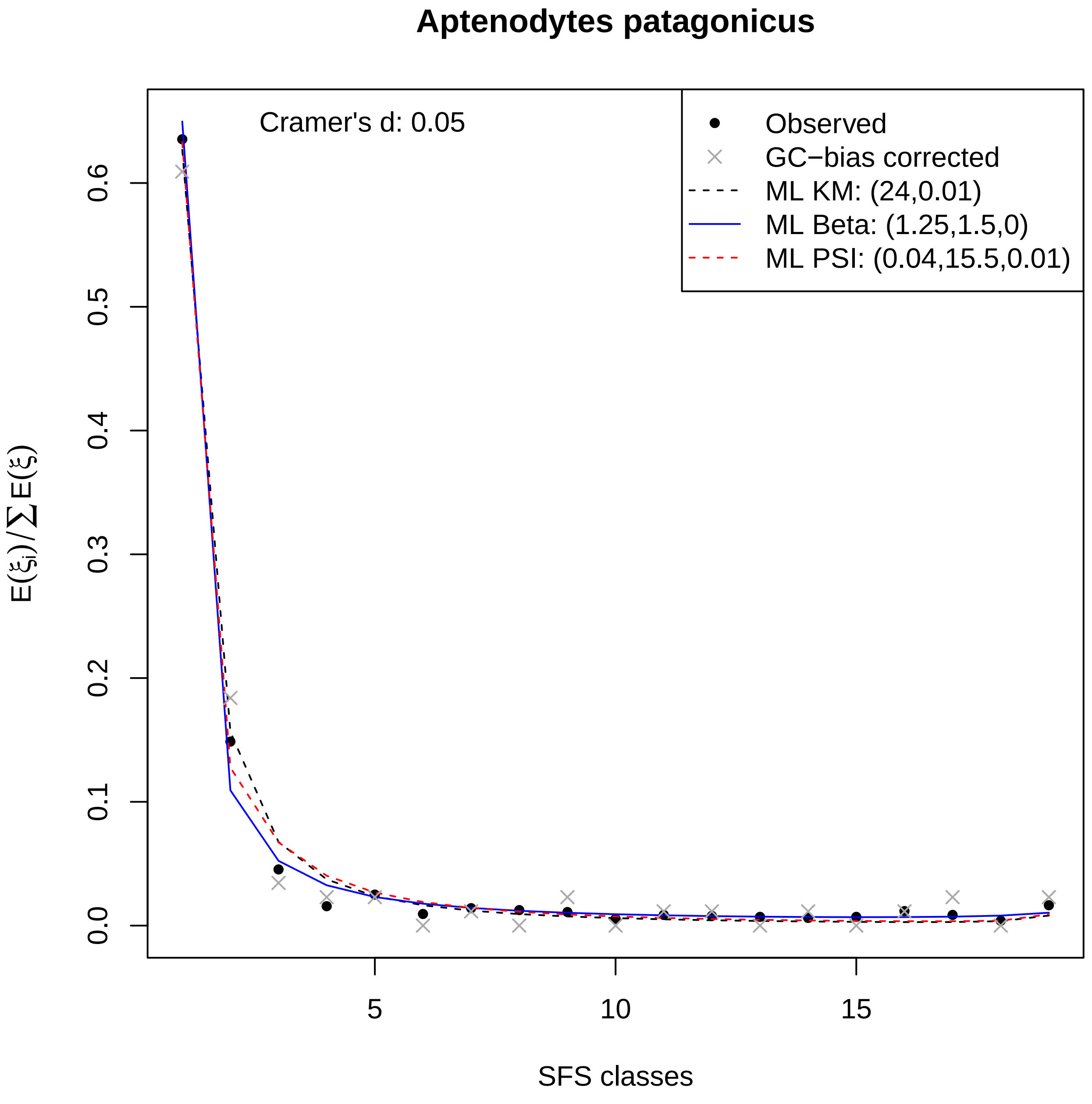

Aptenodytes patagonicus
Cramer's d: 0.05
Observed
GC−bias corrected
ML KM: (24,0.01)
ML Beta: (1.25,1.5,0)
ML PSI: (0.04,15.5,0.01)
$E(\xi_i)/\sum E(\xi)$
0.0
0.1
0.2
0.3
0.4
0.5
0.6
5
10
15
SFS classes

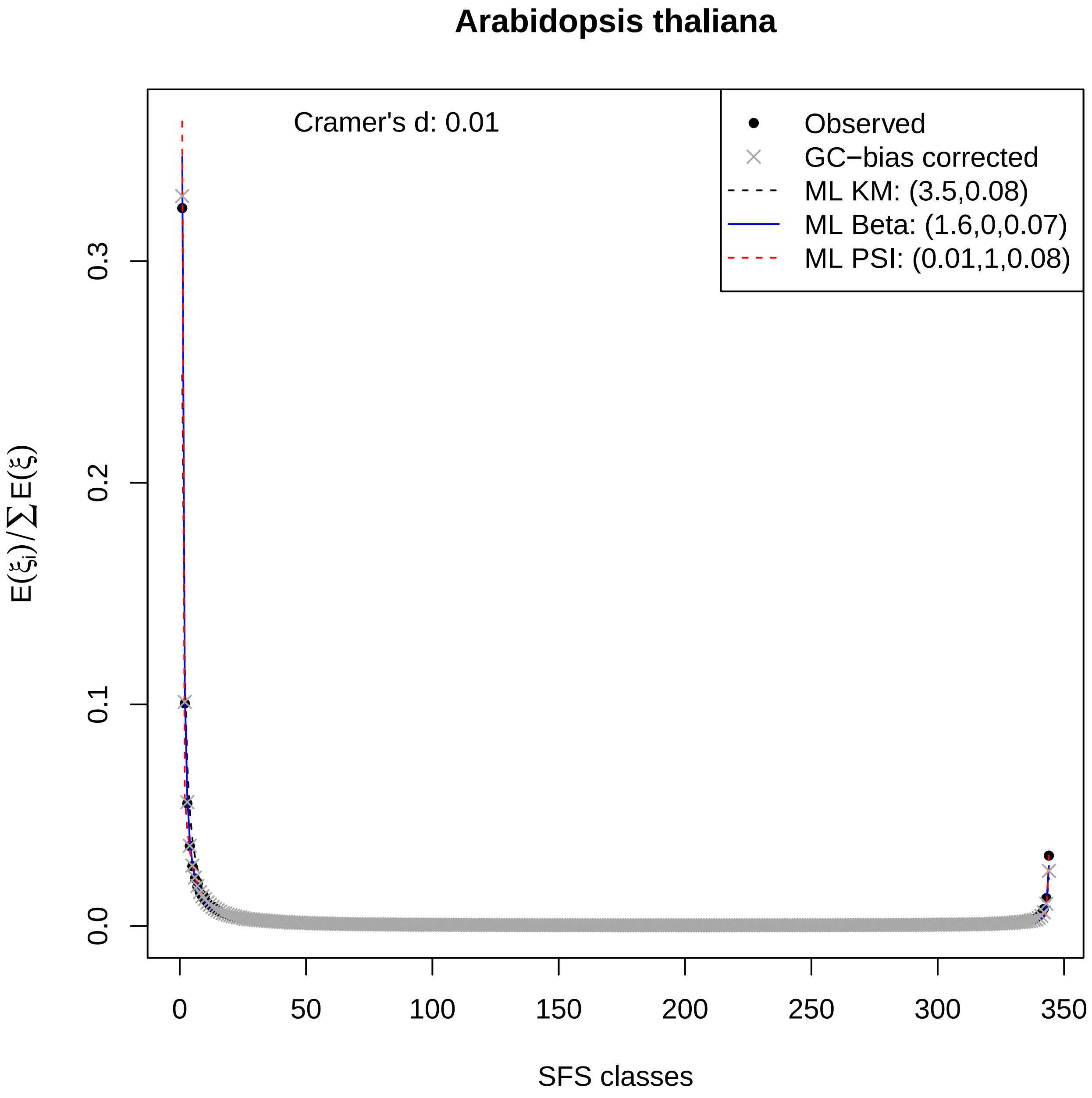

Arabidopsis thaliana
Cramer's d: 0.01
Observed
GC−bias corrected
ML KM: (3.5,0.08)
ML Beta: (1.6,0,0.07)
ML PSI: (0.01,1,0.08)
0.0
0.1
0.2
0.3
E(ξi)/∑E(ξ)
0
50
100
150
200
250
300
350
SFS classes

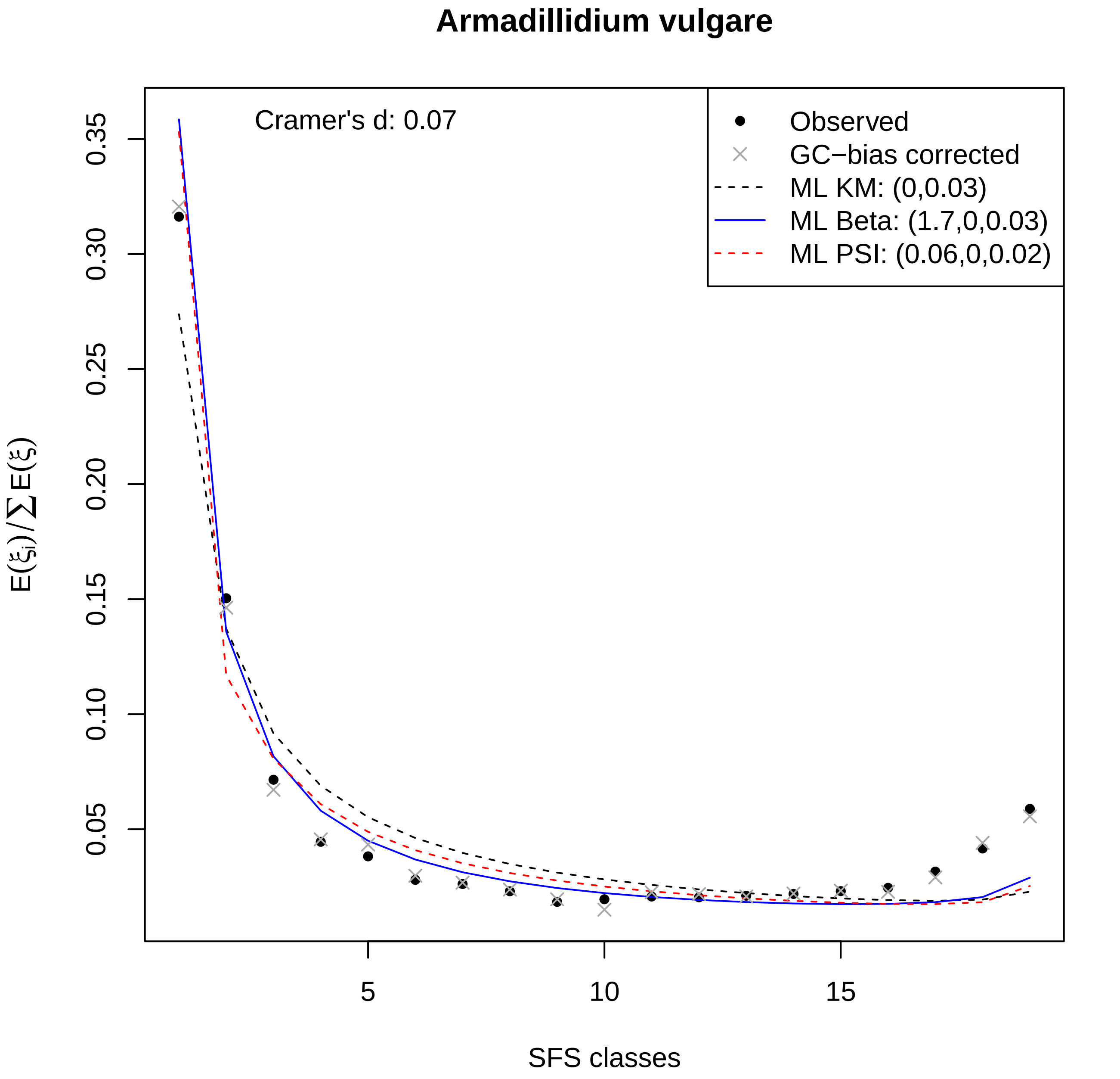

Armadillidium vulgare
Cramer's d: 0.07
Observed
GC−bias corrected
ML KM: (0,0.03)
ML Beta: (1.7,0,0.03)
ML PSI: (0.06,0,0.02)
0.05
0.10
0.15
0.20
0.25
0.30
0.35
5
10
15
SFS classes
E(ξi)/∑E(ξ)

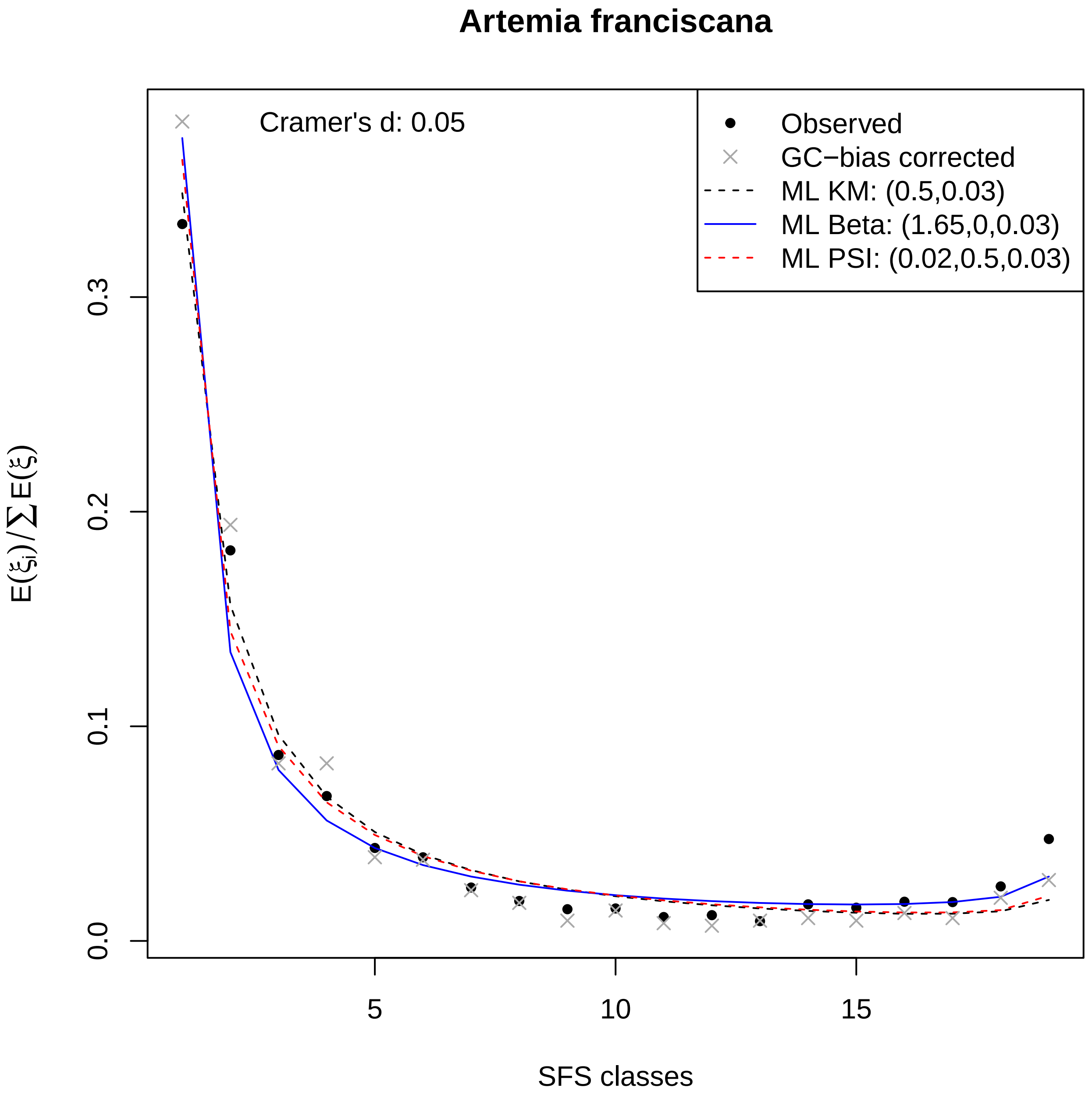

Artemia franciscana
Cramer's d: 0.05
Observed
GC−bias corrected
ML KM: (0.5,0.03)
ML Beta: (1.65,0,0.03)
ML PSI: (0.02,0.5,0.03)
$E(\xi_i)/\sum E(\xi)$
SFS classes
0.0
0.1
0.2
0.3
5
10
15

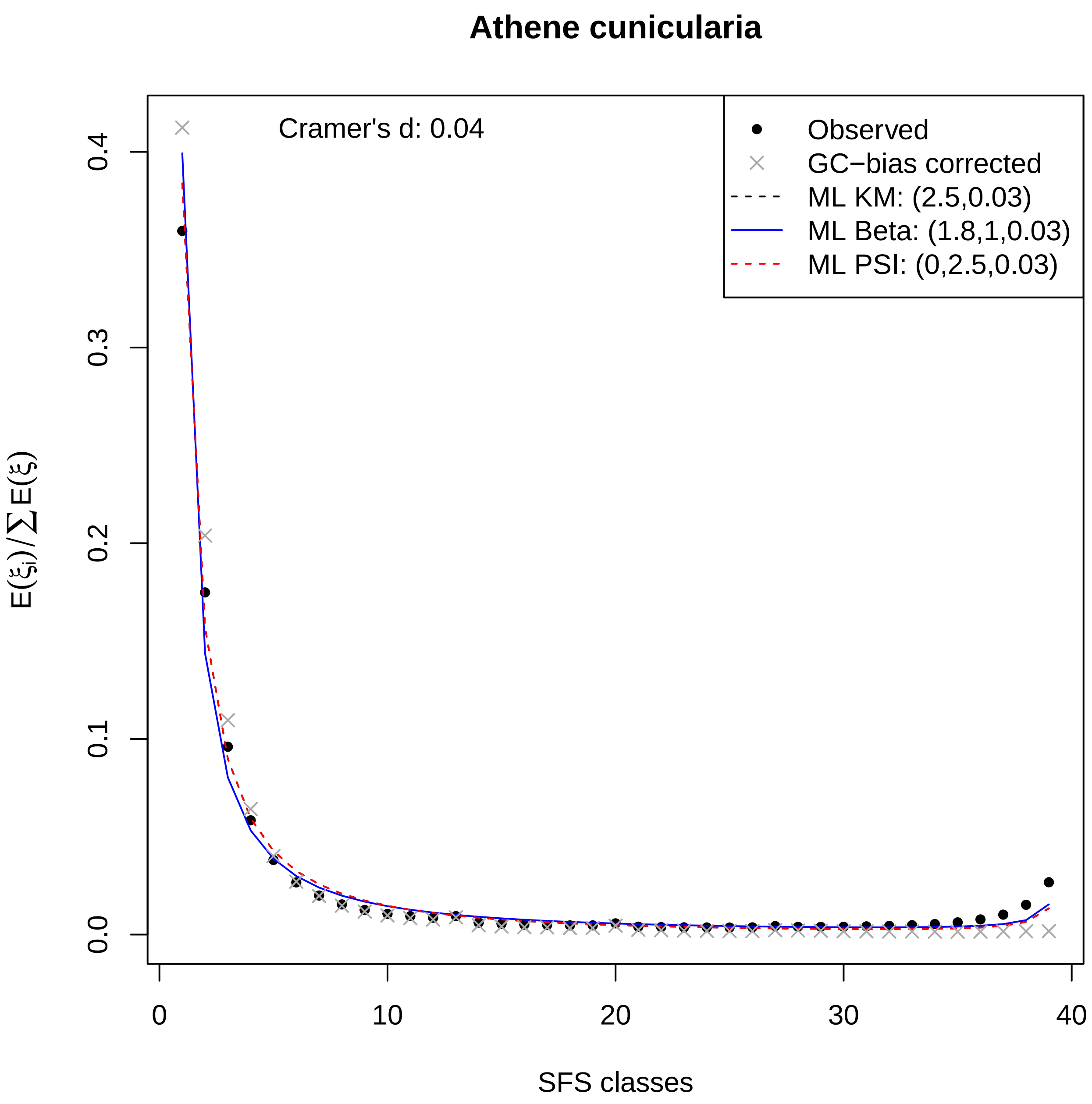
Athene cunicularia
Cramer's d: 0.04
Observed
GC−bias corrected
ML KM: (2.5,0.03)
ML Beta: (1.8,1,0.03)
ML PSI: (0,2.5,0.03)
0.0
0.1
0.2
0.3
0.4
0
10
20
30
40
SFS classes
$E(\xi_i)/\sum E(\xi)$

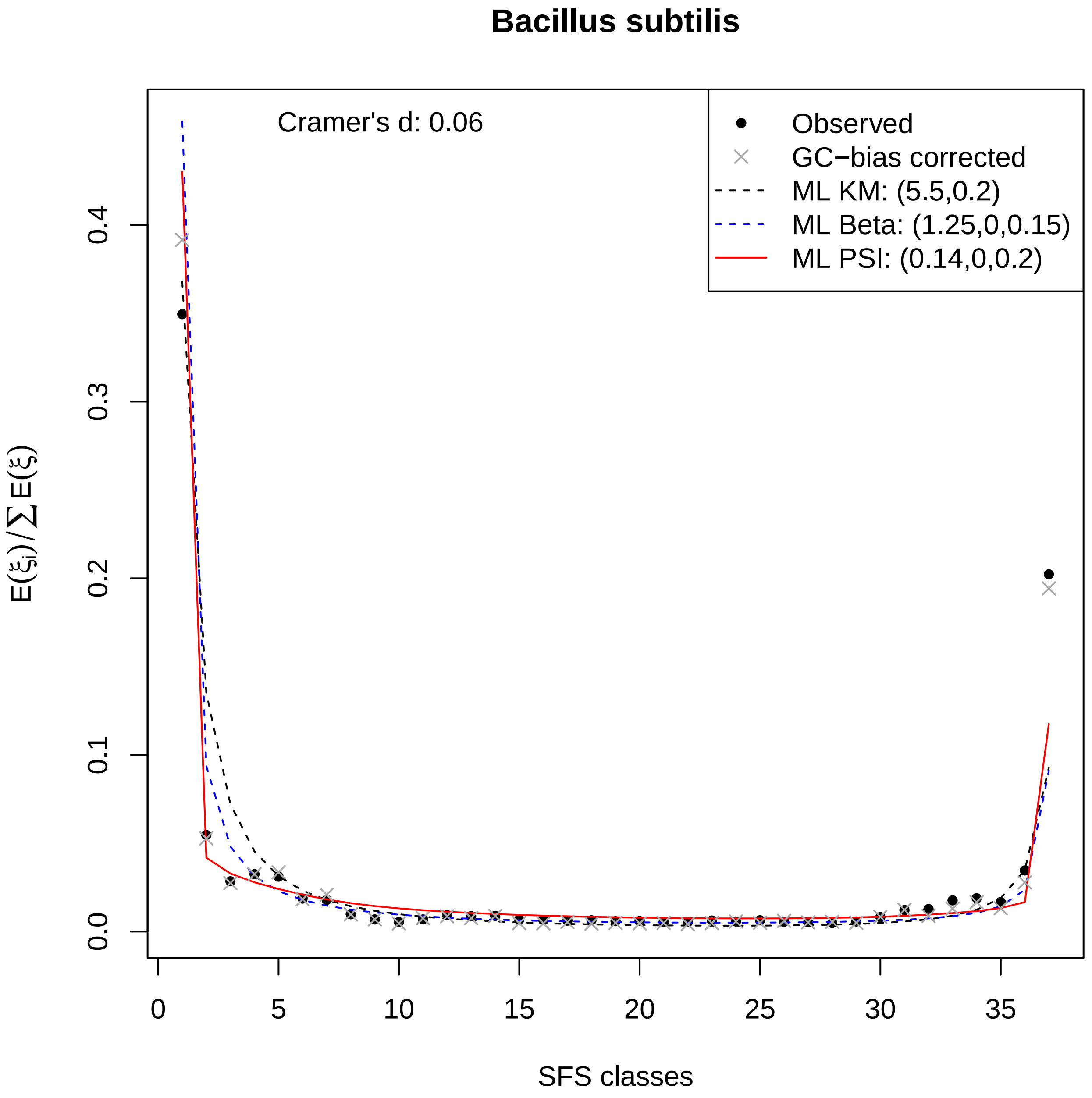

Bacillus subtilis
Cramer's d: 0.06
Observed
GC−bias corrected
ML KM: (5.5,0.2)
ML Beta: (1.25,0,0.15)
ML PSI: (0.14,0,0.2)
$E(\xi_i)/\sum E(\xi)$
SFS classes
0.0
0.1
0.2
0.3
0.4
0
5
10
15
20
25
30
35

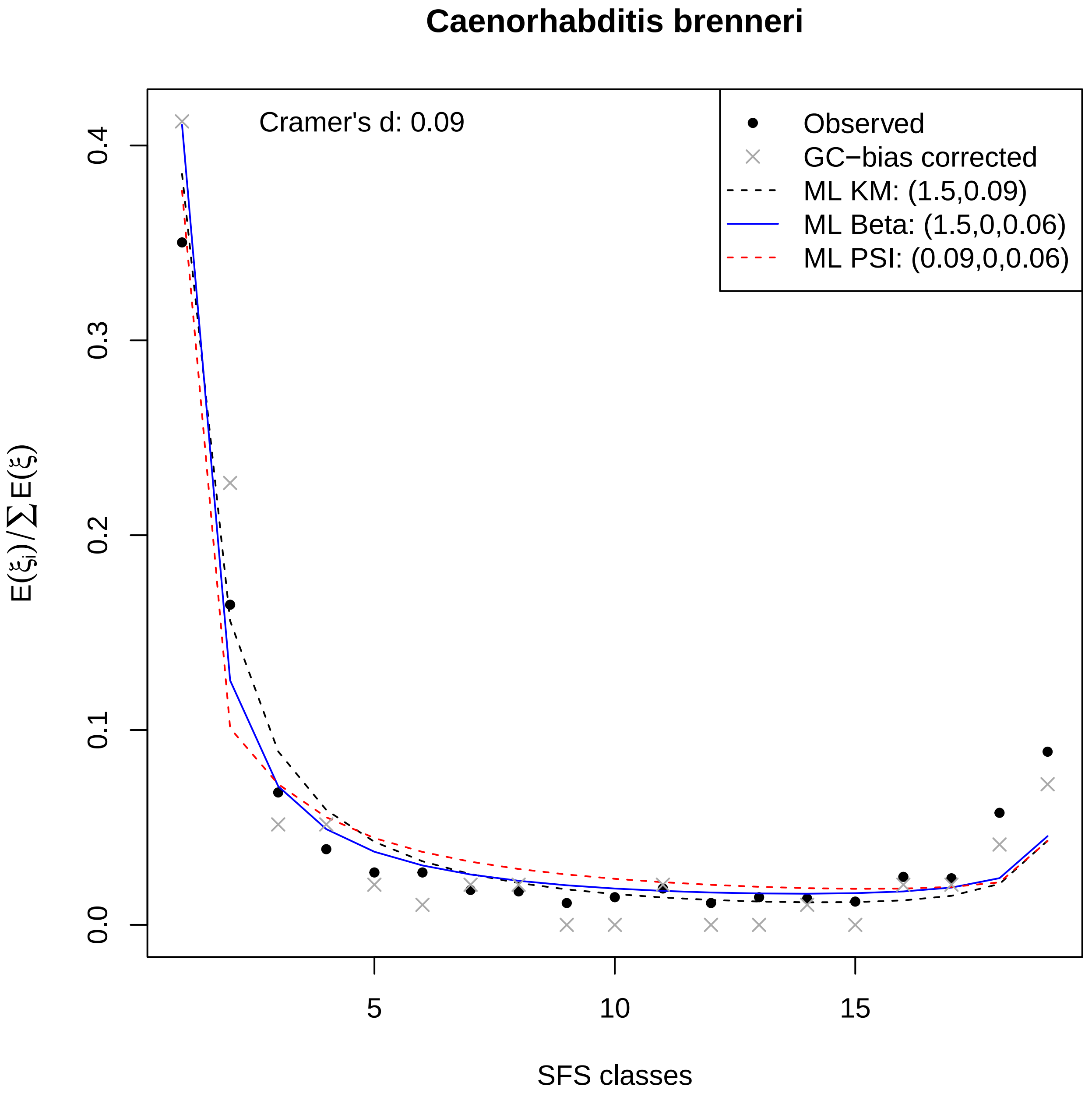

Caenorhabditis brenneri
Cramer's d: 0.09
Observed
GC−bias corrected
ML KM: (1.5,0.09)
ML Beta: (1.5,0,0.06)
ML PSI: (0.09,0,0.06)
0.0
0.1
0.2
0.3
0.4
5
10
15
SFS classes
$E(\xi_i)/\sum E(\xi)$

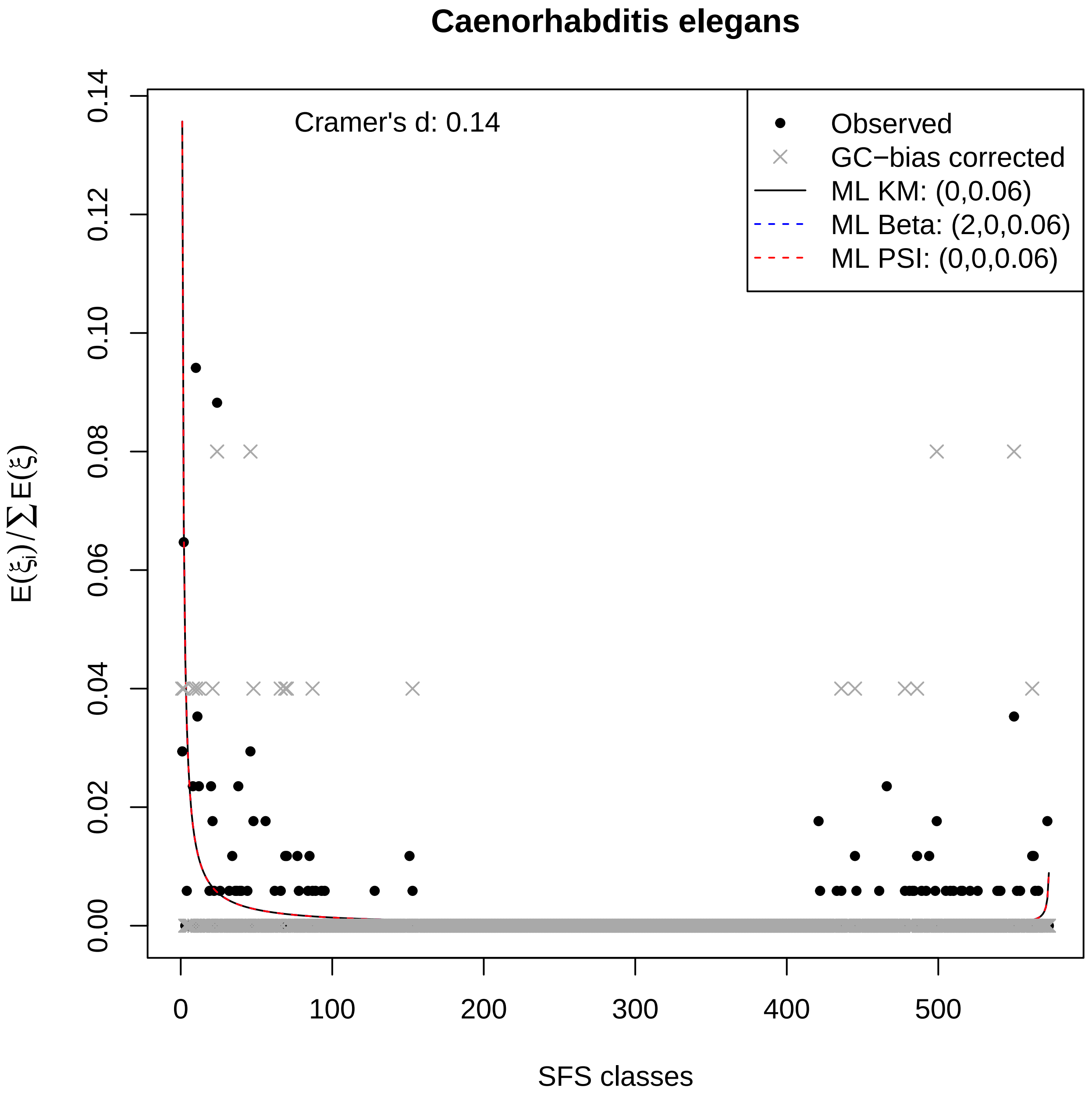

Caenorhabditis elegans
Cramer's d: 0.14
Observed
GC−bias corrected
ML KM: (0,0.06)
ML Beta: (2,0,0.06)
ML PSI: (0,0,0.06)
0.00
0.02
0.04
0.06
0.08
0.10
0.12
0.14
0
100
200
300
400
500
SFS classes
$E(\xi_i)/\sum E(\xi)$

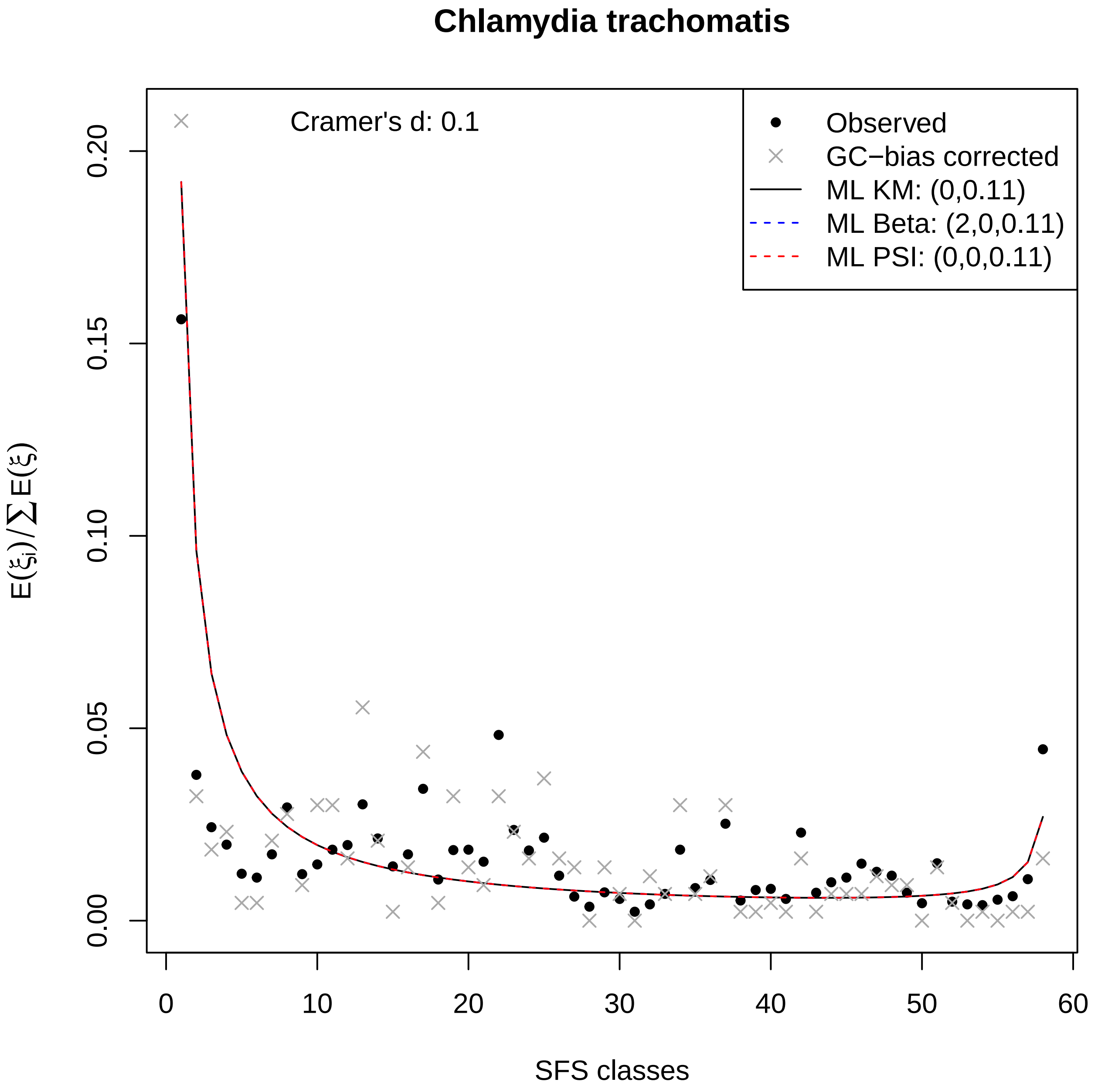

Chlamydia trachomatis
Cramer's d: 0.1
Observed
GC−bias corrected
ML KM: (0,0.11)
ML Beta: (2,0,0.11)
ML PSI: (0,0,0.11)
$E(\xi_i)/\sum E(\xi)$
SFS classes
0.00
0.05
0.10
0.15
0.20
0
10
20
30
40
50
60

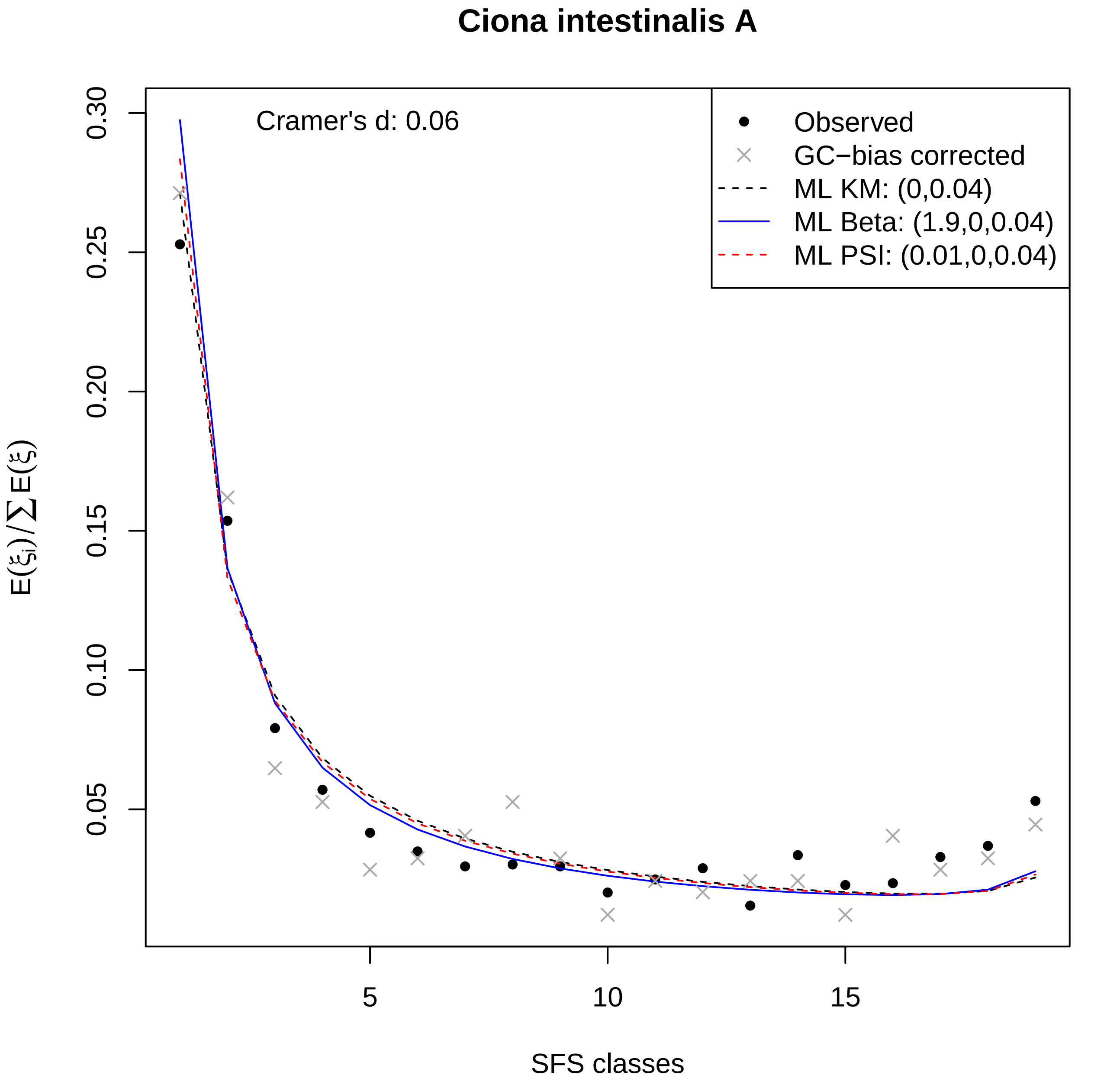

Ciona intestinalis A
Cramer's d: 0.06
Observed
GC−bias corrected
ML KM: (0,0.04)
ML Beta: (1.9,0,0.04)
ML PSI: (0.01,0,0.04)
0.30
0.25
0.20
0.15
0.10
0.05
$E(\xi_i)/\sum E(\xi)$
5
10
15
SFS classes

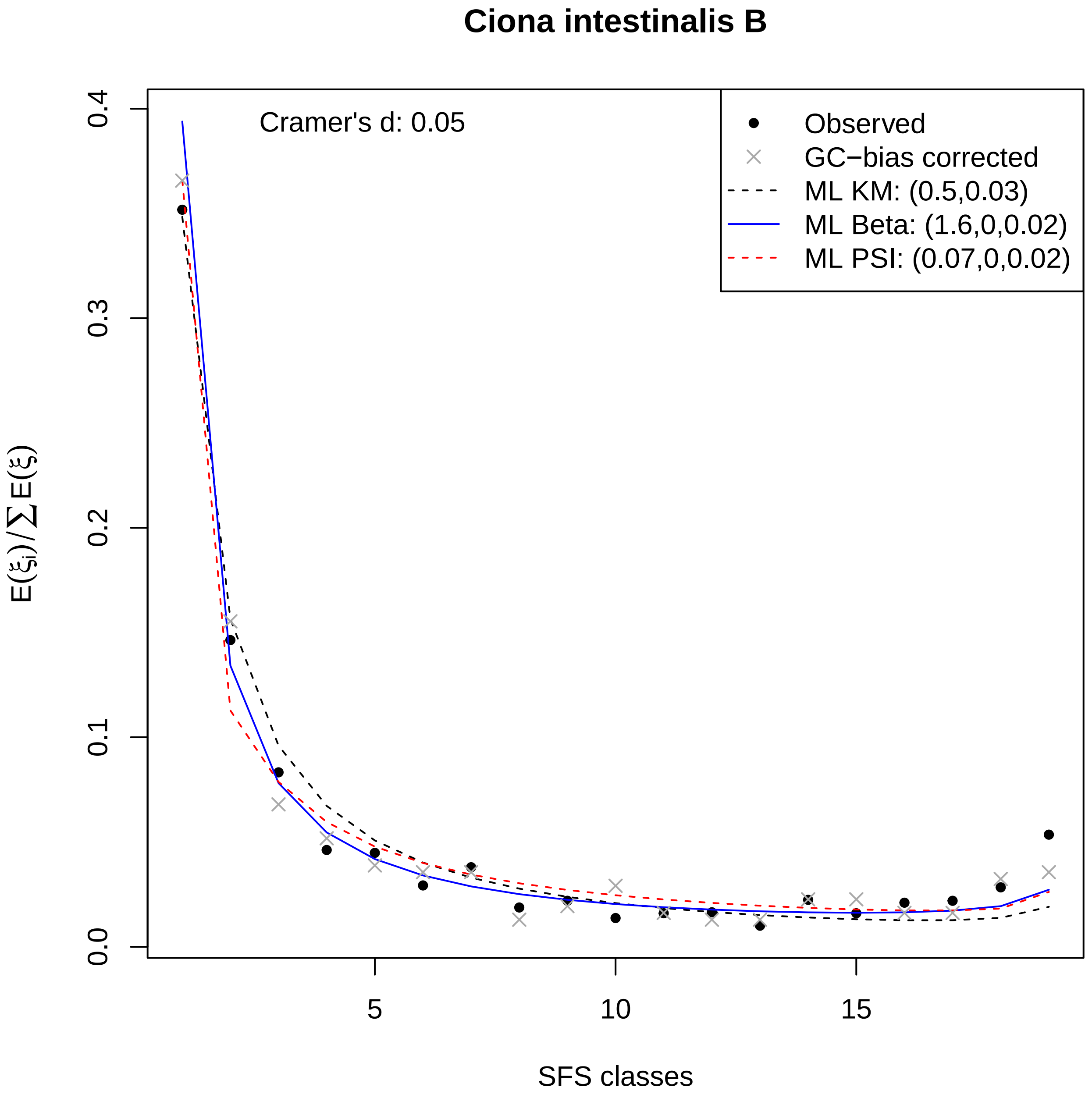

Ciona intestinalis B
Cramer's d: 0.05
Observed
GC−bias corrected
ML KM: (0.5,0.03)
ML Beta: (1.6,0,0.02)
ML PSI: (0.07,0,0.02)
$E(\xi_i)/\sum E(\xi)$
0.0
0.1
0.2
0.3
0.4
5
10
15
SFS classes

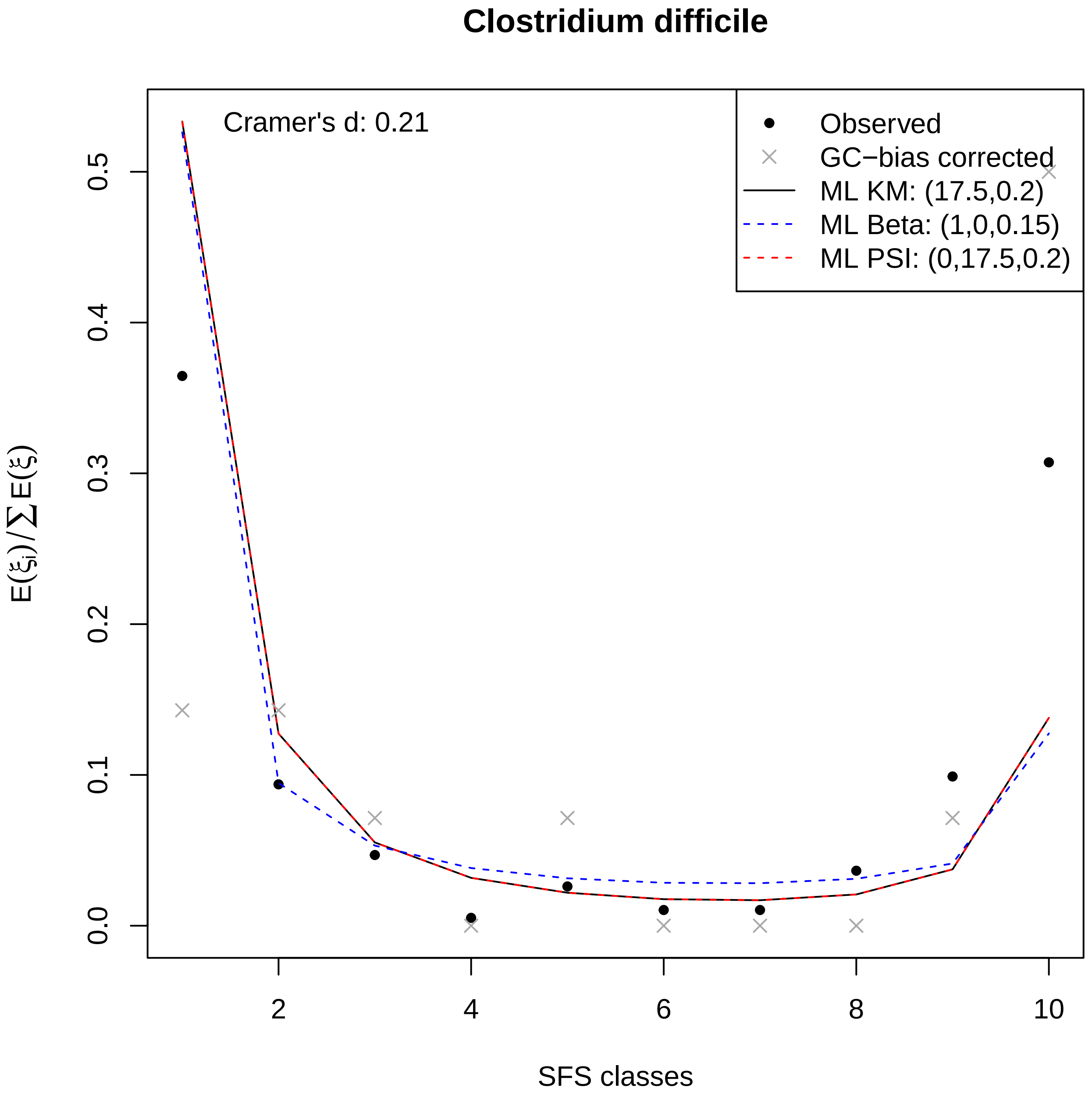

Clostridium difficile
Cramer's d: 0.21
Observed
GC−bias corrected
ML KM: (17.5,0.2)
ML Beta: (1,0,0.15)
ML PSI: (0,17.5,0.2)
0.0
0.1
0.2
0.3
0.4
0.5
$E(\xi_i)/\sum E(\xi)$
2
4
6
8
10
SFS classes

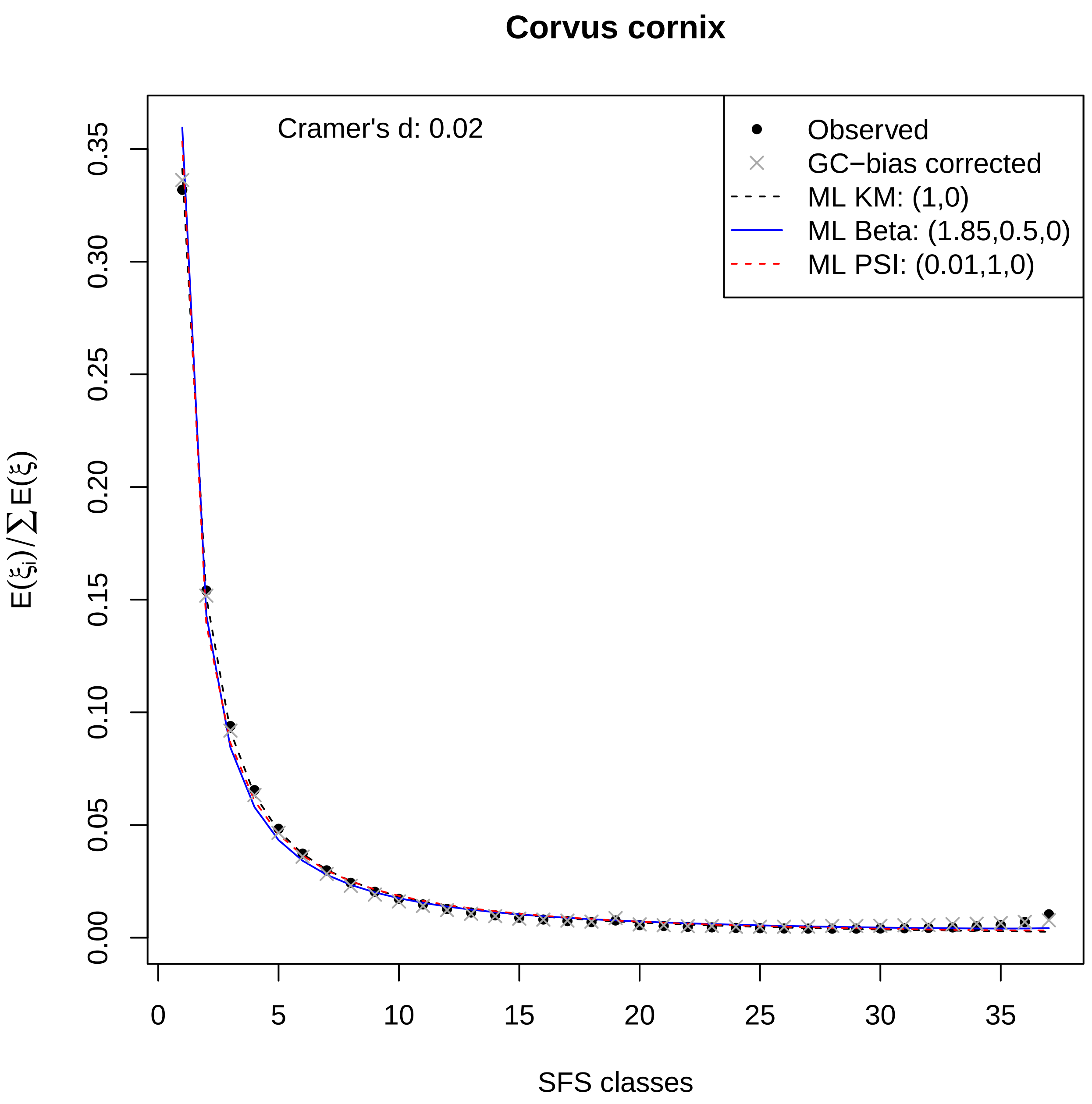

Corvus cornix
Cramer's d: 0.02
Observed
GC−bias corrected
ML KM: (1,0)
ML Beta: (1.85,0.5,0)
ML PSI: (0.01,1,0)
0.00
0.05
0.10
0.15
0.20
0.25
0.30
0.35
0
5
10
15
20
25
30
35
SFS classes

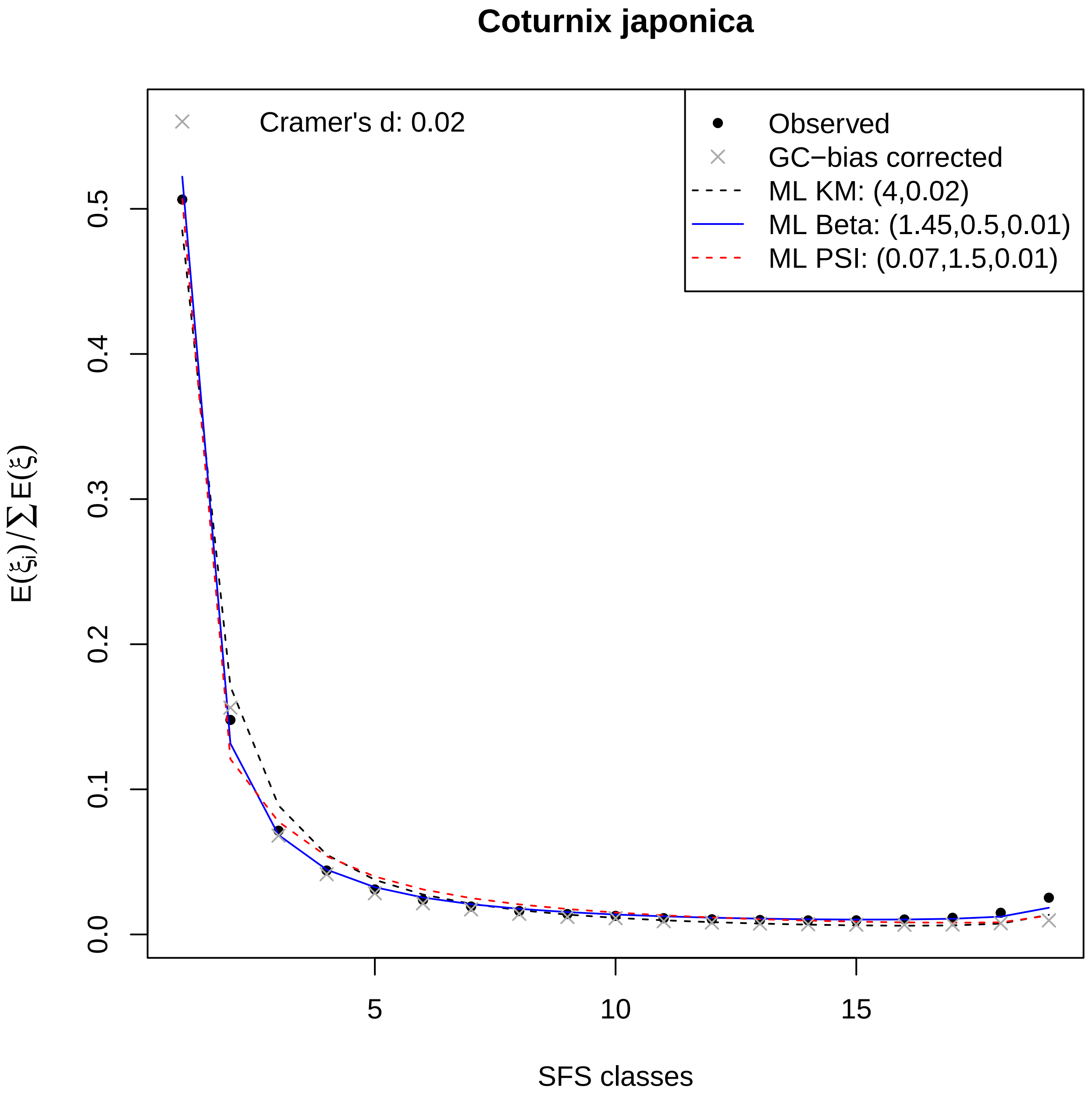

Coturnix japonica
Cramer's d: 0.02
Observed
GC−bias corrected
ML KM: (4,0.02)
ML Beta: (1.45,0.5,0.01)
ML PSI: (0.07,1.5,0.01)
$E(\xi_i)/\sum E(\xi)$
0.0
0.1
0.2
0.3
0.4
0.5
5
10
15
SFS classes

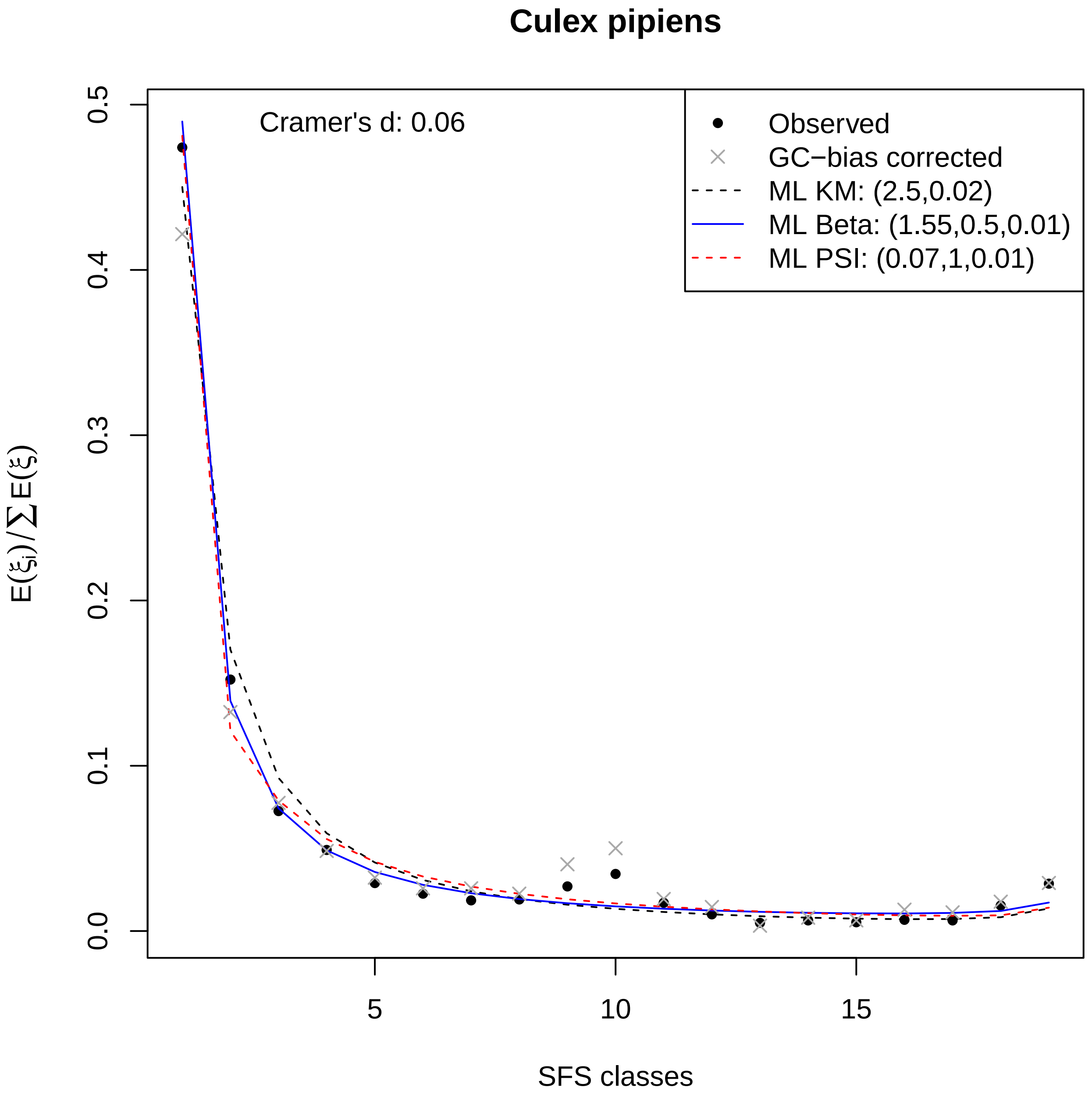
Culex pipiens
Cramer's d: 0.06
Observed
GC−bias corrected
ML KM: (2.5,0.02)
ML Beta: (1.55,0.5,0.01)
ML PSI: (0.07,1,0.01)
$E(\xi_i)/\sum E(\xi)$
SFS classes
0.0
0.1
0.2
0.3
0.4
0.5
5
10
15

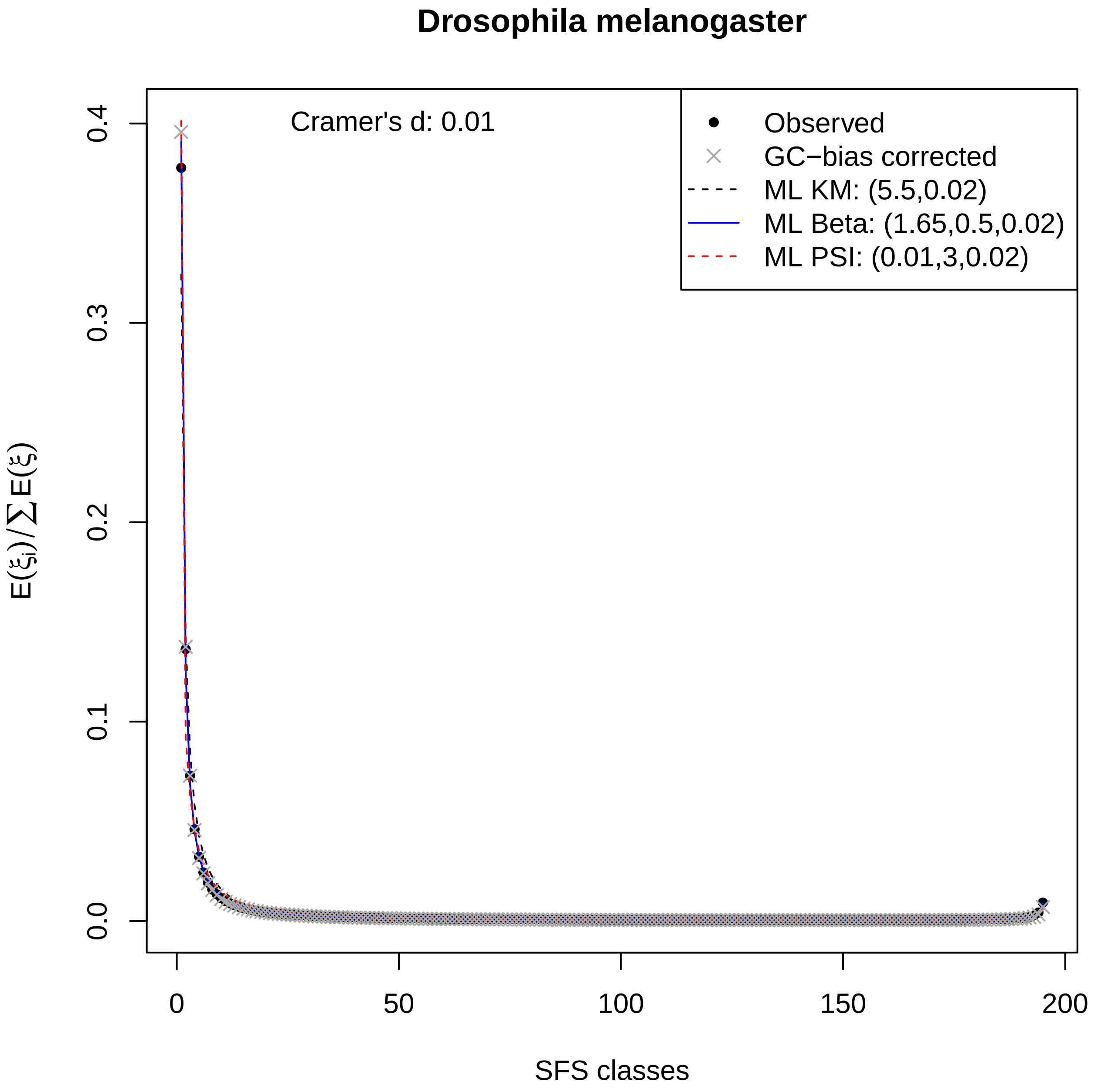

Drosophila melanogaster
Cramer's d: 0.01
Observed
GC−bias corrected
ML KM: (5.5,0.02)
ML Beta: (1.65,0.5,0.02)
ML PSI: (0.01,3,0.02)
0.0
0.1
0.2
0.3
0.4
0
50
100
150
200
SFS classes
$E(\xi_i)/\sum E(\xi)$

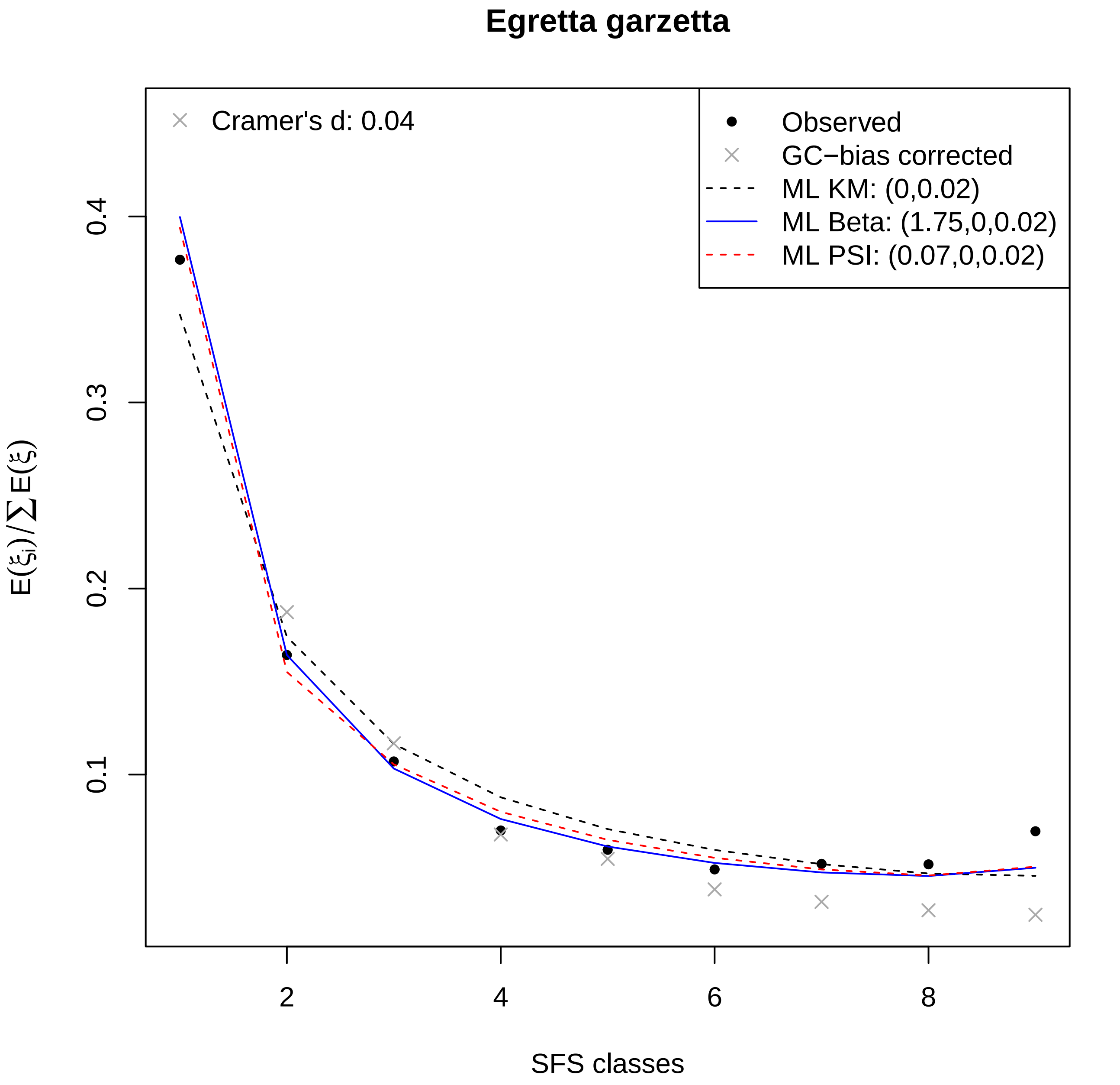

Egretta garzetta
Cramer's d: 0.04
Observed
GC−bias corrected
ML KM: (0,0.02)
ML Beta: (1.75,0,0.02)
ML PSI: (0.07,0,0.02)
0.1
0.2
0.3
0.4
2
4
6
8
SFS classes
$E(\xi_i)/\sum E(\xi)$

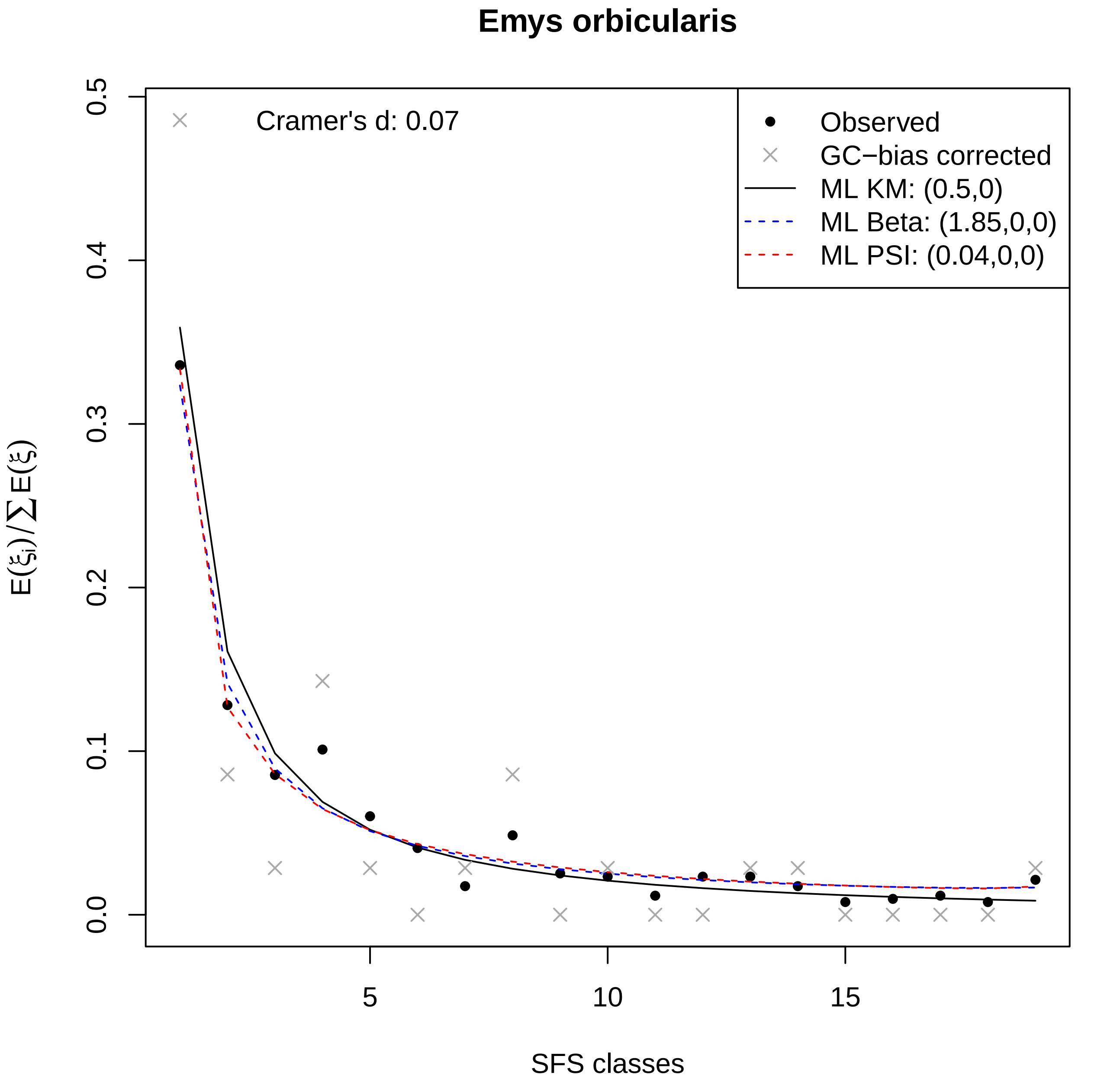

Emys orbicularis
Cramer's d: 0.07
Observed
GC−bias corrected
ML KM: (0.5,0)
ML Beta: (1.85,0,0)
ML PSI: (0.04,0,0)
0.0
0.1
0.2
0.3
0.4
0.5
5
10
15
SFS classes
$E(\xi_i)/\sum E(\xi)$

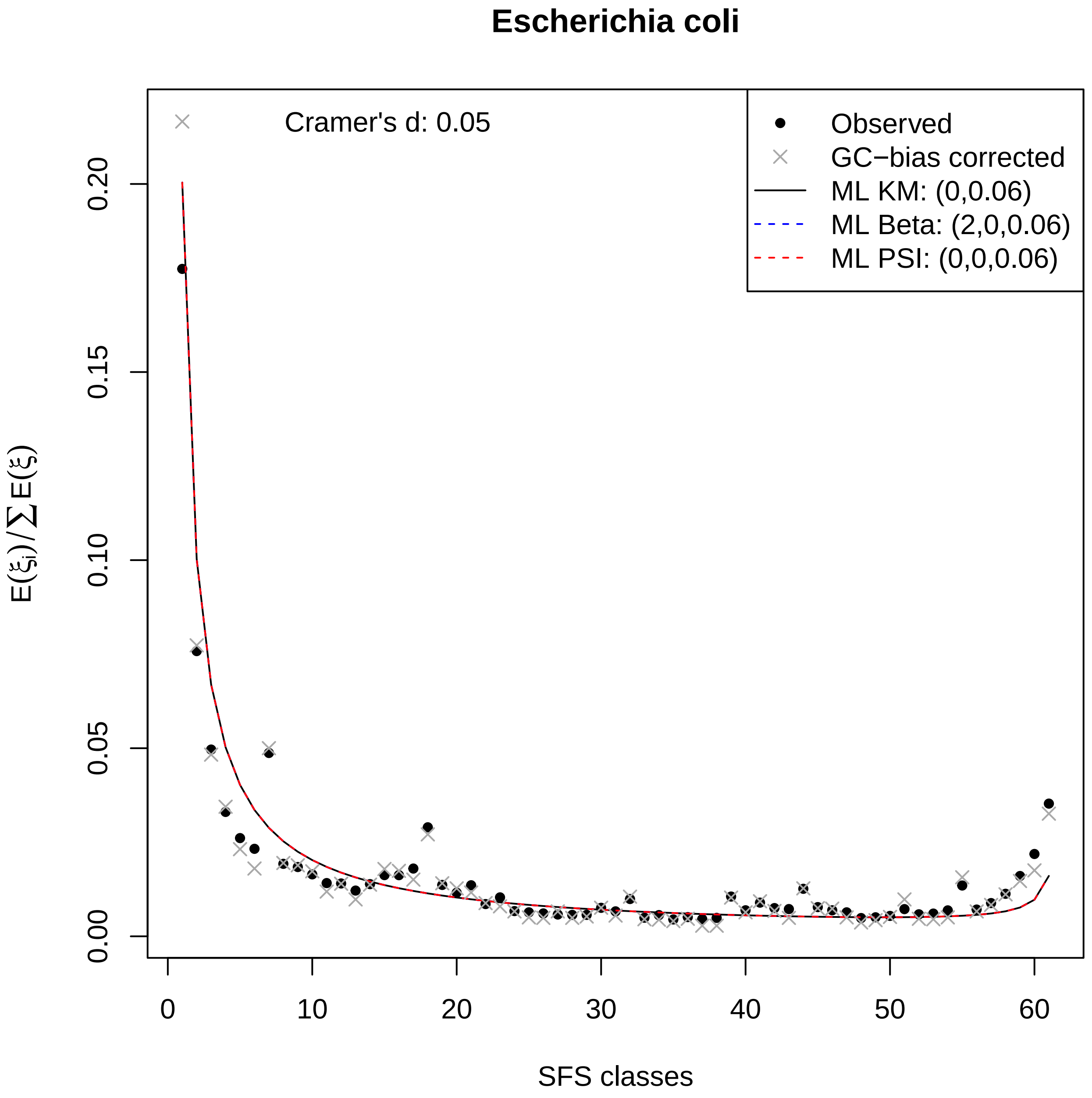

Escherichia coli
Cramer's d: 0.05
Observed
GC−bias corrected
ML KM: (0,0.06)
ML Beta: (2,0,0.06)
ML PSI: (0,0,0.06)
$E(\xi_i)/\sum E(\xi)$
0.00
0.05
0.10
0.15
0.20
0
10
20
30
40
50
60
SFS classes

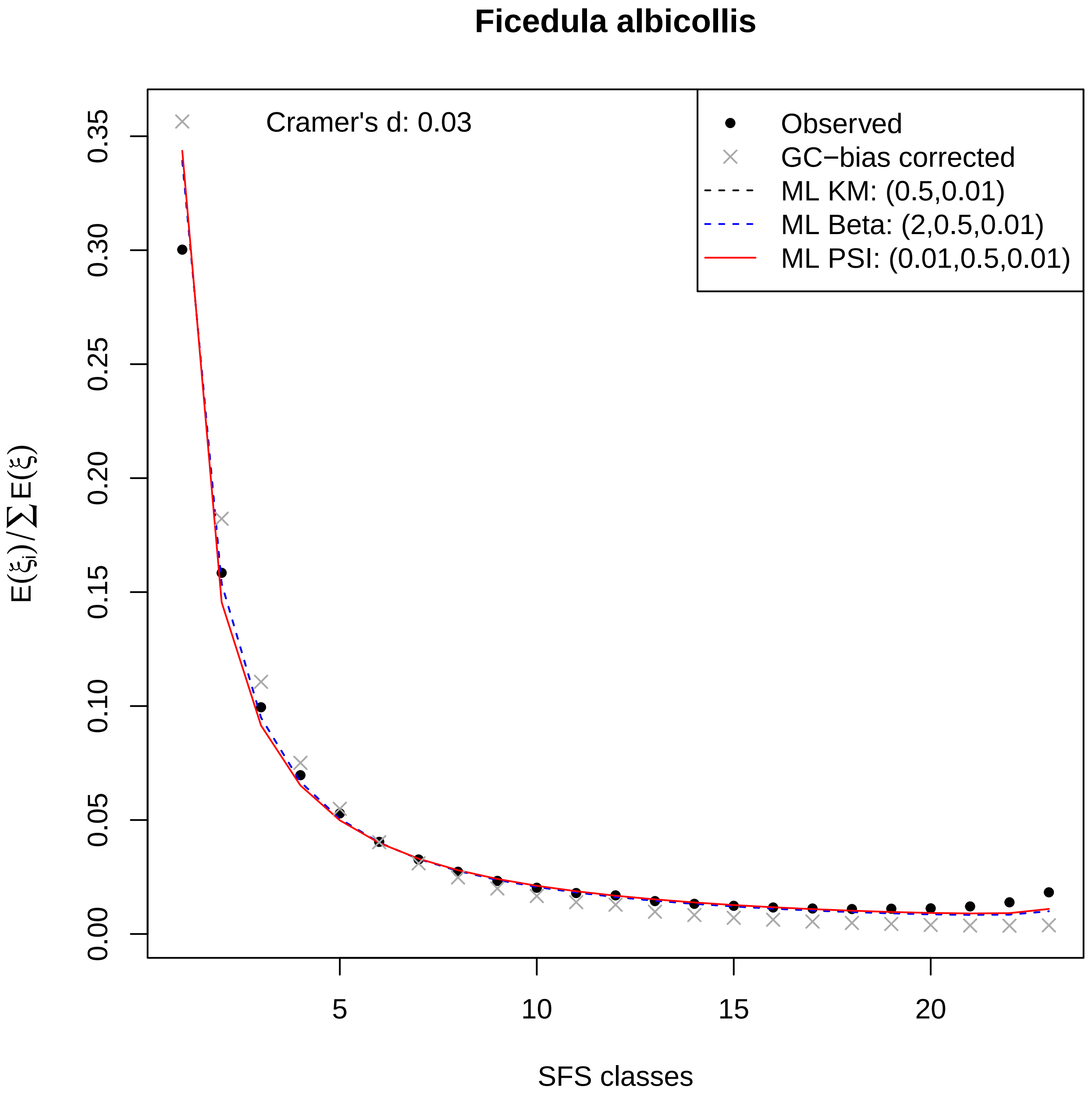

Ficedula albicollis
Cramer's d: 0.03
Observed
GC−bias corrected
ML KM: (0.5,0.01)
ML Beta: (2,0.5,0.01)
ML PSI: (0.01,0.5,0.01)
0.00
0.05
0.10
0.15
0.20
0.25
0.30
0.35
$E(\xi_i)/\sum E(\xi)$
5
10
15
20
SFS classes

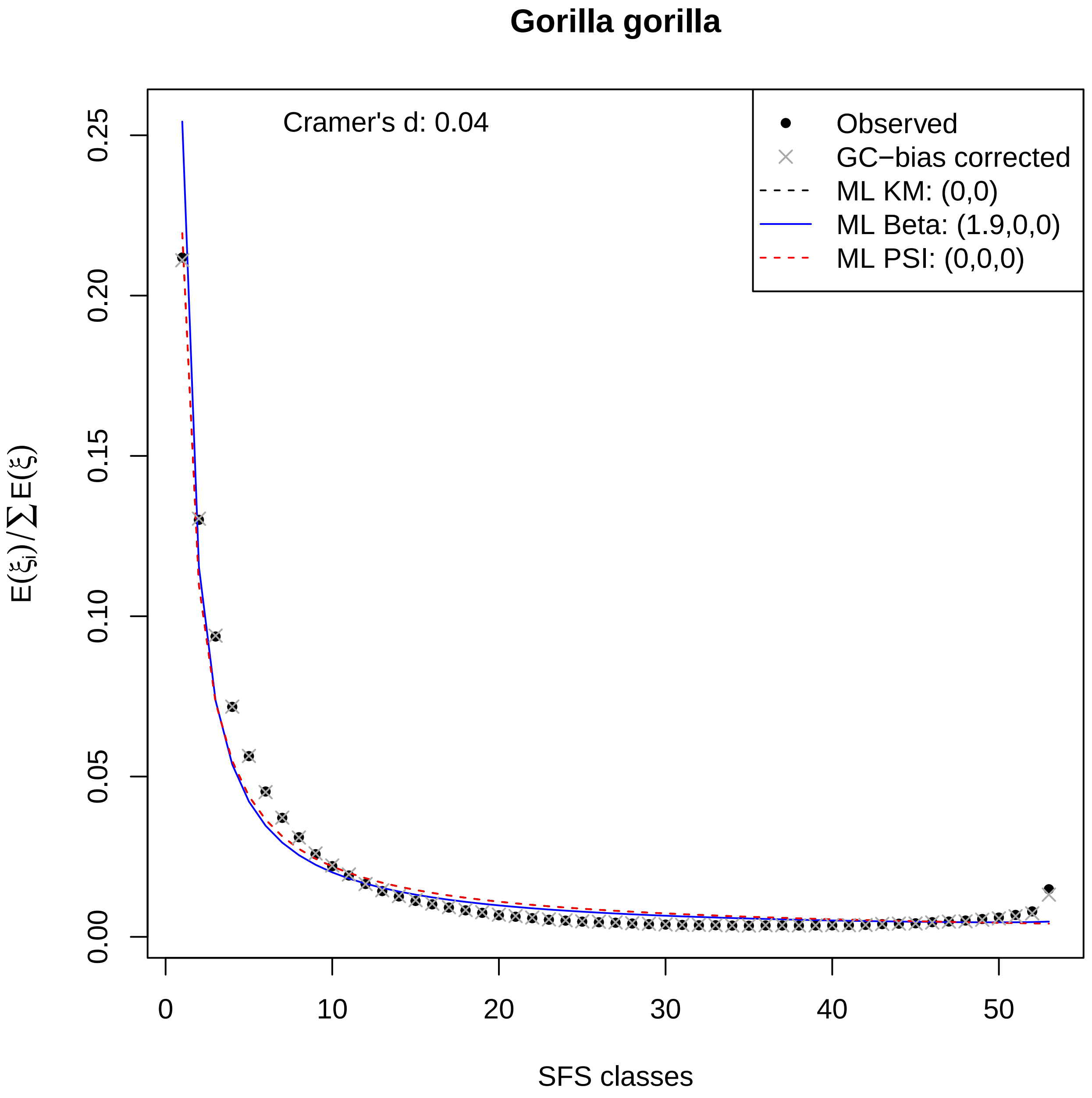

Gorilla gorilla
Cramer's d: 0.04
Observed
GC−bias corrected
ML KM: (0,0)
ML Beta: (1.9,0,0)
ML PSI: (0,0,0)
0.00
0.05
0.10
0.15
0.20
0.25
0
10
20
30
40
50
SFS classes
$E(\xi_i)/\sum E(\xi)$

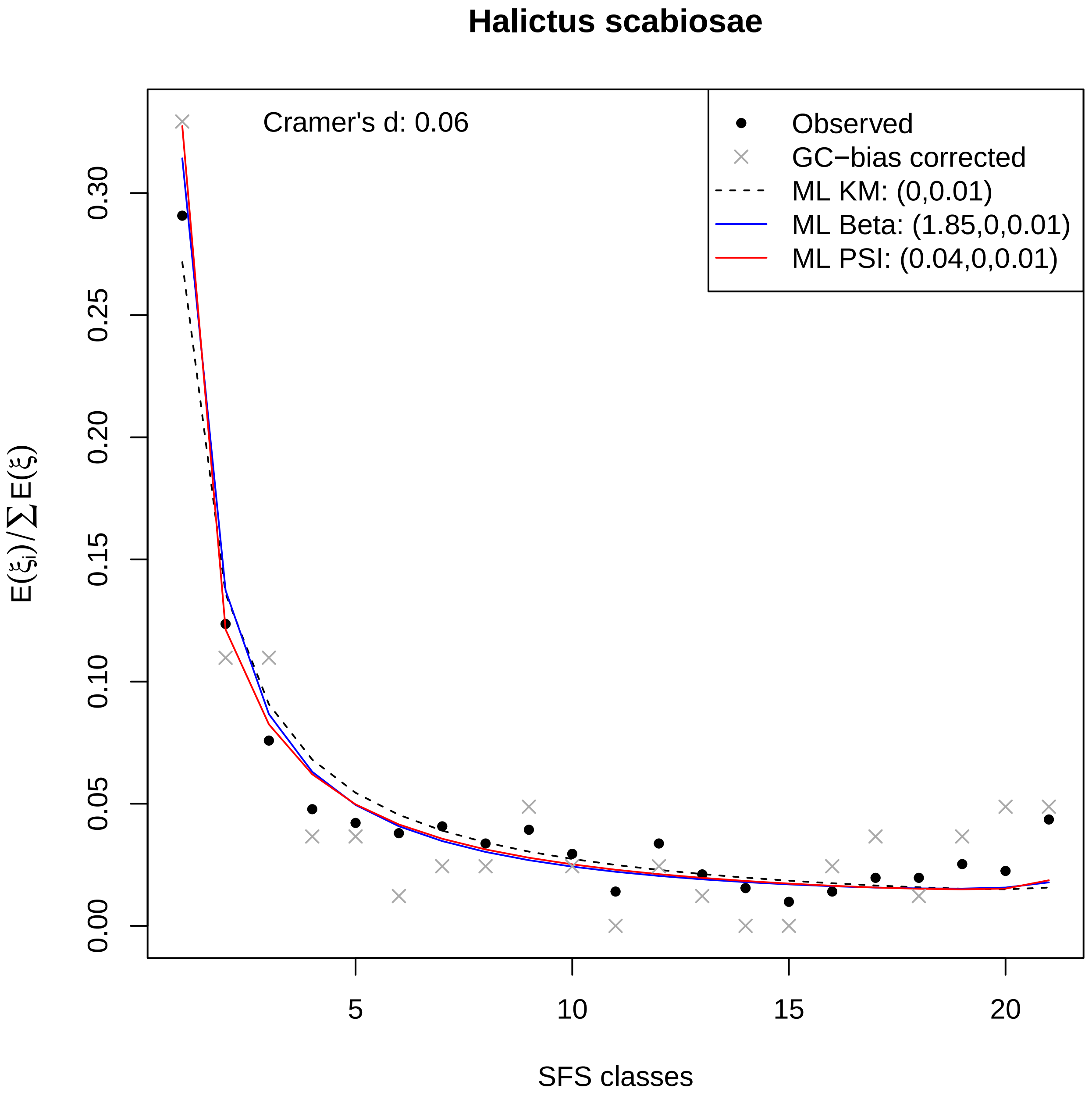

Halictus scabiosae
Cramer's d: 0.06
Observed
GC−bias corrected
ML KM: (0,0.01)
ML Beta: (1.85,0,0.01)
ML PSI: (0.04,0,0.01)
0.00
0.05
0.10
0.15
0.20
0.25
0.30
5
10
15
20
SFS classes
$E(\xi_i)/\sum E(\xi)$

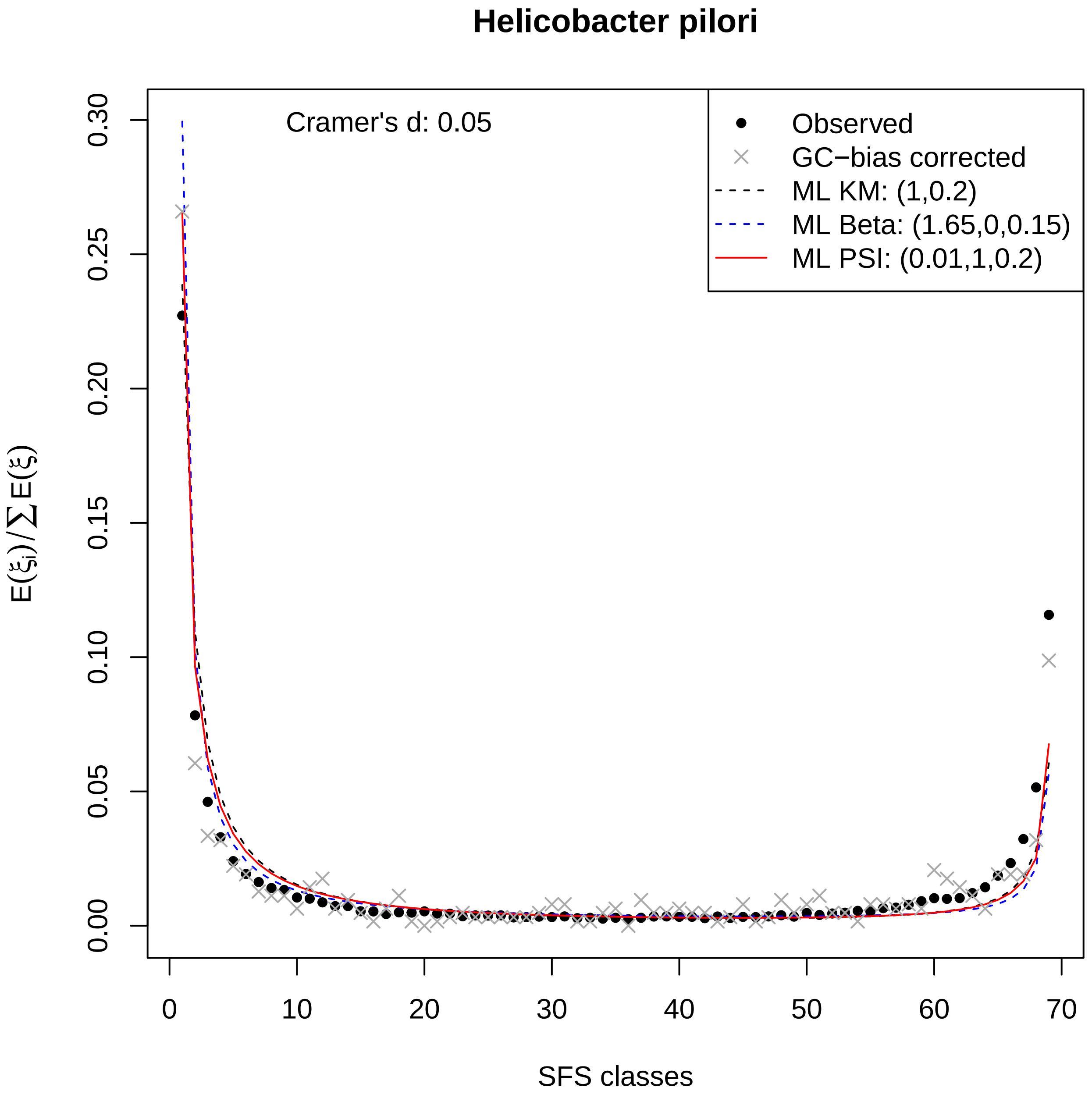

Helicobacter pilori
Cramer's d: 0.05
Observed
GC−bias corrected
ML KM: (1,0.2)
ML Beta: (1.65,0,0.15)
ML PSI: (0.01,1,0.2)
$E(\xi_i)/\sum E(\xi)$
0.00
0.05
0.10
0.15
0.20
0.25
0.30
0
10
20
30
40
50
60
70
SFS classes

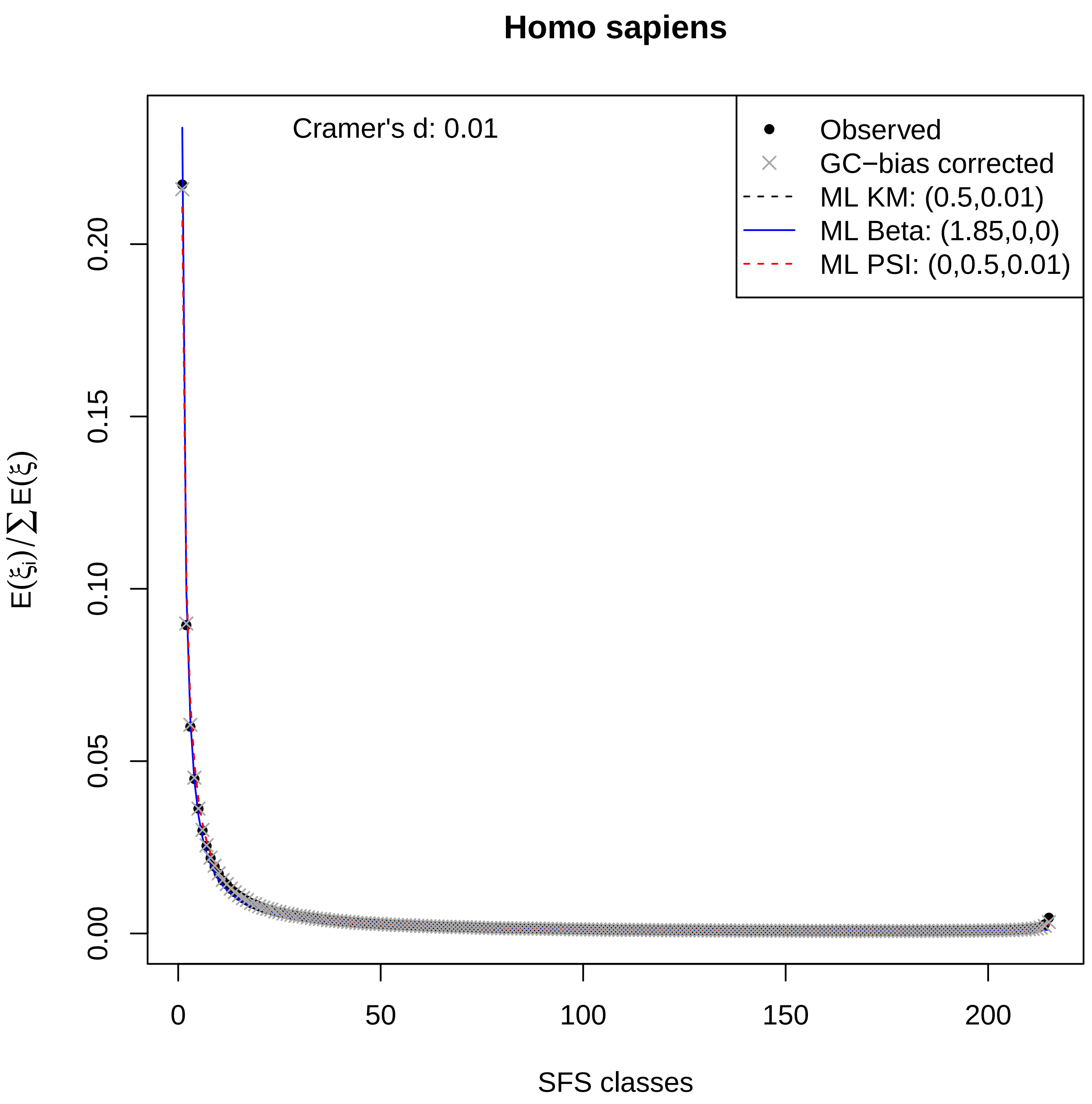

Homo sapiens
Cramer's d: 0.01
Observed
GC−bias corrected
ML KM: (0.5,0.01)
ML Beta: (1.85,0,0)
ML PSI: (0,0.5,0.01)
$E(\xi_i)/\sum E(\xi)$
0.00
0.05
0.10
0.15
0.20
0
50
100
150
200
SFS classes

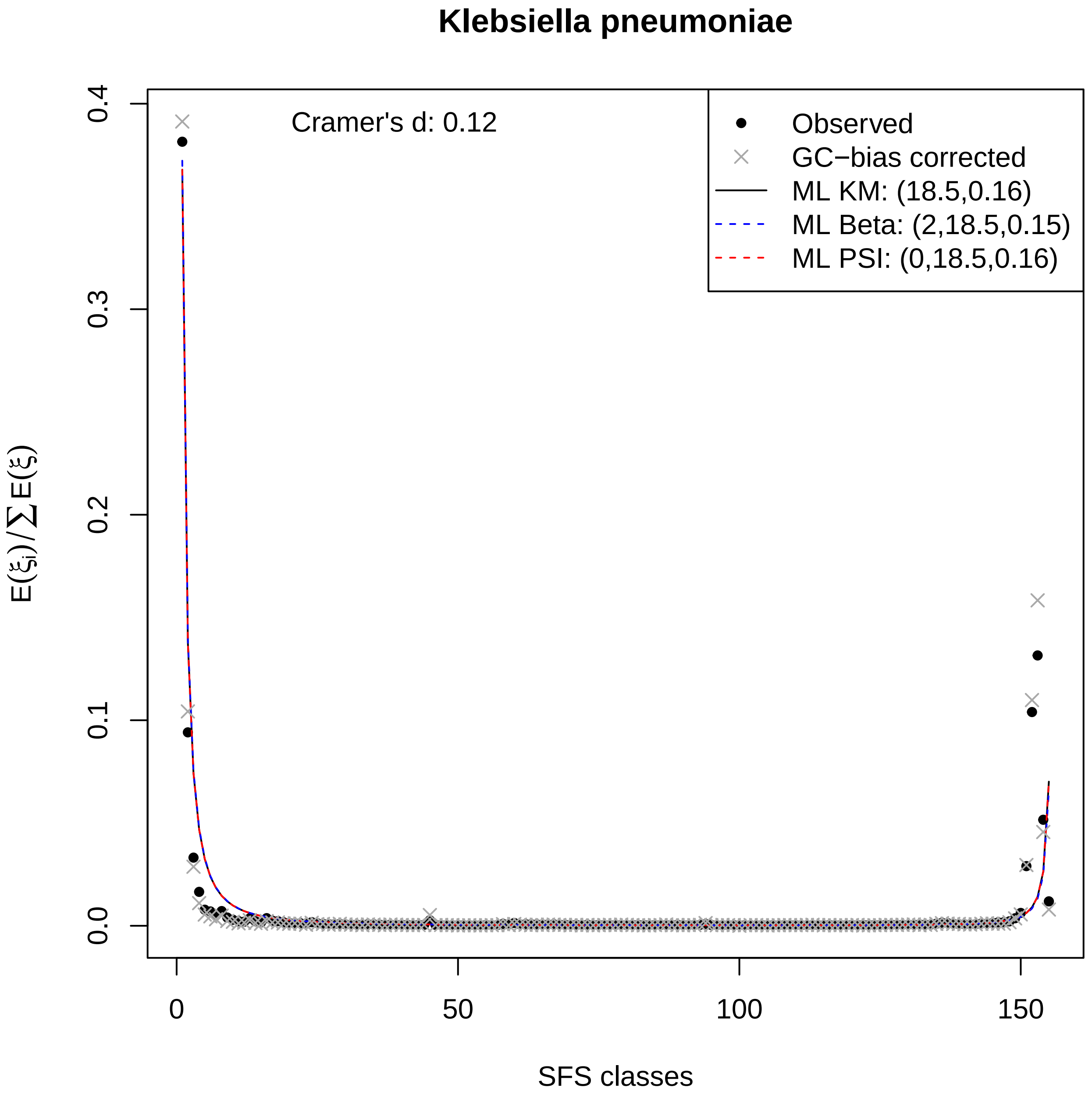

Klebsiella pneumoniae
Cramer's d: 0.12
Observed
GC−bias corrected
ML KM: (18.5,0.16)
ML Beta: (2,18.5,0.15)
ML PSI: (0,18.5,0.16)
E(ξi)/∑E(ξ)
0.0
0.1
0.2
0.3
0.4
0
50
100
150
SFS classes

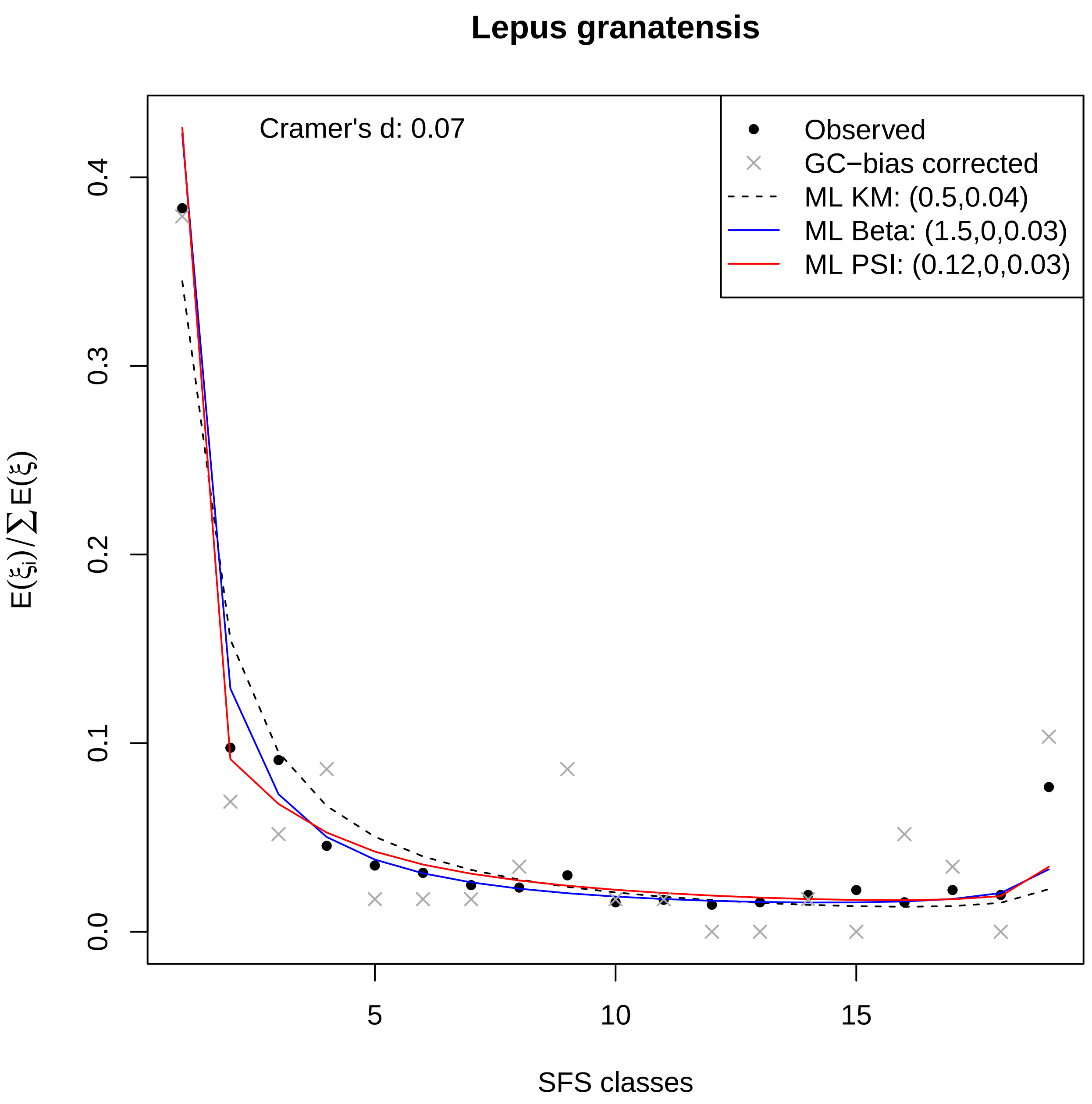

Lepus granatensis
Cramer's d: 0.07
Observed
GC−bias corrected
ML KM: (0.5,0.04)
ML Beta: (1.5,0,0.03)
ML PSI: (0.12,0,0.03)
0.0
0.1
0.2
0.3
0.4
5
10
15
SFS classes
E(ξi)/∑E(ξ)

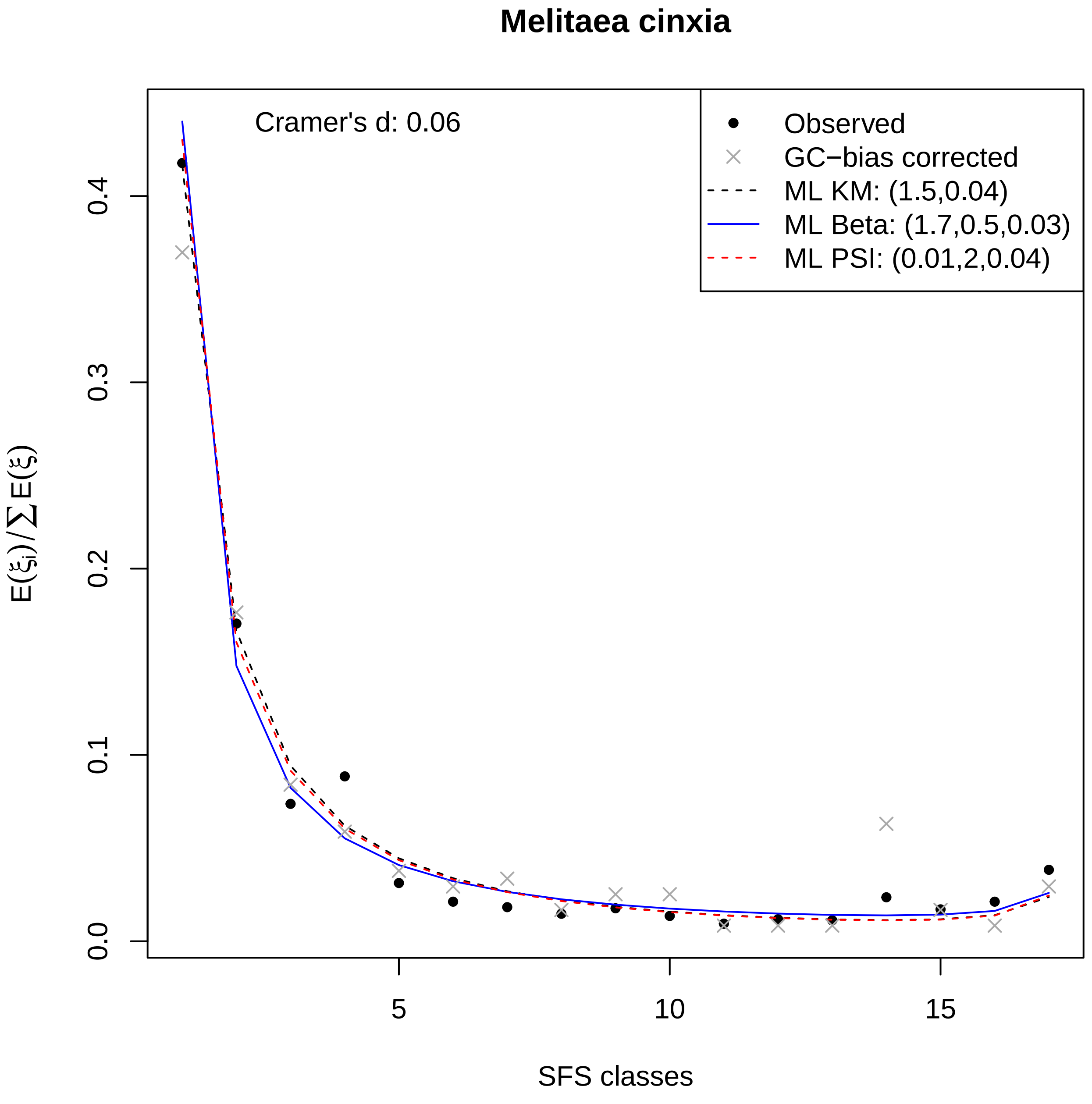

Melitaea cinxia
Cramer's d: 0.06
Observed
GC−bias corrected
ML KM: (1.5,0.04)
ML Beta: (1.7,0.5,0.03)
ML PSI: (0.01,2,0.04)
$E(\xi_i)/\sum E(\xi)$
0.0
0.1
0.2
0.3
0.4
5
10
15
SFS classes

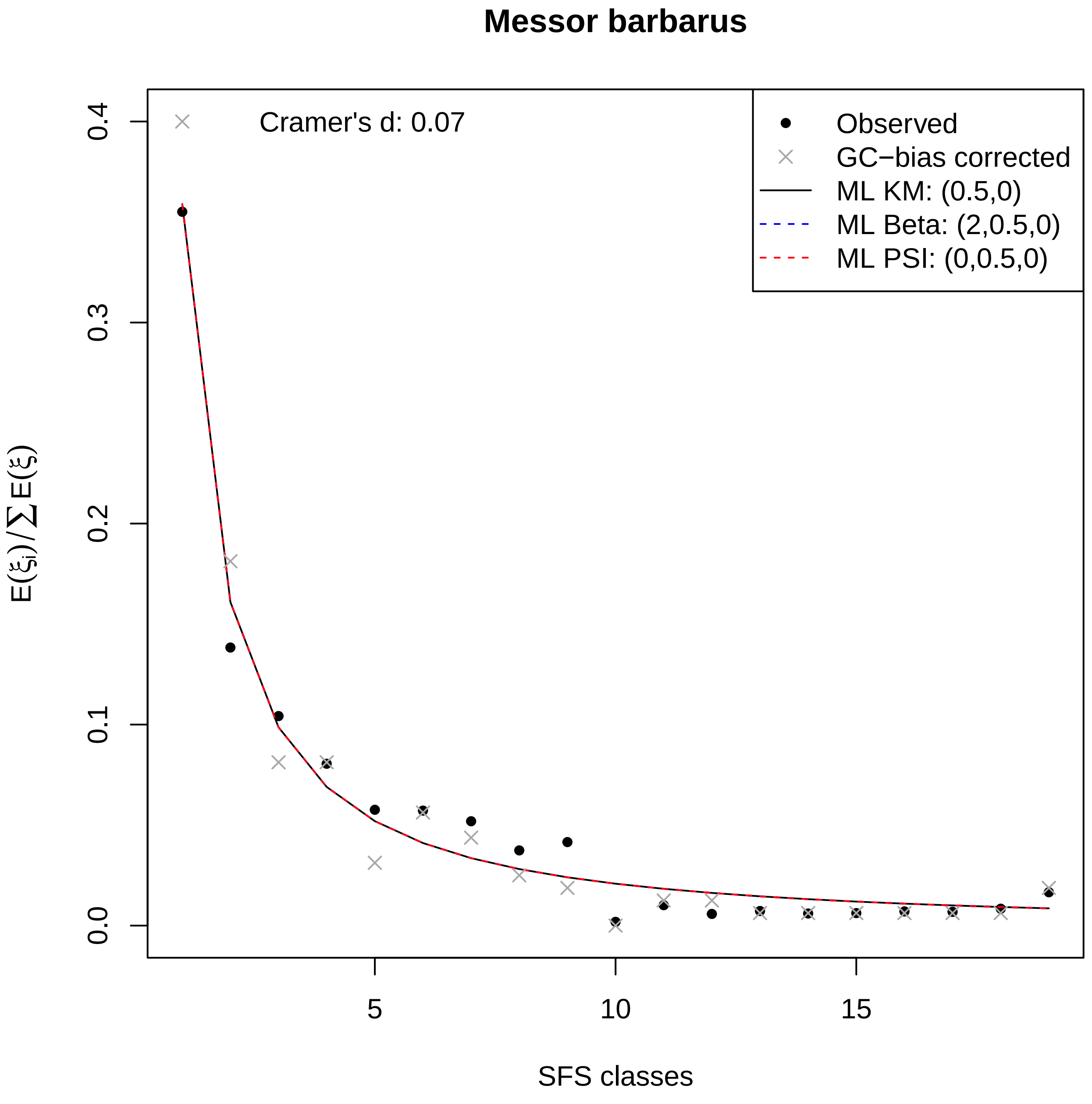

Messor barbarus
Cramer's d: 0.07
Observed
GC−bias corrected
ML KM: (0.5,0)
ML Beta: (2,0.5,0)
ML PSI: (0,0.5,0)
0.0
0.1
0.2
0.3
0.4
5
10
15
SFS classes
E(ξi)/∑E(ξ)

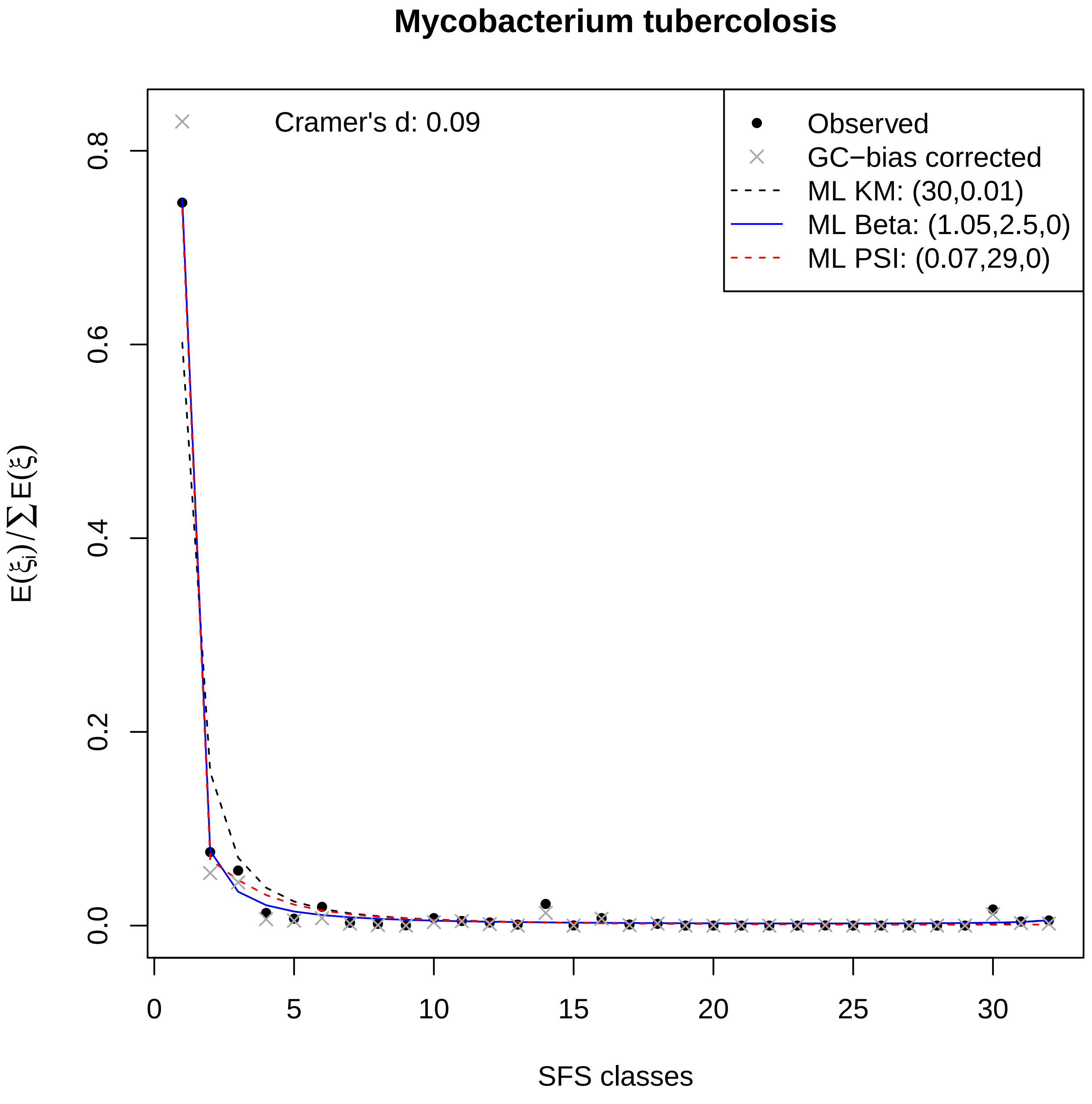

Mycobacterium tubercolosis
Cramer's d: 0.09
Observed
GC−bias corrected
ML KM: (30,0.01)
ML Beta: (1.05,2.5,0)
ML PSI: (0.07,29,0)
$E(\xi_i)/\sum E(\xi)$
SFS classes
0.0
0.2
0.4
0.6
0.8
0
5
10
15
20
25
30

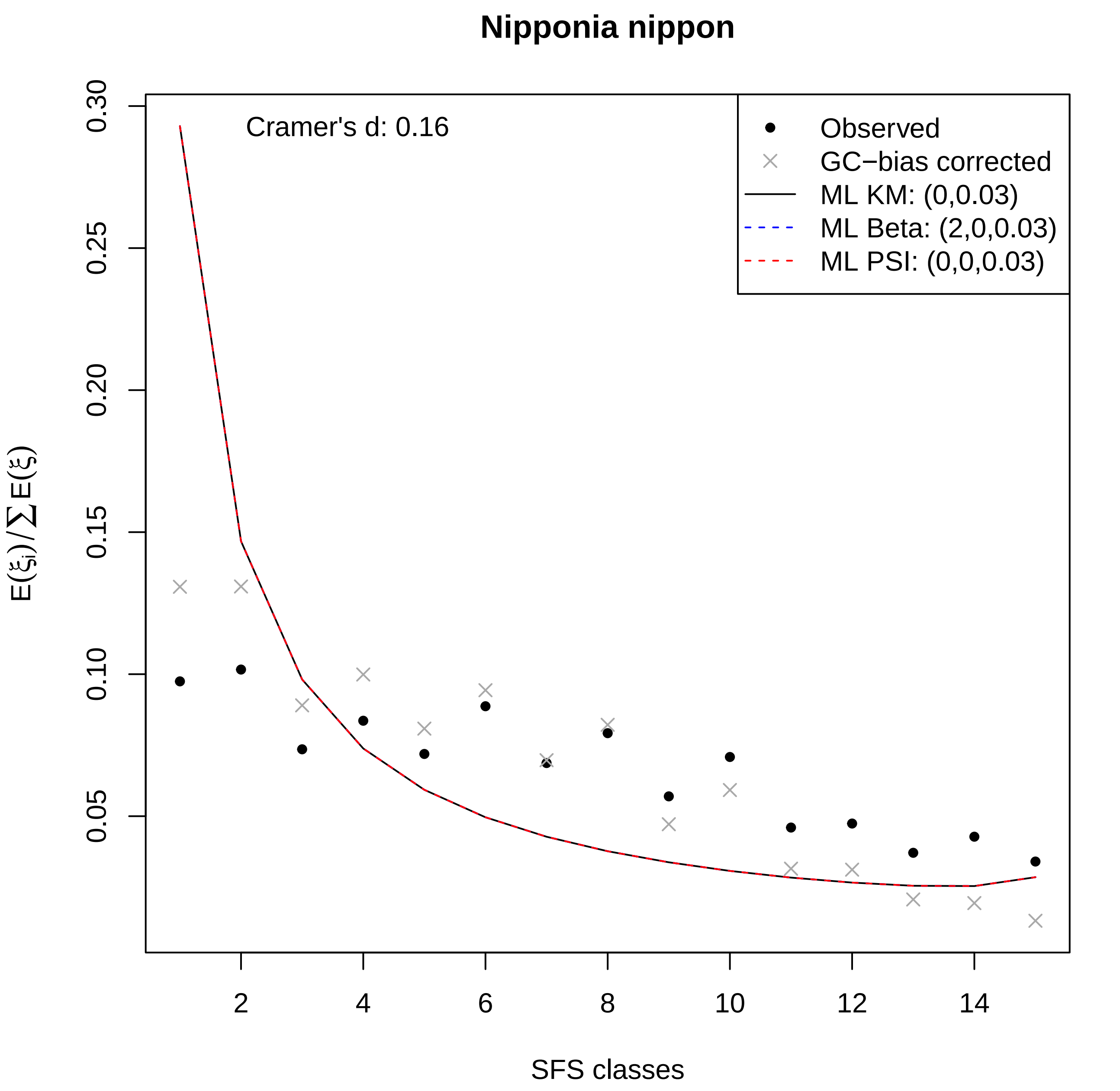

Nipponia nippon
Cramer's d: 0.16
Observed
GC−bias corrected
ML KM: (0,0.03)
ML Beta: (2,0,0.03)
ML PSI: (0,0,0.03)
0.30
0.25
0.20
0.15
0.10
0.05
2
4
6
8
10
12
14
SFS classes
$E(\xi_i)/\sum E(\xi)$

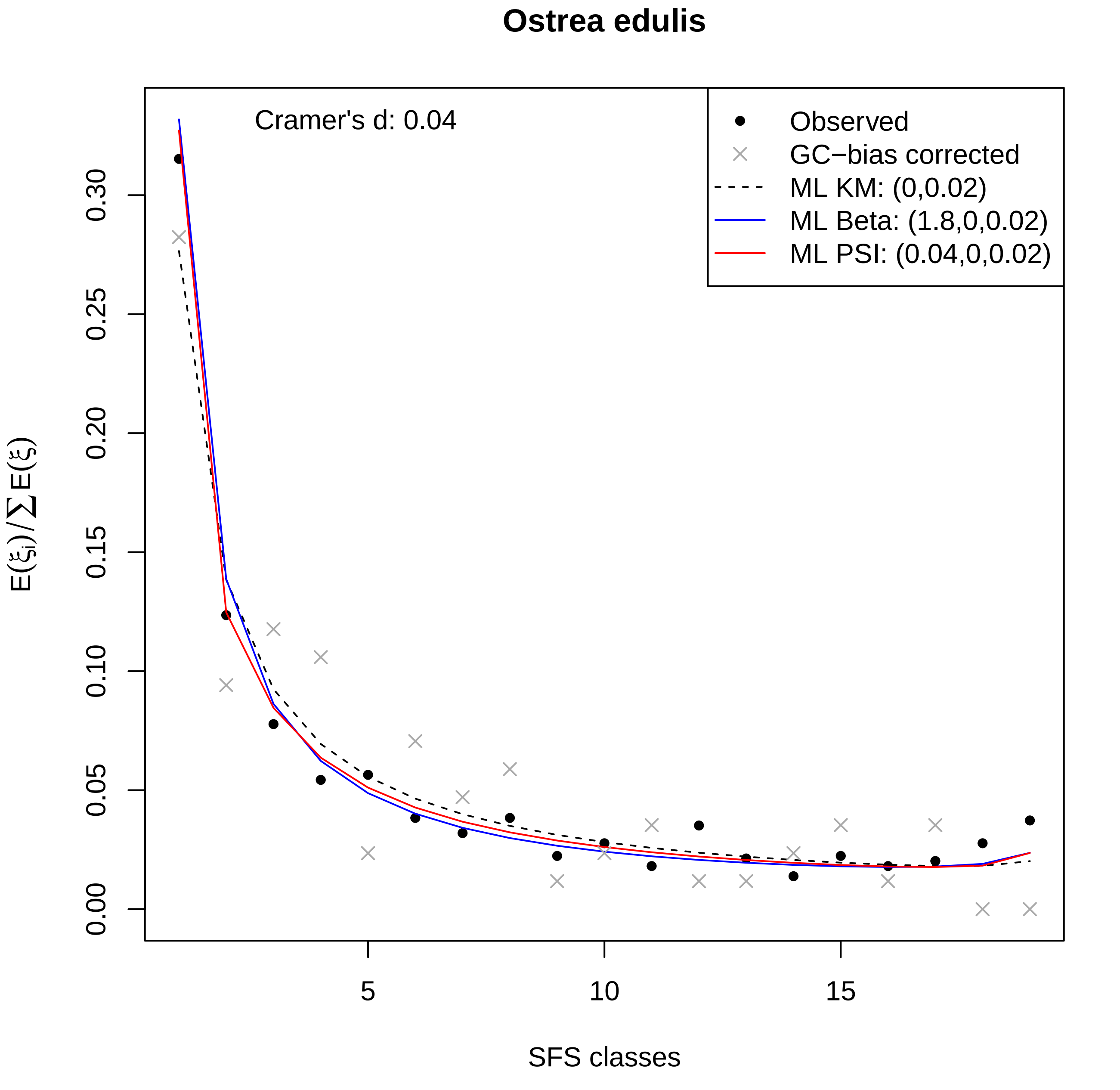
Ostrea edulis
Cramer's d: 0.04
Observed
GC−bias corrected
ML KM: (0,0.02)
ML Beta: (1.8,0,0.02)
ML PSI: (0.04,0,0.02)
$E(\xi_i)/\sum E(\xi)$
0.00
0.05
0.10
0.15
0.20
0.25
0.30
5
10
15
SFS classes

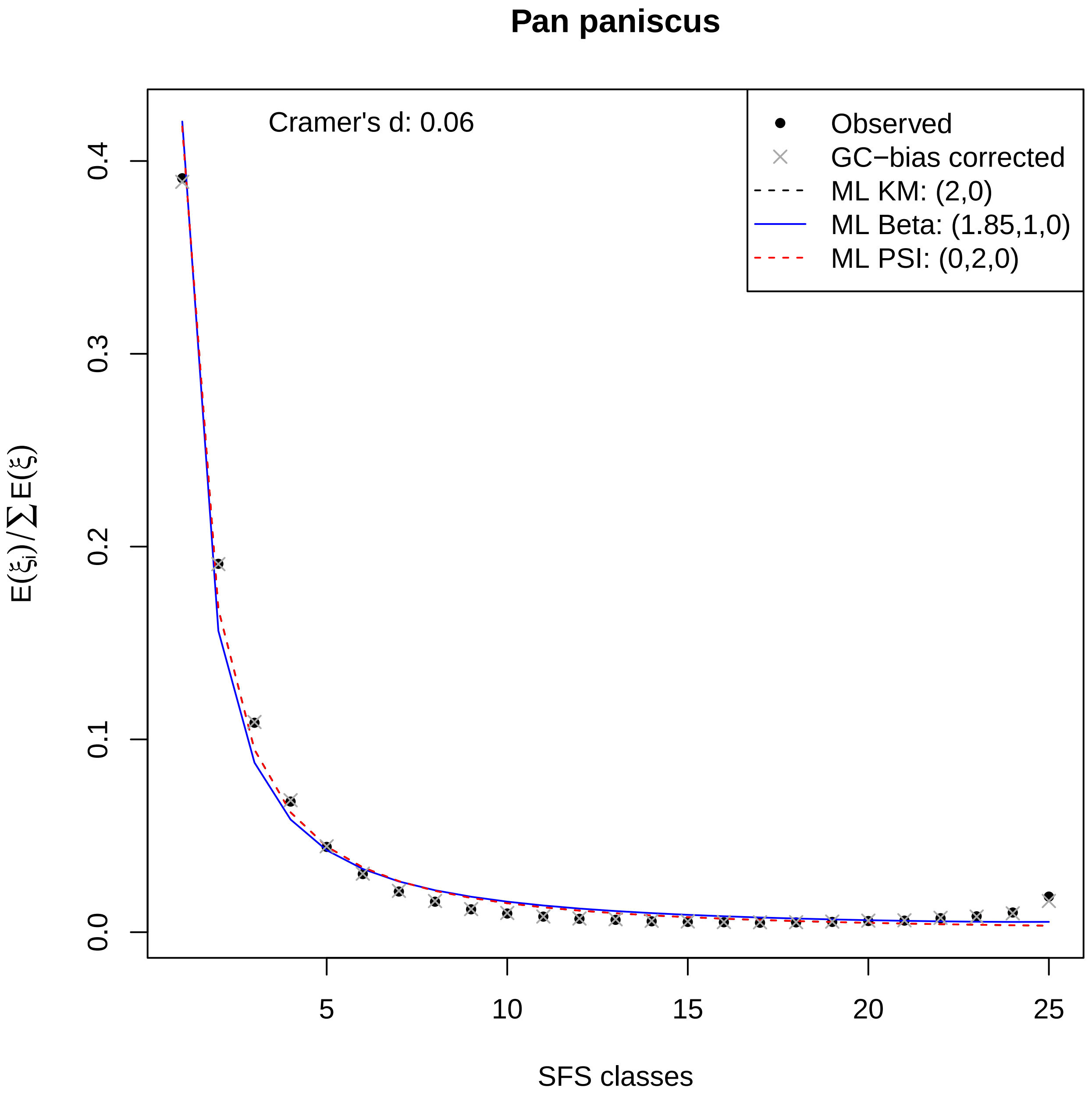
Pan paniscus
Cramer's d: 0.06
Observed
GC−bias corrected
ML KM: (2,0)
ML Beta: (1.85,1,0)
ML PSI: (0,2,0)
$E(\xi_i)/\sum E(\xi)$
0.0
0.1
0.2
0.3
0.4
5
10
15
20
25
SFS classes

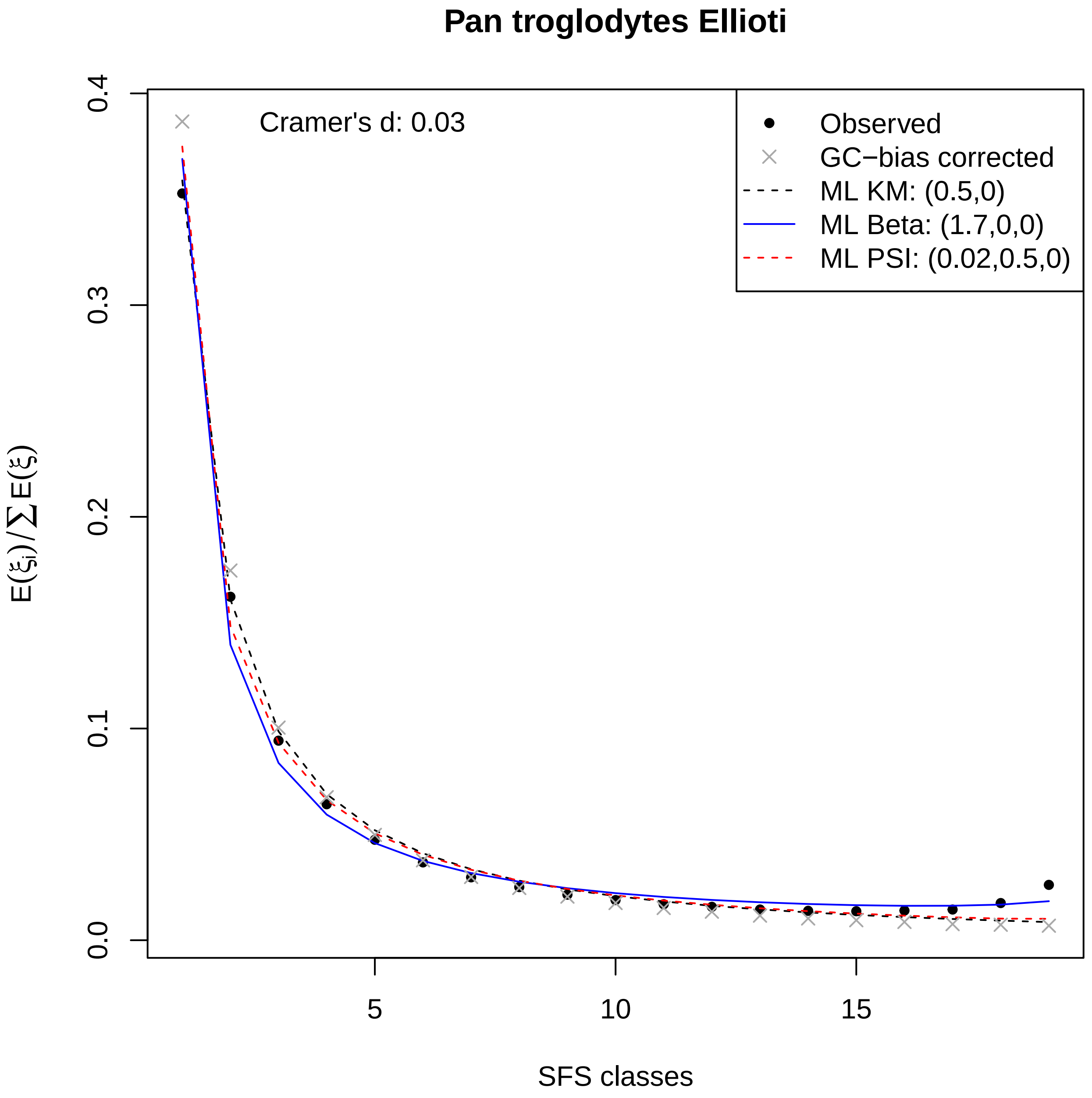

Pan troglodytes Ellioti
Cramer's d: 0.03
Observed
GC−bias corrected
ML KM: (0.5,0)
ML Beta: (1.7,0,0)
ML PSI: (0.02,0.5,0)
0.0
0.1
0.2
0.3
0.4
5
10
15
SFS classes
$E(\xi_i)/\sum E(\xi)$

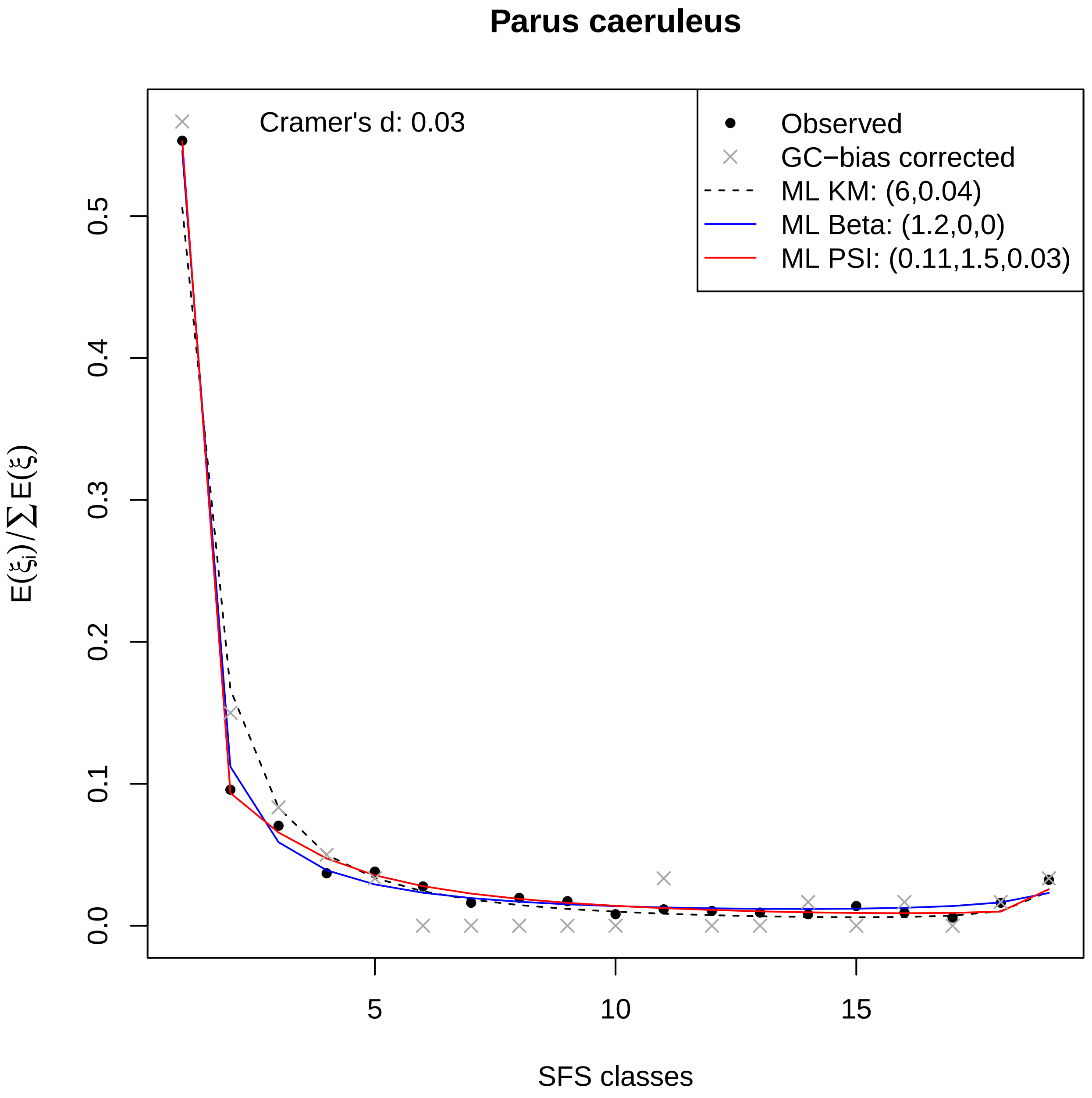

Parus caeruleus
Cramer's d: 0.03
Observed
GC−bias corrected
ML KM: (6,0.04)
ML Beta: (1.2,0,0)
ML PSI: (0.11,1.5,0.03)
0.0
0.1
0.2
0.3
0.4
0.5
5
10
15
SFS classes
$E(\xi_i)/\sum E(\xi)$

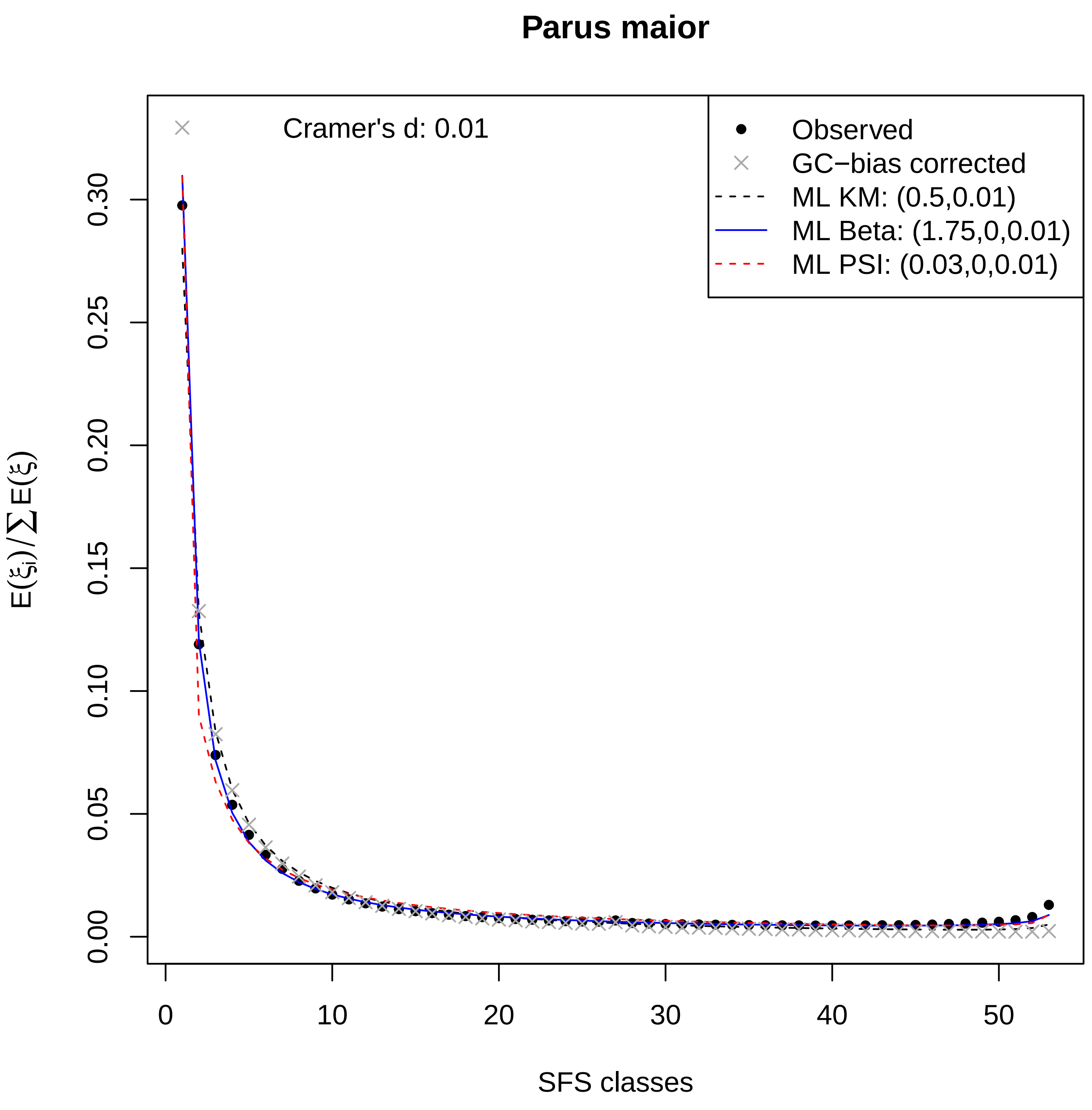

Parus maior
Cramer's d: 0.01
Observed
GC−bias corrected
ML KM: (0.5,0.01)
ML Beta: (1.75,0,0.01)
ML PSI: (0.03,0,0.01)
0.00
0.05
0.10
0.15
0.20
0.25
0.30
0
10
20
30
40
50
SFS classes
E(ξi)/∑E(ξ)

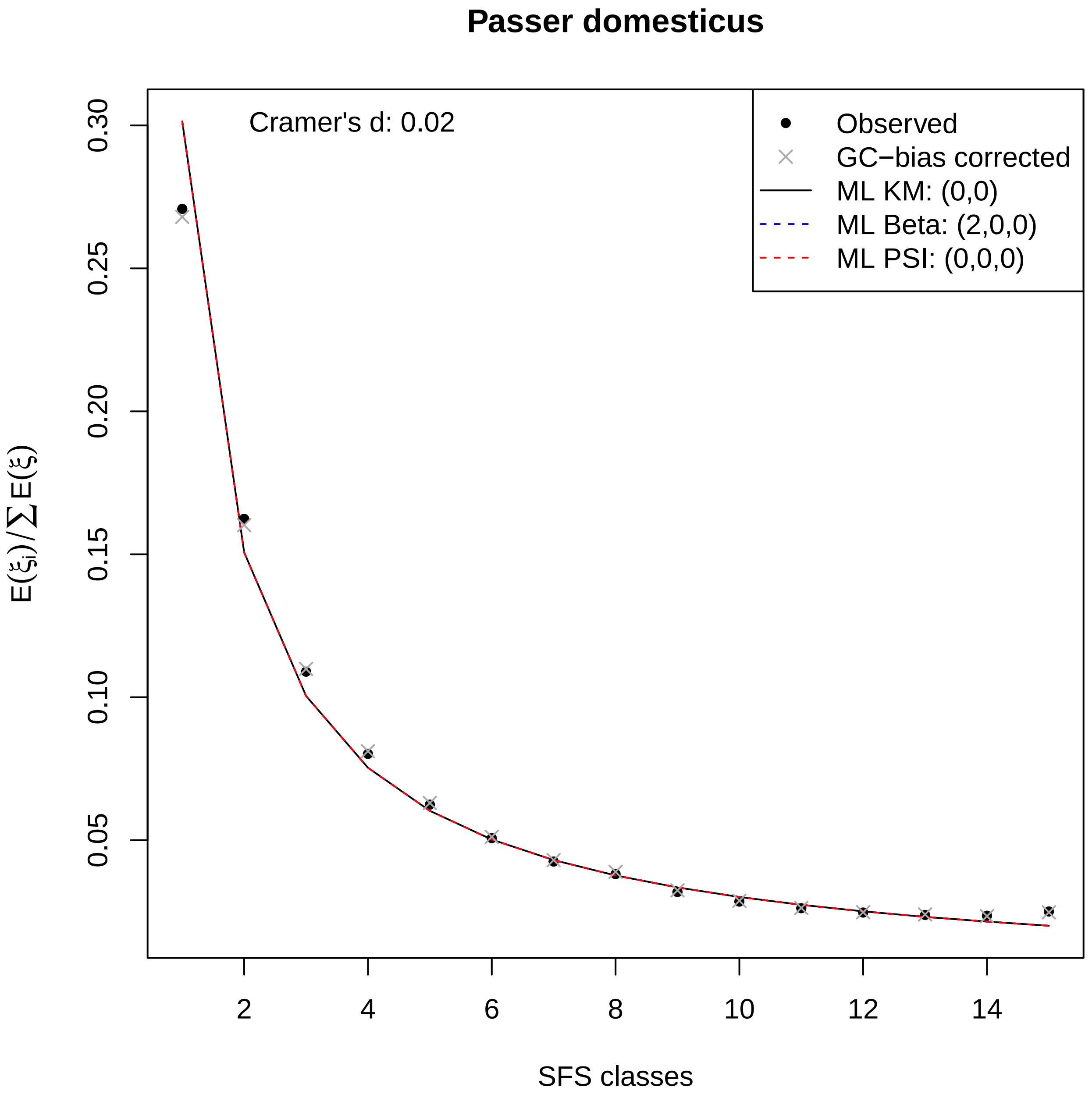

Passer domesticus
Cramer's d: 0.02
Observed
GC−bias corrected
ML KM: (0,0)
ML Beta: (2,0,0)
ML PSI: (0,0,0)
0.30
0.25
0.20
0.15
0.10
0.05
$E(\xi_i)/\sum E(\xi)$
2
4
6
8
10
12
14
SFS classes

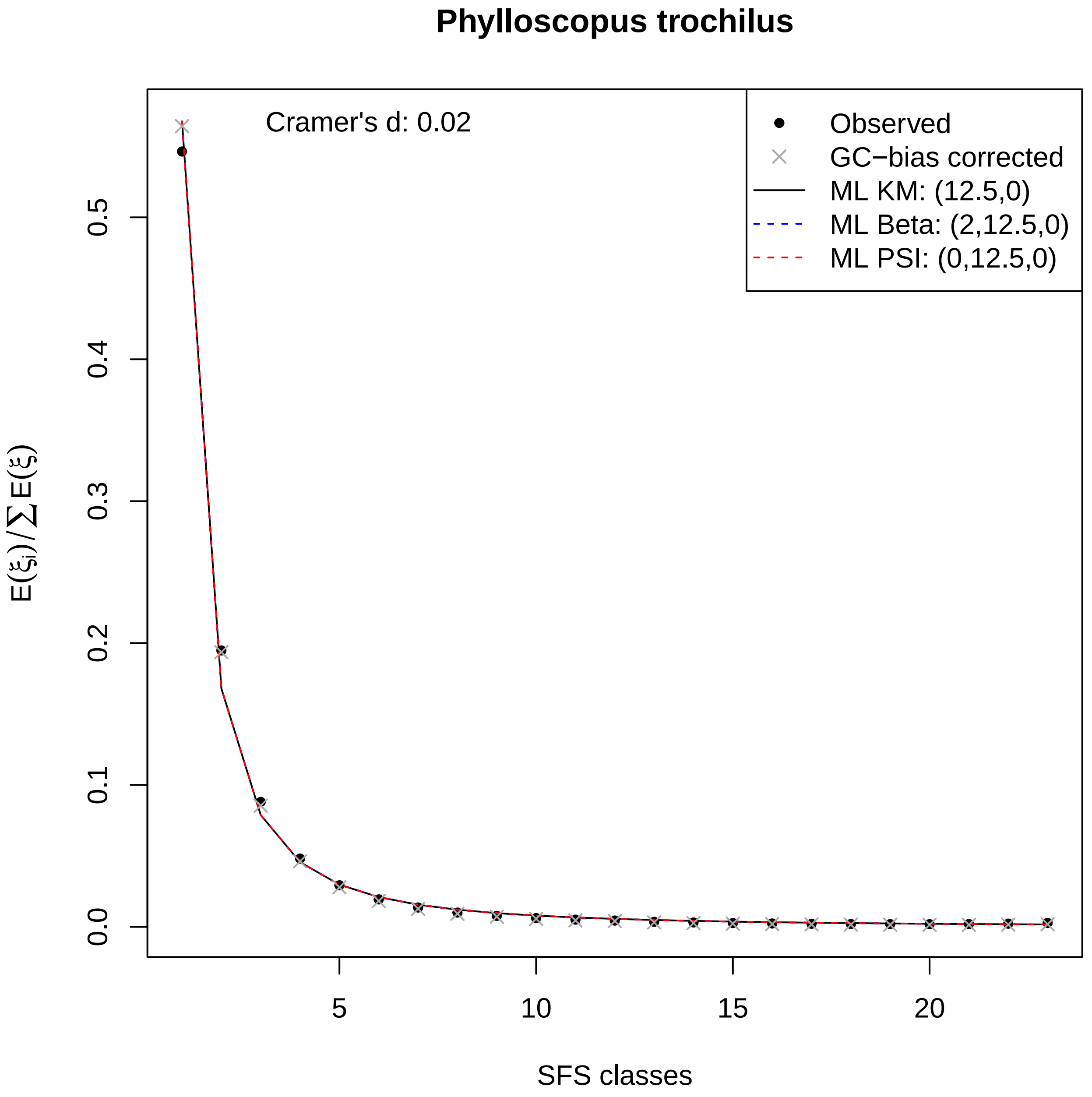

Phylloscopus trochilus
Cramer's d: 0.02
Observed
GC−bias corrected
ML KM: (12.5,0)
ML Beta: (2,12.5,0)
ML PSI: (0,12.5,0)
$E(\xi_i)/\sum E(\xi)$
0.0
0.1
0.2
0.3
0.4
0.5
5
10
15
20
SFS classes

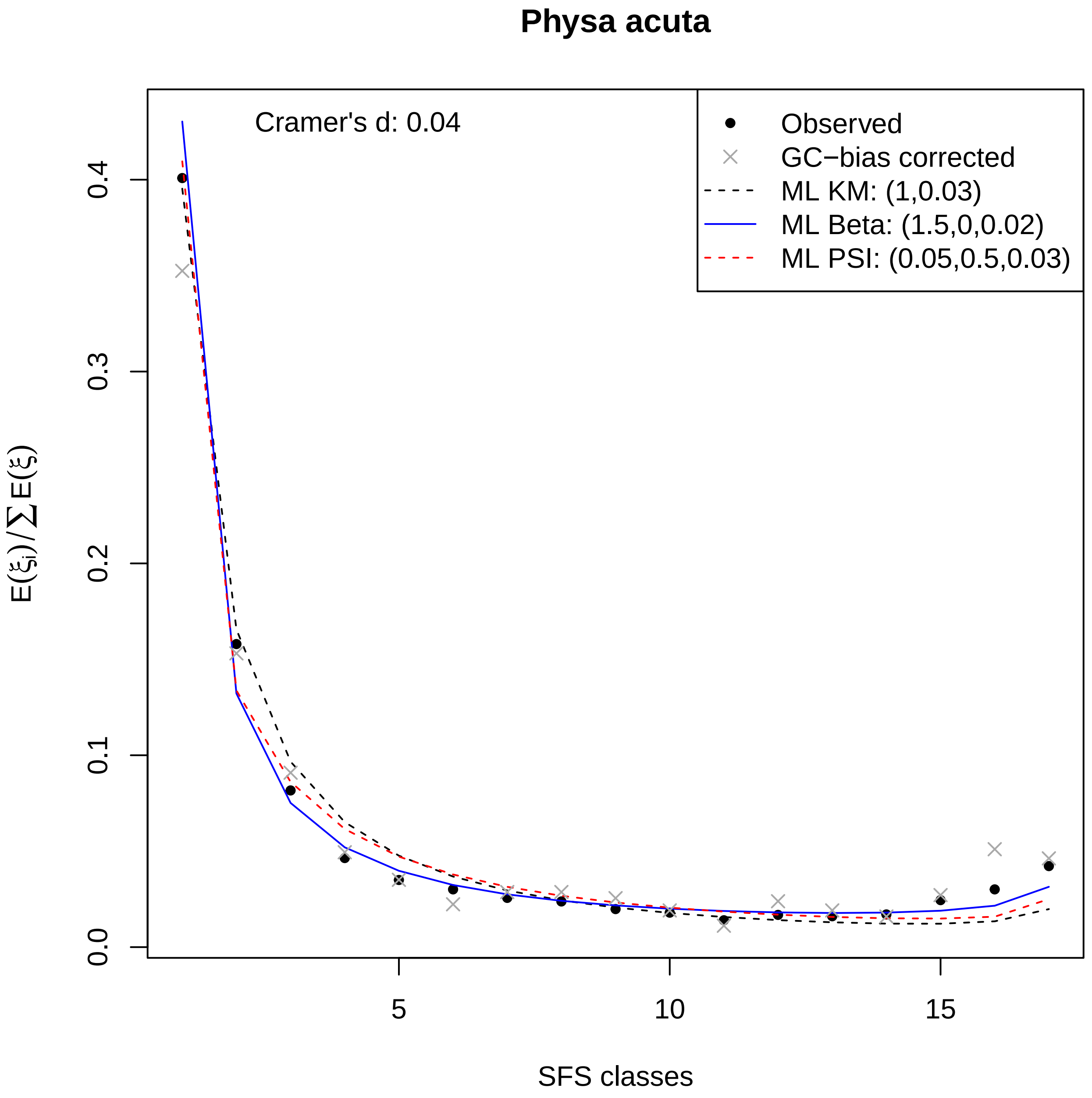
Physa acuta
Cramer's d: 0.04
Observed
GC−bias corrected
ML KM: (1,0.03)
ML Beta: (1.5,0,0.02)
ML PSI: (0.05,0.5,0.03)
$E(\xi_i)/\sum E(\xi)$
0.0
0.1
0.2
0.3
0.4
5
10
15
SFS classes

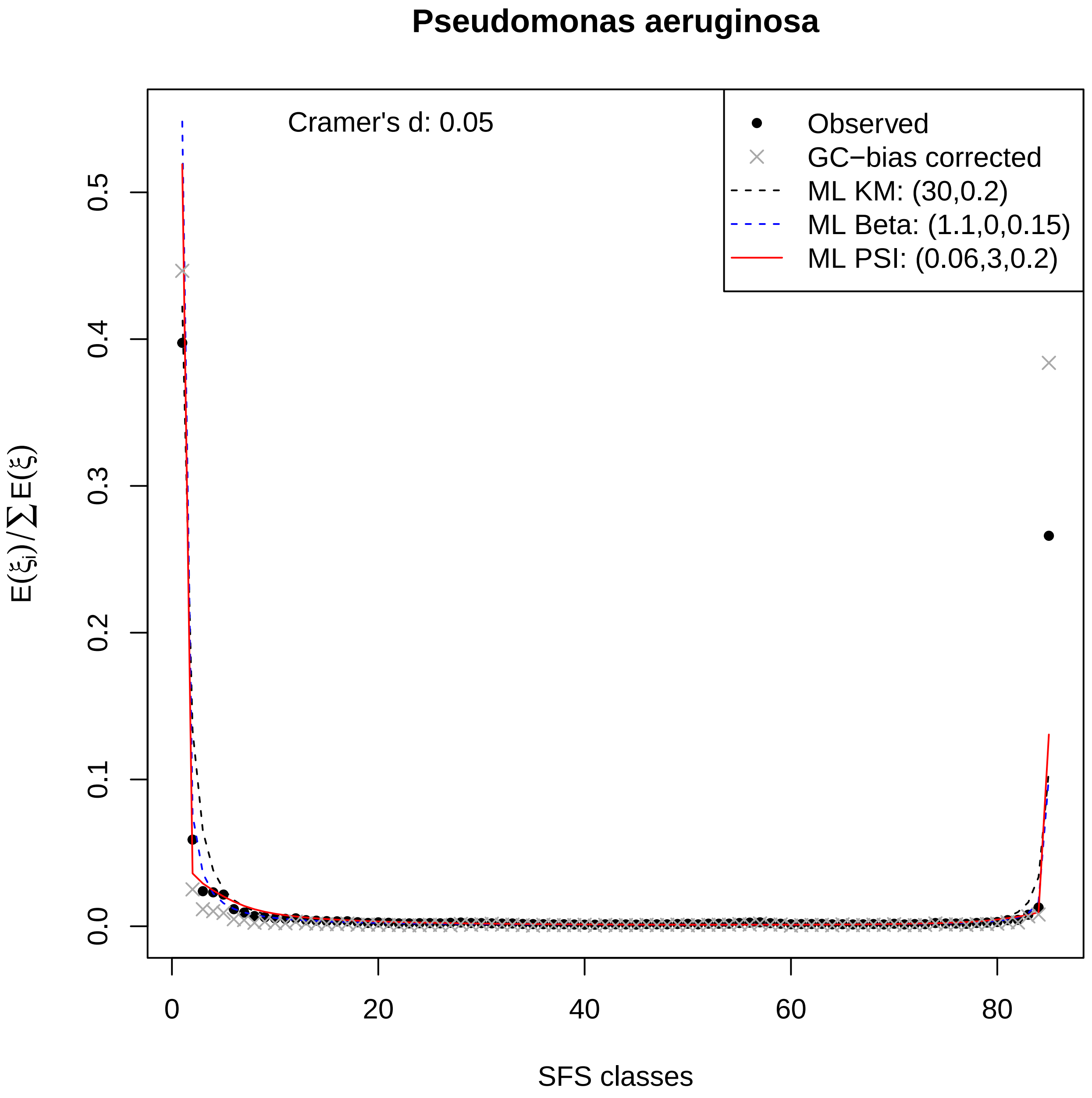
Pseudomonas aeruginosa
Cramer's d: 0.05
Observed
GC−bias corrected
ML KM: (30,0.2)
ML Beta: (1.1,0,0.15)
ML PSI: (0.06,3,0.2)
$E(\xi_i)/\sum E(\xi)$
SFS classes
0.0
0.1
0.2
0.3
0.4
0.5
0
20
40
60
80

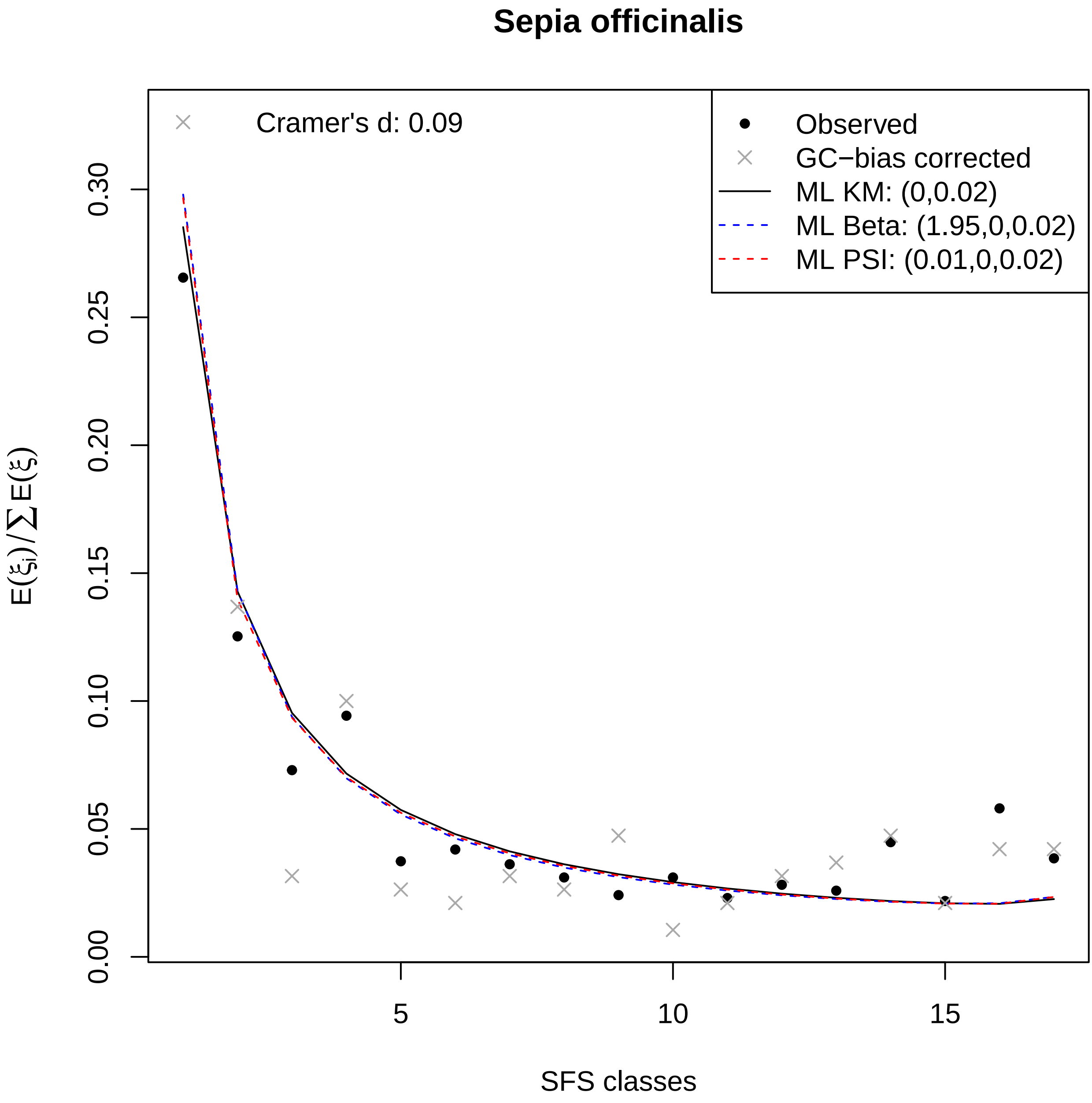

Sepia officinalis
Cramer's d: 0.09
Observed
GC−bias corrected
ML KM: (0,0.02)
ML Beta: (1.95,0,0.02)
ML PSI: (0.01,0,0.02)
0.00
0.05
0.10
0.15
0.20
0.25
0.30
$E(\xi_i)/\sum E(\xi)$
5
10
15
SFS classes

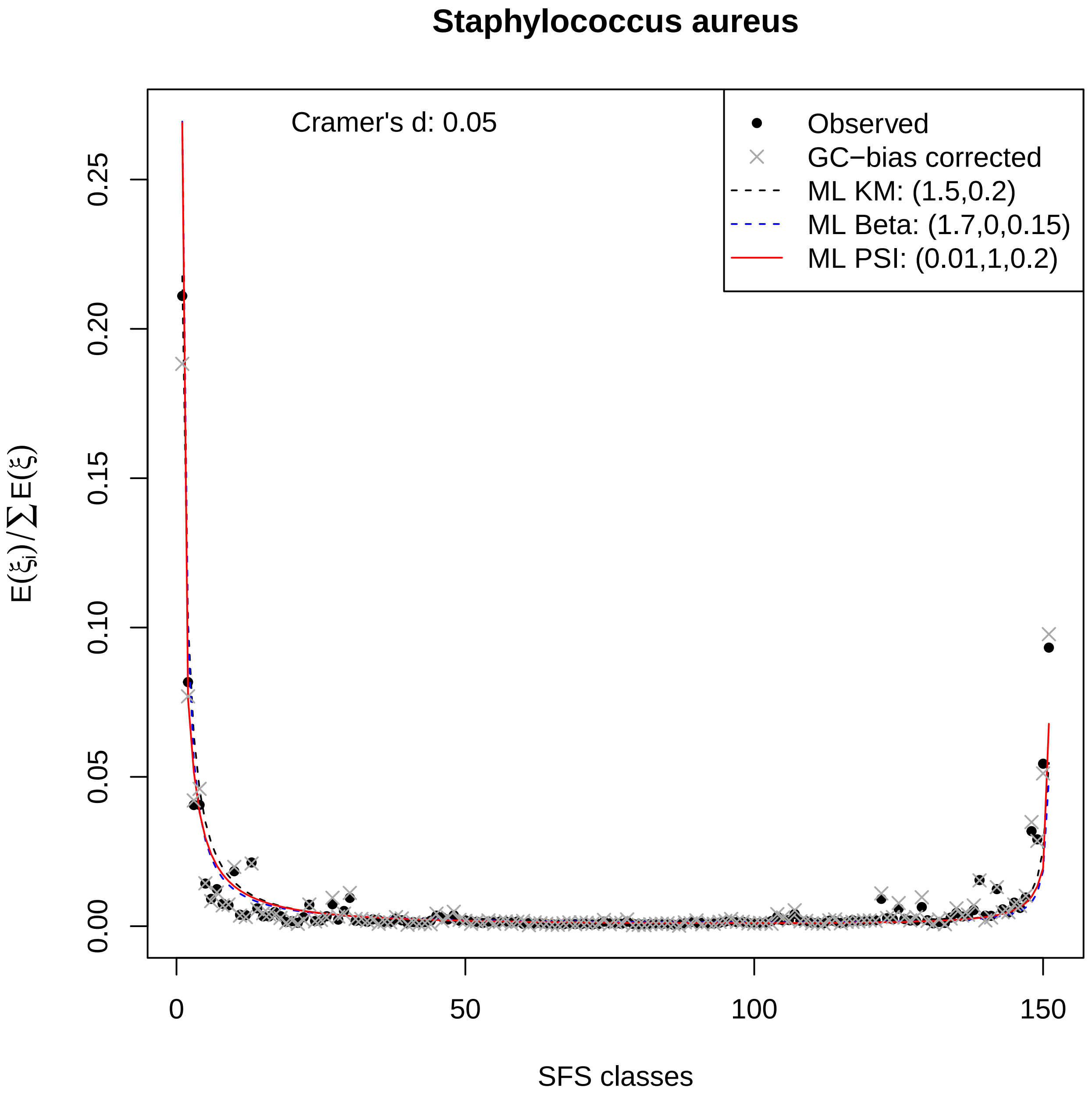
Staphylococcus aureus
Cramer's d: 0.05
Observed
GC−bias corrected
ML KM: (1.5,0.2)
ML Beta: (1.7,0,0.15)
ML PSI: (0.01,1,0.2)
$E(\xi_i)/\sum E(\xi)$
0.00
0.05
0.10
0.15
0.20
0.25
0
50
100
150
SFS classes

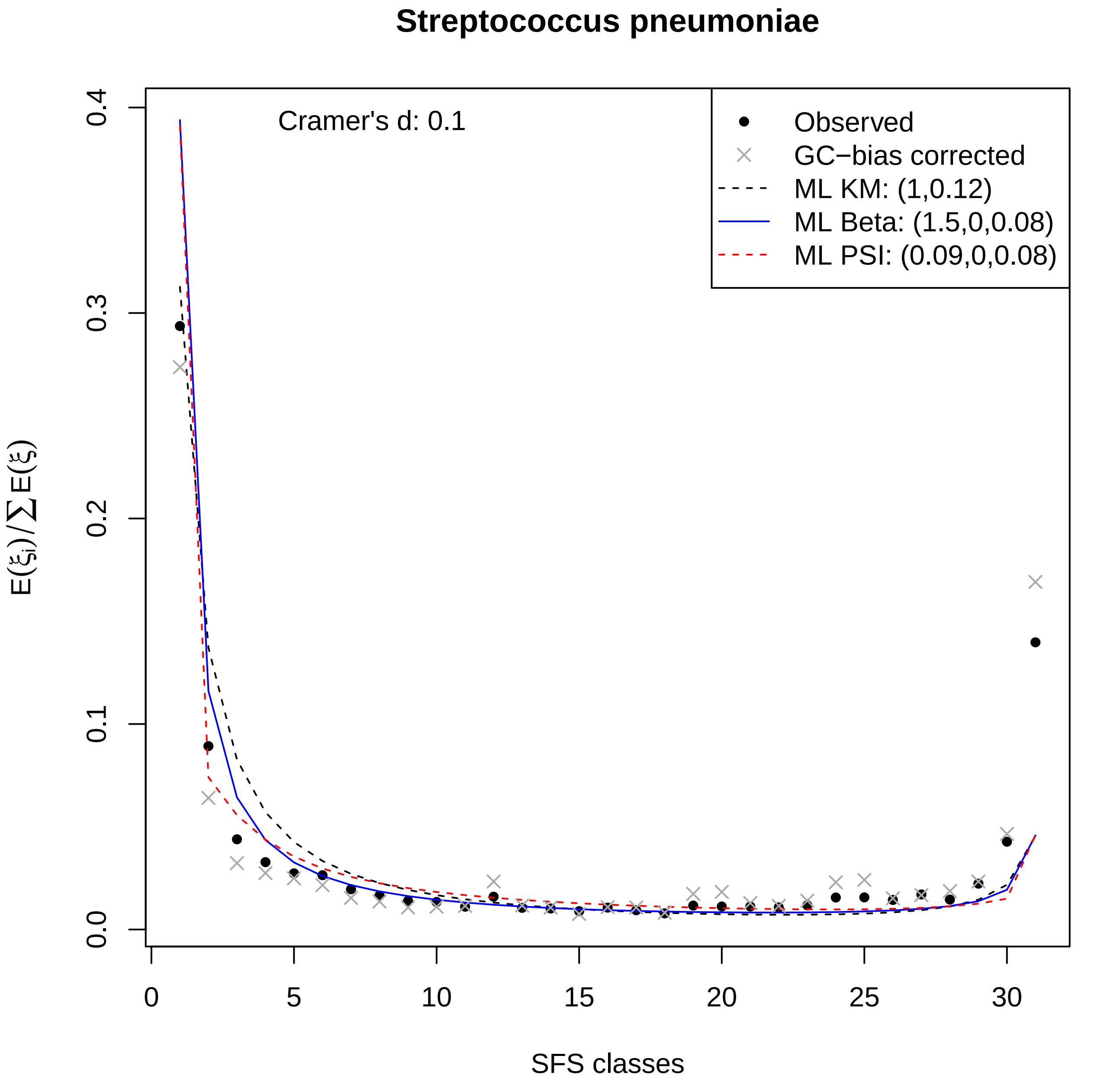

Streptococcus pneumoniae
Cramer's d: 0.1
Observed
GC−bias corrected
ML KM: (1,0.12)
ML Beta: (1.5,0,0.08)
ML PSI: (0.09,0,0.08)
$E(\xi_i)/\sum E(\xi)$
SFS classes
0.0
0.1
0.2
0.3
0.4
0
5
10
15
20
25
30

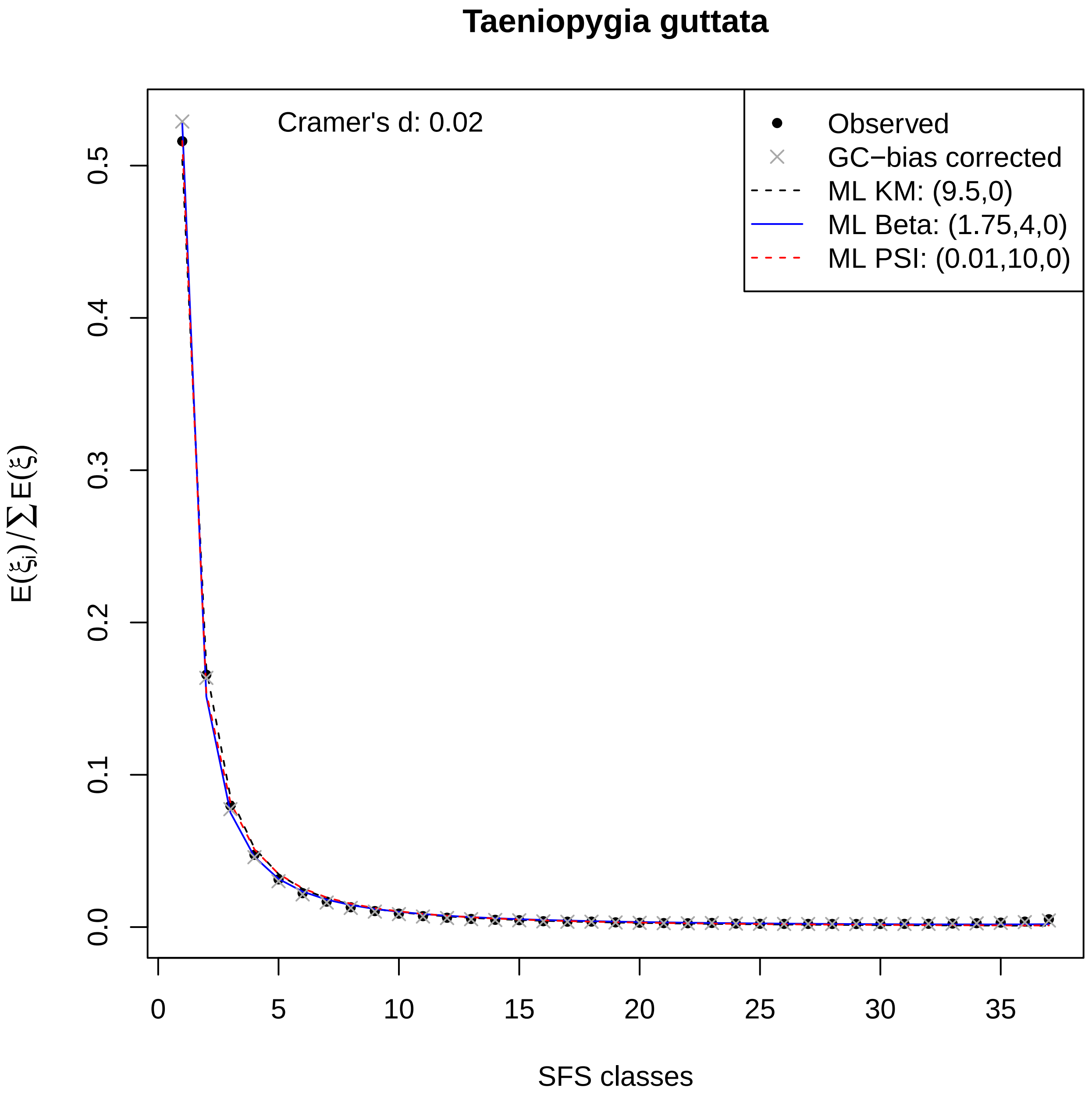

Taeniopygia guttata
Cramer's d: 0.02
Observed
GC−bias corrected
ML KM: (9.5,0)
ML Beta: (1.75,4,0)
ML PSI: (0.01,10,0)
$E(\xi_i)/\sum E(\xi)$
SFS classes
0.0
0.1
0.2
0.3
0.4
0.5
0
5
10
15
20
25
30
35

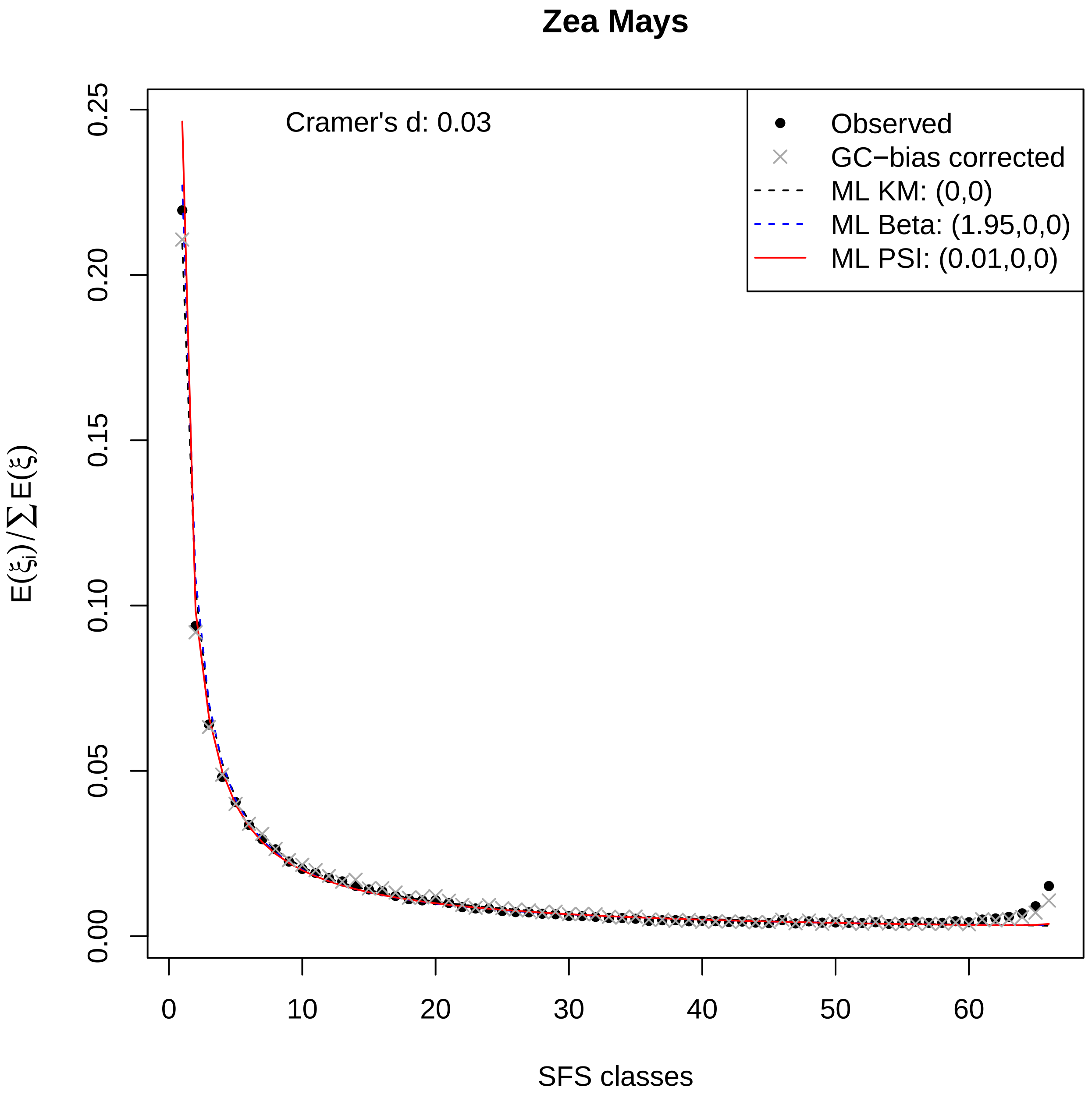
Zea Mays
Cramer's d: 0.03
Observed
GC−bias corrected
ML KM: (0,0)
ML Beta: (1.95,0,0)
ML PSI: (0.01,0,0)
$E(\xi_i)/\sum E(\xi)$
0.00
0.05
0.10
0.15
0.20
0.25
0
10
20
30
40
50
60
SFS classes
