# Supplementary material for: Interpreting the pervasive observation of U-shaped Site Frequency Spectra
Source: PLoS Genet. 2023 Mar 23;19(3):e1010677. doi: 10.1371/journal.pgen.1010677 (PMC10072462; doi:10.1371/journal.pgen.1010677)
Supplement: S2 Fig — (PDF) [file pgen.1010677.s003.pdf]

Supplementary Figures - (transformed) site frequency spectra vs. best fitting expectations

Supplementary material for
"Interpreting the pervasive observation of U-shaped Site Frequency Spectra"

Let The following plots show, for each data set analysed,

- $\left(\frac{i\cdot\xi_i}{\sum_{j=1}^{n-1}\xi_j}\right)_{i\in\{1,\dots,n-1\}} = \left(\frac{1\cdot\xi_i}{\sum_{j=1}^{n-1}\xi_j},\dots,\frac{(n-1)\cdot\xi_i}{\sum_{j=1}^{n-1}\xi_j}\right)$ the observed site frequency spectrum (SFS) $\xi_1,\dots,\xi_{i-1}$ multiplied by $1,\dots,n-1$ (so ith entry multiplied by i) and then scaled by the total number of segregating sites
- the non-GC-biased proportion of the observed SFS (again with its ith entry multiplied by i), scaled by its total number of mutations
- the expected SFS, transformed by multiplying the ith entry by i, under the best fitting parameters
  - among all Kingman-coalescent-based model choices
  - among all Beta-coalescent-based model choices
  - among all Psi-coalescent-based model choices

  scaled by the total expected number of mutations with these best fitting parameters

Solid lines in the plot show the best fitting model(s) across the three types of coalescent models (Kingman, Beta, Psi), while dotted lines indicate inferior fit (as derived by the testing procedure using Eq. 3 in the main manuscript).

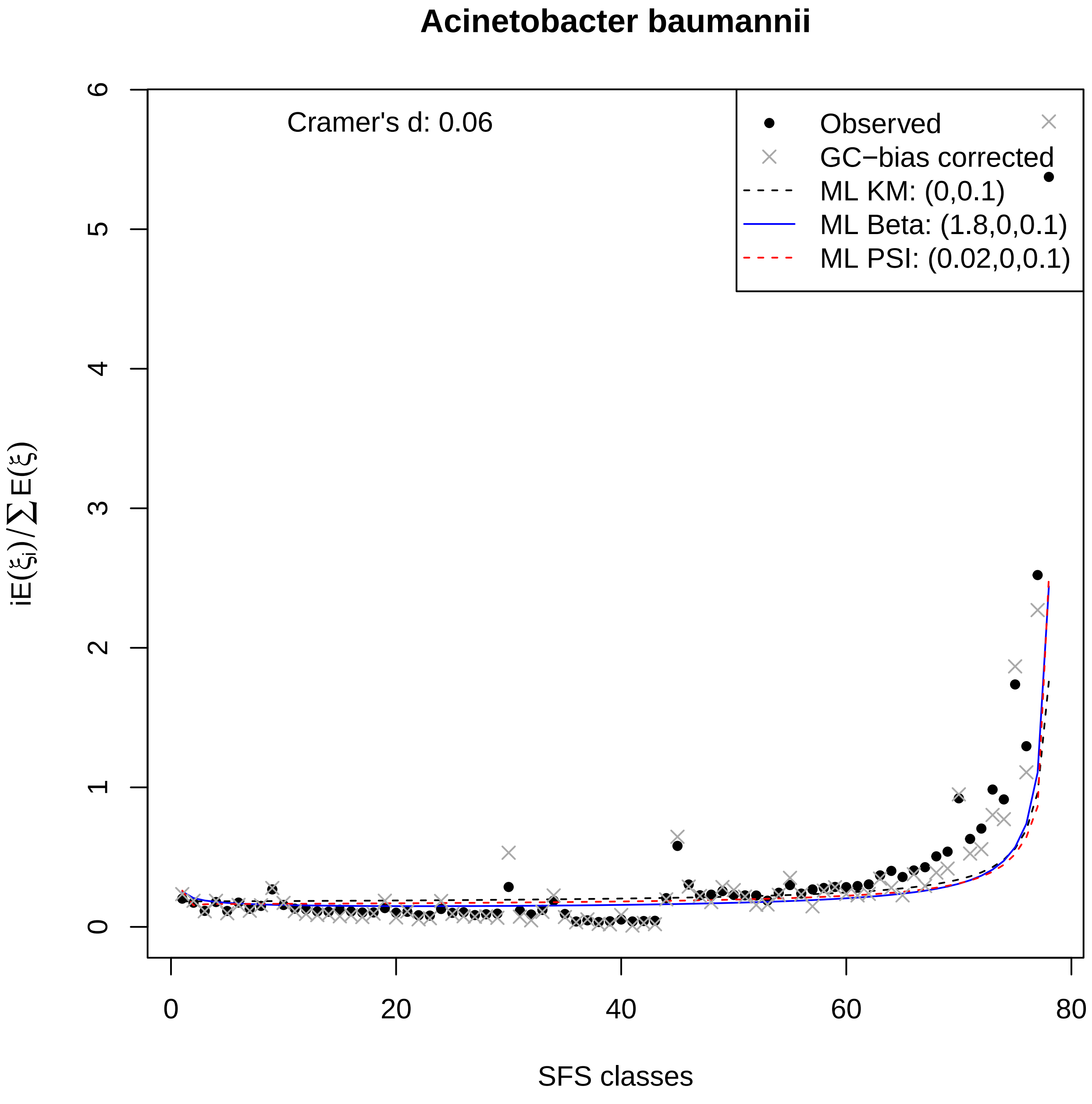
Acinetobacter baumannii
Cramer's d: 0.06
Observed
GC−bias corrected
ML KM: (0,0.1)
ML Beta: (1.8,0,0.1)
ML PSI: (0.02,0,0.1)
0
1
2
3
4
5
6
0
20
40
60
80
SFS classes
iE(ξi)/∑E(ξ)

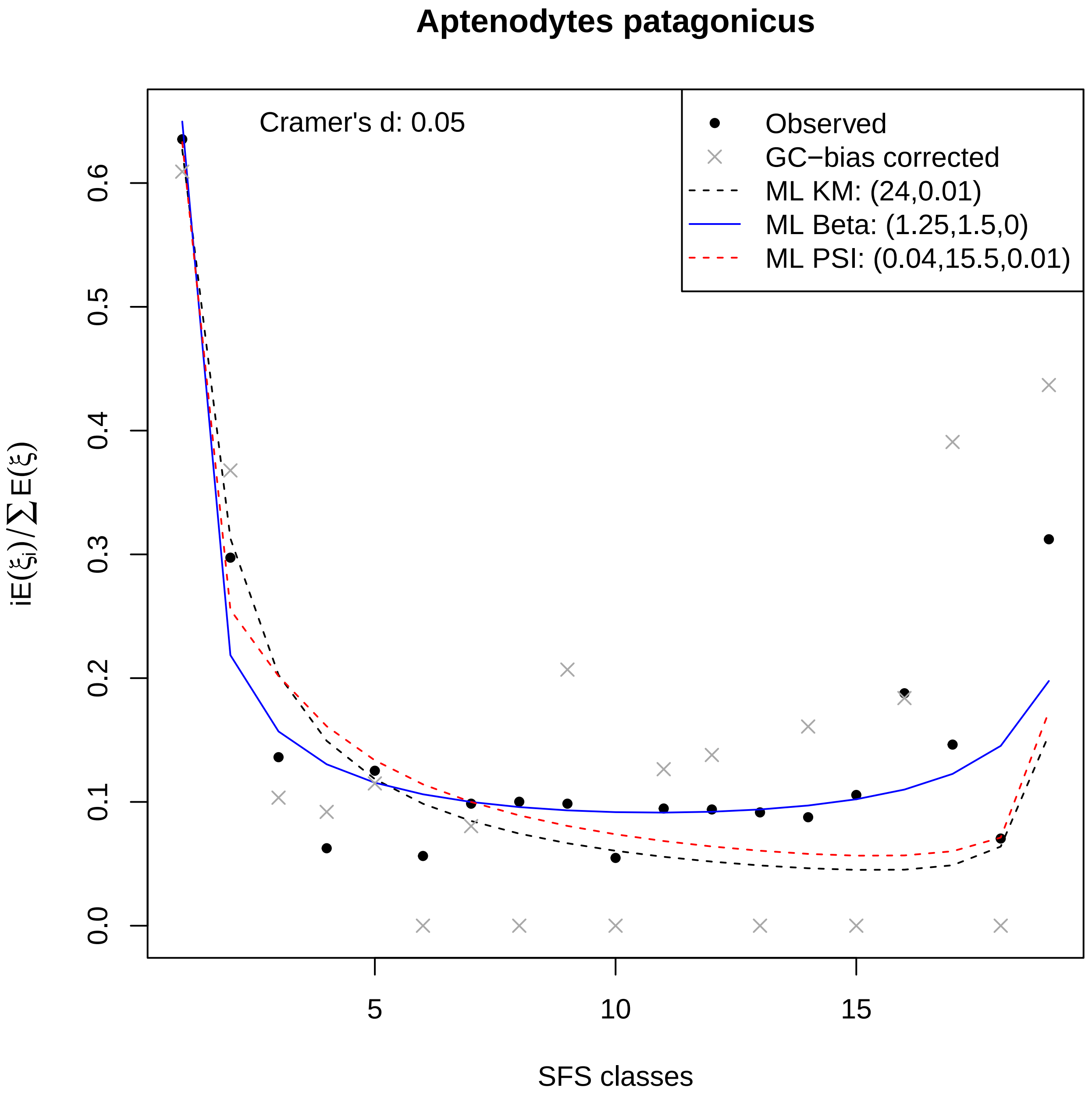

Aptenodytes patagonicus
Cramer's d: 0.05
Observed
GC−bias corrected
ML KM: (24,0.01)
ML Beta: (1.25,1.5,0)
ML PSI: (0.04,15.5,0.01)
$iE(\xi_i)/\sum E(\xi)$
0.0
0.1
0.2
0.3
0.4
0.5
0.6
5
10
15
SFS classes

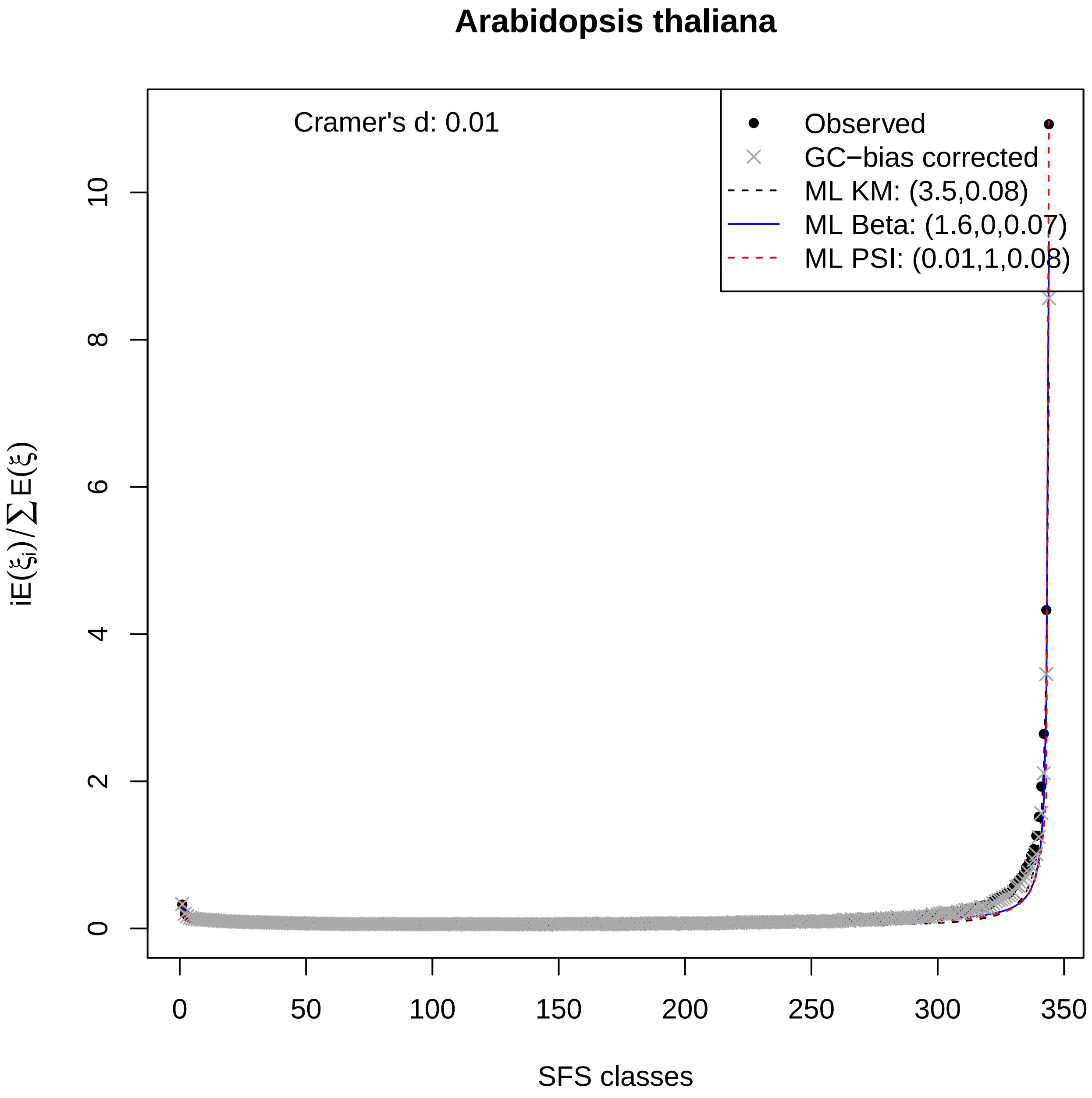

Arabidopsis thaliana
Cramer's d: 0.01
Observed
GC−bias corrected
ML KM: (3.5,0.08)
ML Beta: (1.6,0,0.07)
ML PSI: (0.01,1,0.08)
0
2
4
6
8
10
0
50
100
150
200
250
300
350
SFS classes
$iE(\xi_i)/\sum E(\xi)$

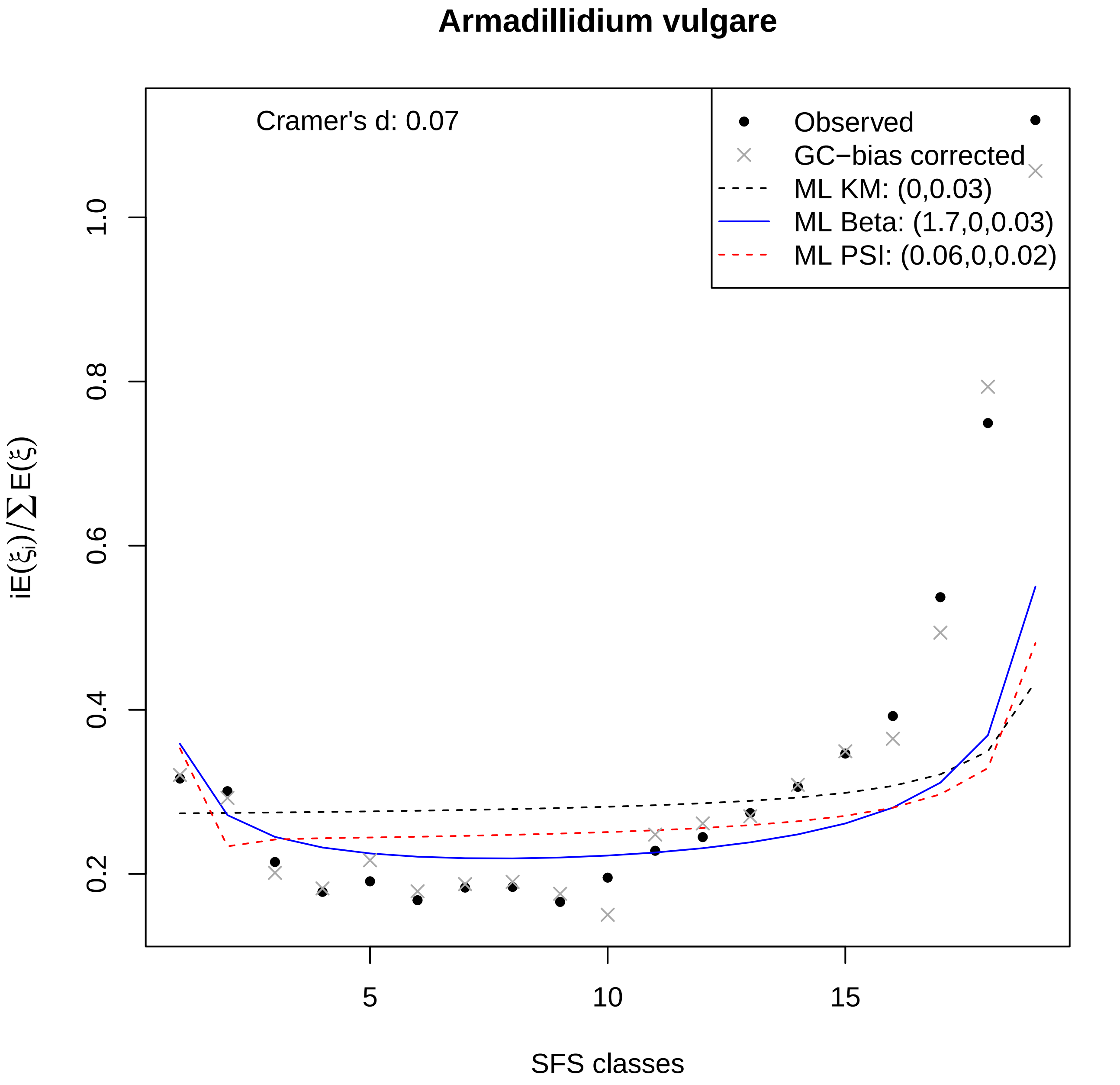

Armadillidium vulgare
Cramer's d: 0.07
Observed
GC−bias corrected
ML KM: (0,0.03)
ML Beta: (1.7,0,0.03)
ML PSI: (0.06,0,0.02)
$iE(\xi_i)/\sum E(\xi)$
0.2
0.4
0.6
0.8
1.0
5
10
15
SFS classes

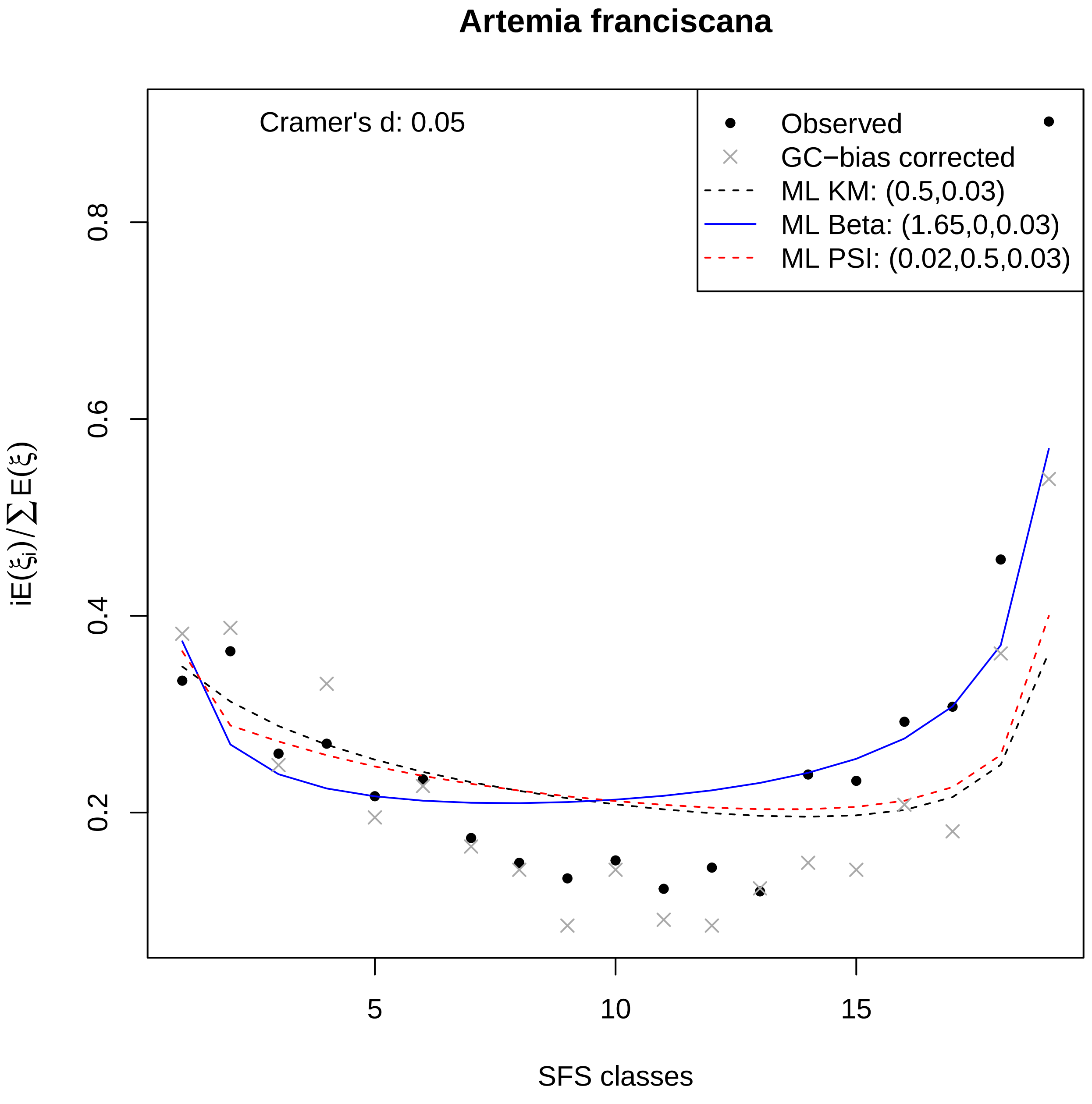

Artemia franciscana
Cramer's d: 0.05
Observed
GC−bias corrected
ML KM: (0.5,0.03)
ML Beta: (1.65,0,0.03)
ML PSI: (0.02,0.5,0.03)
0.2
0.4
0.6
0.8
5
10
15
SFS classes
iE(ξi)/∑E(ξ)

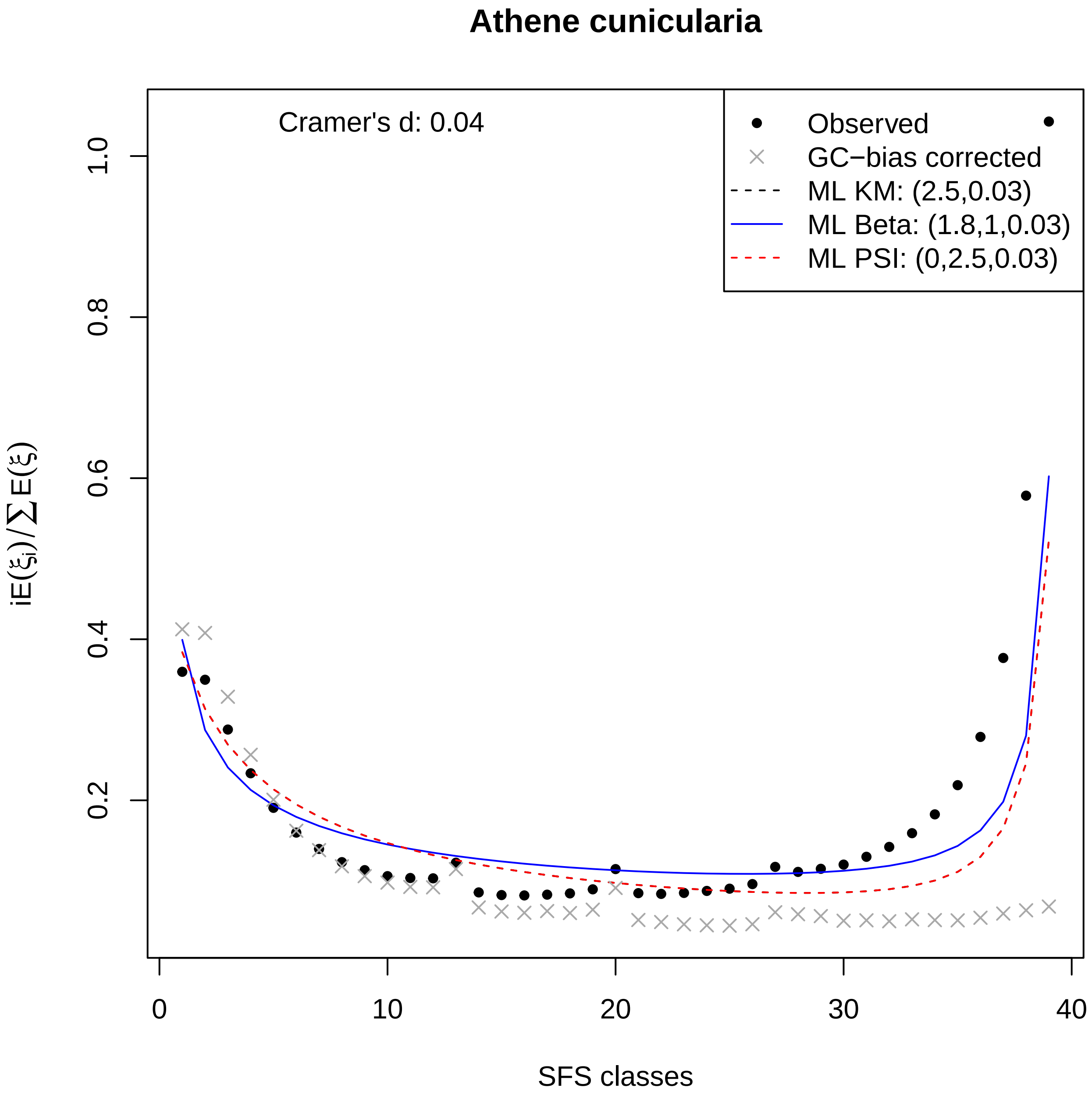
Athene cunicularia
Cramer's d: 0.04
Observed
GC−bias corrected
ML KM: (2.5,0.03)
ML Beta: (1.8,1,0.03)
ML PSI: (0,2.5,0.03)
iE(ξi)/∑E(ξ)
SFS classes
0.2
0.4
0.6
0.8
1.0
0
10
20
30
40

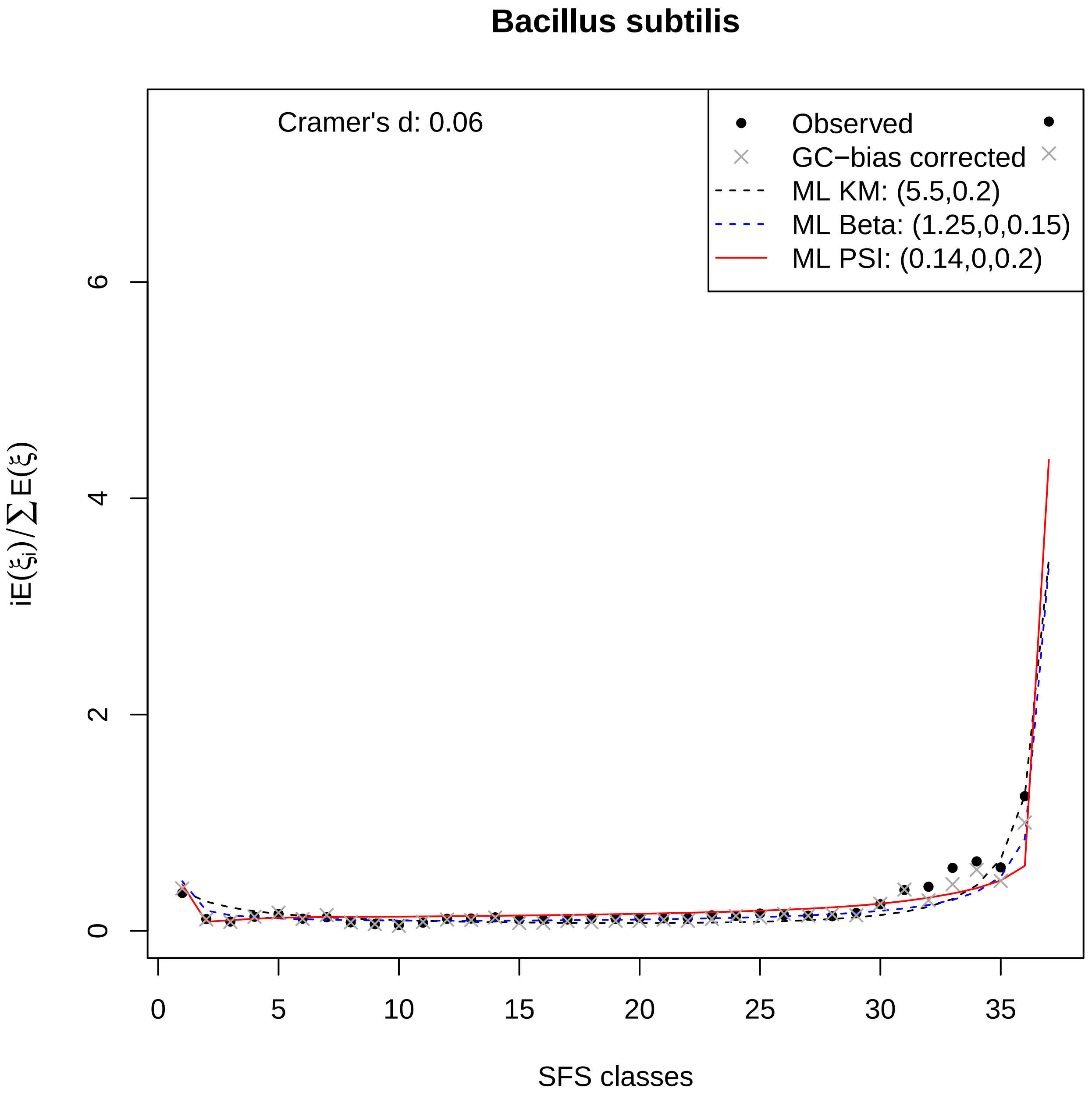

Bacillus subtilis
Cramer's d: 0.06
Observed
GC−bias corrected
ML KM: (5.5,0.2)
ML Beta: (1.25,0,0.15)
ML PSI: (0.14,0,0.2)
0
2
4
6
0
5
10
15
20
25
30
35
SFS classes
$iE(\xi_i)/\sum E(\xi)$

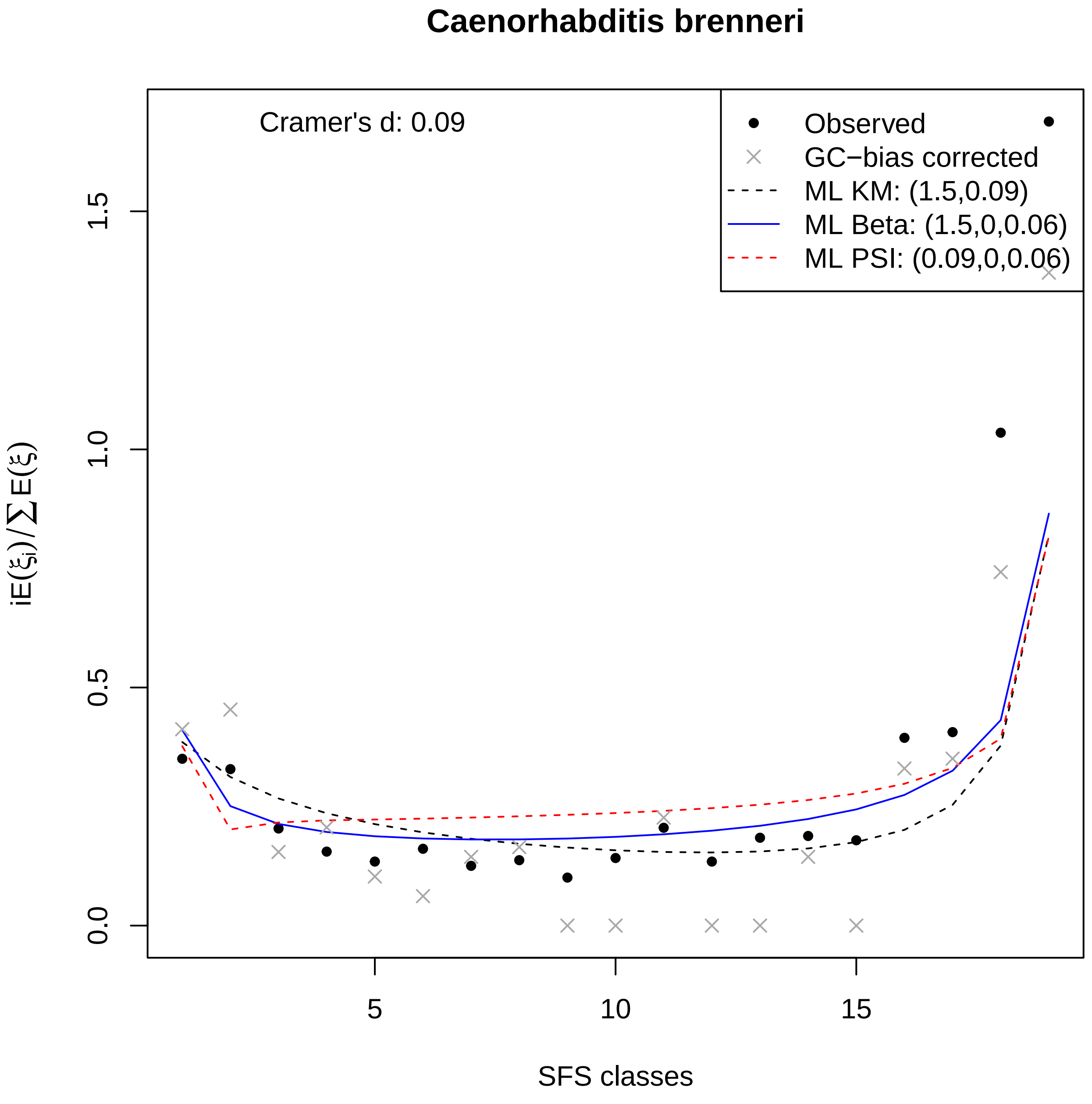
Caenorhabditis brenneri
Cramer's d: 0.09
Observed
GC−bias corrected
ML KM: (1.5,0.09)
ML Beta: (1.5,0,0.06)
ML PSI: (0.09,0,0.06)
$iE(\xi_i)/\sum E(\xi)$
0.0
0.5
1.0
1.5
5
10
15
SFS classes

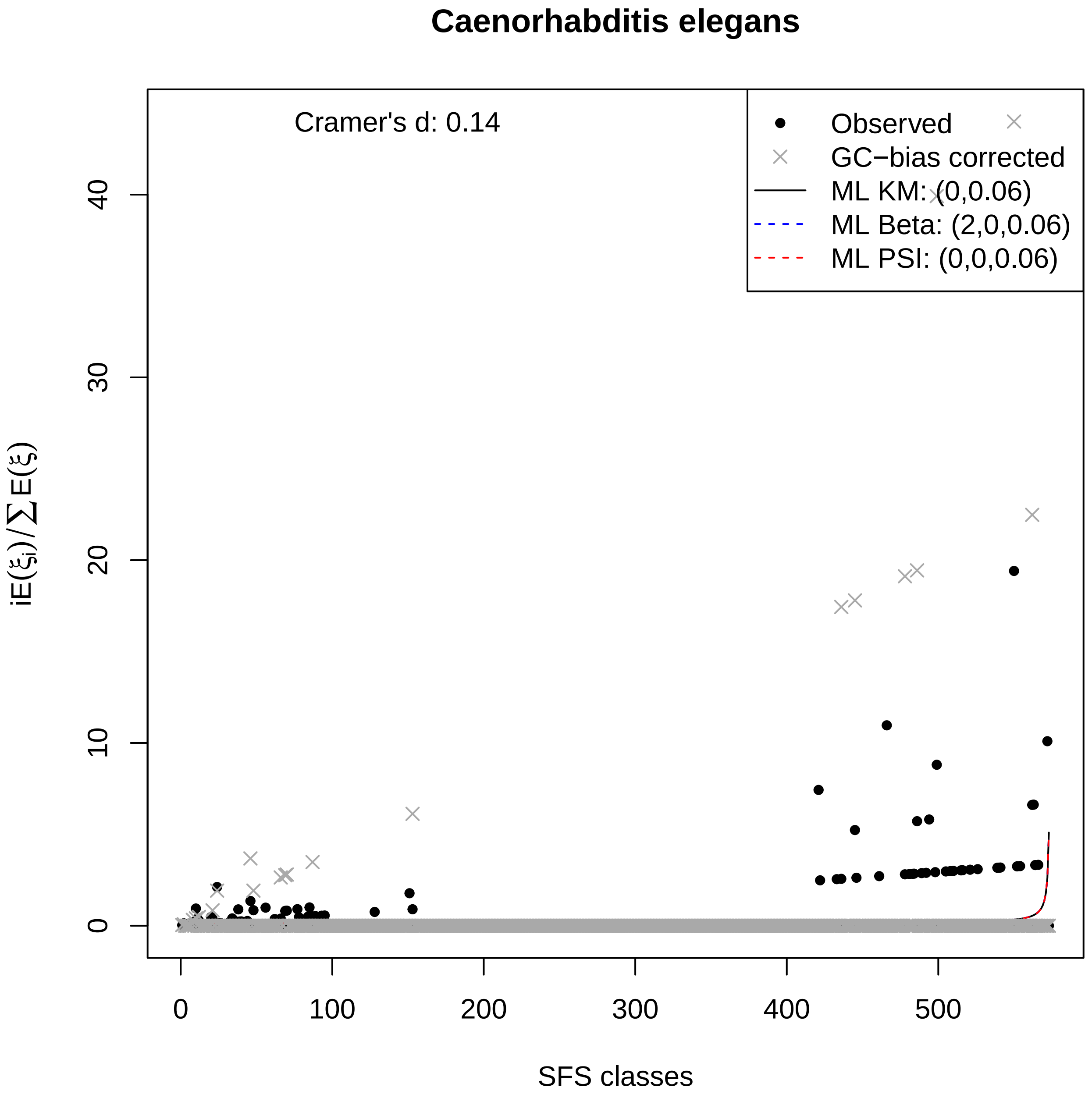
Caenorhabditis elegans
Cramer's d: 0.14
Observed
GC−bias corrected
ML KM: (0,0.06)
ML Beta: (2,0,0.06)
ML PSI: (0,0,0.06)
iE(ξi)/∑E(ξ)
SFS classes
0
10
20
30
40
0
100
200
300
400
500

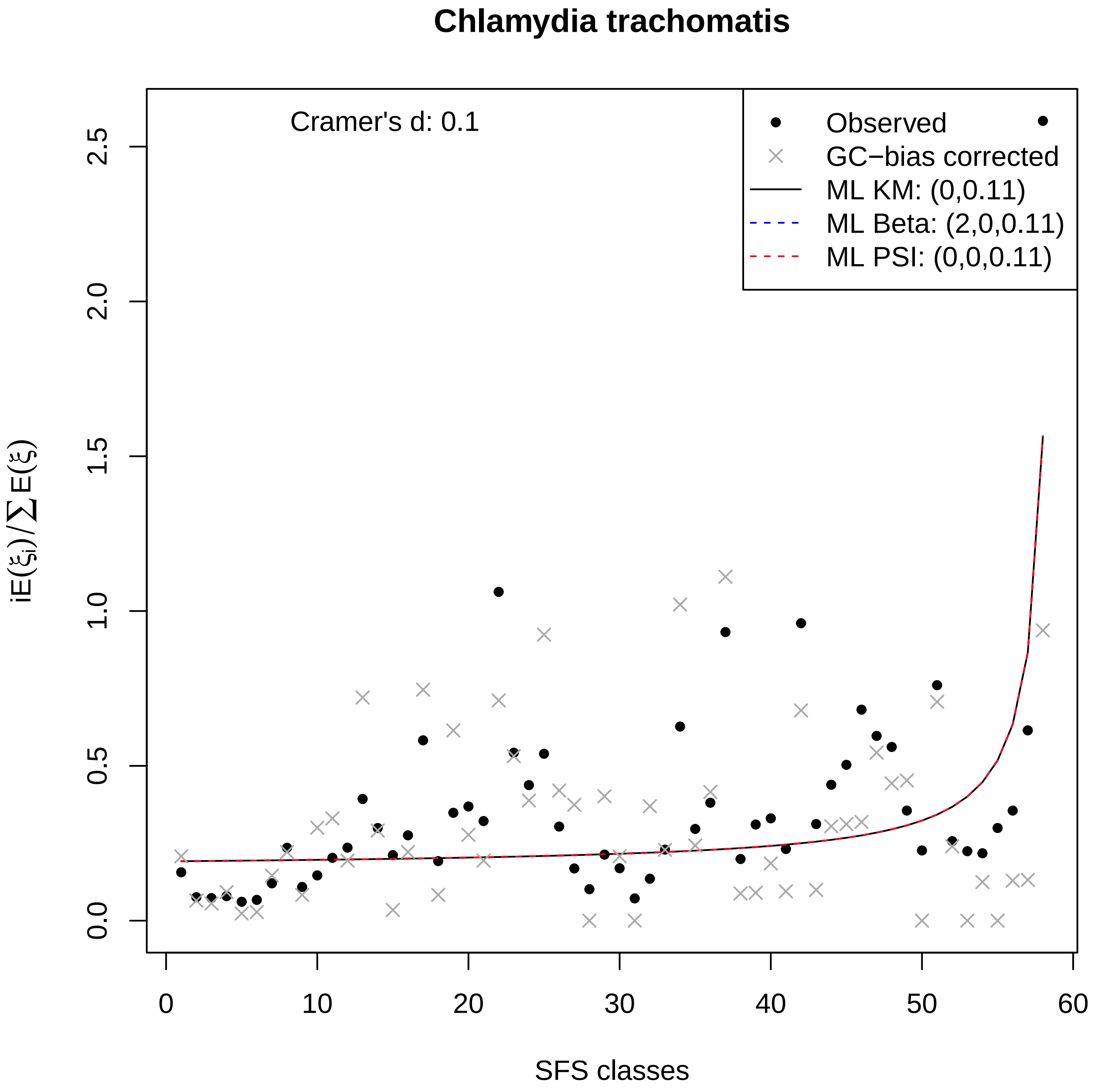
Chlamydia trachomatis
Cramer's d: 0.1
Observed
GC−bias corrected
ML KM: (0,0.11)
ML Beta: (2,0,0.11)
ML PSI: (0,0,0.11)
$iE(\xi_i)/\sum E(\xi)$
SFS classes
0.0
0.5
1.0
1.5
2.0
2.5
0
10
20
30
40
50
60

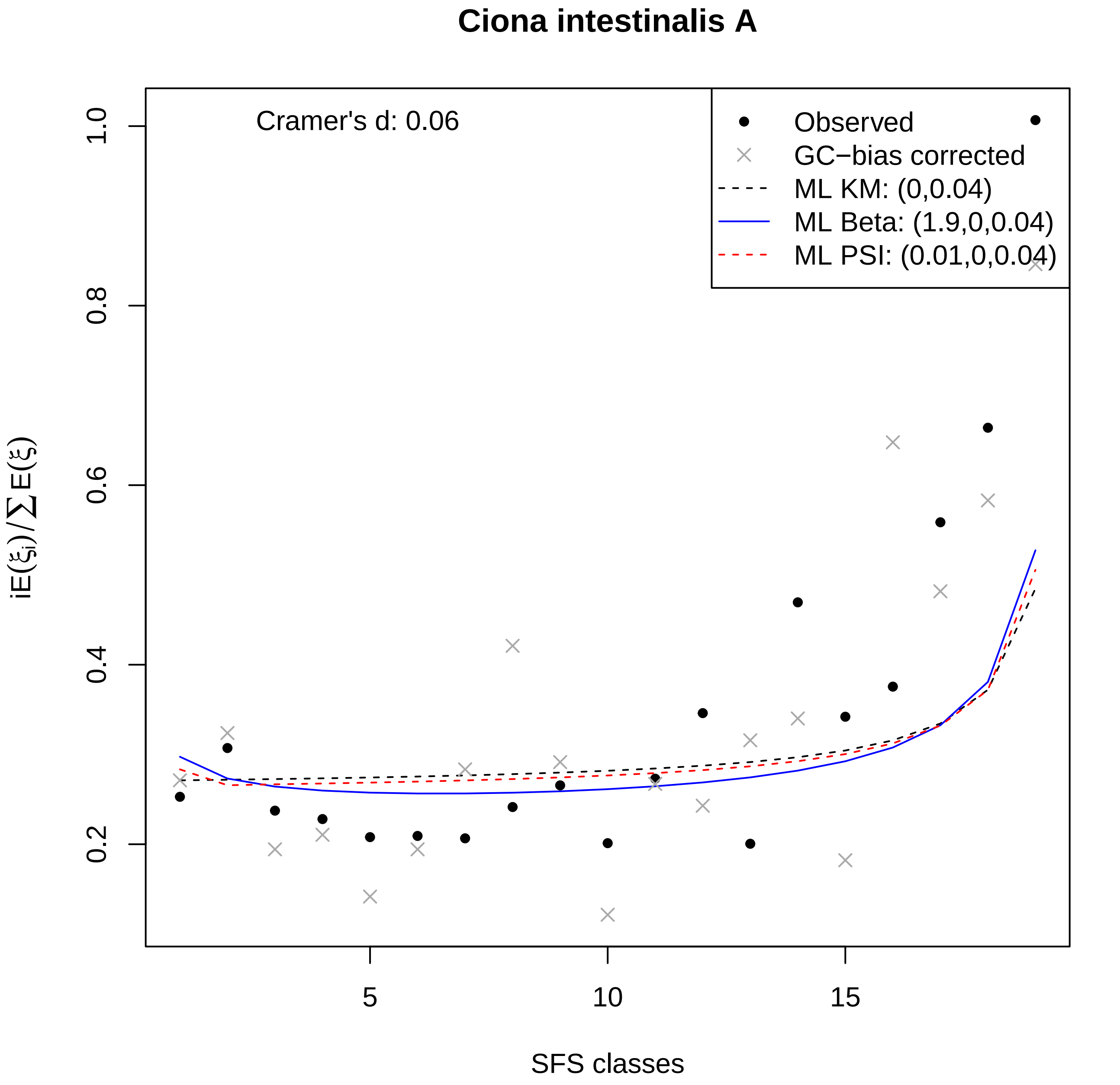

Ciona intestinalis A
Cramer's d: 0.06
Observed
GC−bias corrected
ML KM: (0,0.04)
ML Beta: (1.9,0,0.04)
ML PSI: (0.01,0,0.04)
$iE(\xi_i)/\sum E(\xi)$
SFS classes
0.2
0.4
0.6
0.8
1.0
5
10
15

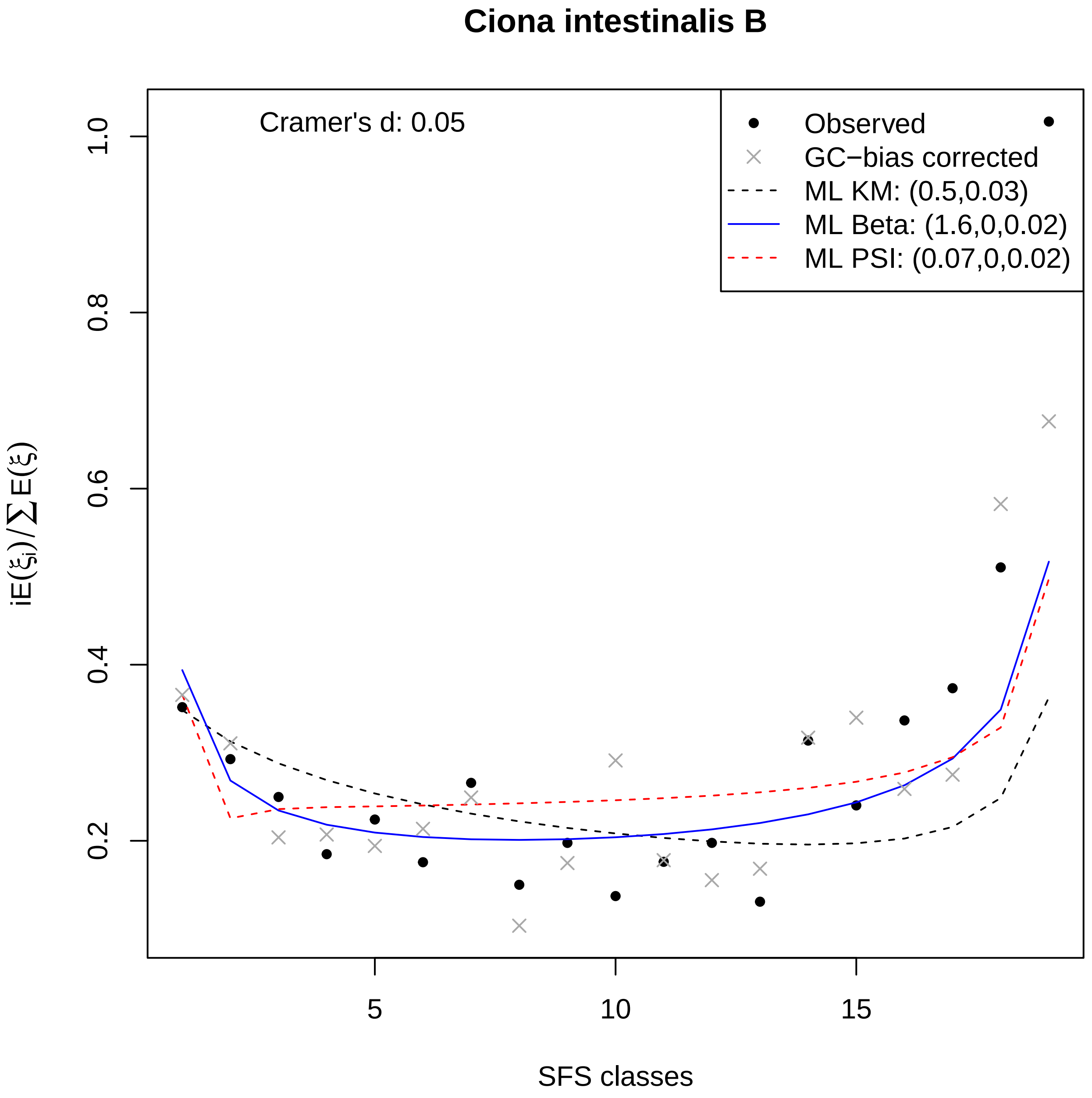
Ciona intestinalis B
Cramer's d: 0.05
Observed
GC−bias corrected
ML KM: (0.5,0.03)
ML Beta: (1.6,0,0.02)
ML PSI: (0.07,0,0.02)
$iE(\xi_i)/\sum E(\xi)$
SFS classes
0.2
0.4
0.6
0.8
1.0
5
10
15

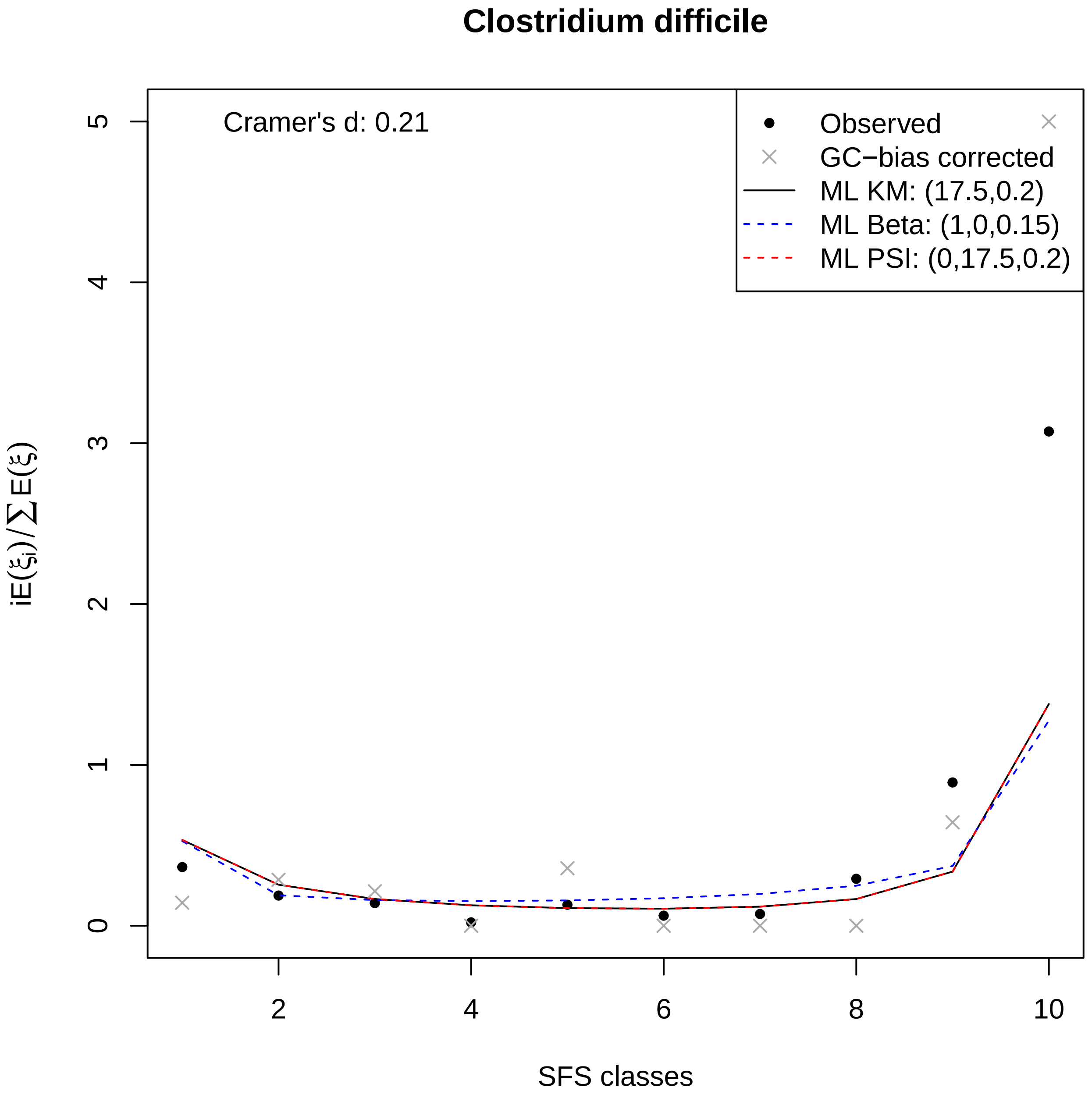

Clostridium difficile
Cramer's d: 0.21
Observed
GC−bias corrected
ML KM: (17.5,0.2)
ML Beta: (1,0,0.15)
ML PSI: (0,17.5,0.2)
$iE(\xi_i)/\sum E(\xi)$
SFS classes
0
1
2
3
4
5
2
4
6
8
10

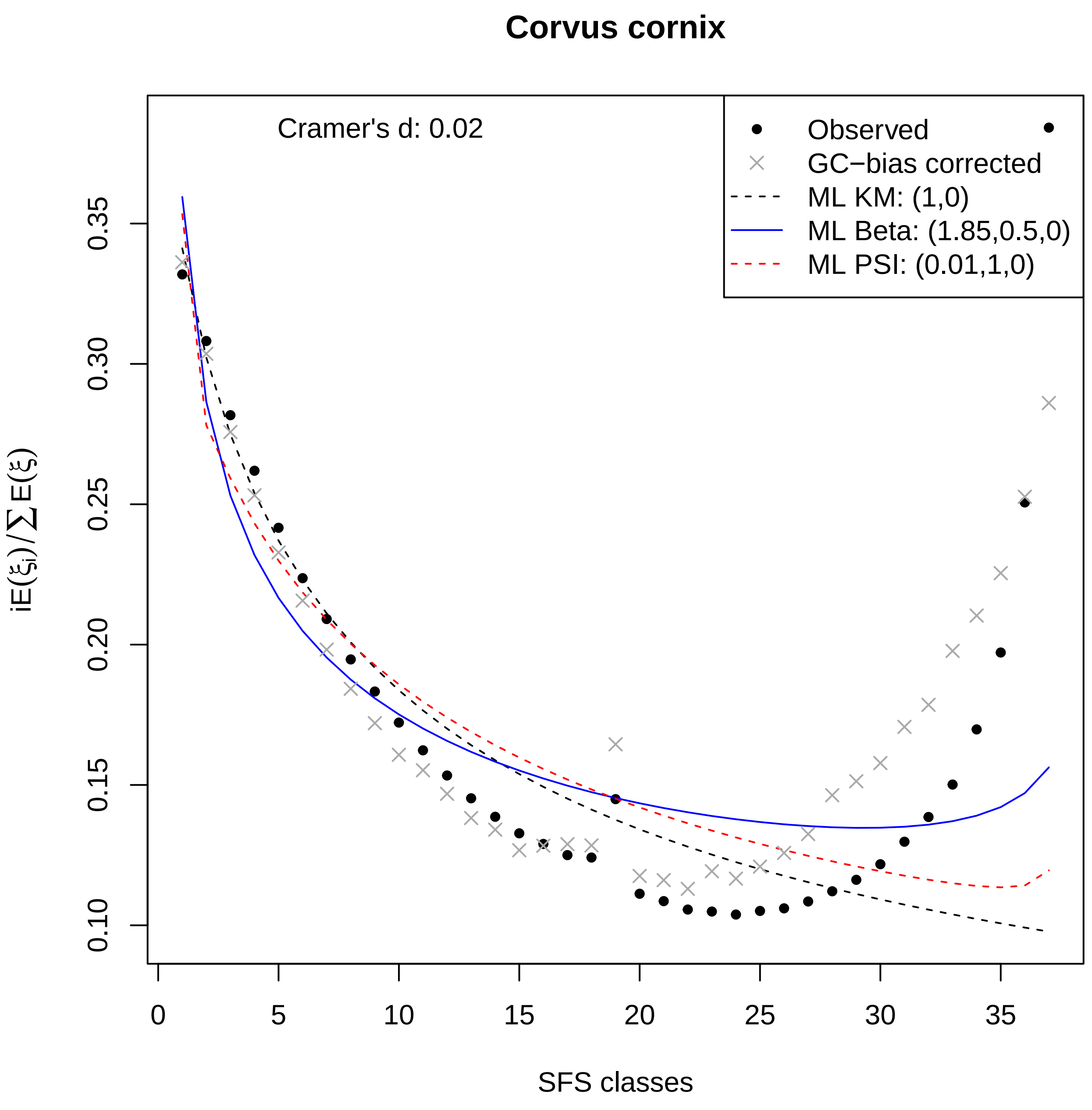
Corvus cornix
Cramer's d: 0.02
Observed
GC−bias corrected
ML KM: (1,0)
ML Beta: (1.85,0.5,0)
ML PSI: (0.01,1,0)
$iE(\xi_i)/\sum E(\xi)$
SFS classes
0.10
0.15
0.20
0.25
0.30
0.35
0
5
10
15
20
25
30
35

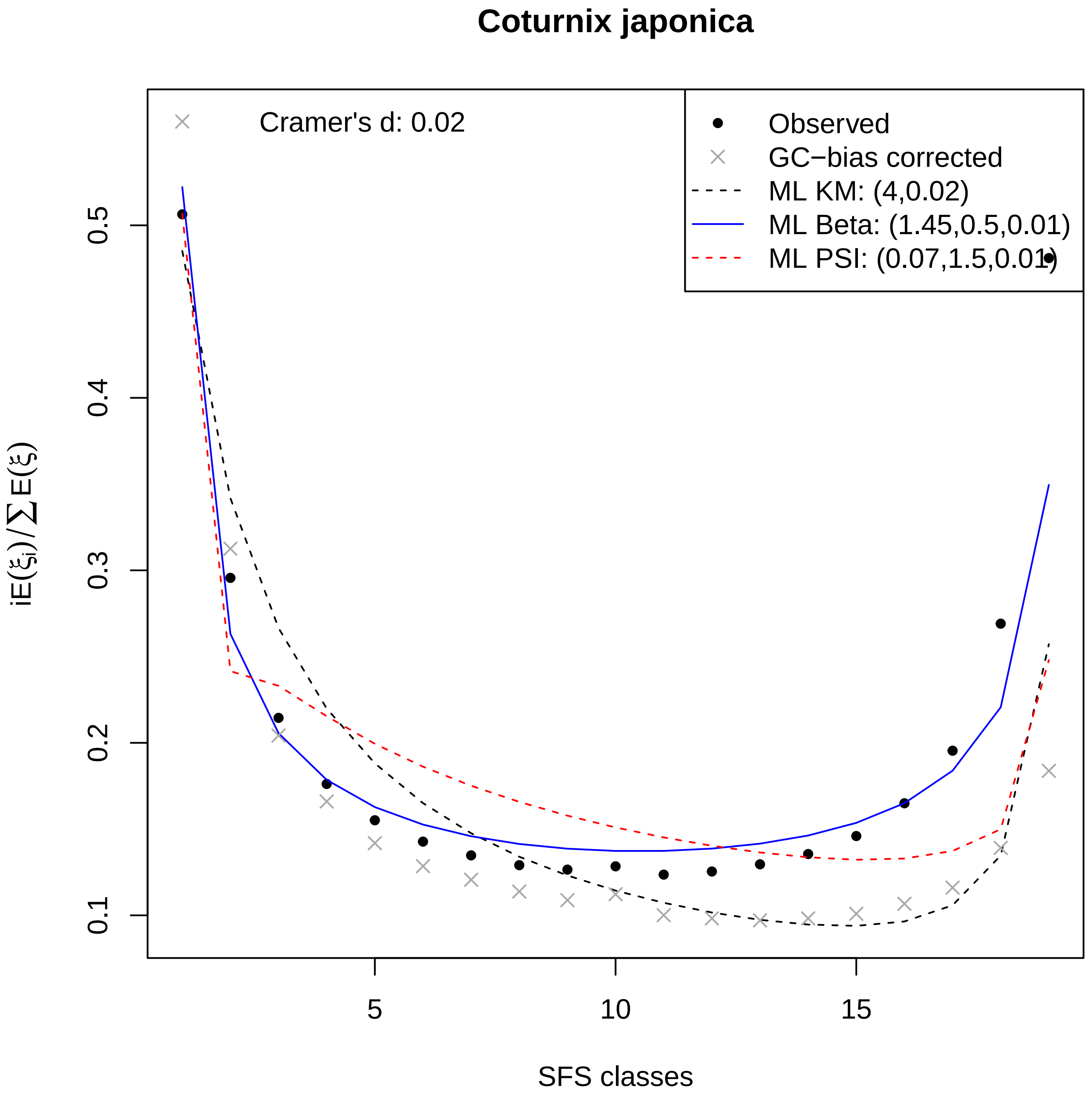
Coturnix japonica
Cramer's d: 0.02
Observed
GC−bias corrected
ML KM: (4,0.02)
ML Beta: (1.45,0.5,0.01)
ML PSI: (0.07,1.5,0.01)
$iE(\xi_i)/\sum E(\xi)$
0.1
0.2
0.3
0.4
0.5
5
10
15
SFS classes

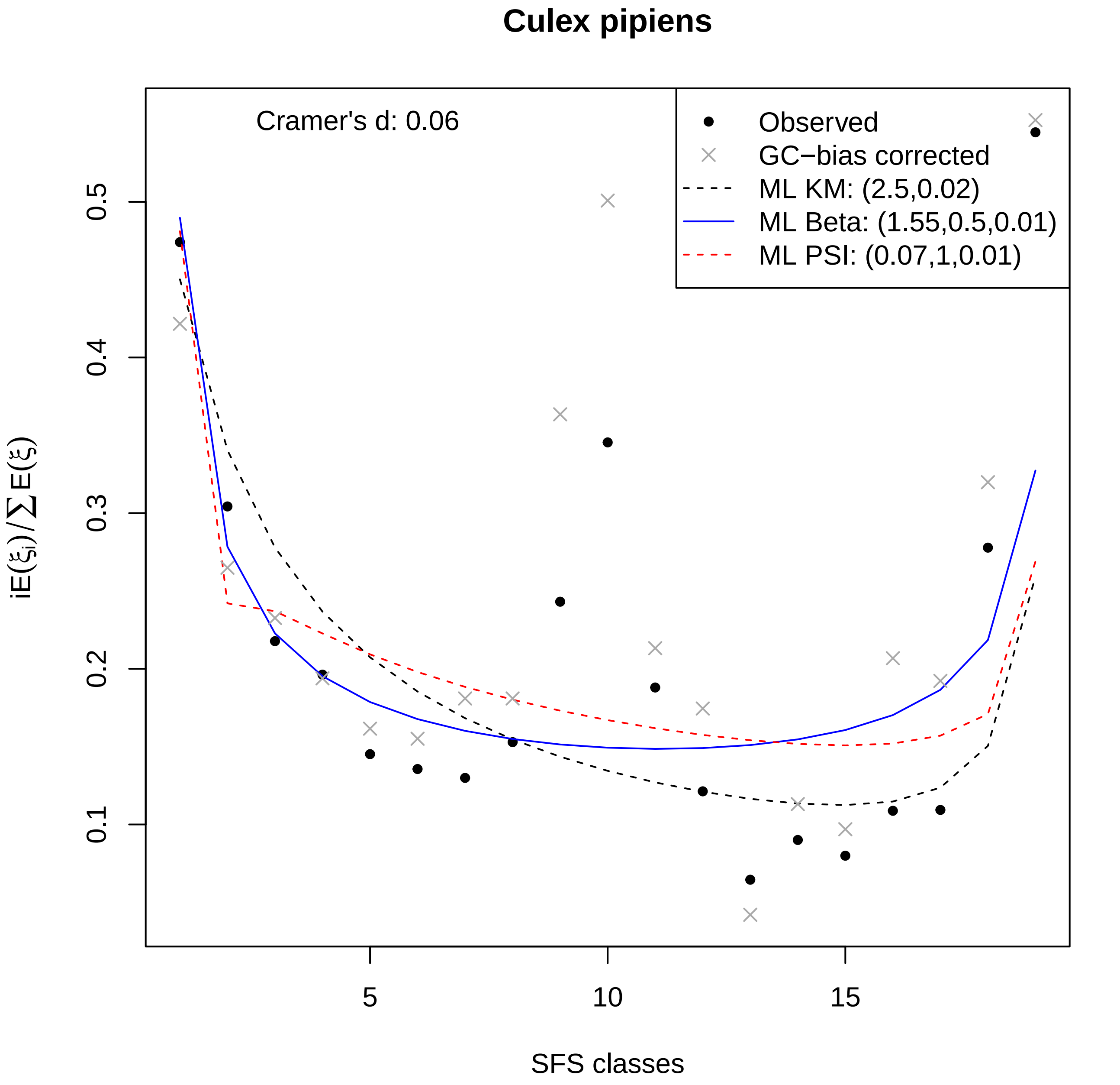

Culex pipiens
Cramer's d: 0.06
Observed
GC−bias corrected
ML KM: (2.5,0.02)
ML Beta: (1.55,0.5,0.01)
ML PSI: (0.07,1,0.01)
$iE(\xi_i)/\sum E(\xi)$
0.1
0.2
0.3
0.4
0.5
5
10
15
SFS classes

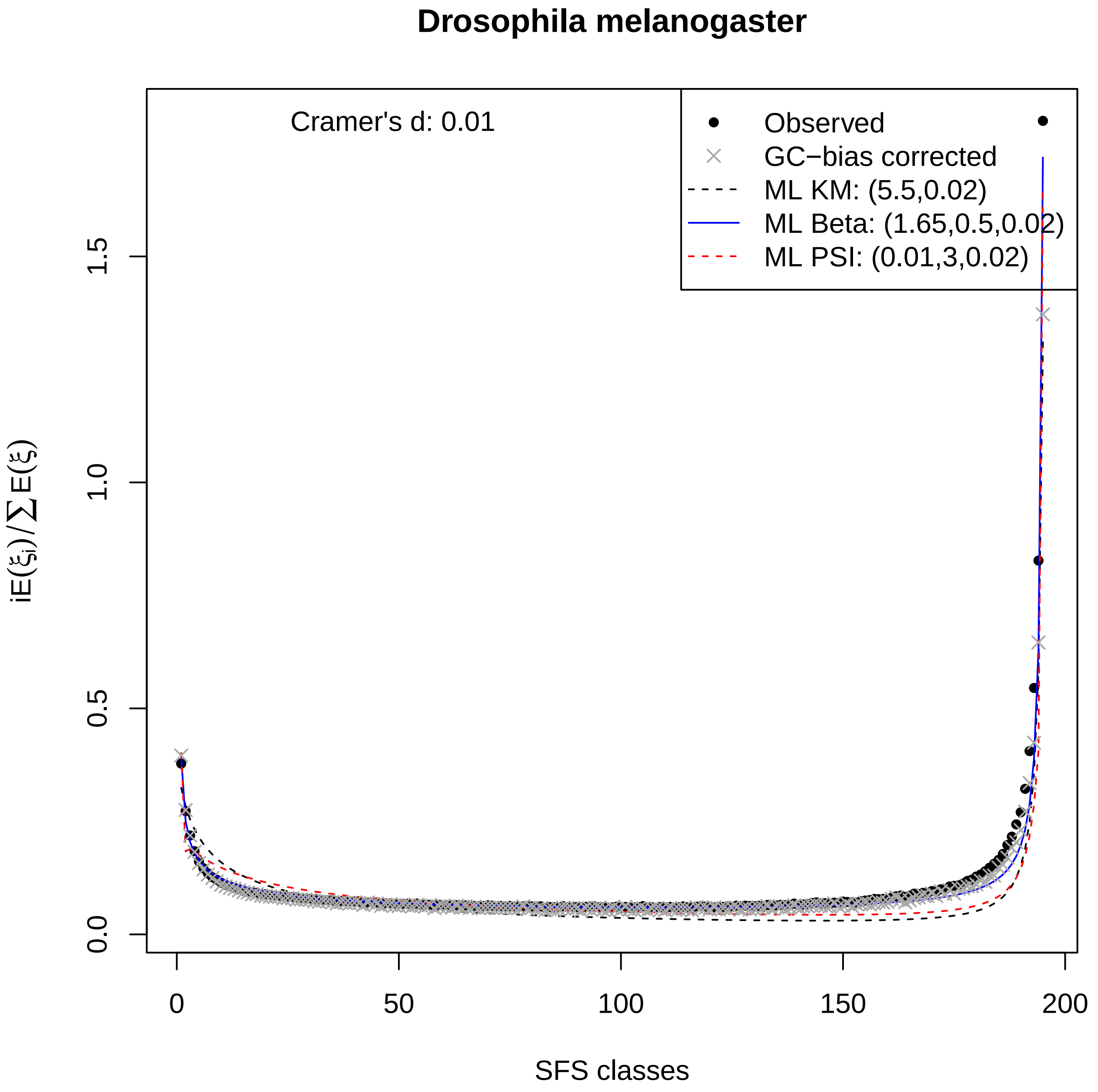
Drosophila melanogaster
Cramer's d: 0.01
Observed
GC−bias corrected
ML KM: (5.5,0.02)
ML Beta: (1.65,0.5,0.02)
ML PSI: (0.01,3,0.02)
$iE(\xi_i)/\sum E(\xi)$
0.0
0.5
1.0
1.5
0
50
100
150
200
SFS classes

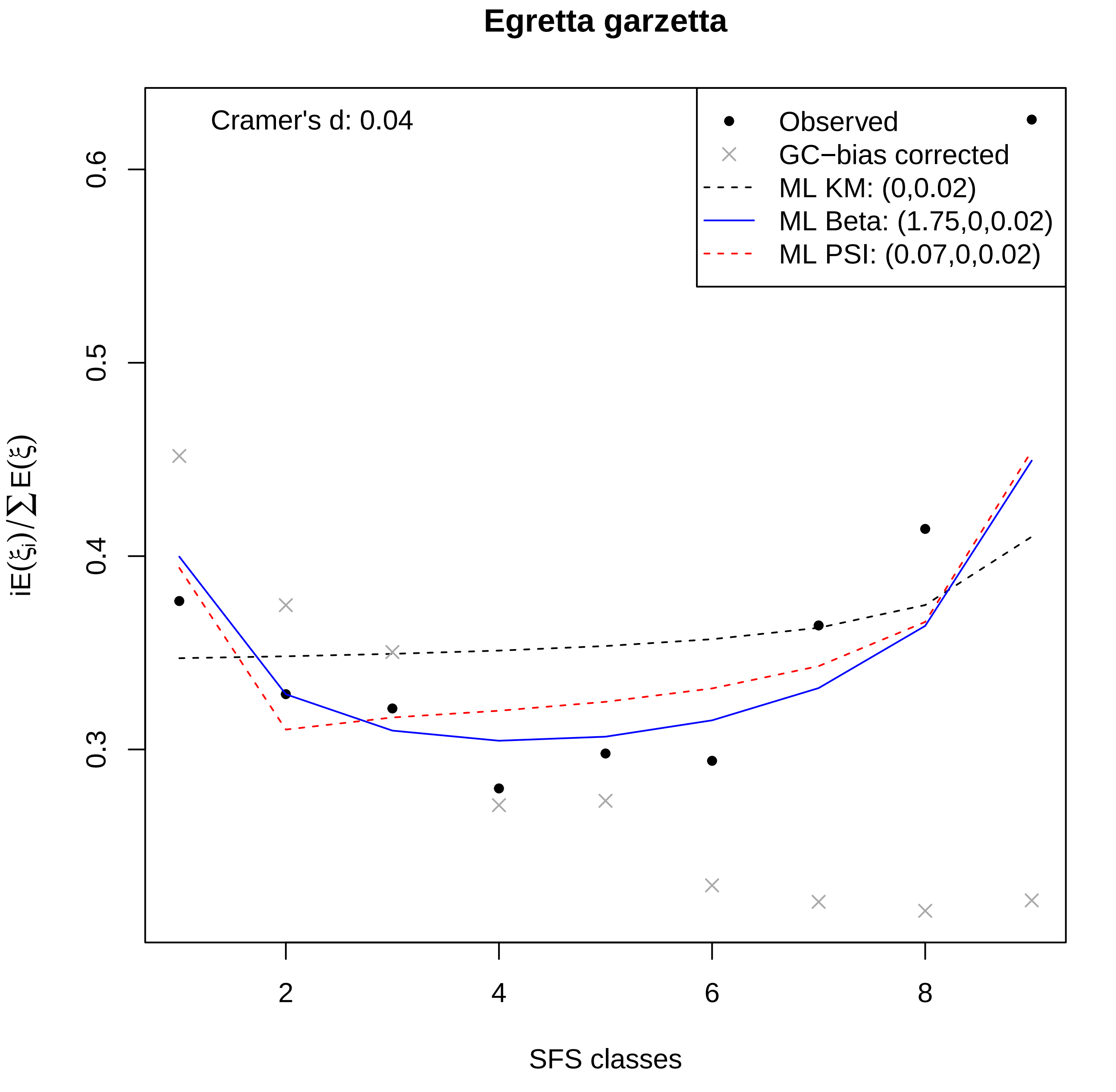
Egretta garzetta
Cramer's d: 0.04
Observed
GC−bias corrected
ML KM: (0,0.02)
ML Beta: (1.75,0,0.02)
ML PSI: (0.07,0,0.02)
0.6
0.5
0.4
0.3
2
4
6
8
SFS classes
iE(ξi)/∑E(ξ)

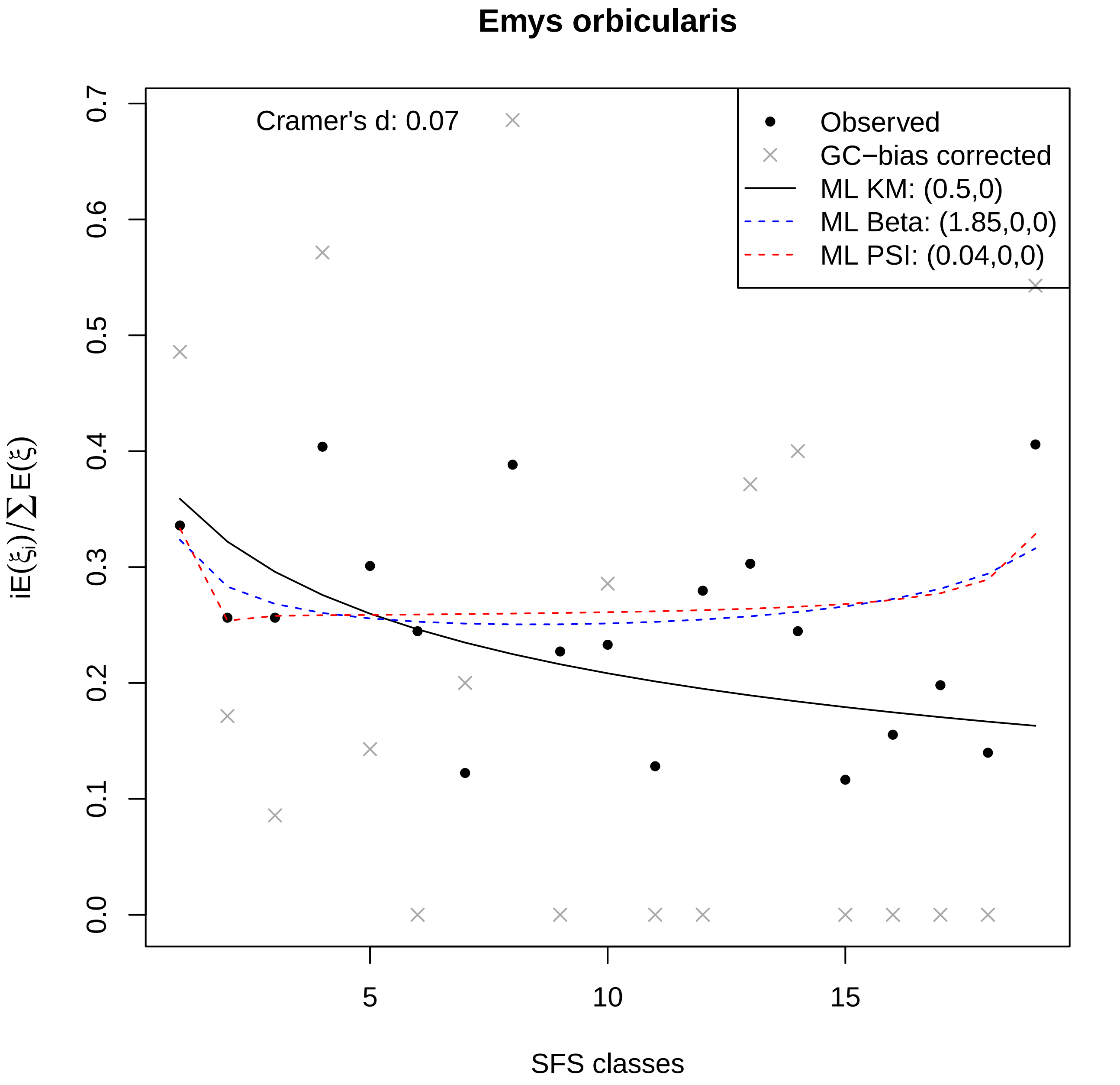
Emys orbicularis
Cramer's d: 0.07
Observed
GC−bias corrected
ML KM: (0.5,0)
ML Beta: (1.85,0,0)
ML PSI: (0.04,0,0)
0.0
0.1
0.2
0.3
0.4
0.5
0.6
0.7
5
10
15
SFS classes
$iE(\xi_i)/\sum E(\xi)$

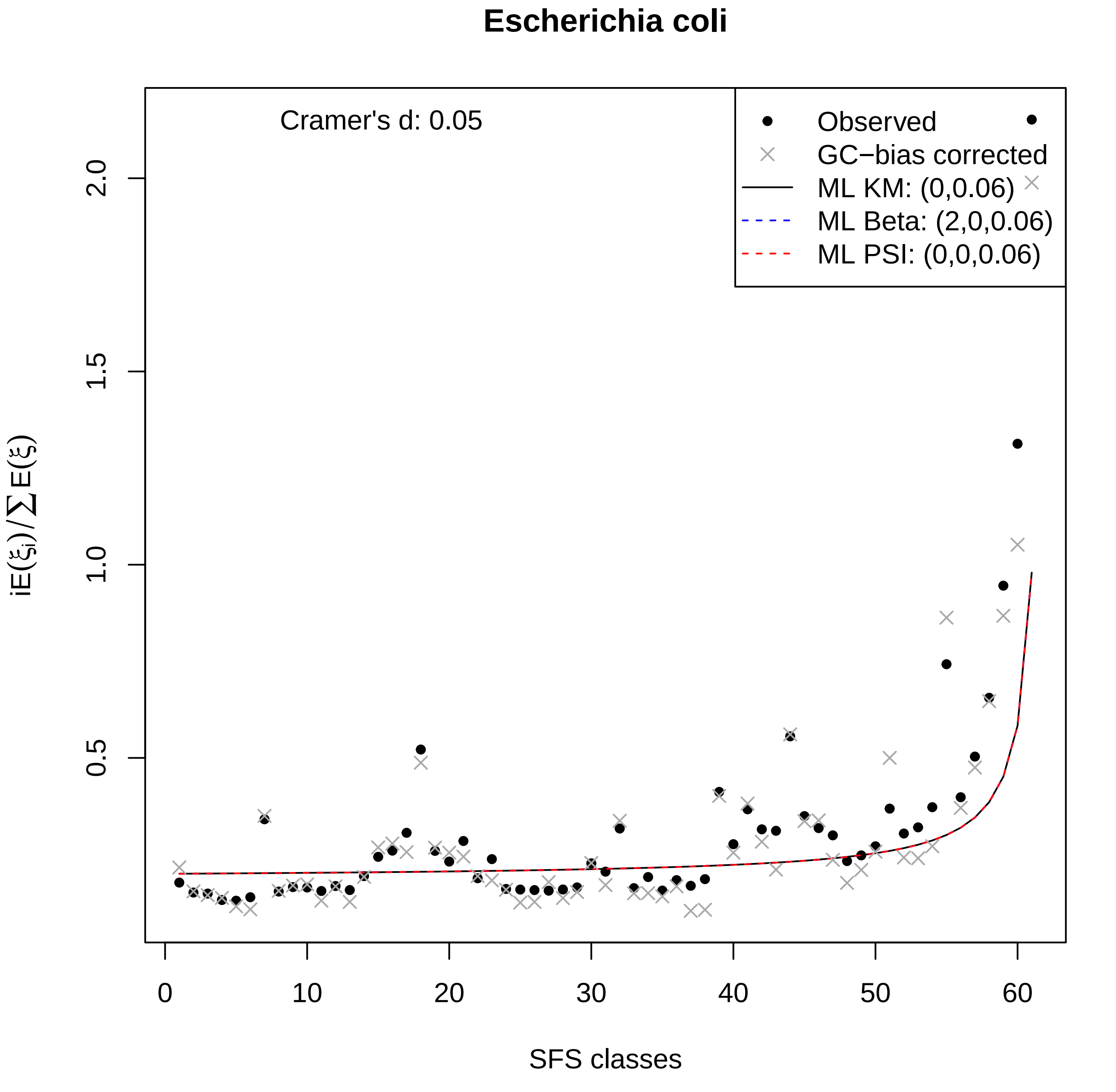
Escherichia coli
Cramer's d: 0.05
Observed
GC−bias corrected
ML KM: (0,0.06)
ML Beta: (2,0,0.06)
ML PSI: (0,0,0.06)
$iE(\xi_i)/\sum E(\xi)$
SFS classes
0.5
1.0
1.5
2.0
0
10
20
30
40
50
60

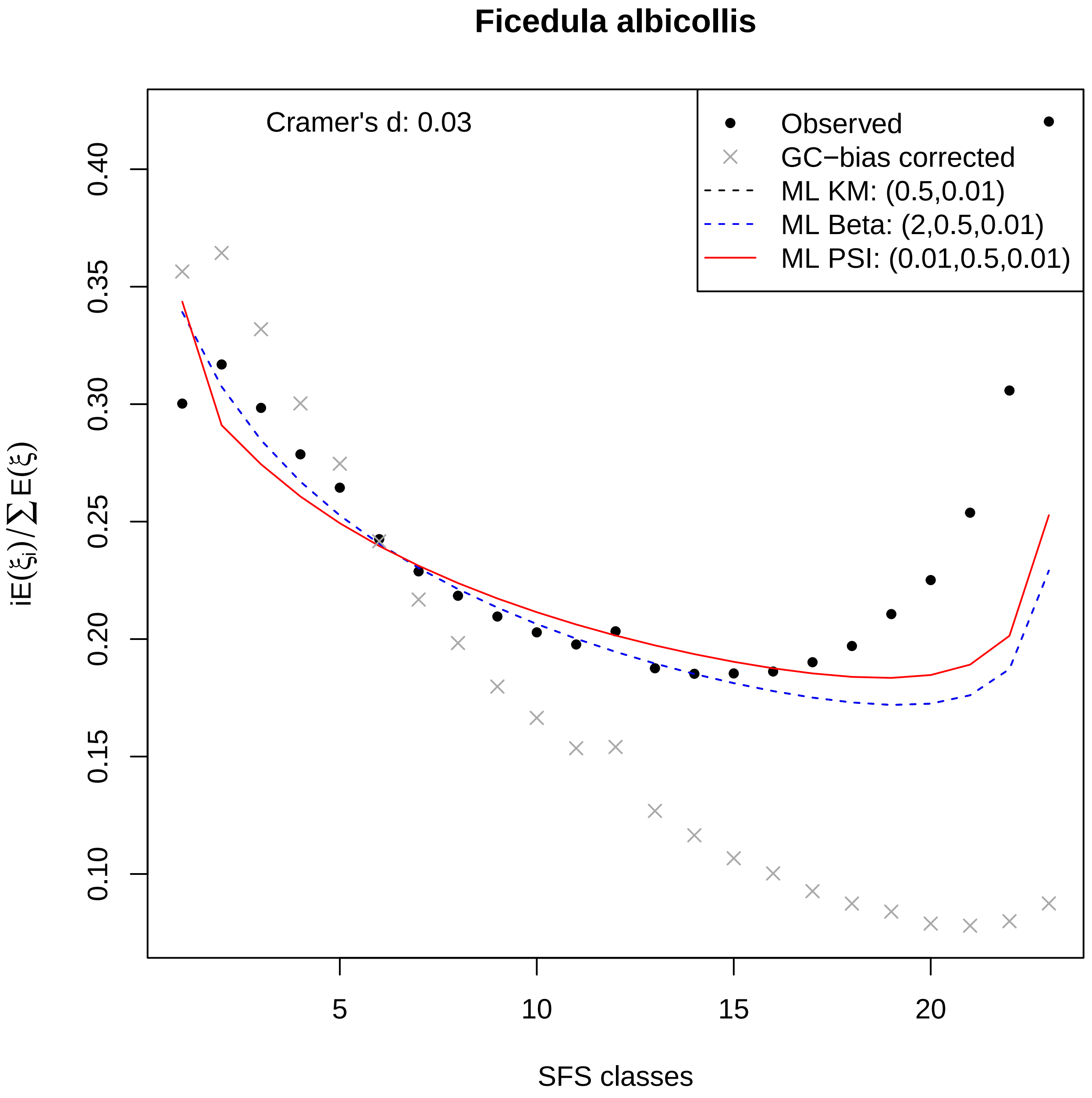

Ficedula albicollis
Cramer's d: 0.03
Observed
GC−bias corrected
ML KM: (0.5,0.01)
ML Beta: (2,0.5,0.01)
ML PSI: (0.01,0.5,0.01)
$iE(\xi_i)/\sum E(\xi)$
0.10
0.15
0.20
0.25
0.30
0.35
0.40
5
10
15
20
SFS classes

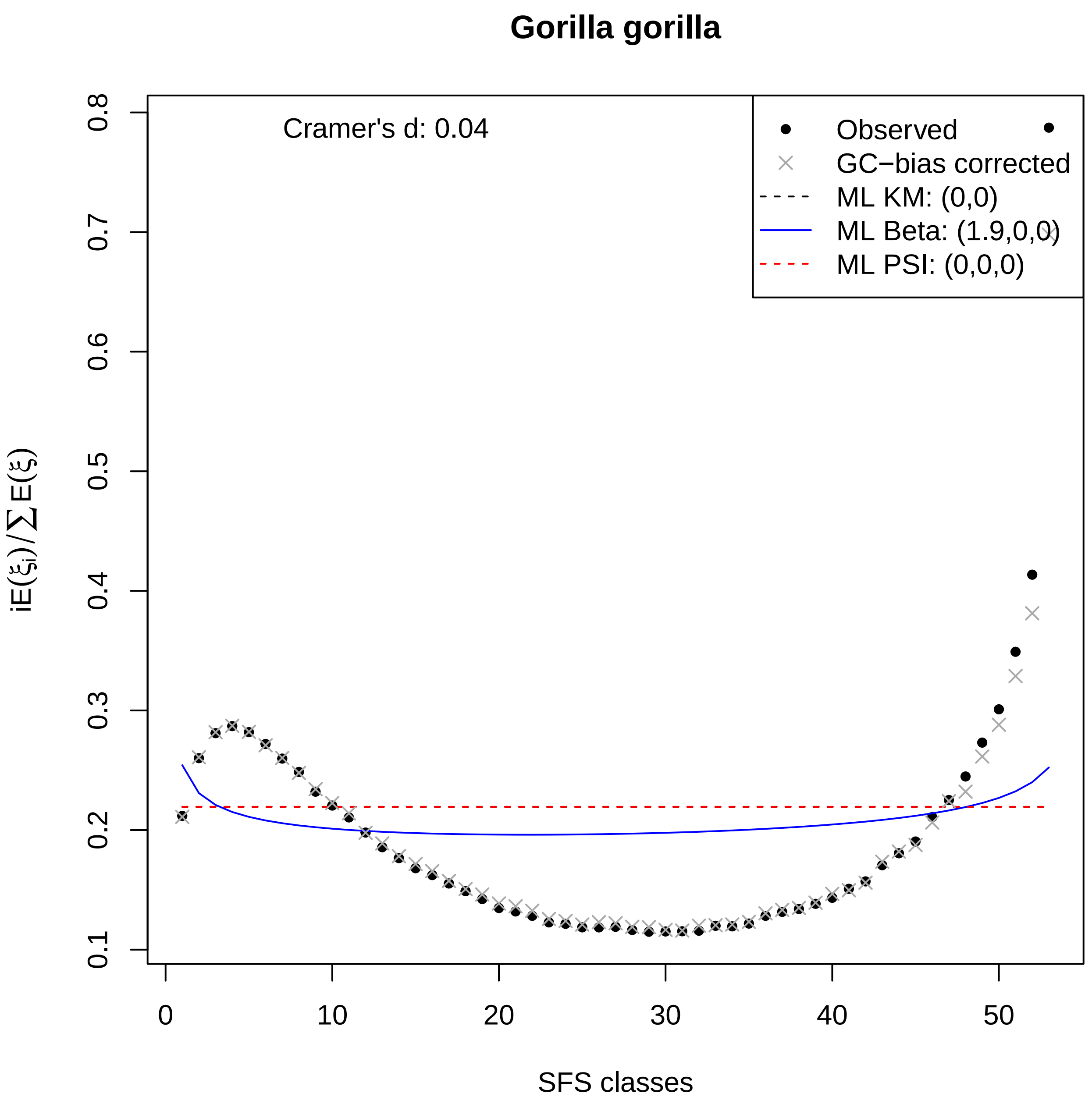

Gorilla gorilla
Cramer's d: 0.04
Observed
GC−bias corrected
ML KM: (0,0)
ML Beta: (1.9,0,0)
ML PSI: (0,0,0)
0.1
0.2
0.3
0.4
0.5
0.6
0.7
0.8
0
10
20
30
40
50
SFS classes
$iE(\xi_i)/\sum E(\xi)$

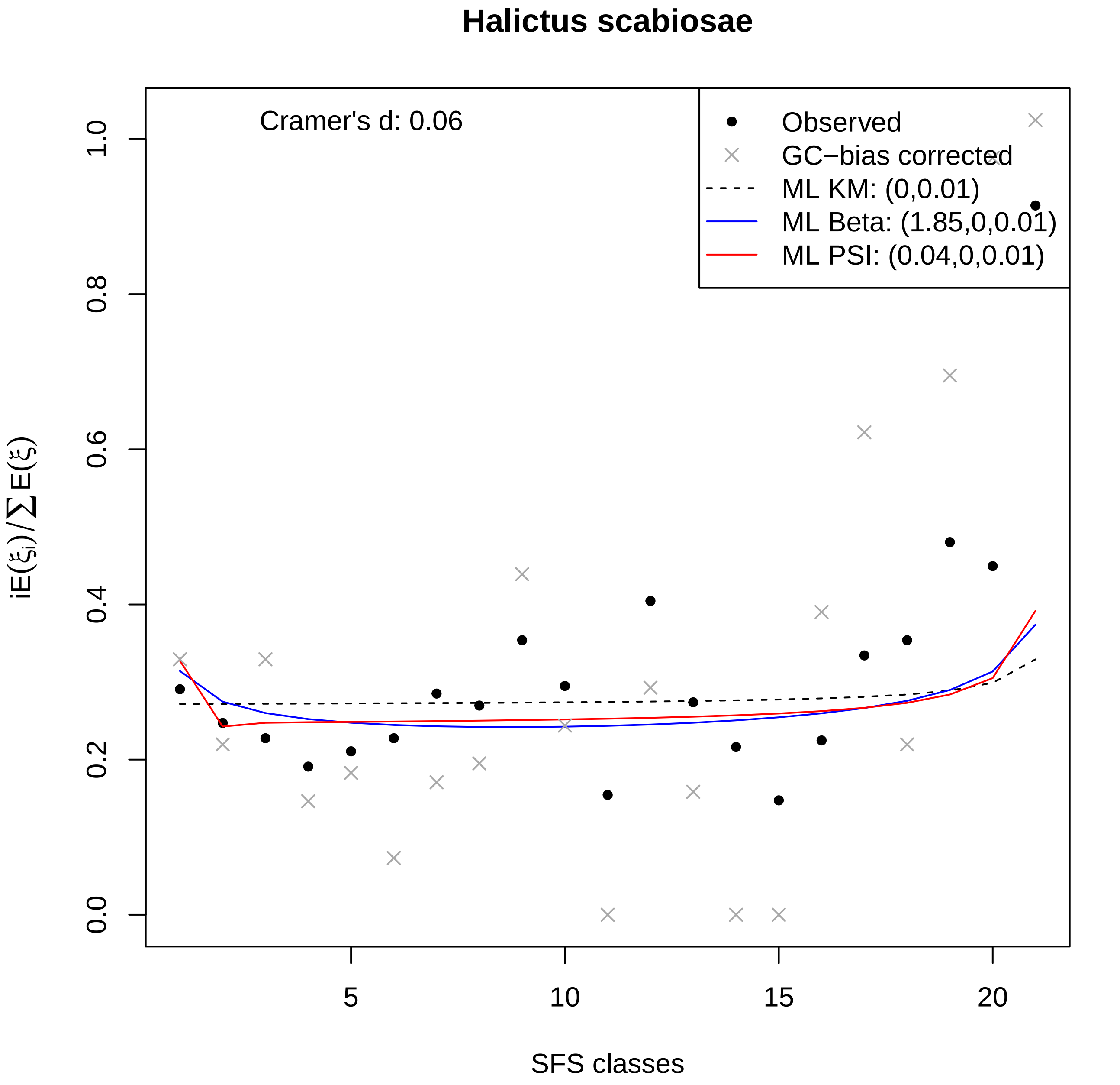

Halictus scabiosae
Cramer's d: 0.06
Observed
GC−bias corrected
ML KM: (0,0.01)
ML Beta: (1.85,0,0.01)
ML PSI: (0.04,0,0.01)
iE(ξi)/∑E(ξ)
0.0
0.2
0.4
0.6
0.8
1.0
5
10
15
20
SFS classes

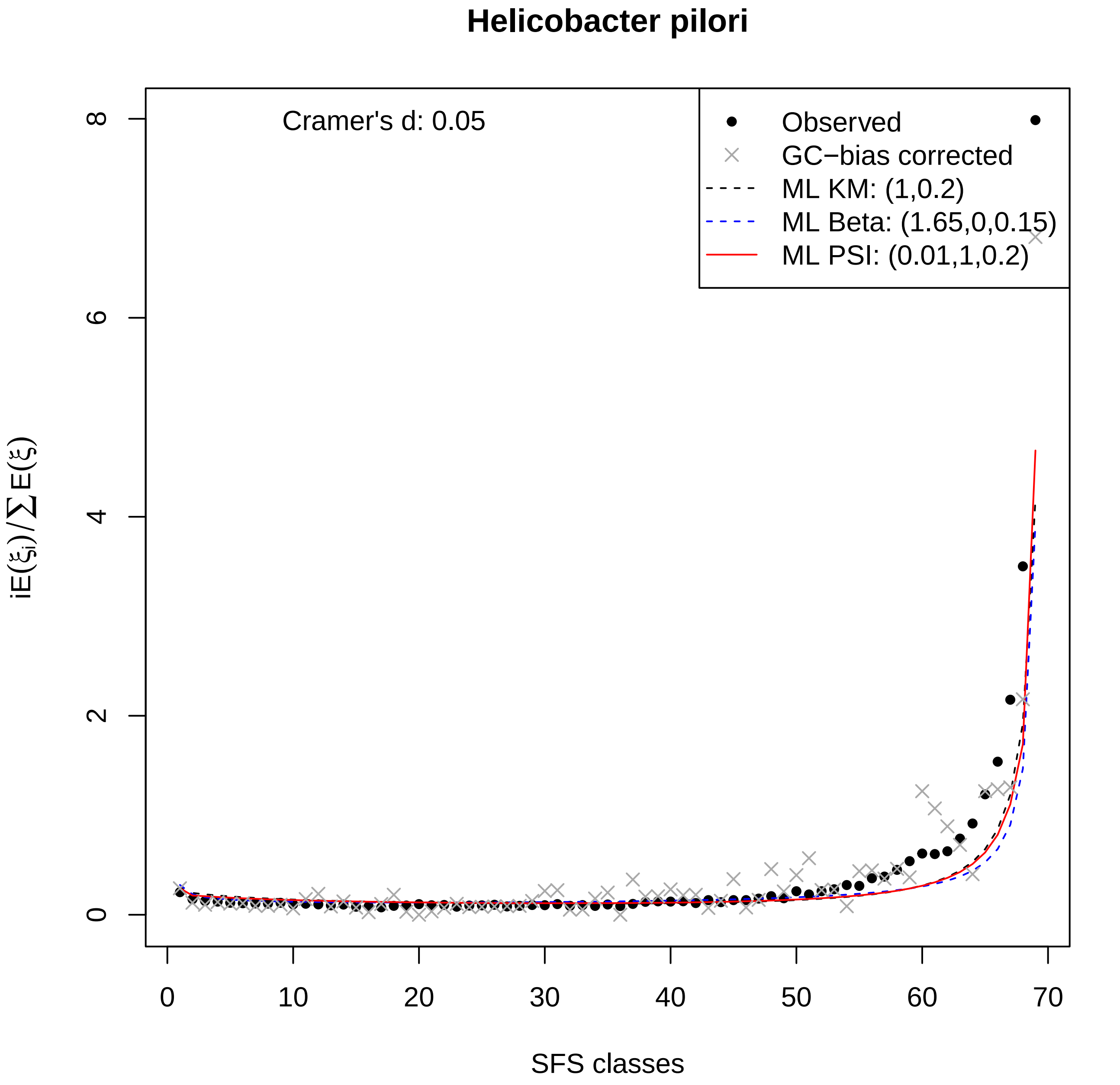
Helicobacter pilori
Cramer's d: 0.05
Observed
GC−bias corrected
ML KM: (1,0.2)
ML Beta: (1.65,0,0.15)
ML PSI: (0.01,1,0.2)
$iE(\xi_i)/\sum E(\xi)$
SFS classes
0
2
4
6
8
0
10
20
30
40
50
60
70

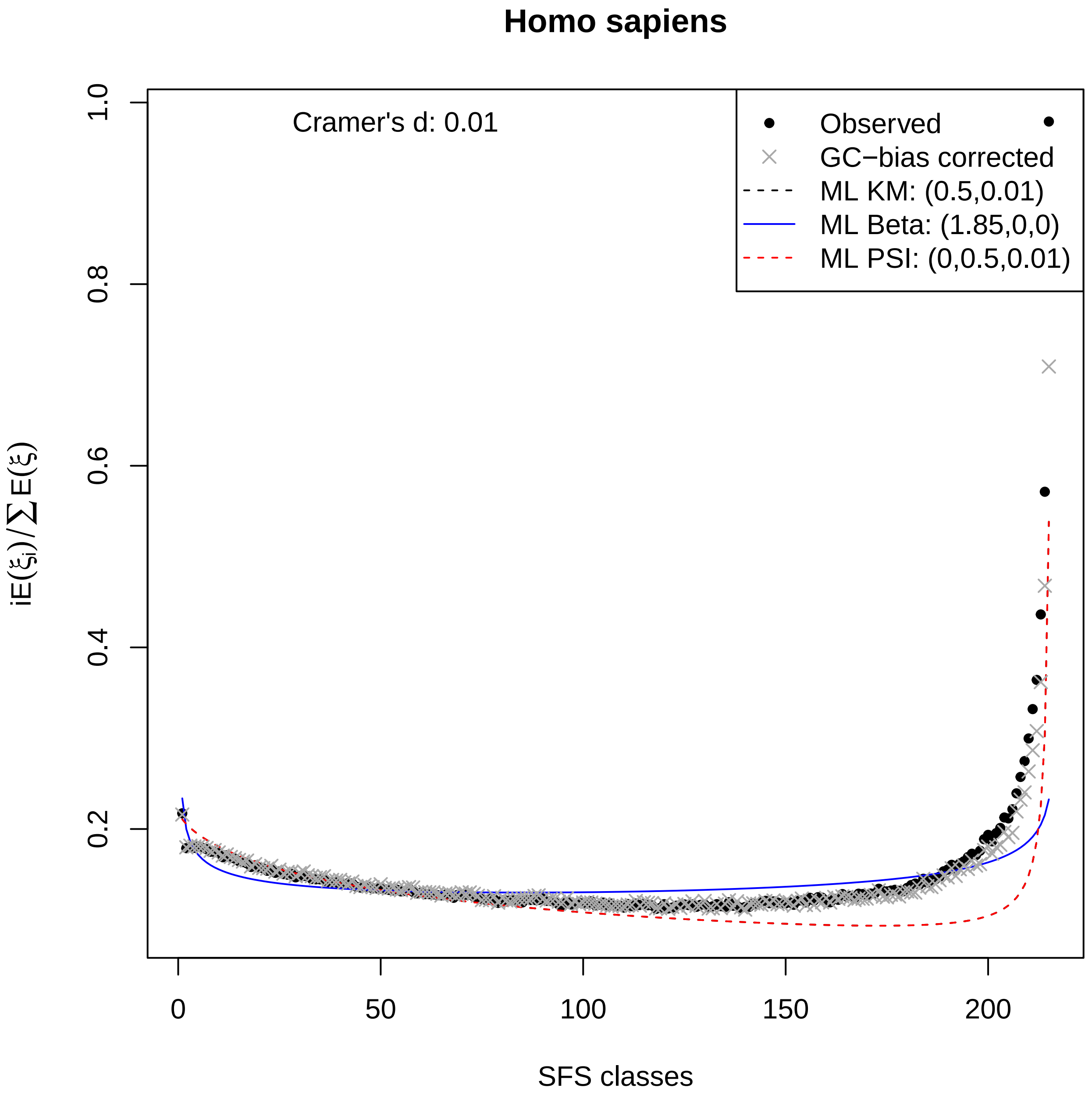
Homo sapiens
Cramer's d: 0.01
Observed
GC−bias corrected
ML KM: (0.5,0.01)
ML Beta: (1.85,0,0)
ML PSI: (0,0.5,0.01)
iE(ξi)/∑E(ξ)
SFS classes
0.2
0.4
0.6
0.8
1.0
0
50
100
150
200

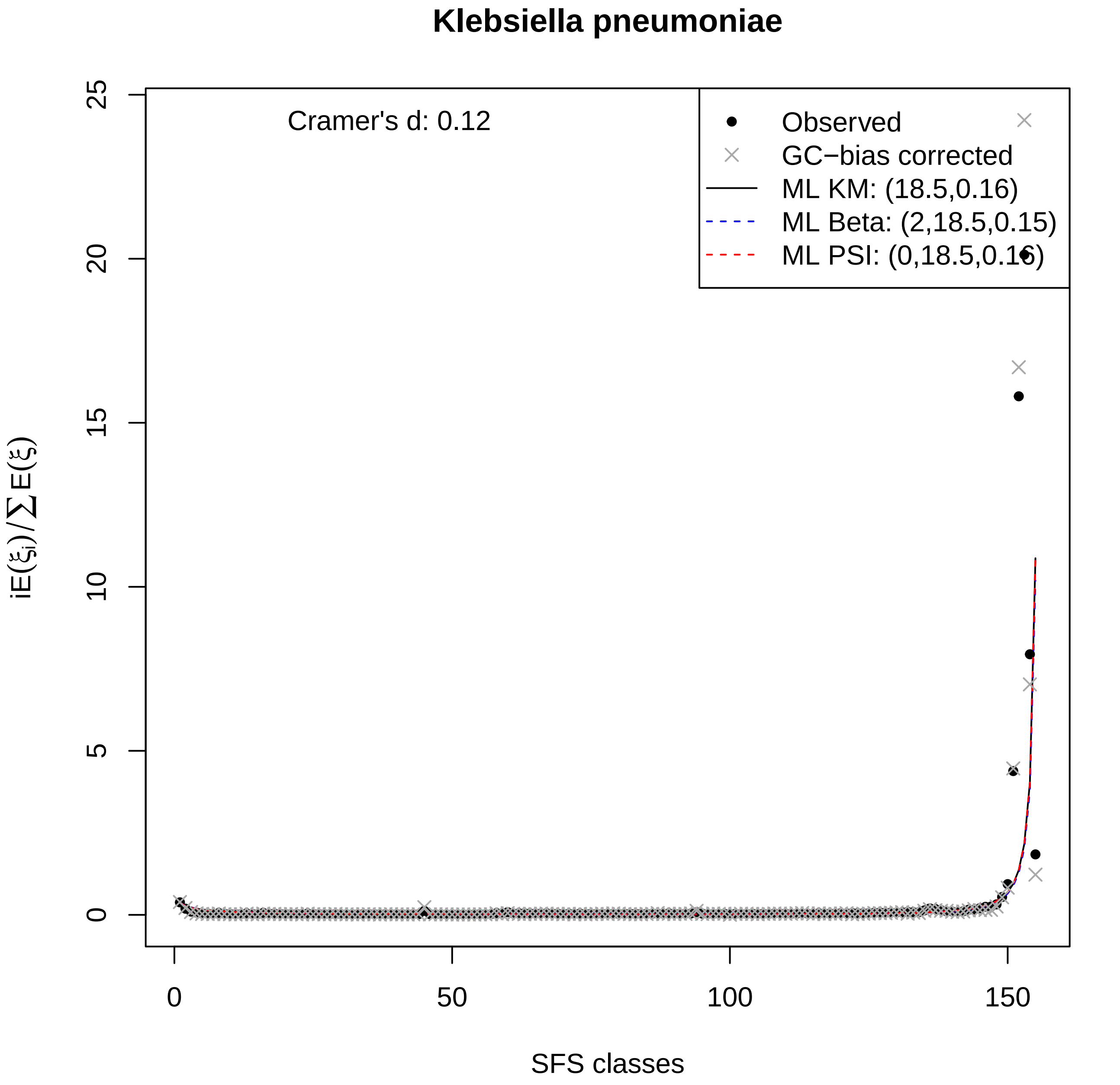

Klebsiella pneumoniae
Cramer's d: 0.12
Observed
GC−bias corrected
ML KM: (18.5,0.16)
ML Beta: (2,18.5,0.15)
ML PSI: (0,18.5,0.16)
0
5
10
15
20
25
0
50
100
150
SFS classes
iE(ξi)/∑E(ξ)

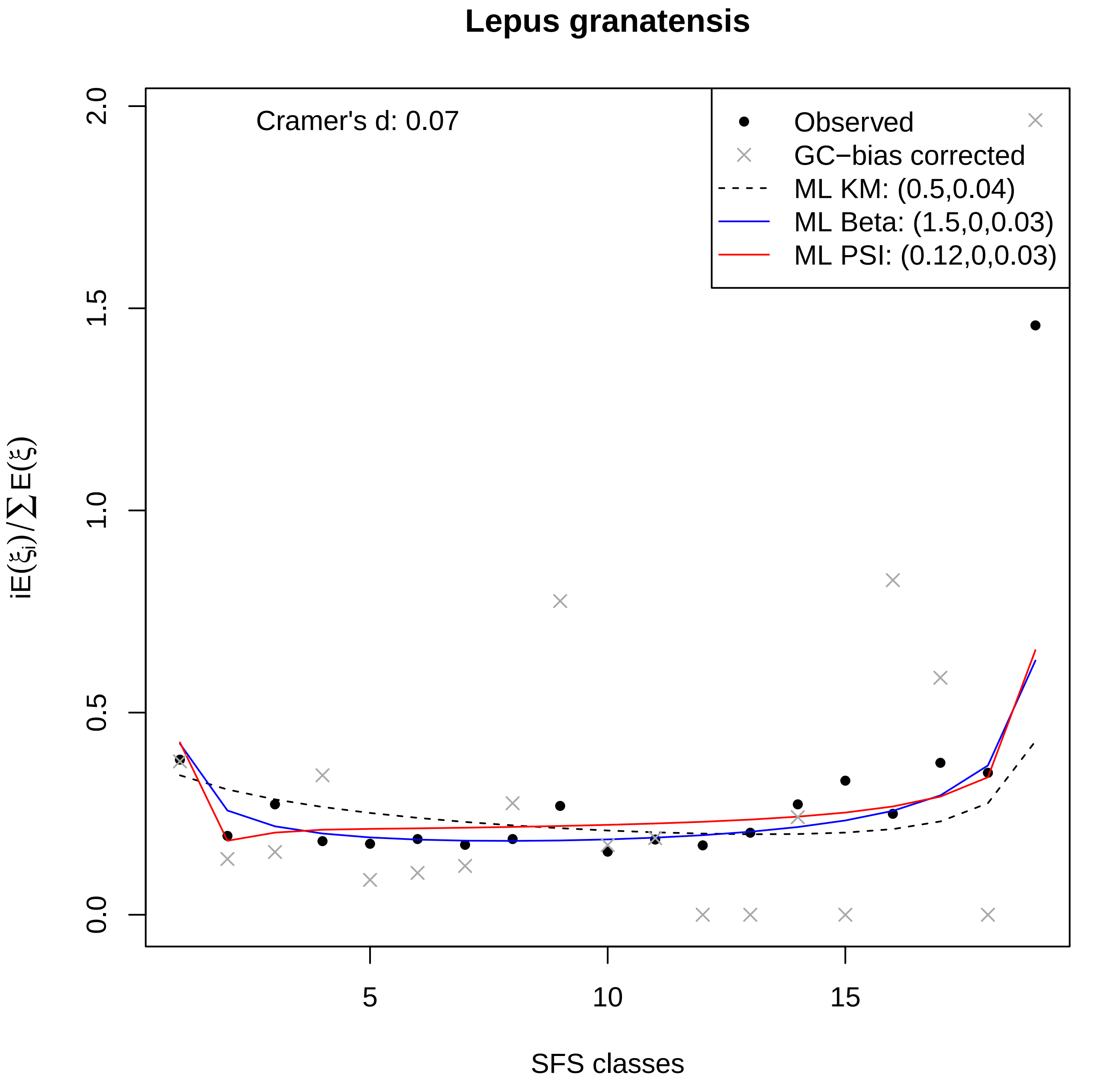
Lepus granatensis
Cramer's d: 0.07
Observed
GC−bias corrected
ML KM: (0.5,0.04)
ML Beta: (1.5,0,0.03)
ML PSI: (0.12,0,0.03)
0.0
0.5
1.0
1.5
2.0
5
10
15
SFS classes
$iE(\xi_i)/\sum E(\xi)$

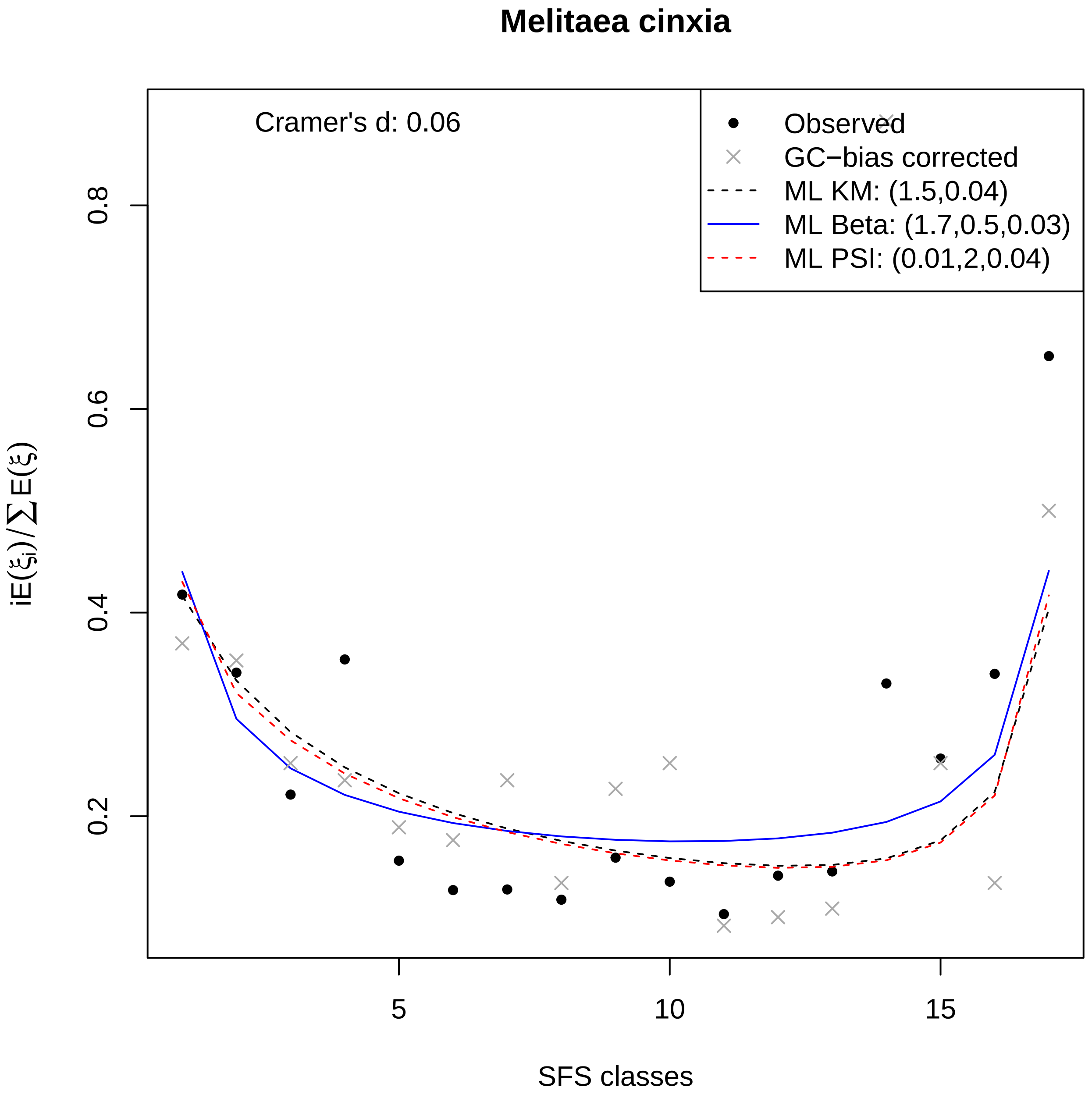

Melitaea cinxia
Cramer's d: 0.06
Observed
GC−bias corrected
ML KM: (1.5,0.04)
ML Beta: (1.7,0.5,0.03)
ML PSI: (0.01,2,0.04)
$iE(\xi_i)/\sum E(\xi)$
0.2
0.4
0.6
0.8
5
10
15
SFS classes

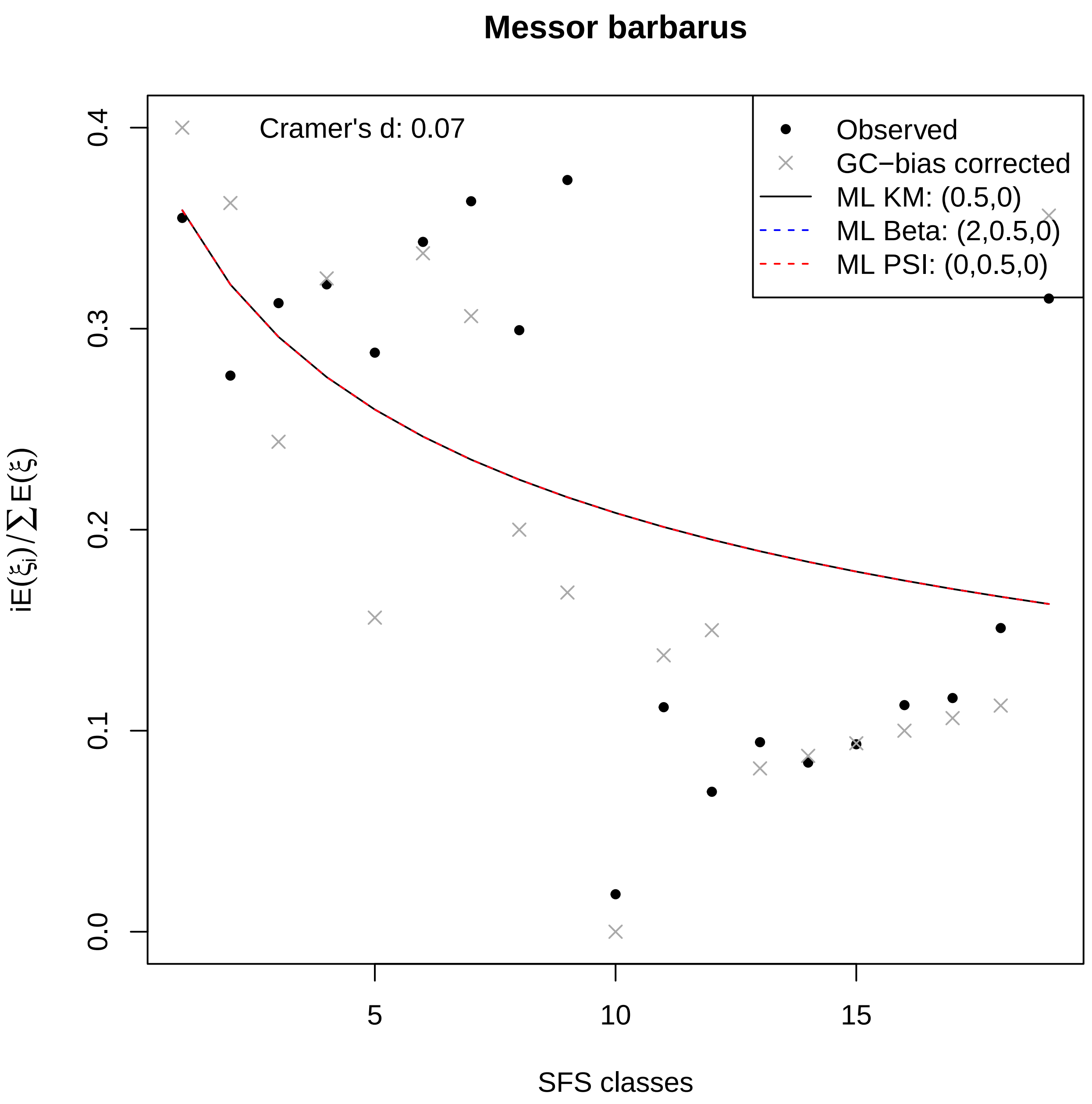

Messor barbarus
Cramer's d: 0.07
Observed
GC−bias corrected
ML KM: (0.5,0)
ML Beta: (2,0.5,0)
ML PSI: (0,0.5,0)
0.0
0.1
0.2
0.3
0.4
5
10
15
SFS classes

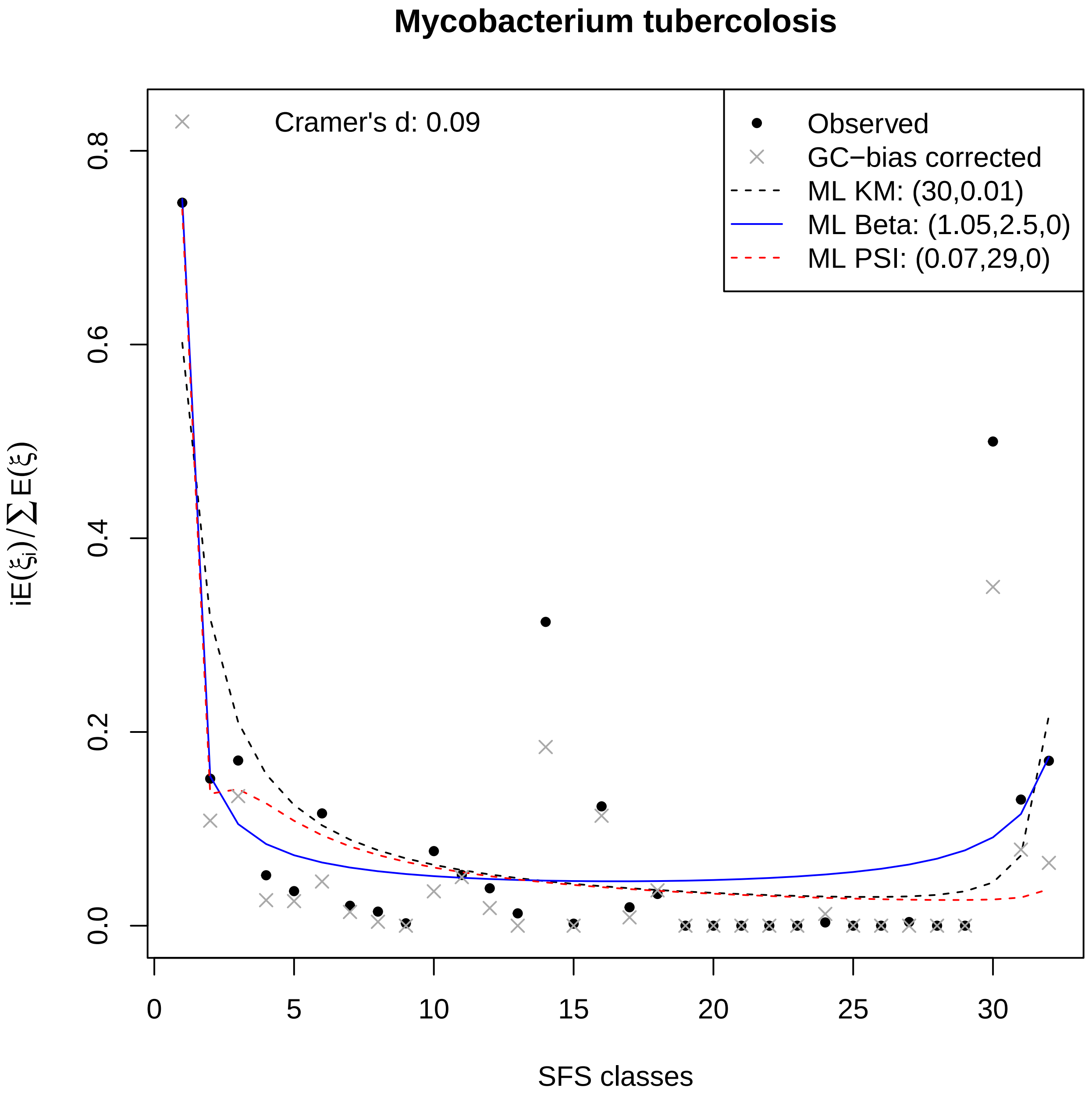

Mycobacterium tubercolosis
Cramer's d: 0.09
Observed
GC−bias corrected
ML KM: (30,0.01)
ML Beta: (1.05,2.5,0)
ML PSI: (0.07,29,0)
0.0
0.2
0.4
0.6
0.8
0
5
10
15
20
25
30
$iE(\xi_i)/\sum E(\xi)$
SFS classes

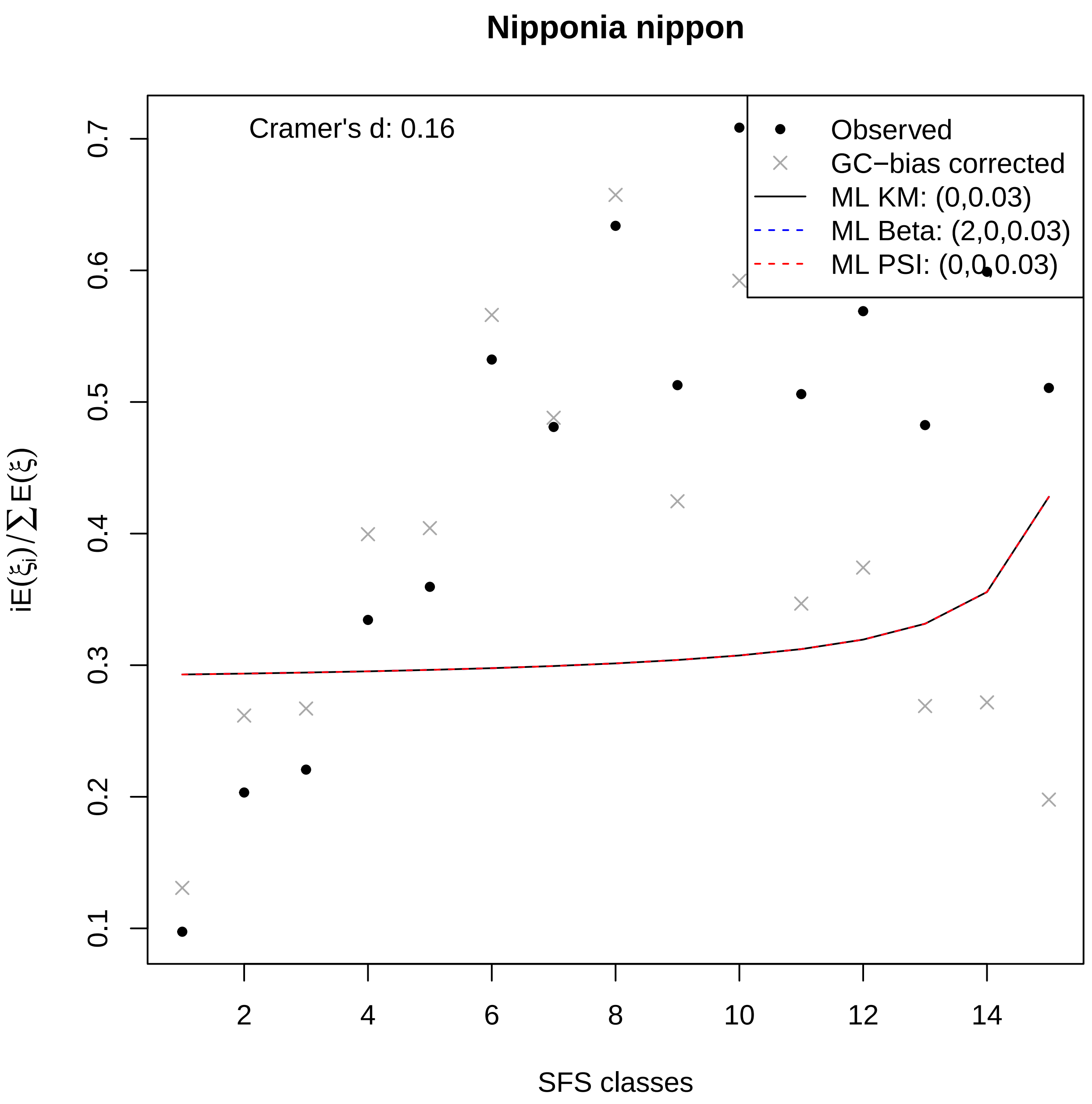

Nipponia nippon
Cramer's d: 0.16
Observed
GC−bias corrected
ML KM: (0,0.03)
ML Beta: (2,0,0.03)
ML PSI: (0,0,0.03)
iE(ξi)/∑E(ξ)
0.1
0.2
0.3
0.4
0.5
0.6
0.7
2
4
6
8
10
12
14
SFS classes

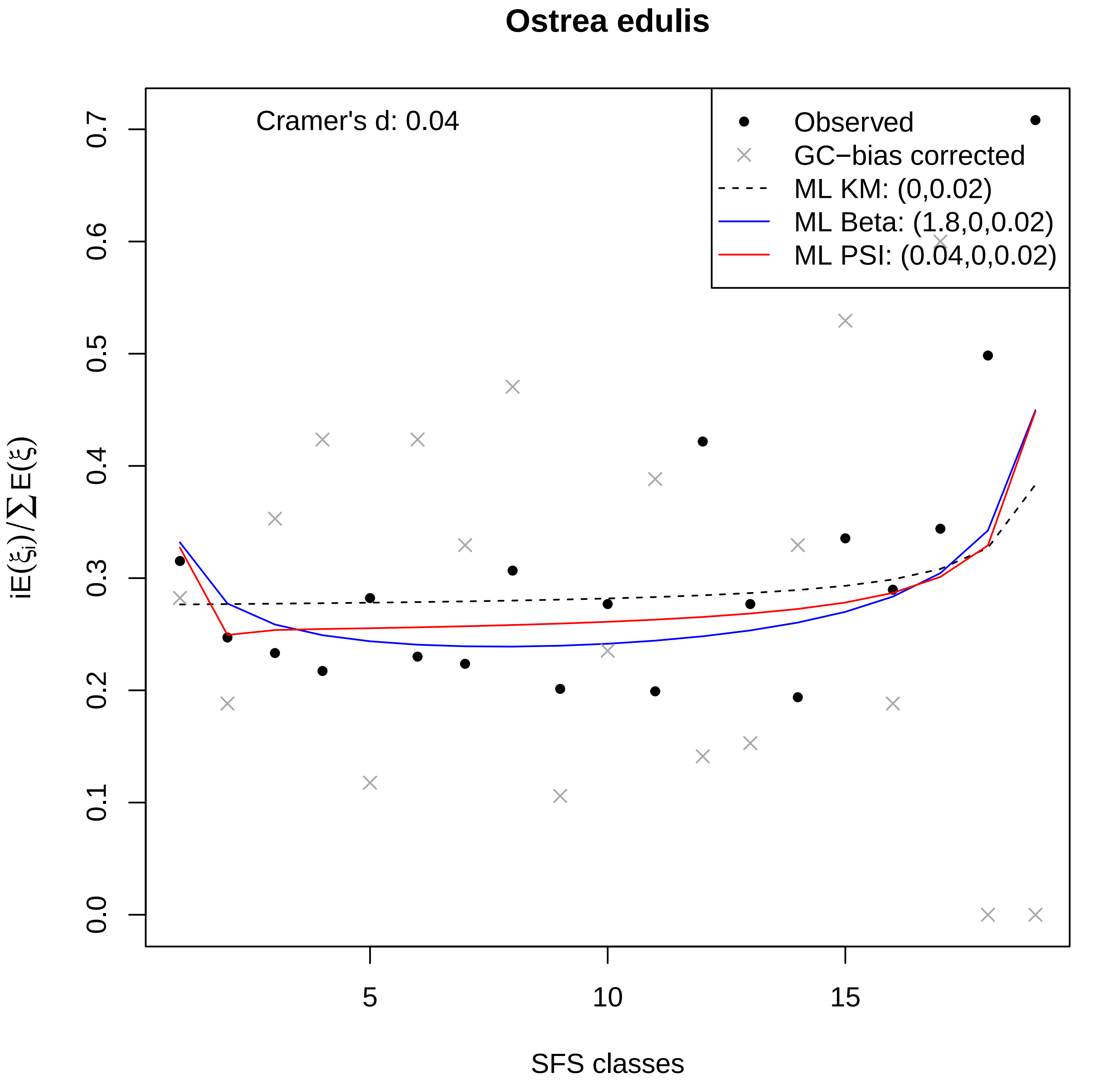

Ostrea edulis
Cramer's d: 0.04
Observed
GC−bias corrected
ML KM: (0,0.02)
ML Beta: (1.8,0,0.02)
ML PSI: (0.04,0,0.02)
$iE(\xi_i)/\sum E(\xi)$
SFS classes
0.0
0.1
0.2
0.3
0.4
0.5
0.6
0.7
5
10
15

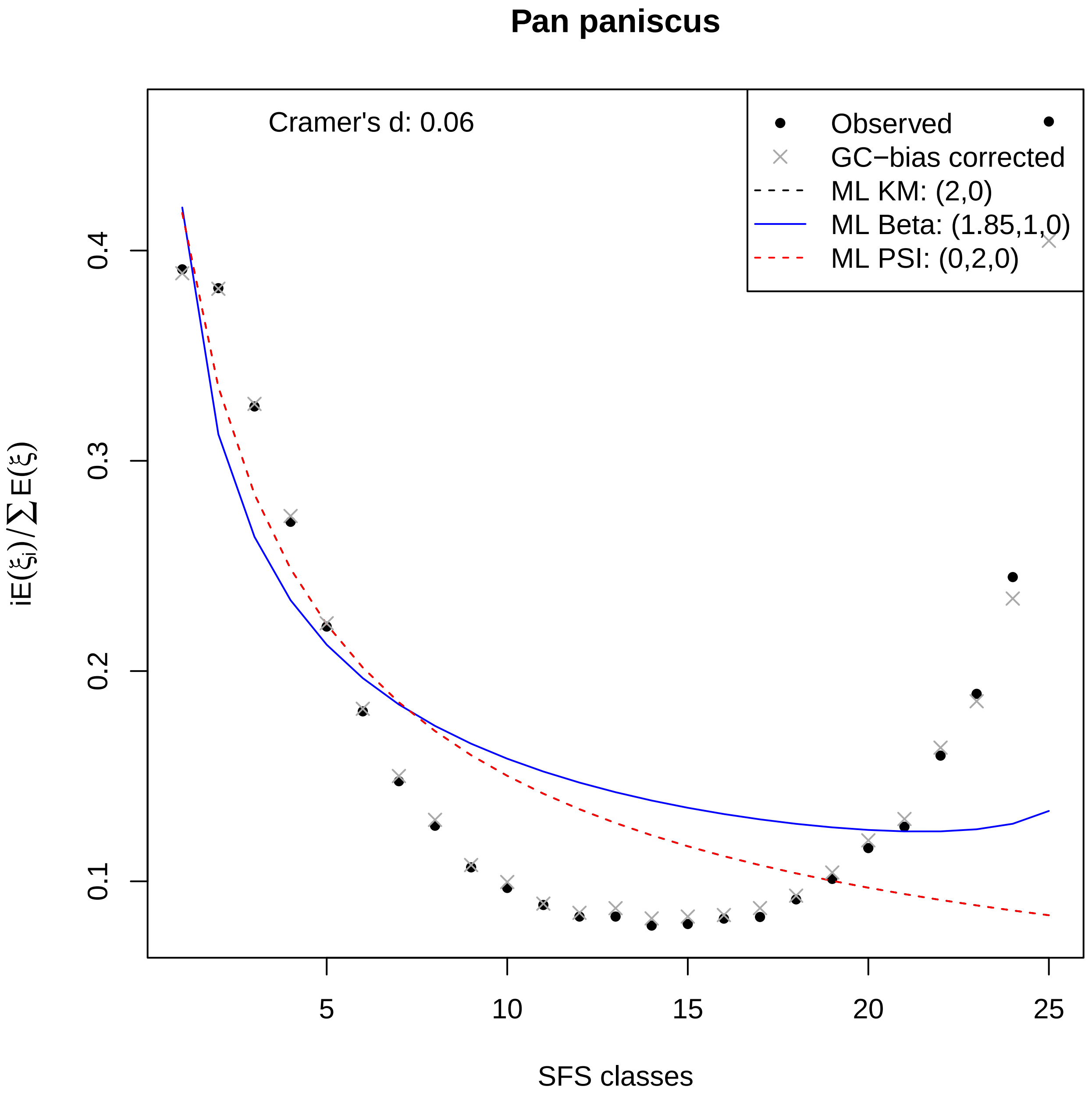
Pan paniscus
Cramer's d: 0.06
Observed
GC−bias corrected
ML KM: (2,0)
ML Beta: (1.85,1,0)
ML PSI: (0,2,0)
0.1
0.2
0.3
0.4
5
10
15
20
25
SFS classes
iE(ξi)/∑E(ξ)

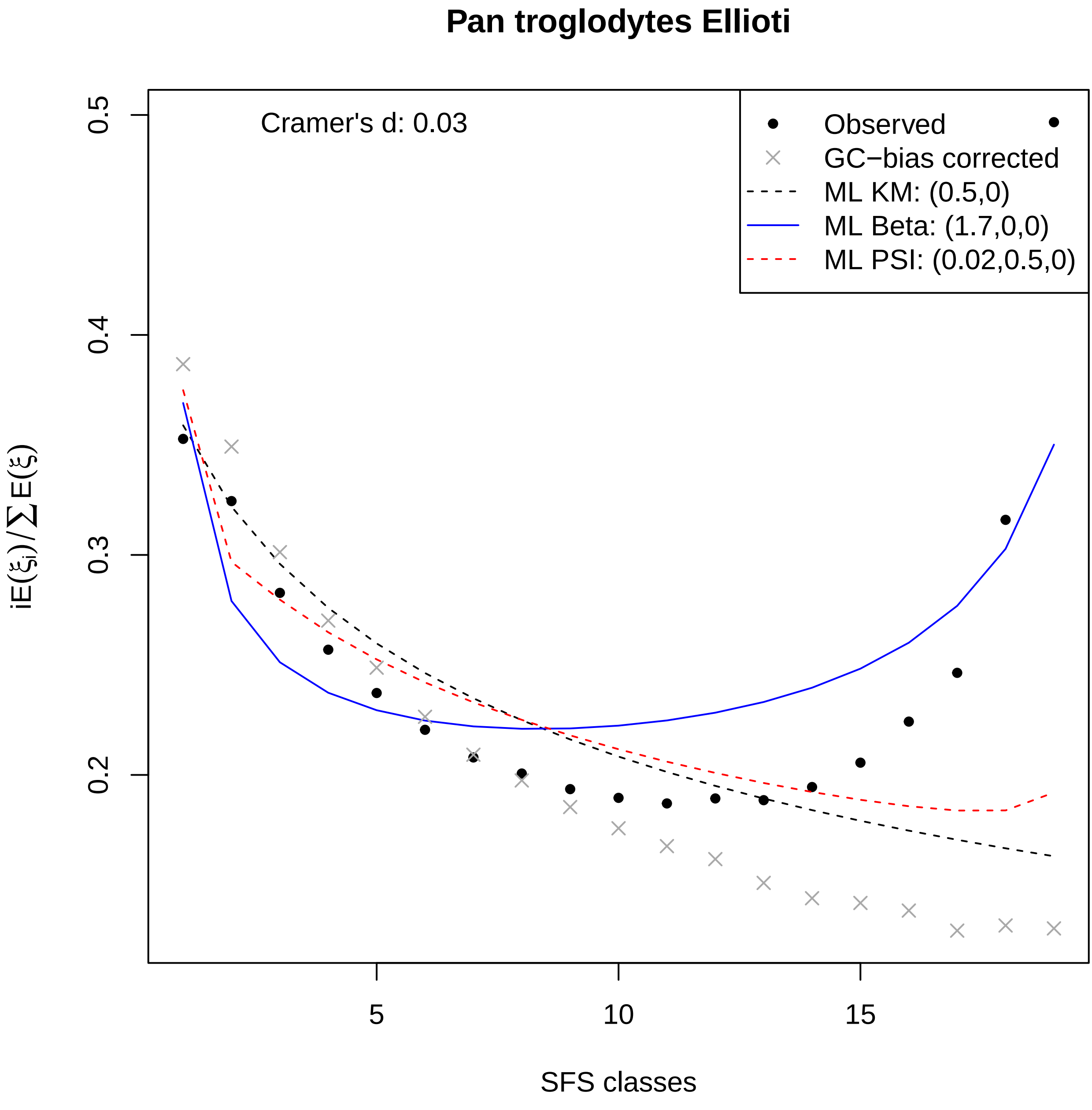

Pan troglodytes Ellioti
Cramer's d: 0.03
Observed
GC−bias corrected
ML KM: (0.5,0)
ML Beta: (1.7,0,0)
ML PSI: (0.02,0.5,0)
0.2
0.3
0.4
0.5
5
10
15
SFS classes
$iE(\xi_i)/\sum E(\xi)$

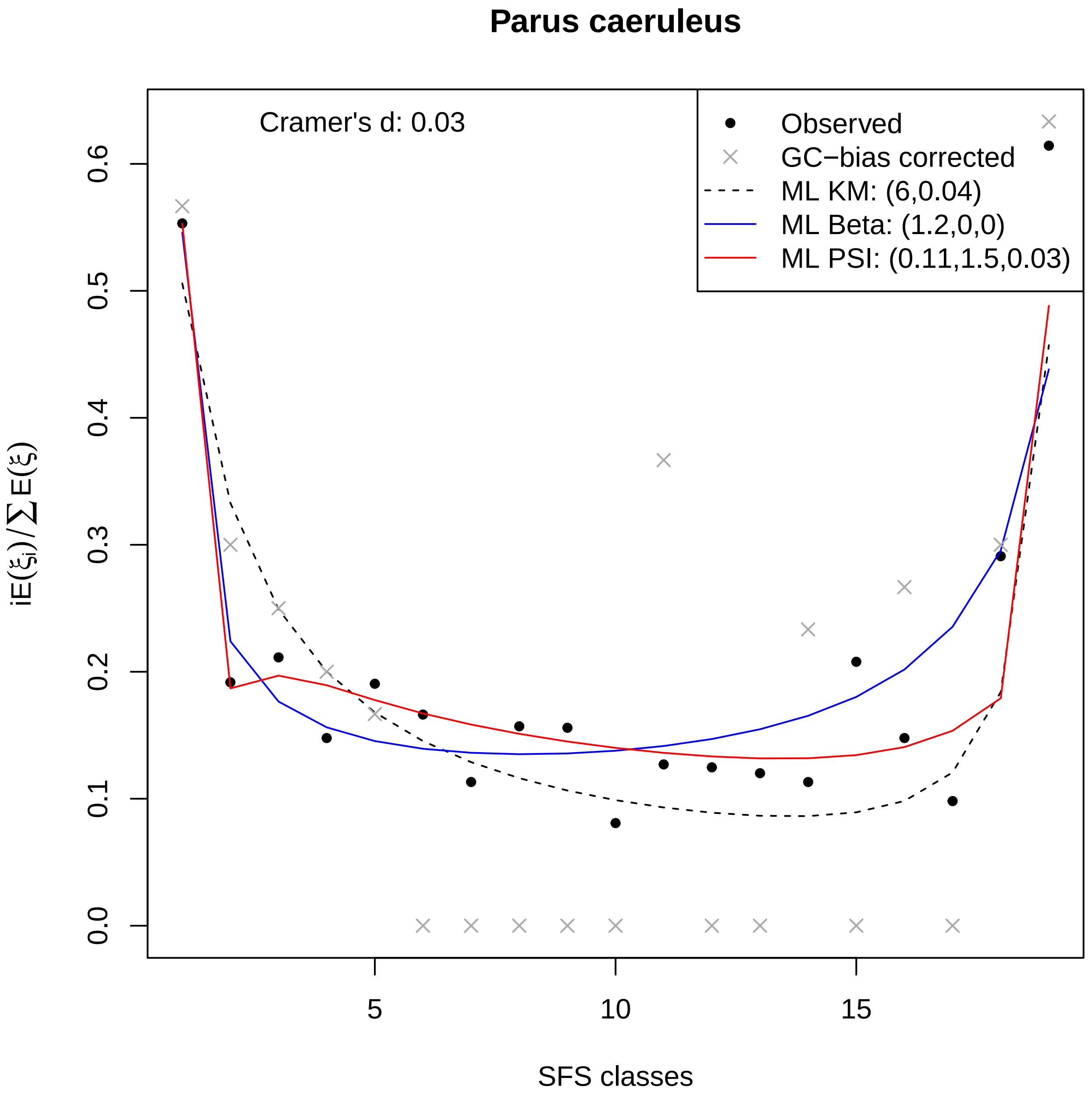

Parus caeruleus
Cramer's d: 0.03
Observed
GC−bias corrected
ML KM: (6,0.04)
ML Beta: (1.2,0,0)
ML PSI: (0.11,1.5,0.03)
0.0
0.1
0.2
0.3
0.4
0.5
0.6
5
10
15
SFS classes
iE(ξi)/∑E(ξ)

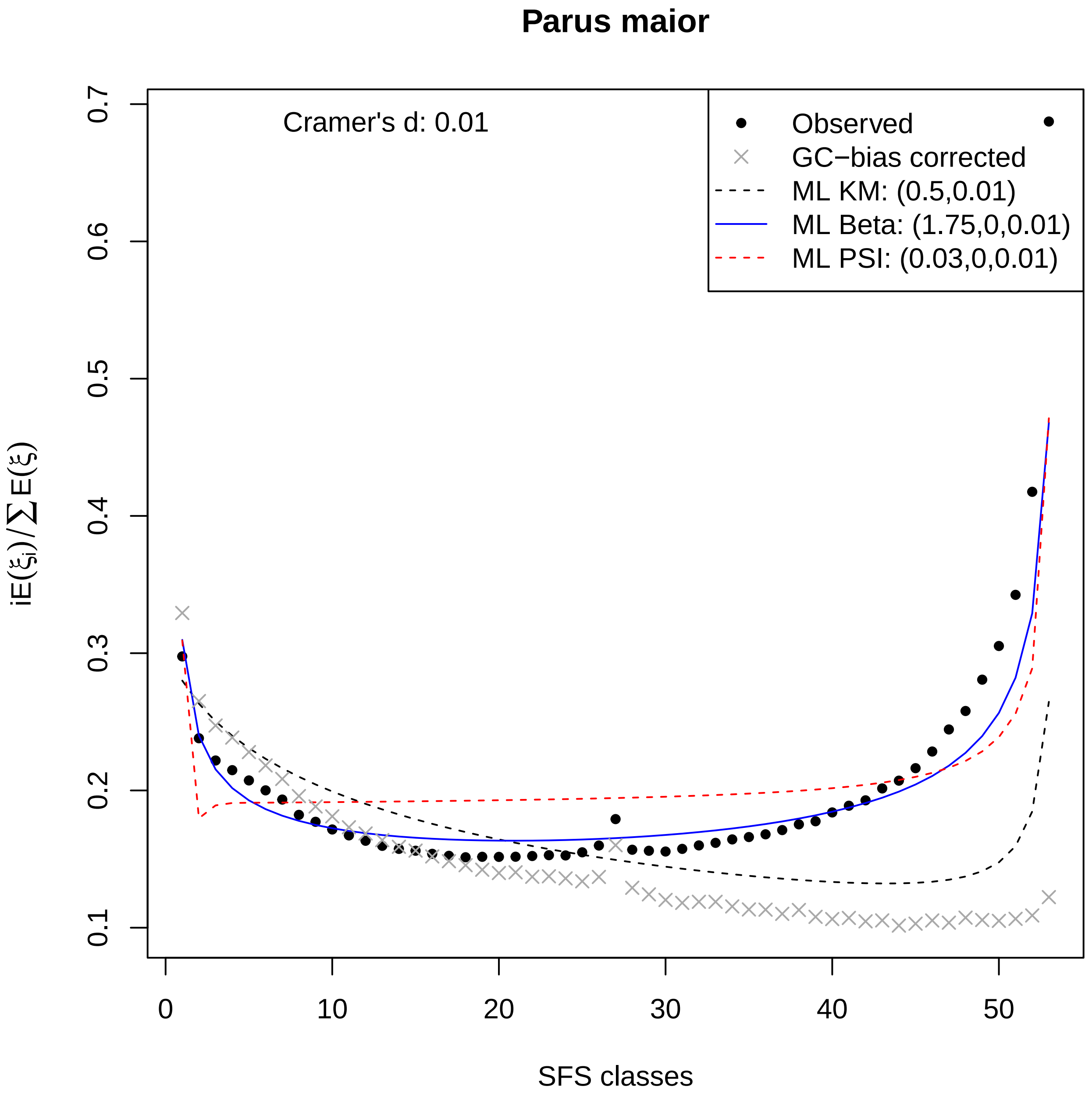

Parus maior
Cramer's d: 0.01
Observed
GC−bias corrected
ML KM: (0.5,0.01)
ML Beta: (1.75,0,0.01)
ML PSI: (0.03,0,0.01)
$iE(\xi_i)/\sum E(\xi)$
SFS classes
0.1
0.2
0.3
0.4
0.5
0.6
0.7
0
10
20
30
40
50

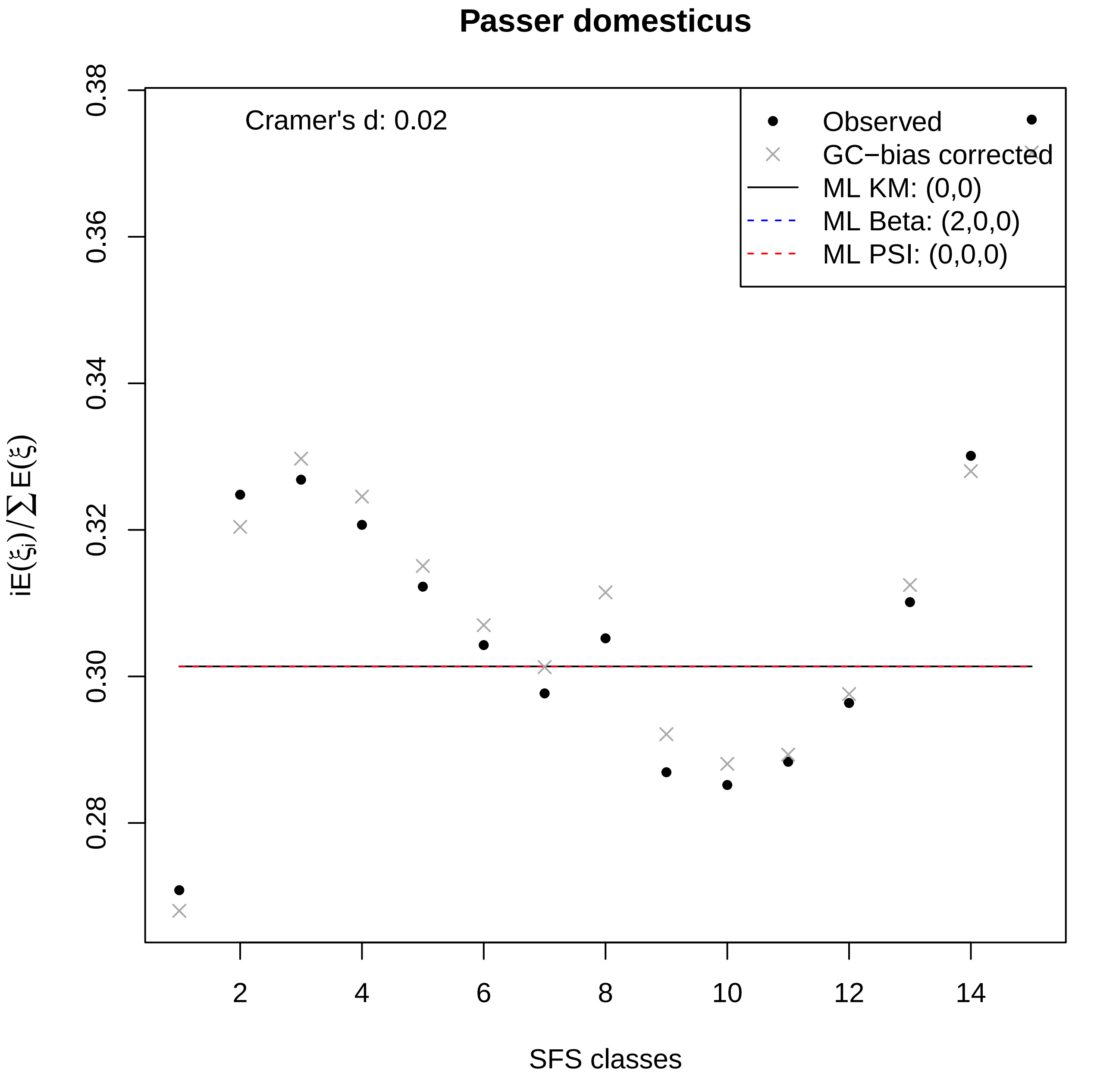

Passer domesticus
Cramer's d: 0.02
Observed
GC−bias corrected
ML KM: (0,0)
ML Beta: (2,0,0)
ML PSI: (0,0,0)
0.28
0.30
0.32
0.34
0.36
0.38
$iE(\xi_i)/\sum E(\xi)$
2
4
6
8
10
12
14
SFS classes

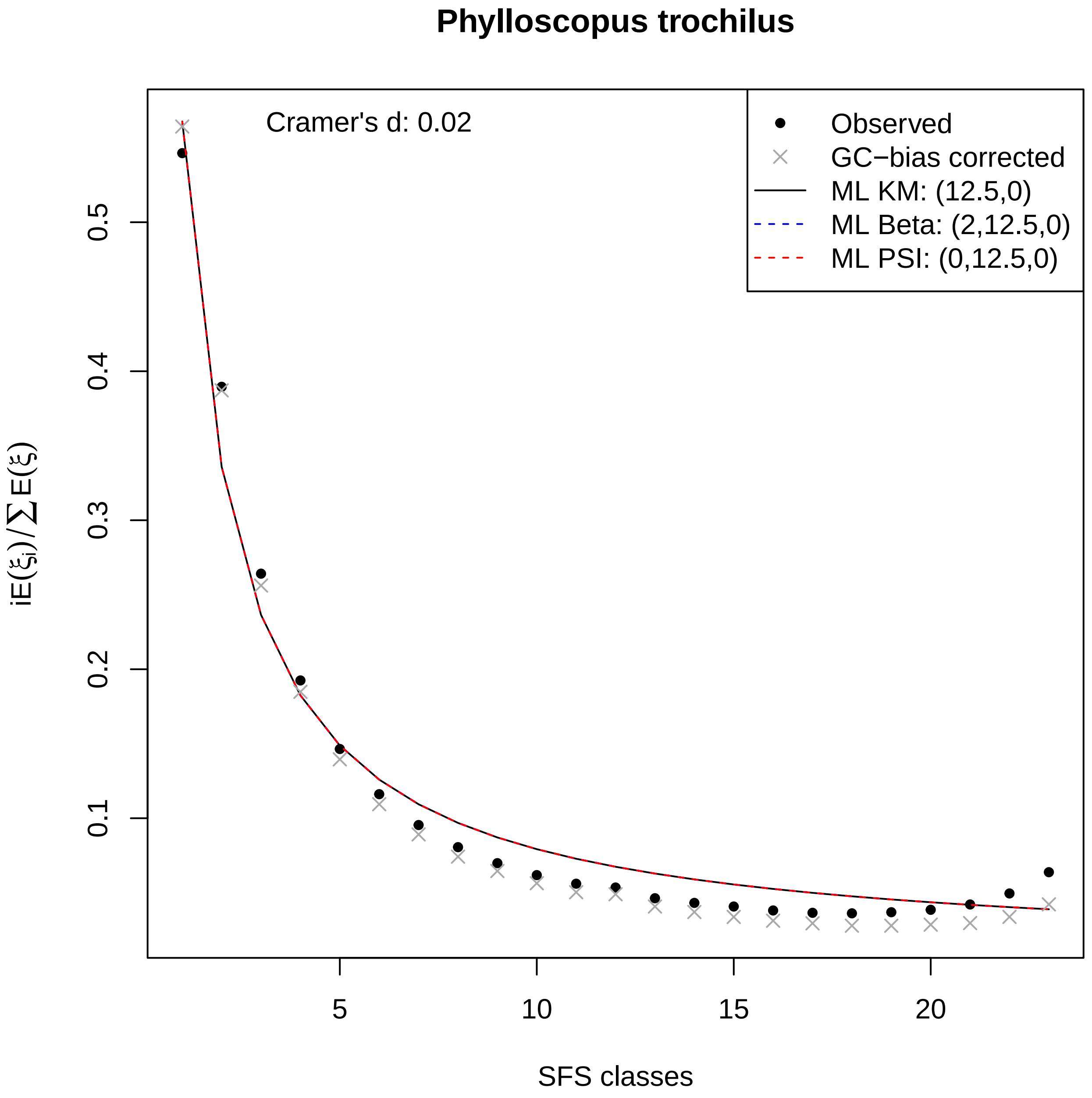

Phylloscopus trochilus
Cramer's d: 0.02
Observed
GC−bias corrected
ML KM: (12.5,0)
ML Beta: (2,12.5,0)
ML PSI: (0,12.5,0)
0.1
0.2
0.3
0.4
0.5
5
10
15
20
SFS classes
$iE(\xi_i)/\sum E(\xi)$

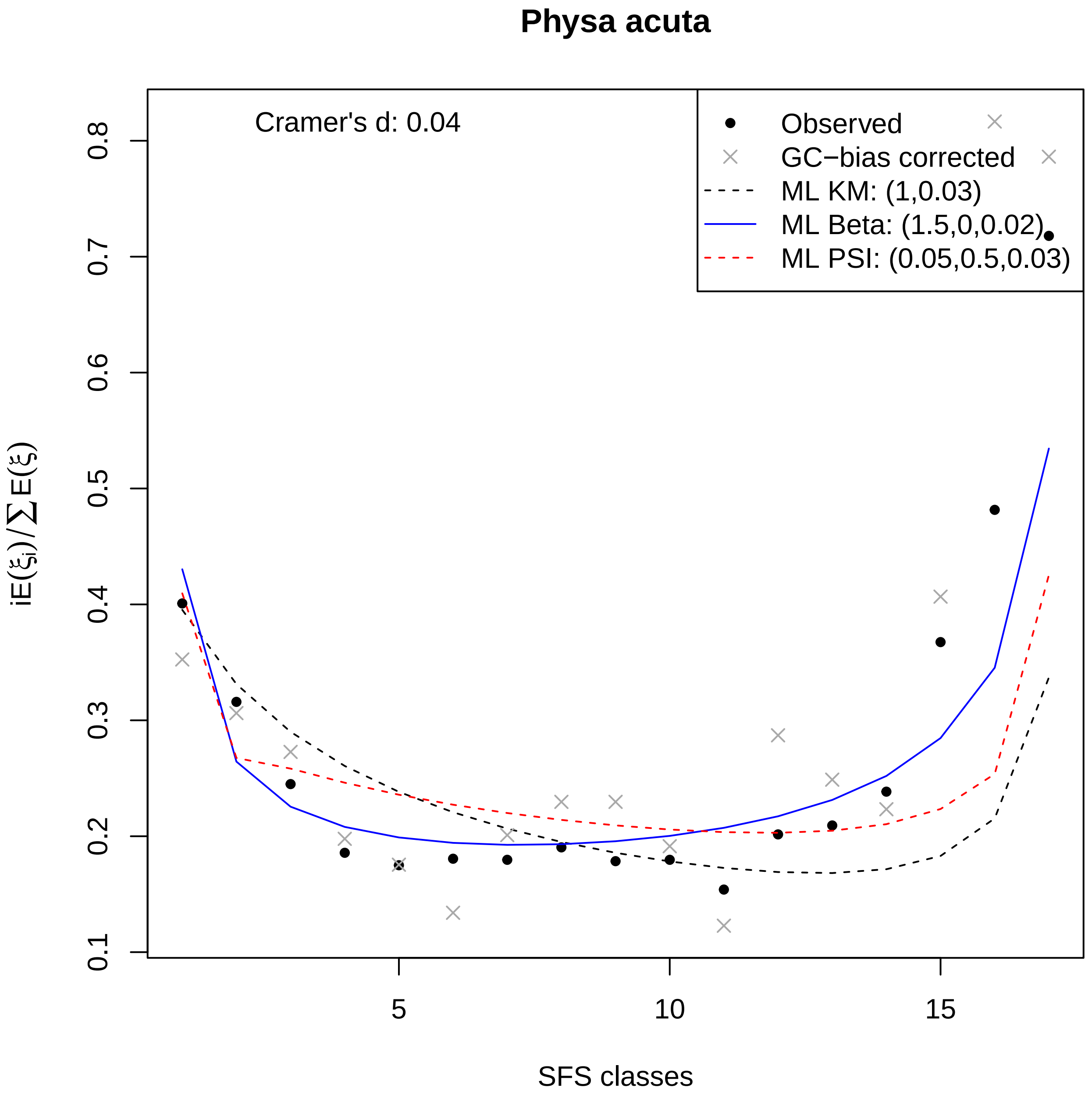

Physa acuta
Cramer's d: 0.04
Observed
GC−bias corrected
ML KM: (1,0.03)
ML Beta: (1.5,0,0.02)
ML PSI: (0.05,0.5,0.03)
0.1
0.2
0.3
0.4
0.5
0.6
0.7
0.8
5
10
15
iE(ξi)/∑E(ξ)
SFS classes

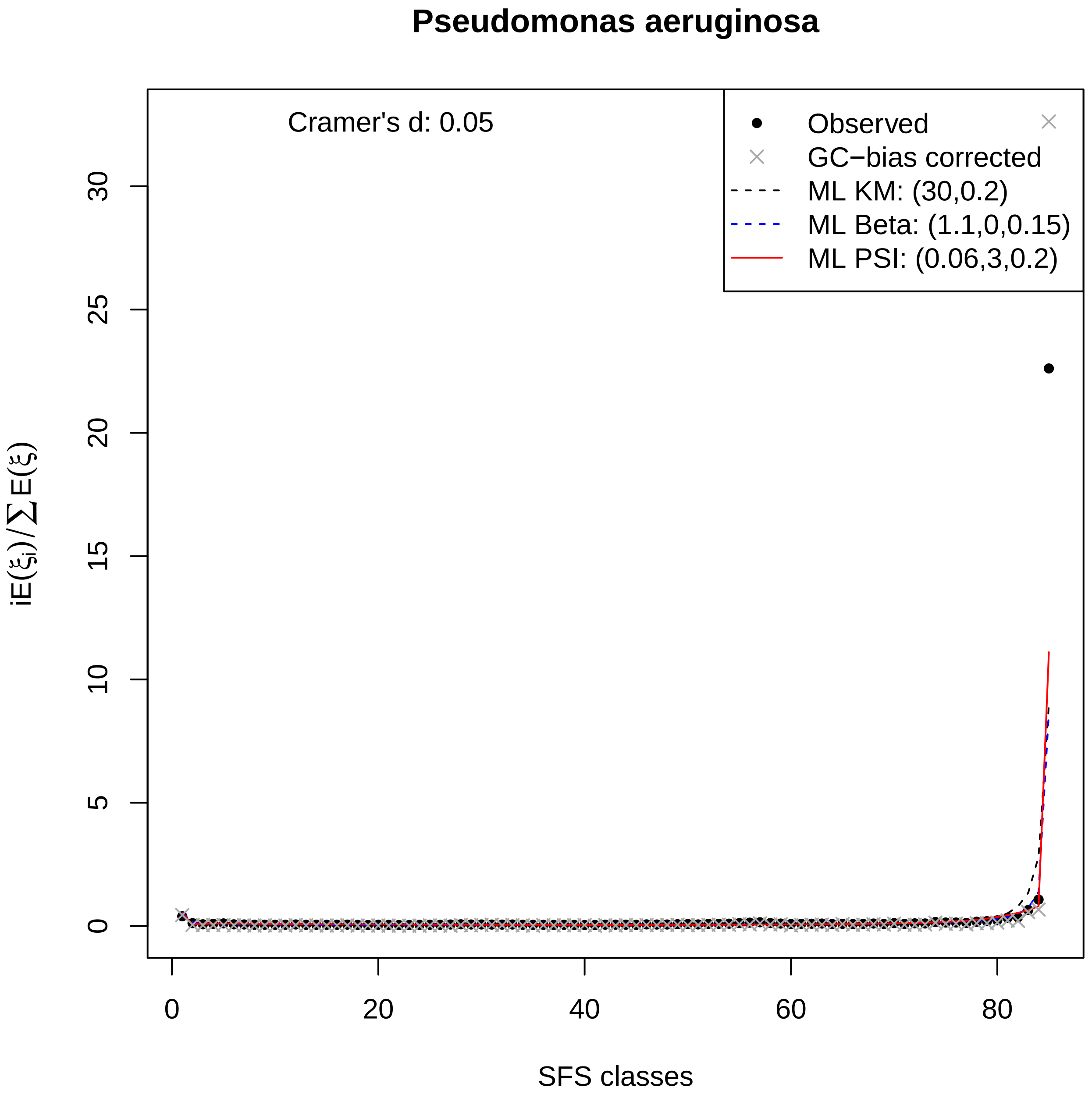
Pseudomonas aeruginosa
Cramer's d: 0.05
Observed
GC−bias corrected
ML KM: (30,0.2)
ML Beta: (1.1,0,0.15)
ML PSI: (0.06,3,0.2)
iE(ξi)/∑E(ξ)
SFS classes
0
5
10
15
20
25
30
0
20
40
60
80

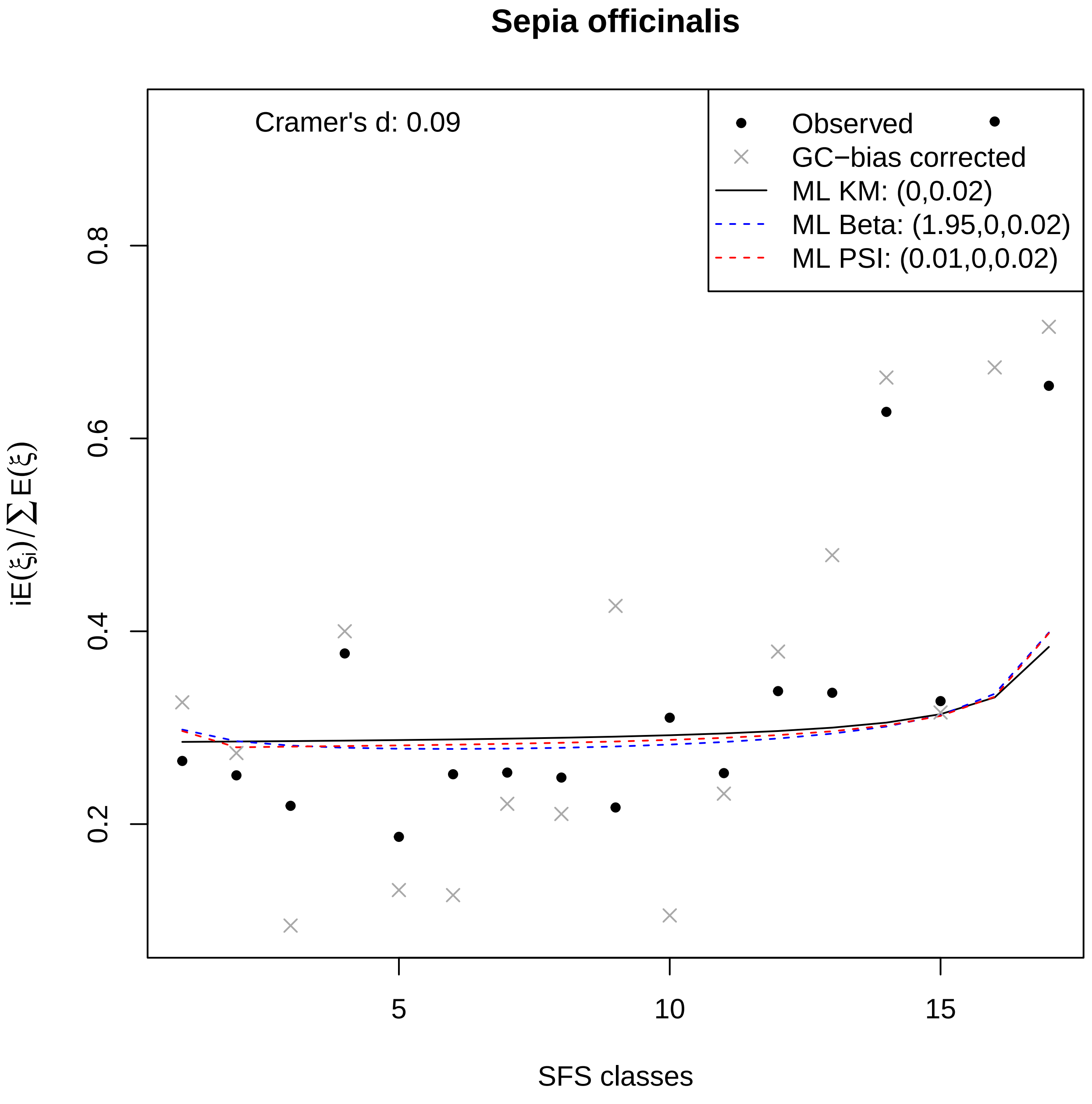

Sepia officinalis
Cramer's d: 0.09
Observed
GC−bias corrected
ML KM: (0,0.02)
ML Beta: (1.95,0,0.02)
ML PSI: (0.01,0,0.02)
0.2
0.4
0.6
0.8
5
10
15
SFS classes
iE(ξi)/∑E(ξ)

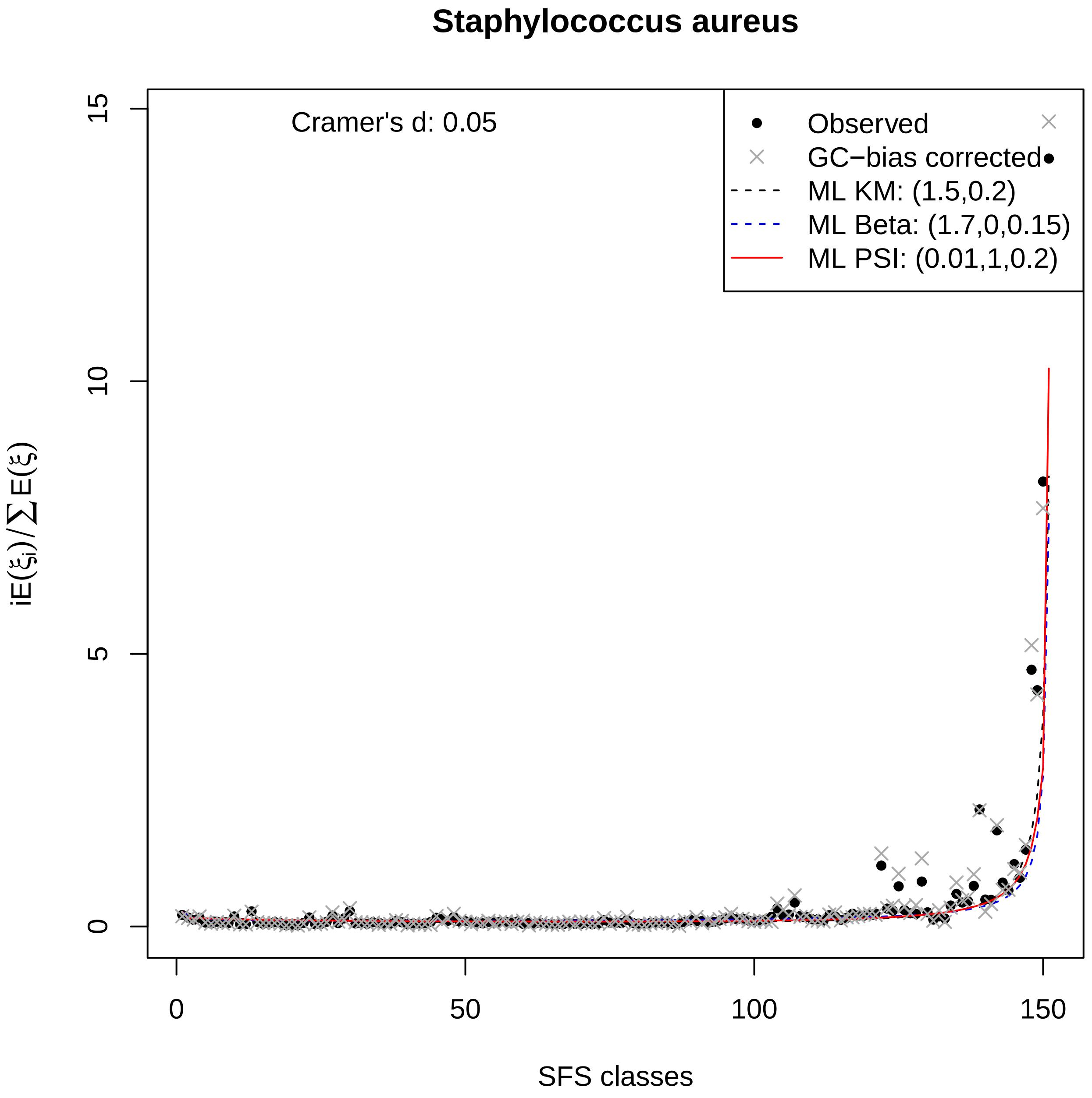

Staphylococcus aureus
Cramer's d: 0.05
Observed
GC−bias corrected
ML KM: (1.5,0.2)
ML Beta: (1.7,0,0.15)
ML PSI: (0.01,1,0.2)
0
5
10
15
0
50
100
150
SFS classes
iE(ξi)/∑E(ξ)

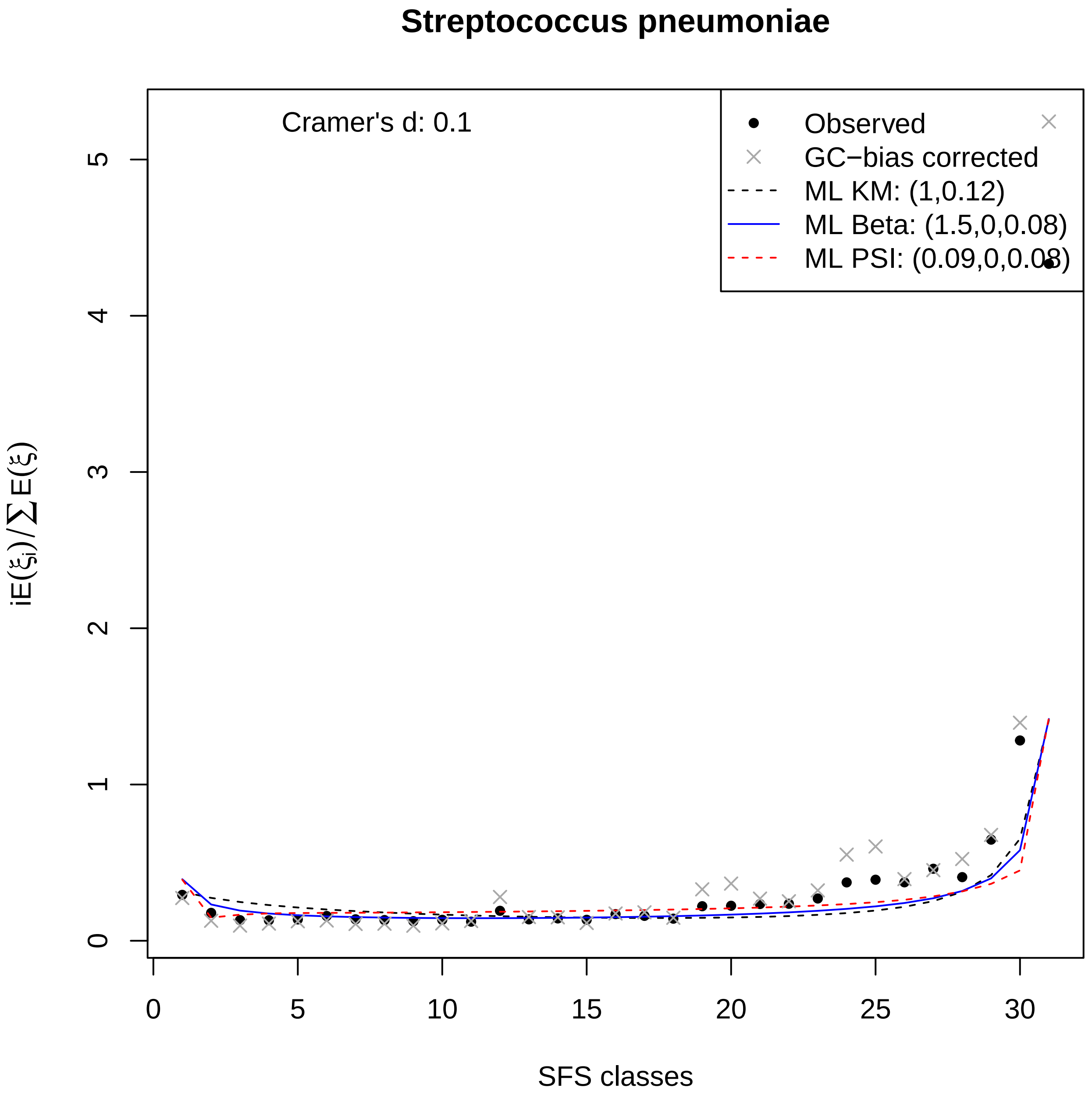
Streptococcus pneumoniae
Cramer's d: 0.1
Observed
GC−bias corrected
ML KM: (1,0.12)
ML Beta: (1.5,0,0.08)
ML PSI: (0.09,0,0.08)
$iE(\xi_i)/\sum E(\xi)$
0
1
2
3
4
5
0
5
10
15
20
25
30
SFS classes

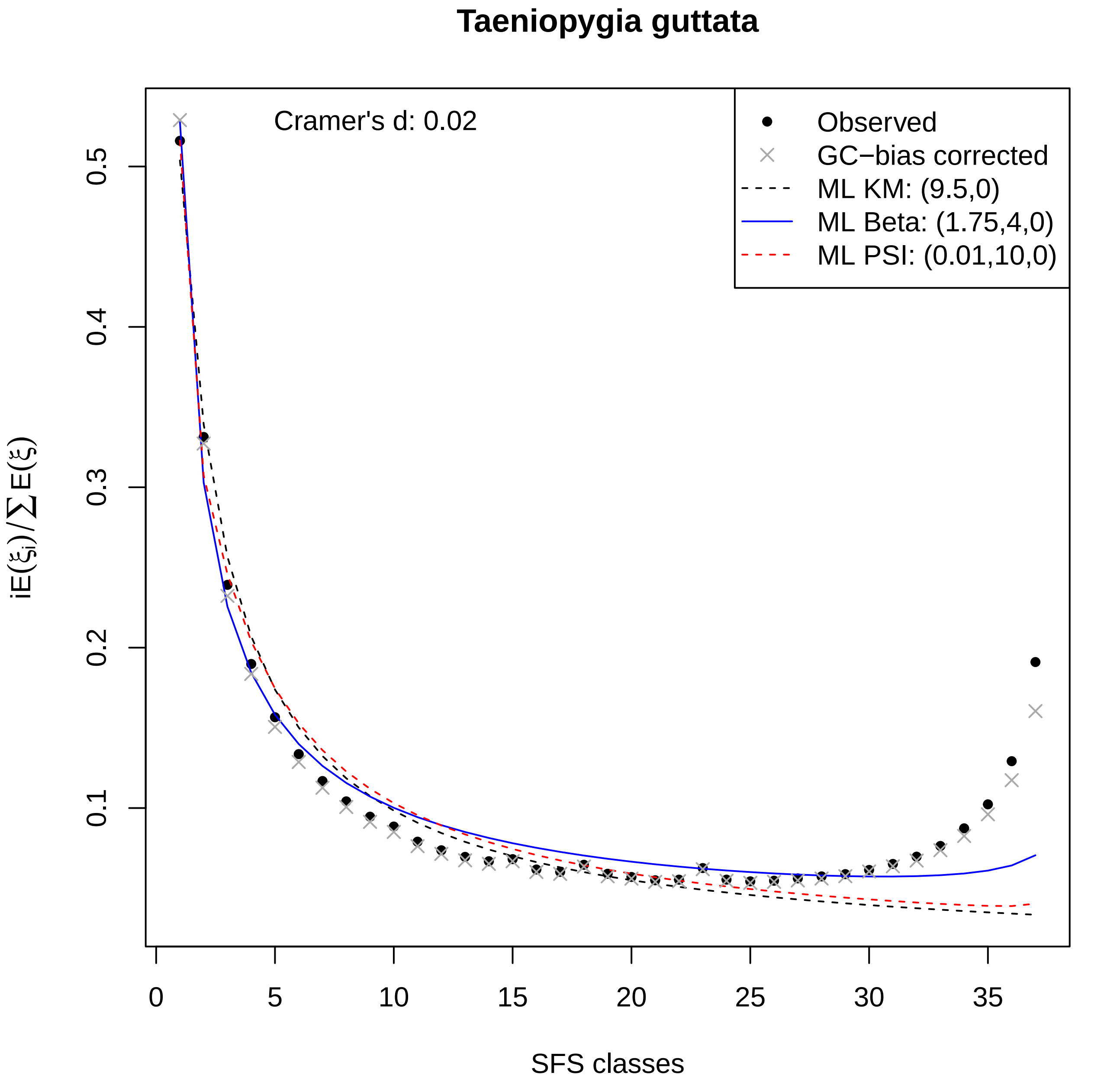
Taeniopygia guttata
Cramer's d: 0.02
Observed
GC−bias corrected
ML KM: (9.5,0)
ML Beta: (1.75,4,0)
ML PSI: (0.01,10,0)
0.1
0.2
0.3
0.4
0.5
$iE(\xi_i)/\sum E(\xi)$
0
5
10
15
20
25
30
35
SFS classes

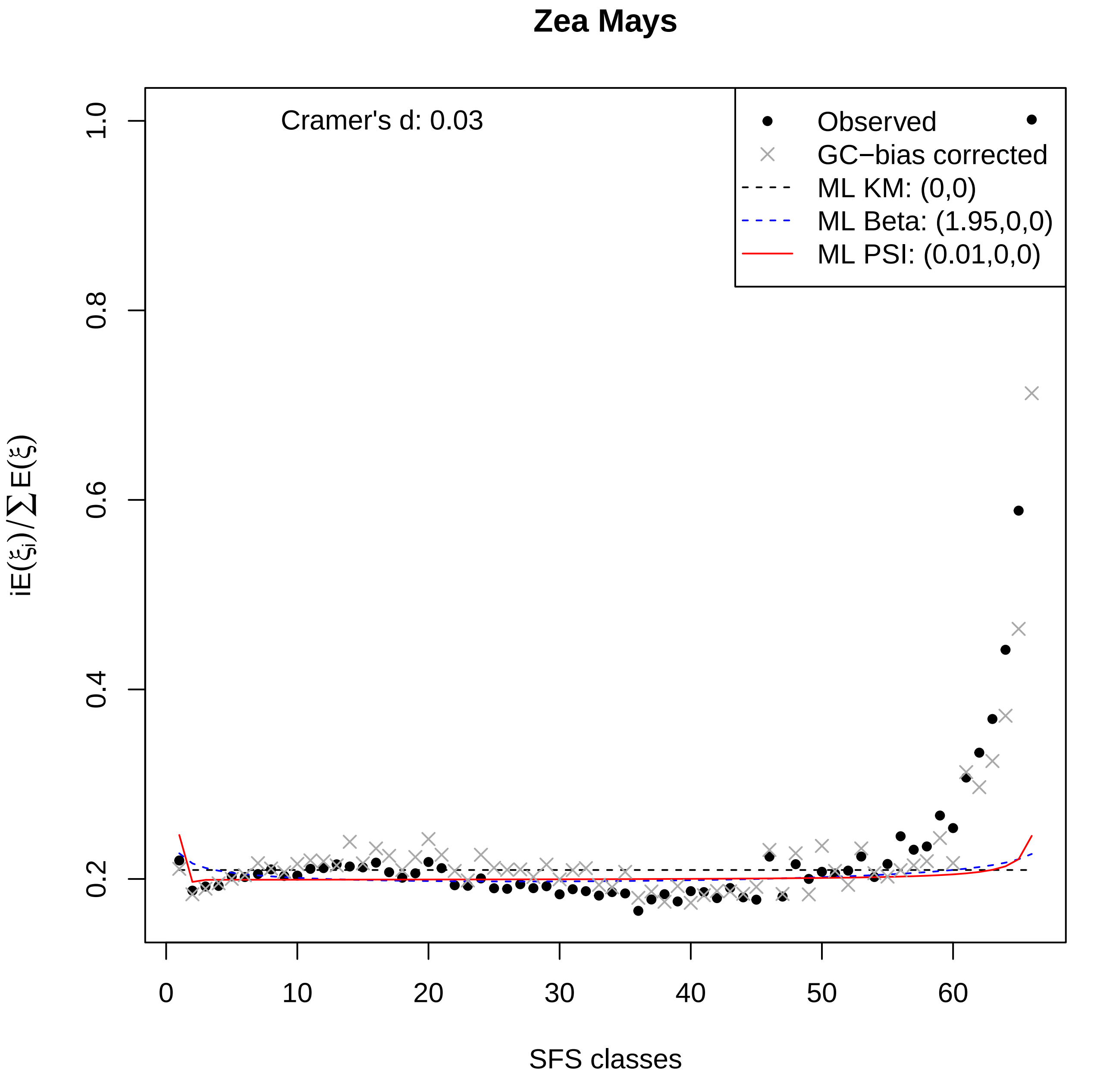
Zea Mays
Cramer's d: 0.03
Observed
GC−bias corrected
ML KM: (0,0)
ML Beta: (1.95,0,0)
ML PSI: (0.01,0,0)
1.0
0.8
0.6
0.4
0.2
0
10
20
30
40
50
60
SFS classes
$iE(\xi_i)/\sum E(\xi)$
