# Supplementary material for: Interpreting the pervasive observation of U-shaped Site Frequency Spectra
Source: PLoS Genet. 2023 Mar 23;19(3):e1010677. doi: 10.1371/journal.pgen.1010677 (PMC10072462; doi:10.1371/journal.pgen.1010677)
Supplement: S3 Fig — (PDF) [file pgen.1010677.s004.pdf]

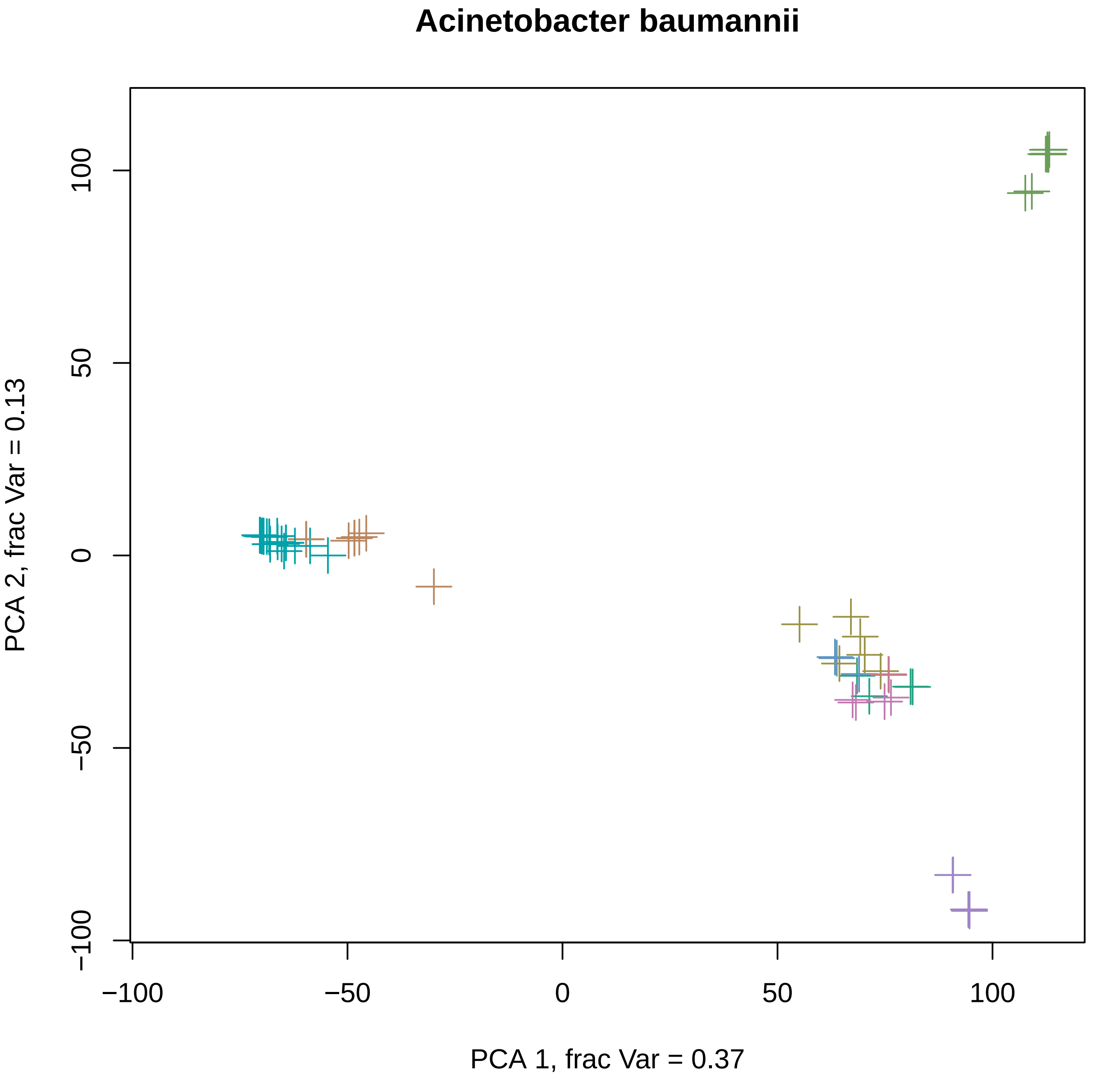

Acinetobacter baumannii
100
50
0
−50
−100
−100
−50
0
50
100
PCA 1, frac Var = 0.37
PCA 2, frac Var = 0.13

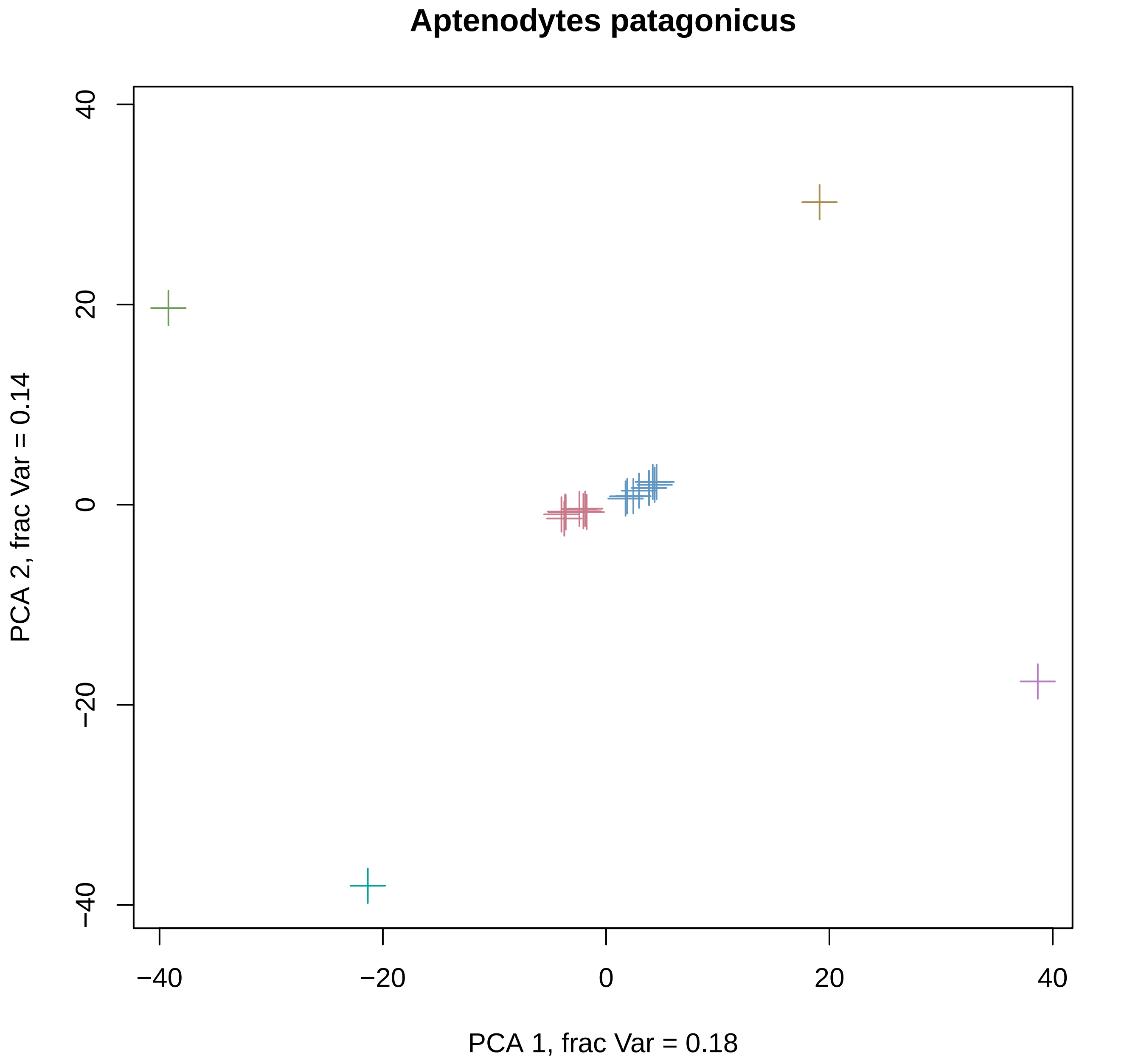

Aptenodytes patagonicus
PCA 2, frac Var = 0.14
40
20
0
−20
−40
−40
−20
0
20
40
PCA 1, frac Var = 0.18

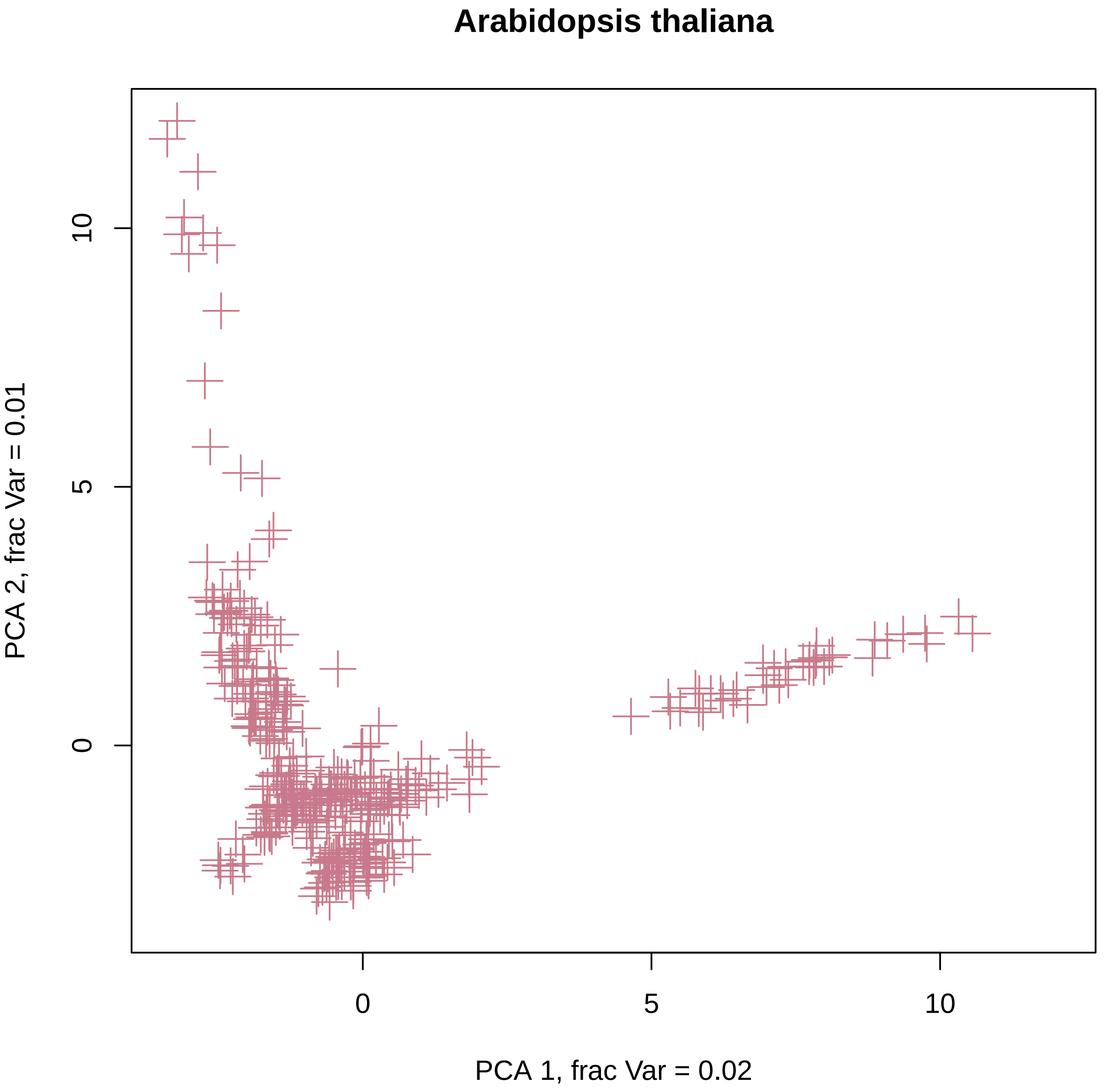
Arabidopsis thaliana
PCA 2, frac Var = 0.01
PCA 1, frac Var = 0.02
0
5
10
0
5
10

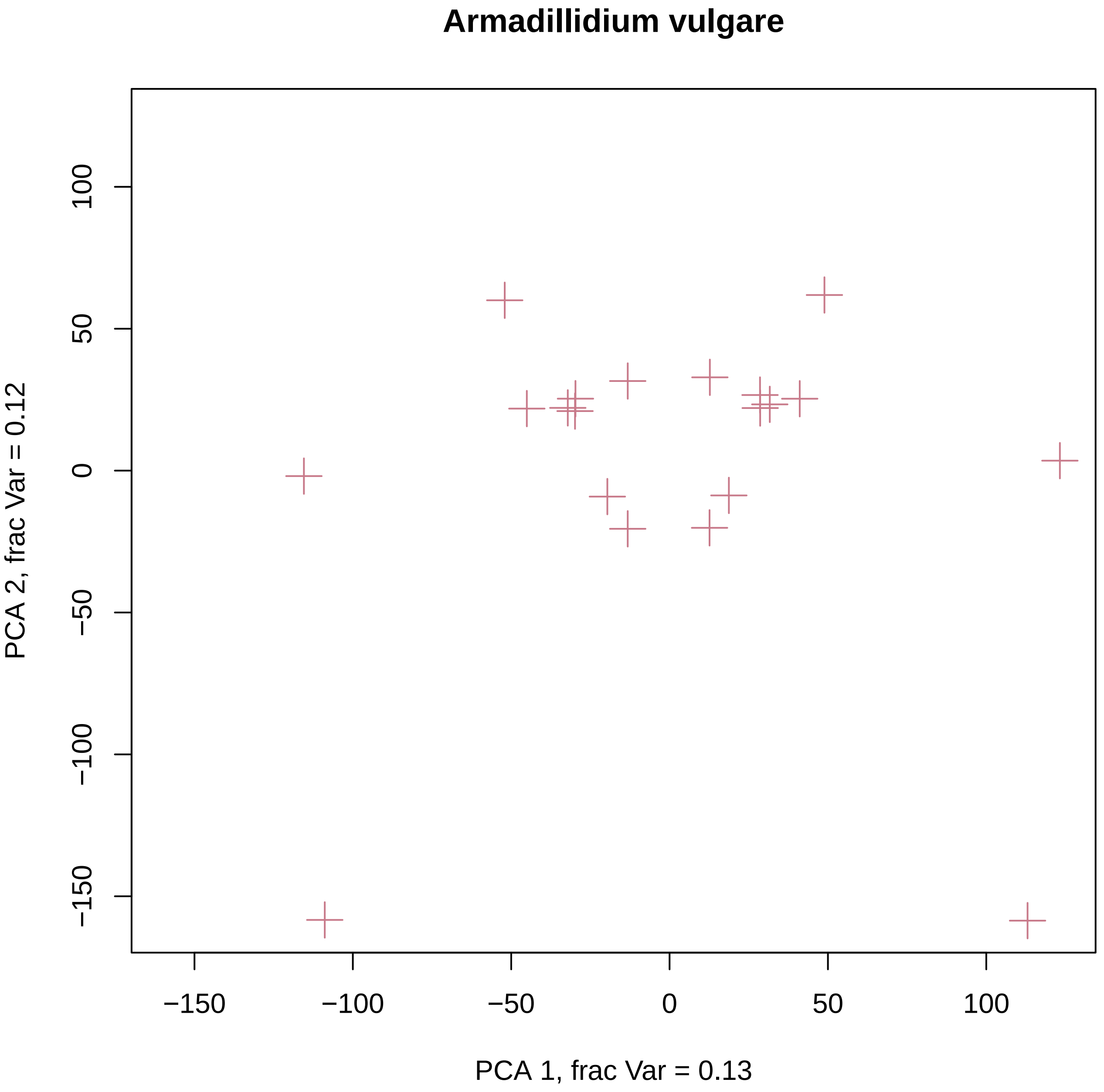

Armadillidium vulgare
PCA 1, frac Var = 0.13
PCA 2, frac Var = 0.12
−150
−100
−50
0
50
100

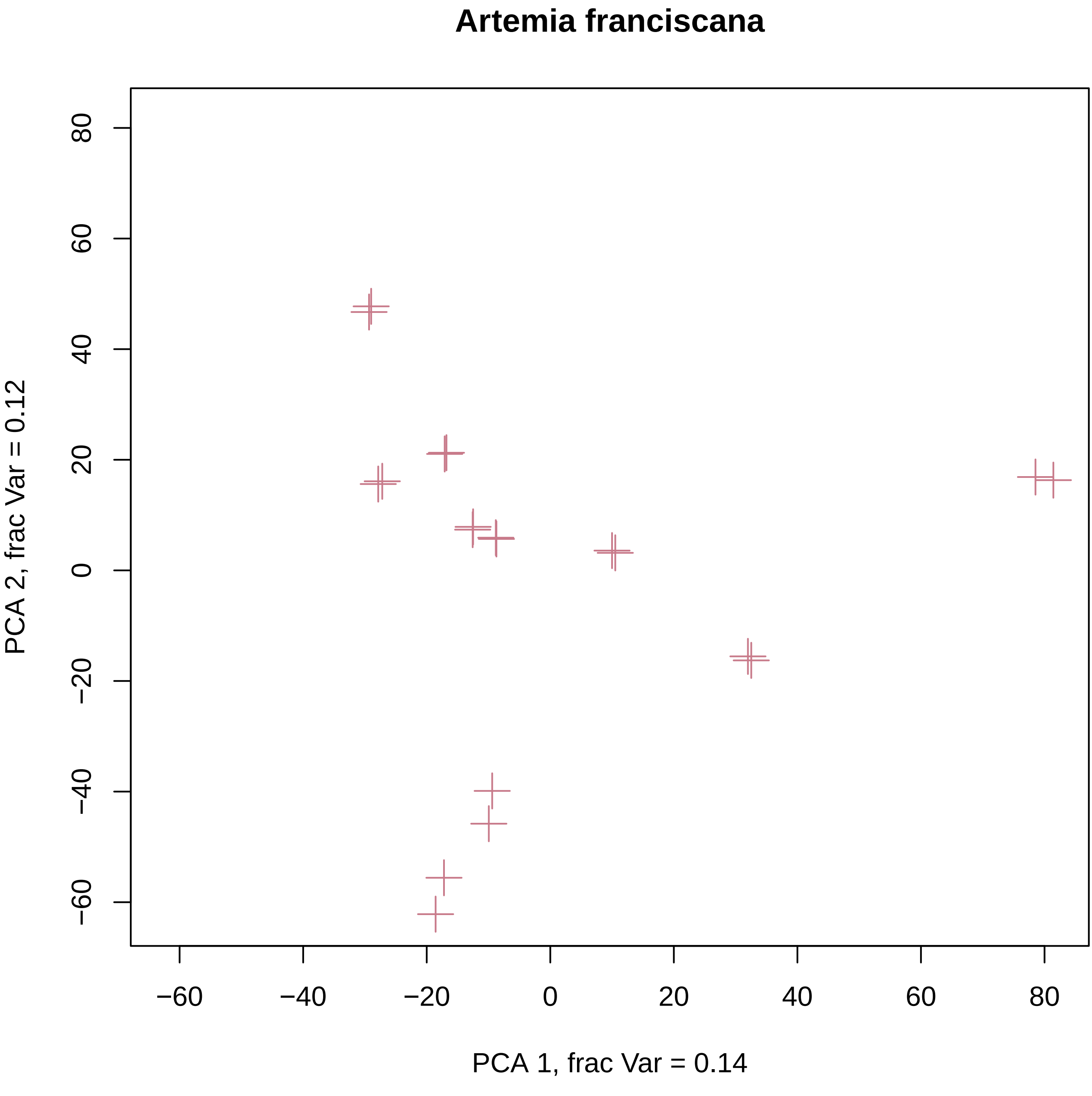
Artemia franciscana
PCA 1, frac Var = 0.14
PCA 2, frac Var = 0.12
−60
−40
−20
0
20
40
60
80

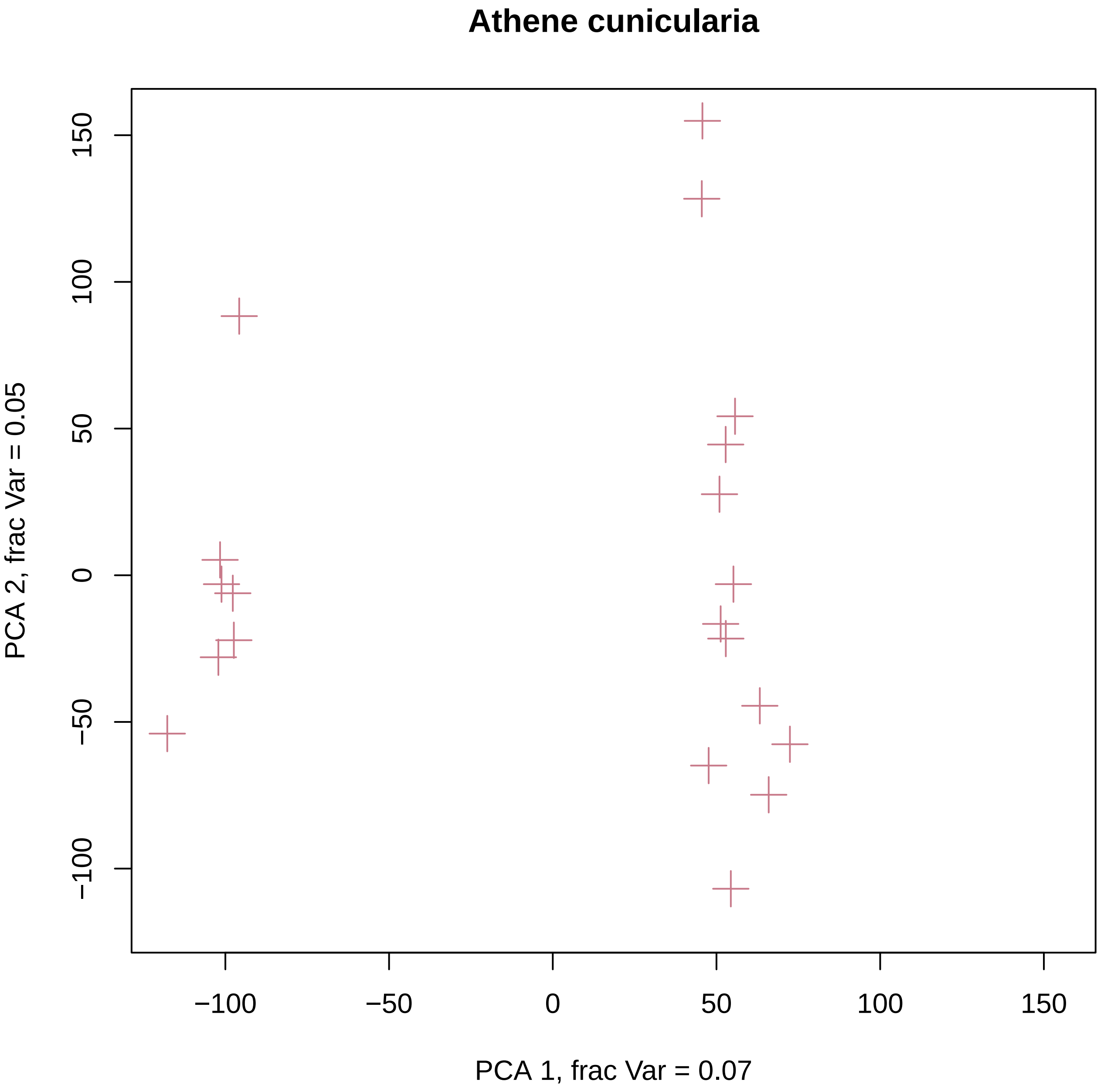

Athene cunicularia
PCA 1, frac Var = 0.07
PCA 2, frac Var = 0.05
−100
−50
0
50
100
150

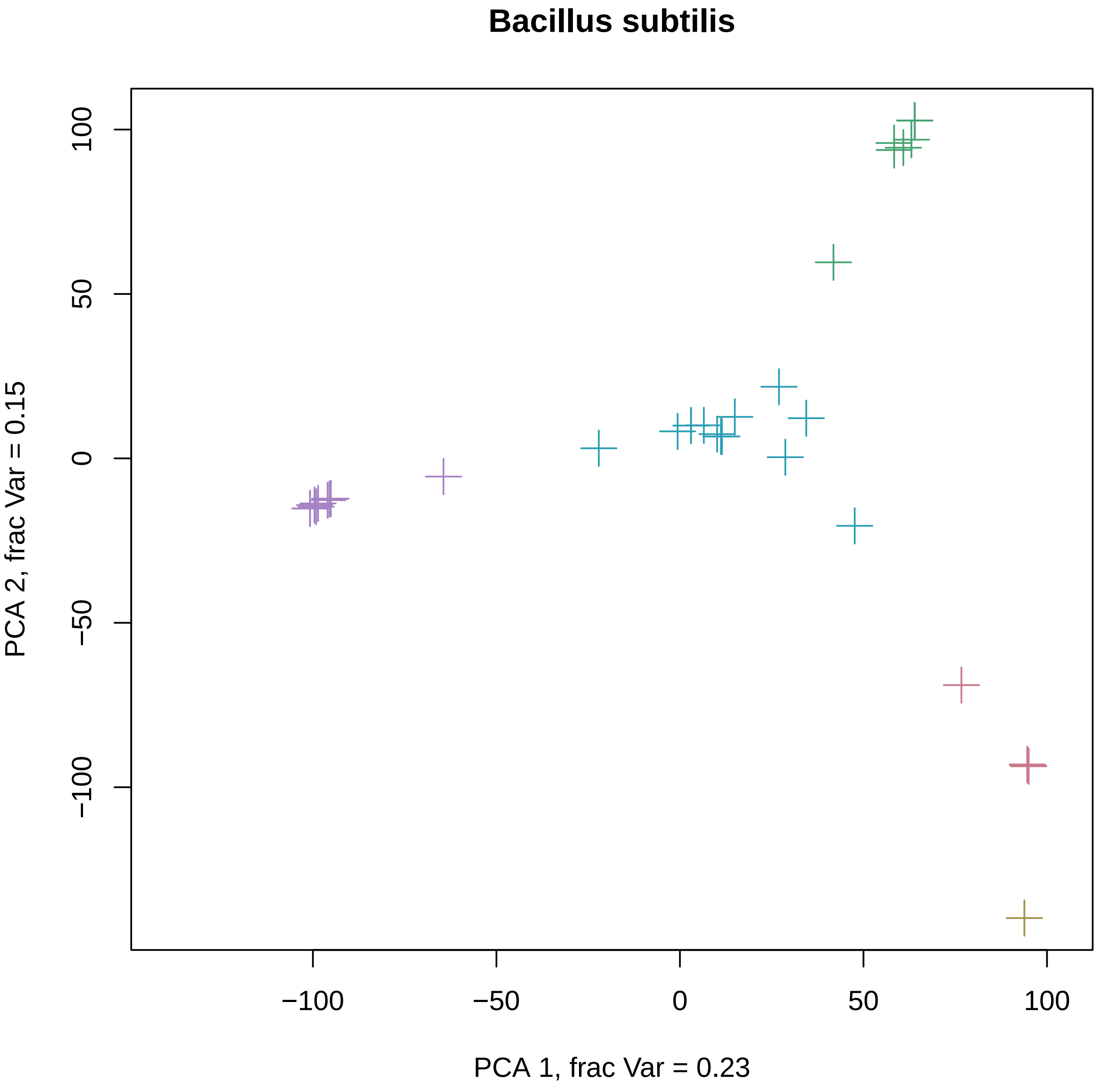
Bacillus subtilis
PCA 1, frac Var = 0.23
PCA 2, frac Var = 0.15
−100
−50
0
50
100

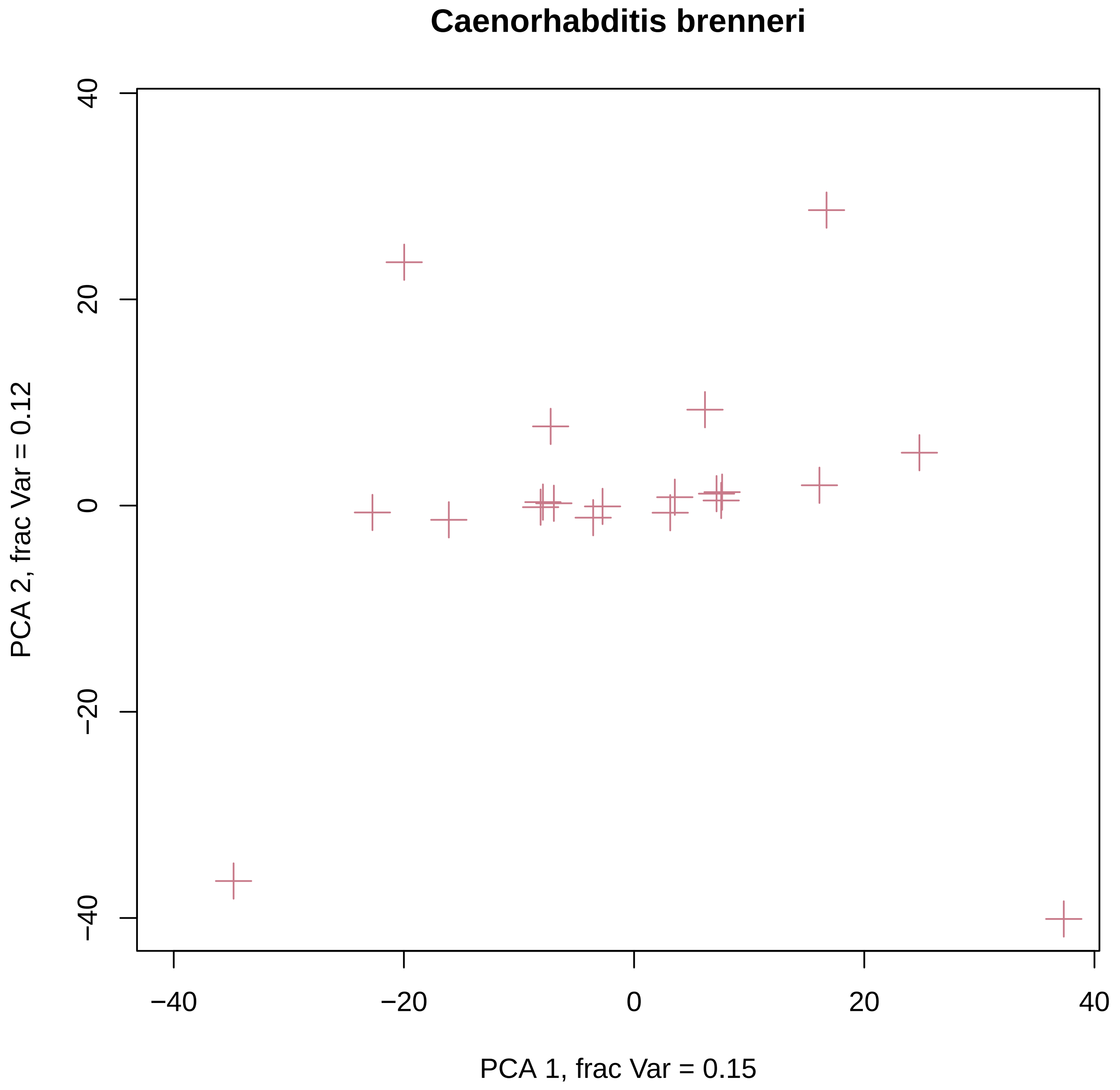
Caenorhabditis brenneri
PCA 2, frac Var = 0.12
PCA 1, frac Var = 0.15
40
20
0
−20
−40
−40
−20
0
20
40

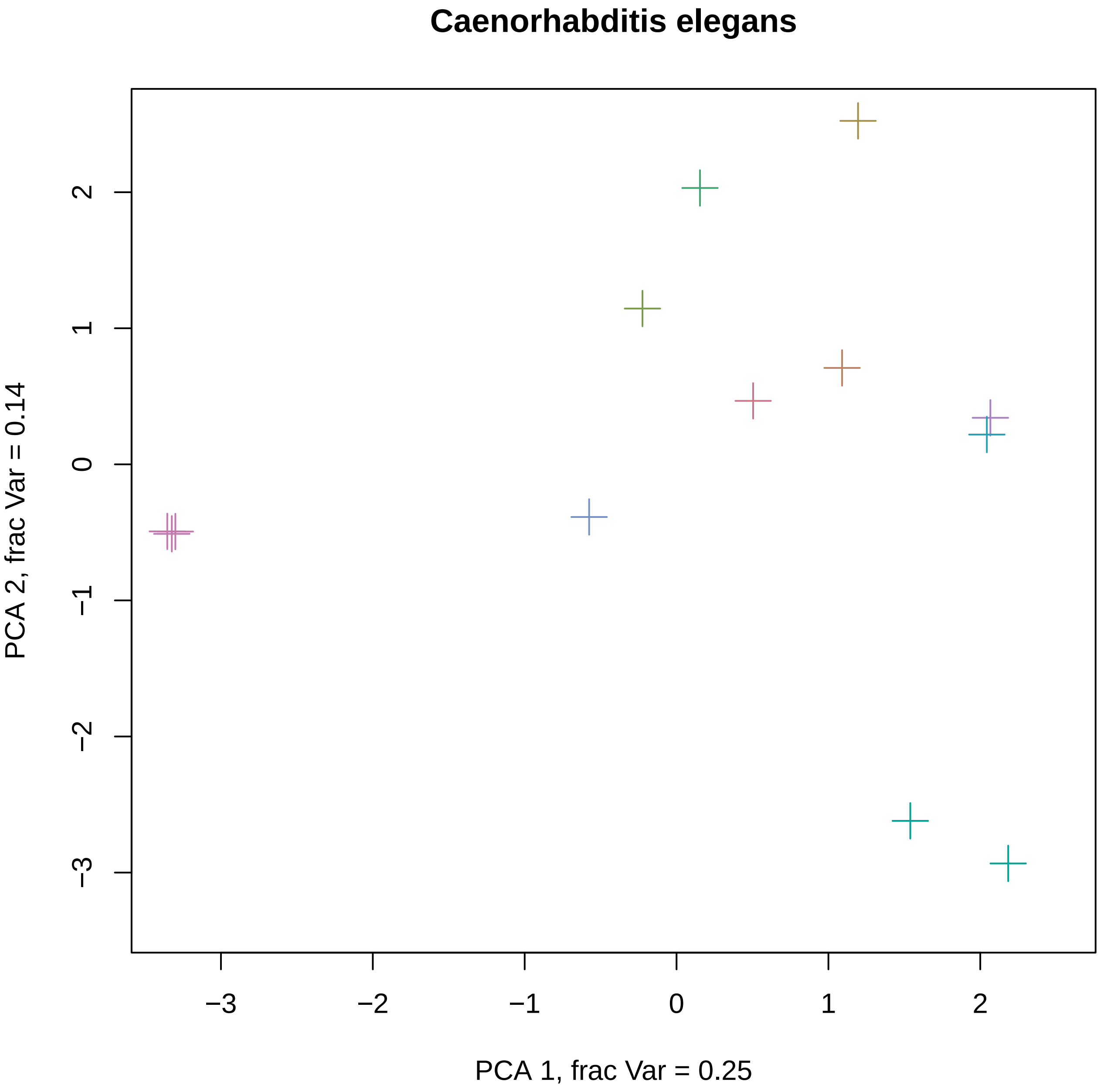

Caenorhabditis elegans
PCA 1, frac Var = 0.25
PCA 2, frac Var = 0.14
−3
−2
−1
0
1
2

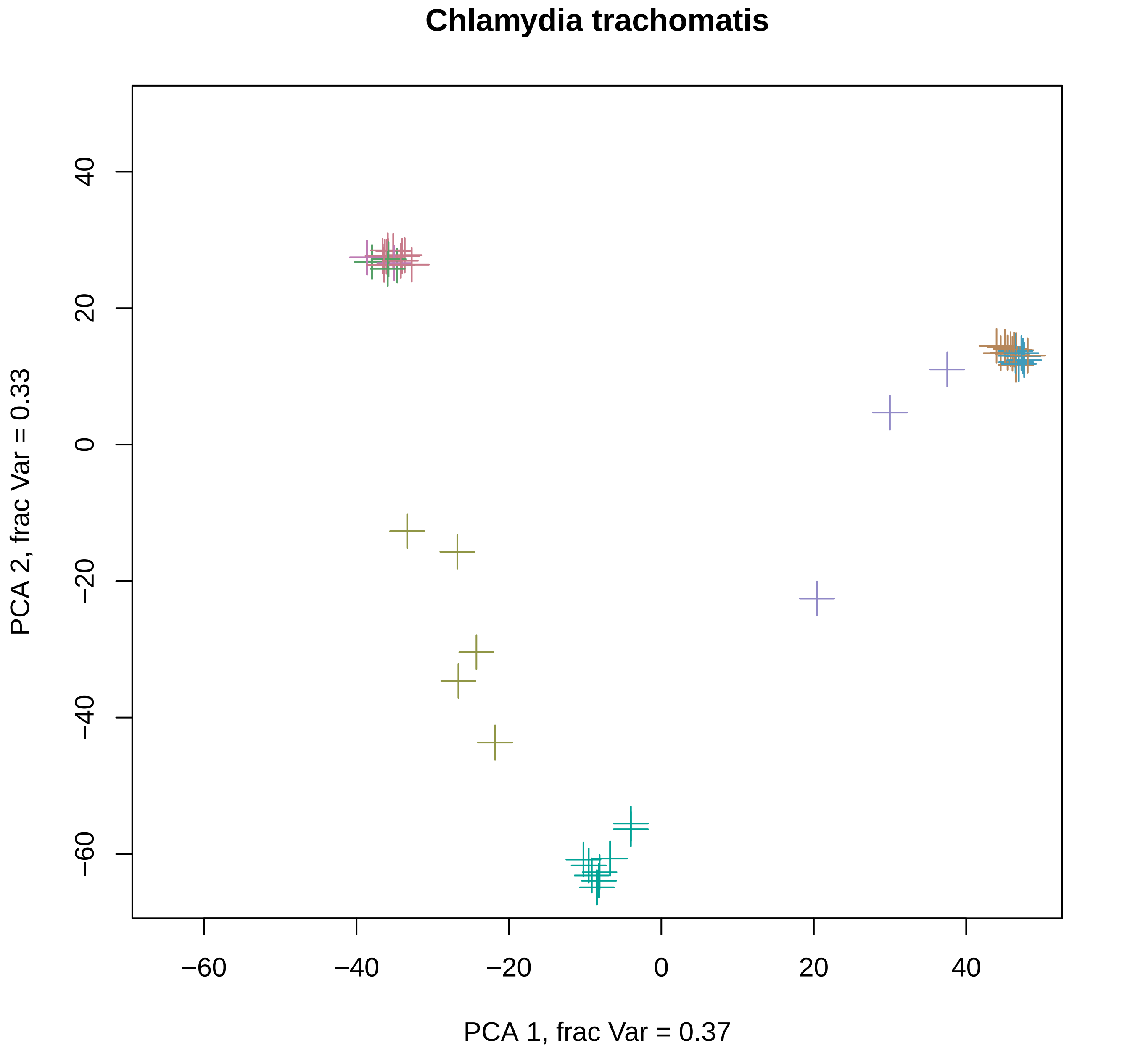
Chlamydia trachomatis
PCA 1, frac Var = 0.37
PCA 2, frac Var = 0.33
−60
−40
−20
0
20
40
−60
−40
−20
0
20
40

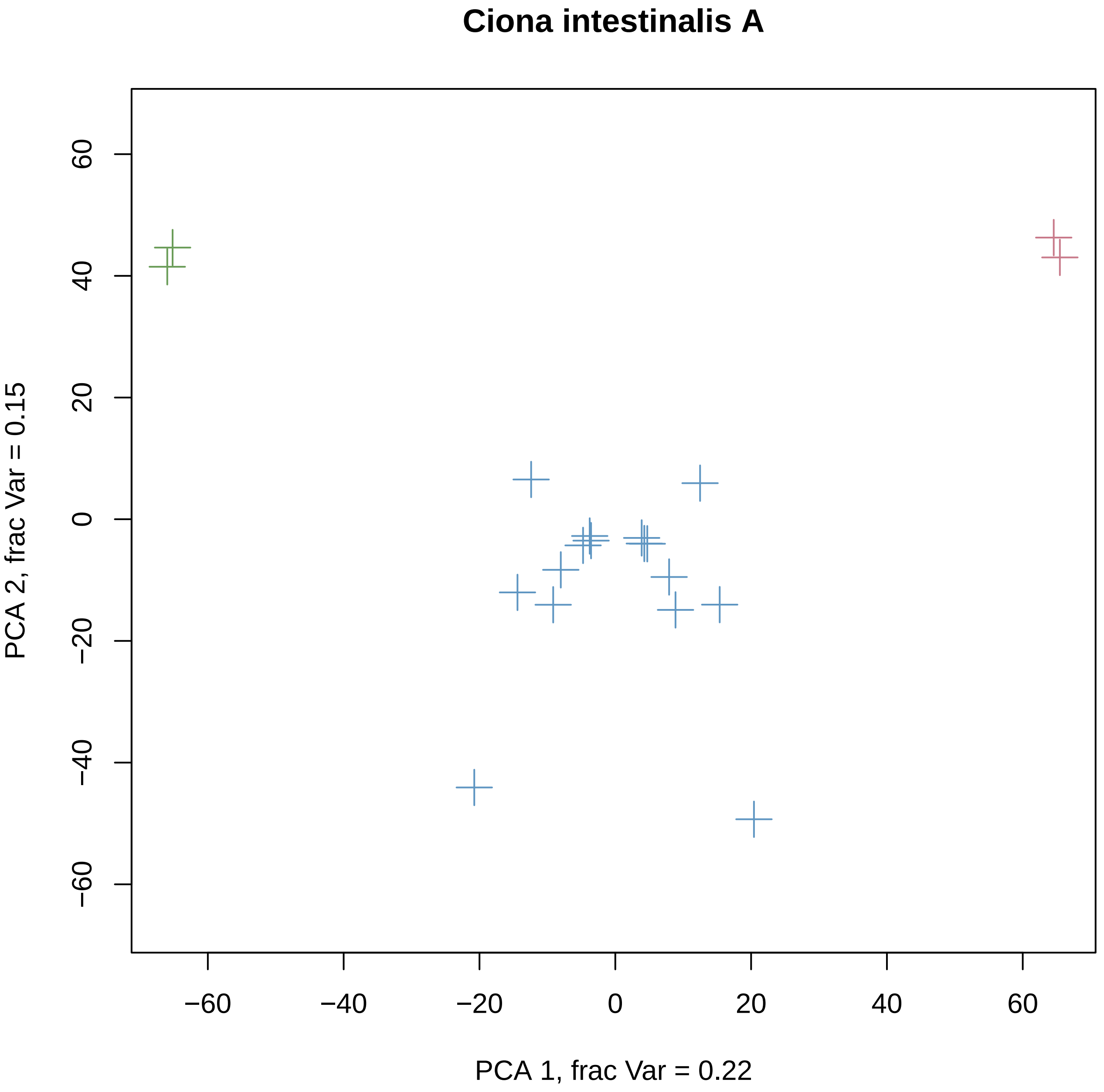

Ciona intestinalis A
PCA 1, frac Var = 0.22
PCA 2, frac Var = 0.15
−60
−40
−20
0
20
40
60

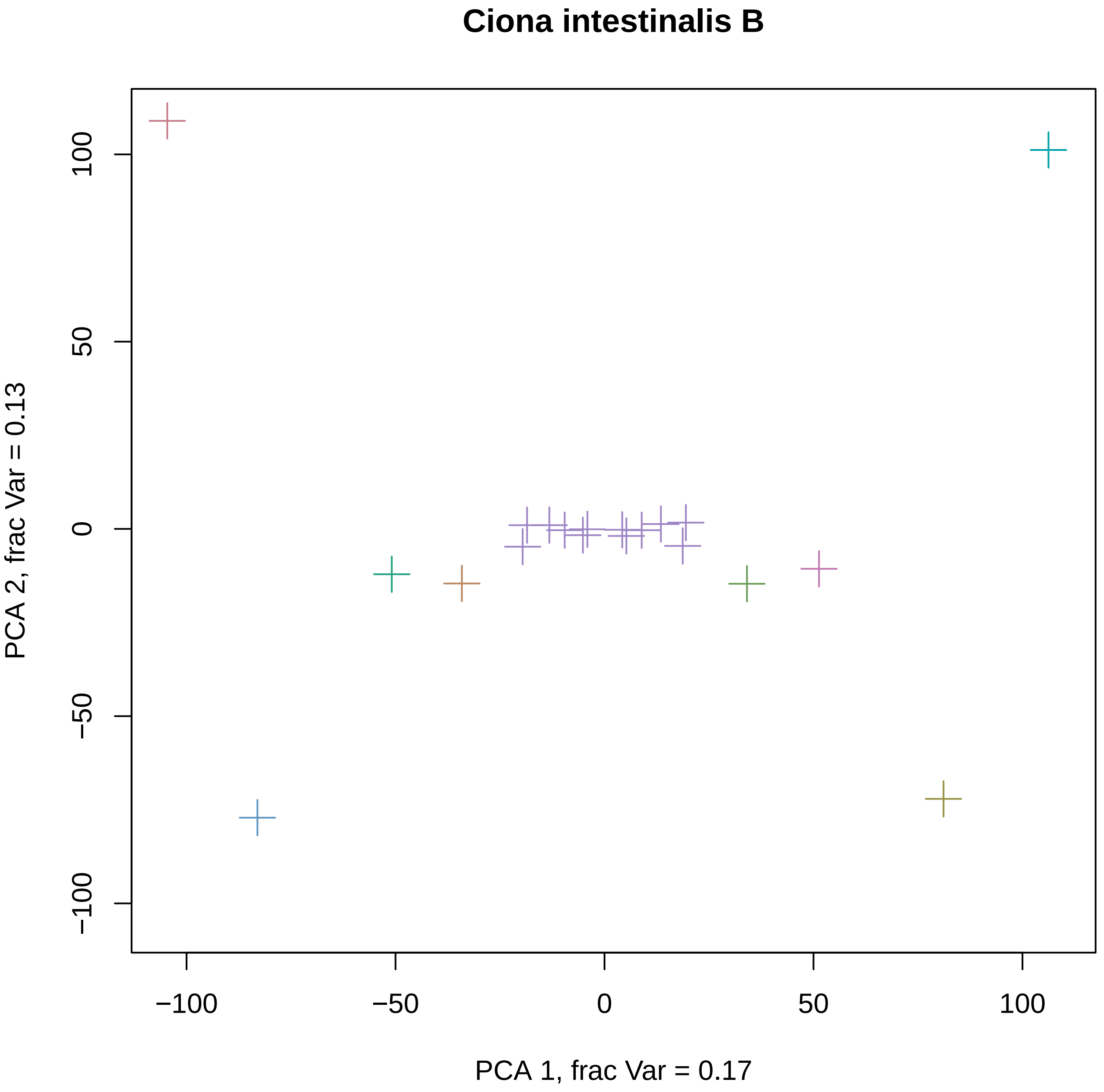
Ciona intestinalis B
PCA 2, frac Var = 0.13
PCA 1, frac Var = 0.17
100
50
0
−50
−100
−100
−50
0
50
100

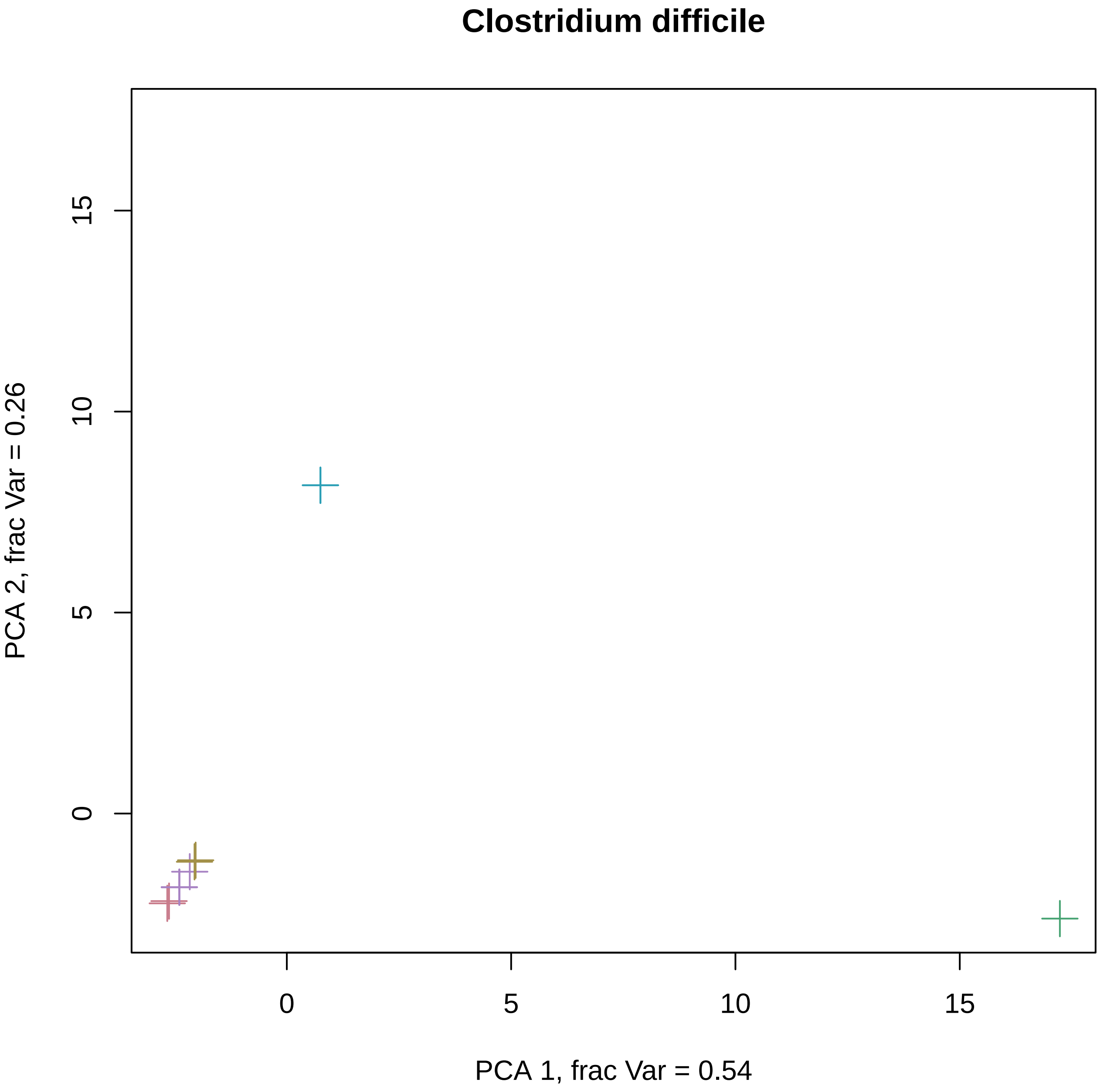

Clostridium difficile
PCA 1, frac Var = 0.54
PCA 2, frac Var = 0.26
0
5
10
15
0
5
10
15

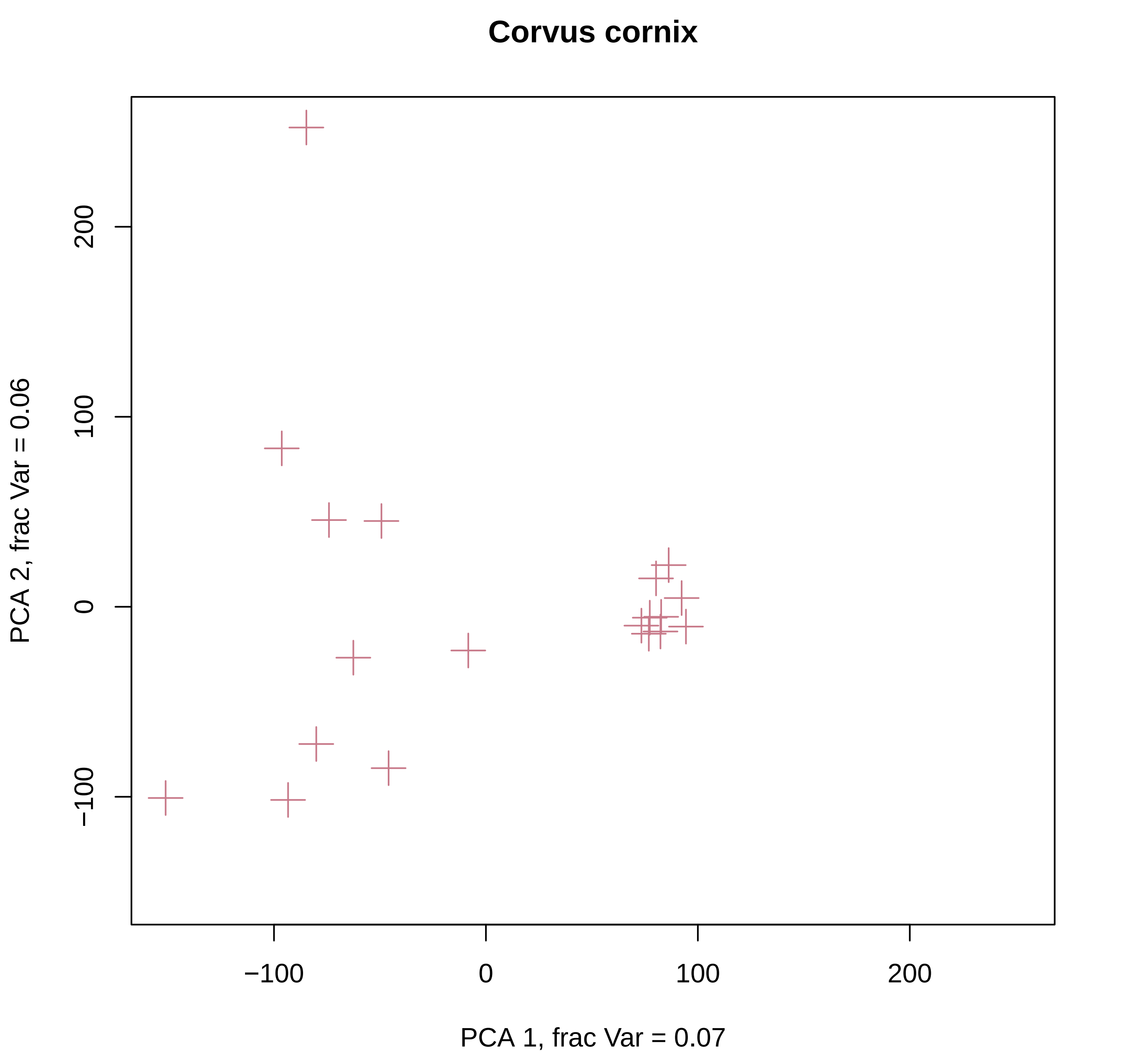
Corvus cornix
PCA 1, frac Var = 0.07
PCA 2, frac Var = 0.06
−100
0
100
200
−100
0
100
200

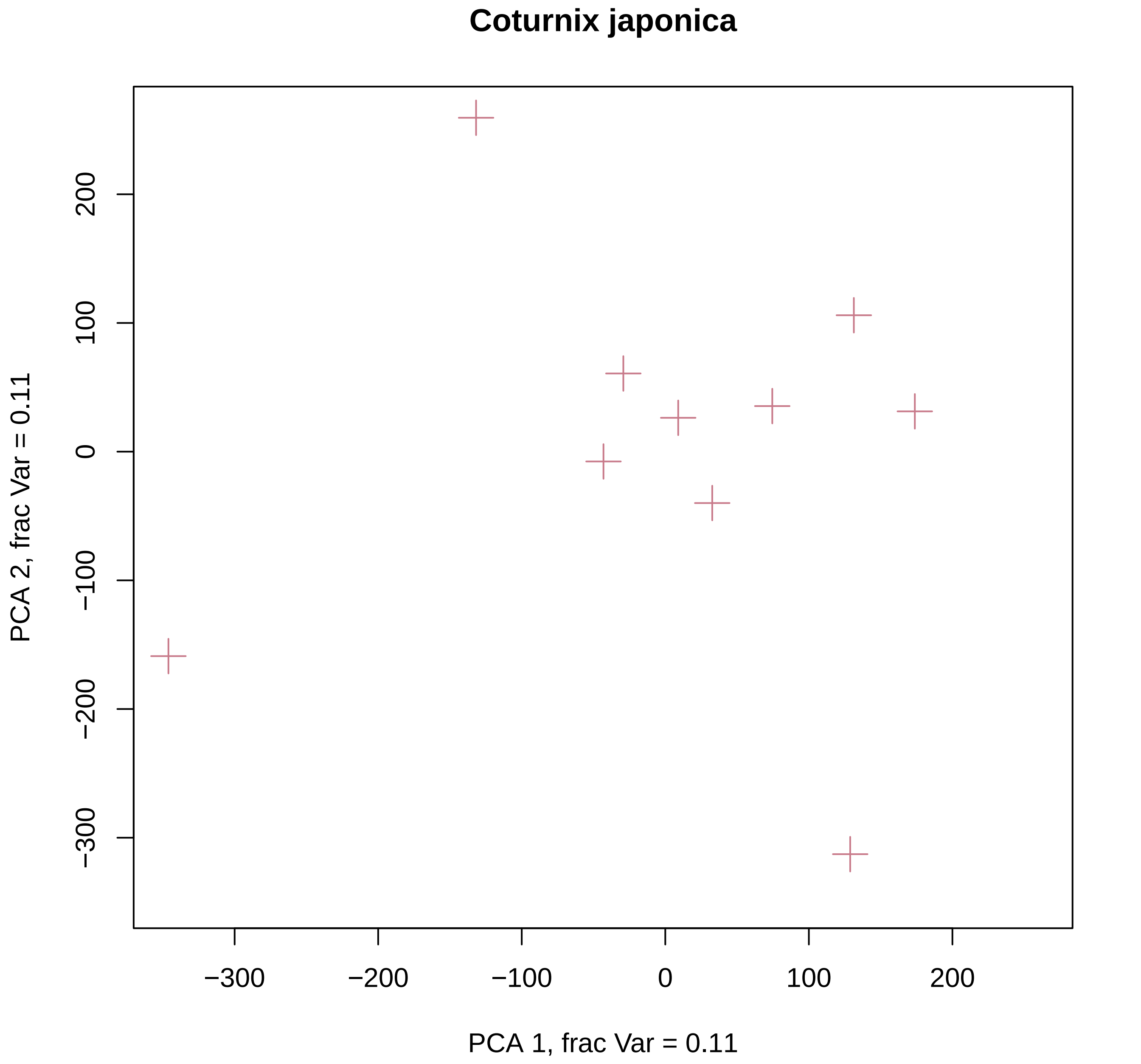
Coturnix japonica
PCA 1, frac Var = 0.11
PCA 2, frac Var = 0.11
−300
−200
−100
0
100
200

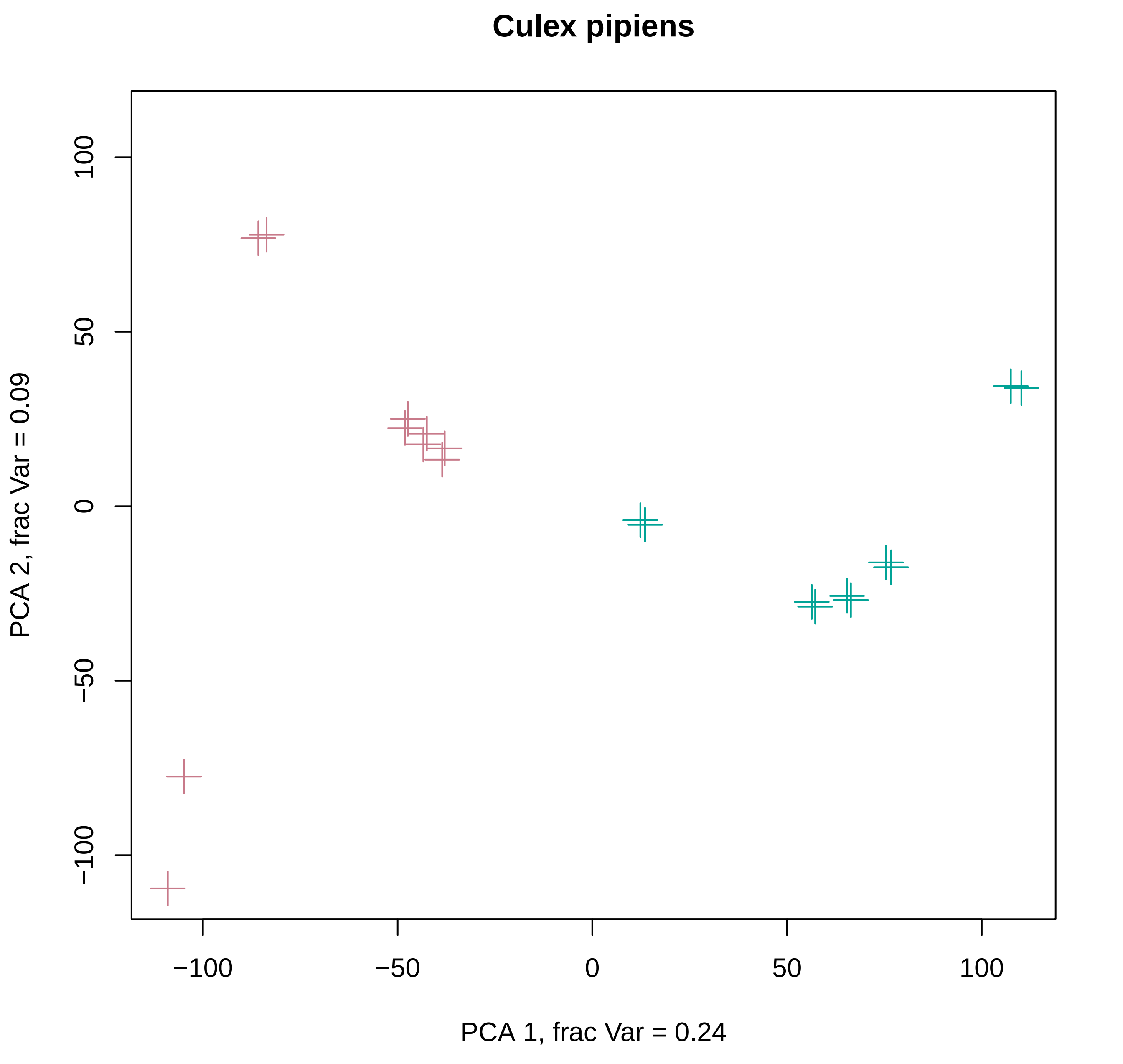
Culex pipiens
PCA 1, frac Var = 0.24
PCA 2, frac Var = 0.09
−100
−50
0
50
100
−100
−50
0
50
100

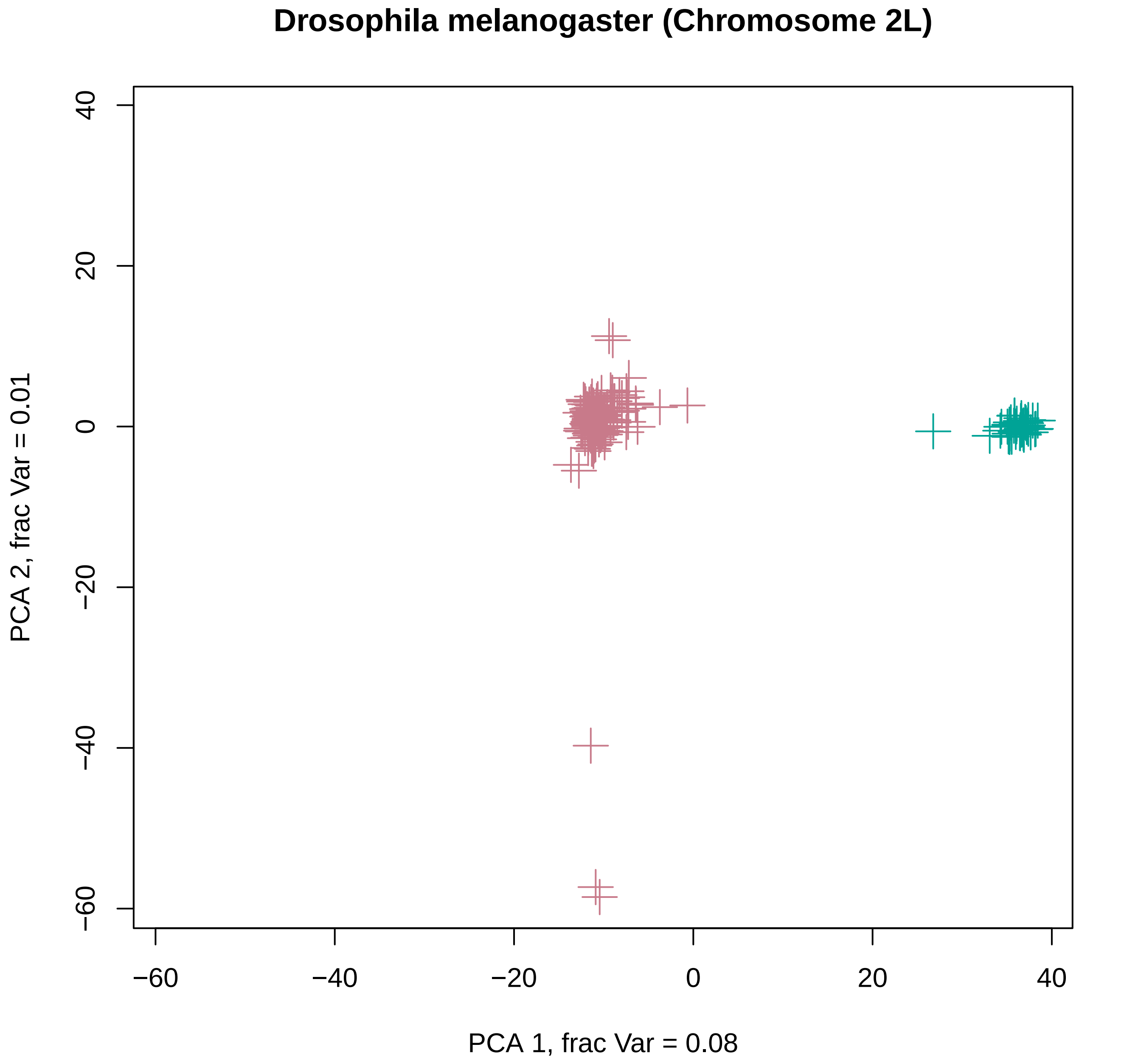
Drosophila melanogaster (Chromosome 2L)
PCA 1, frac Var = 0.08
PCA 2, frac Var = 0.01
−60
−40
−20
0
20
40

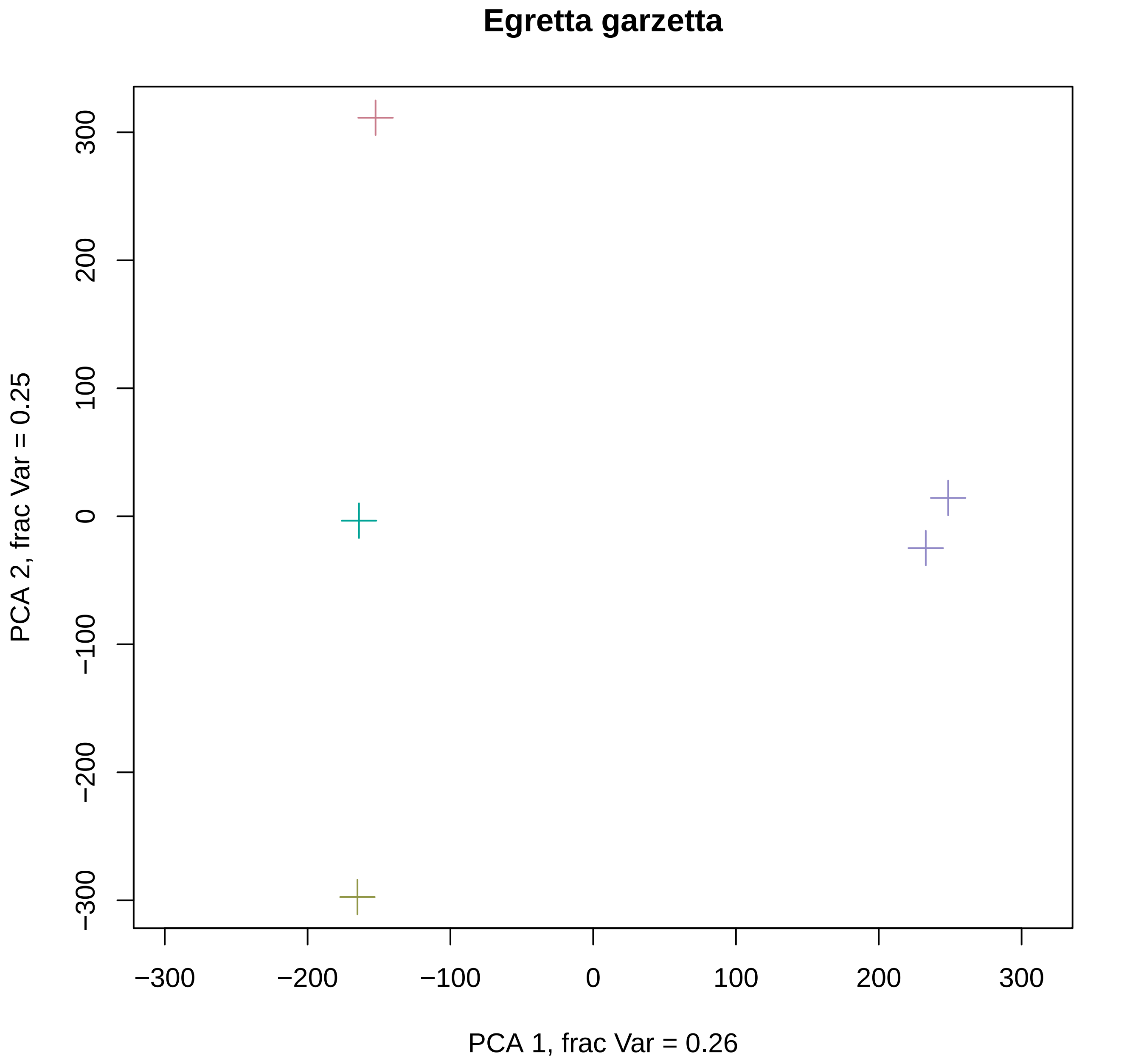

Egretta garzetta
PCA 1, frac Var = 0.26
PCA 2, frac Var = 0.25
−300
−200
−100
0
100
200
300
−300
−200
−100
0
100
200
300

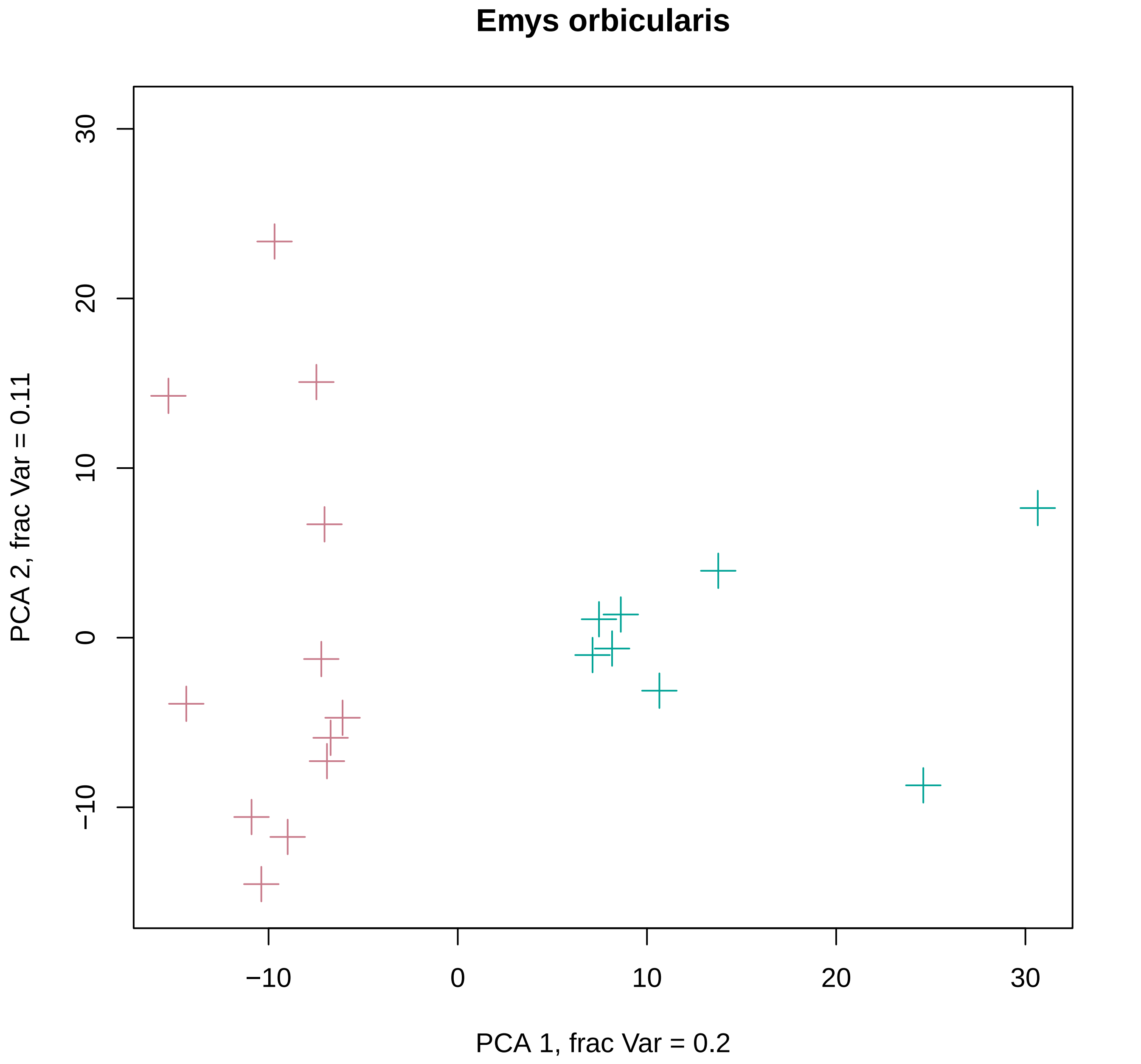
Emys orbicularis
30
20
10
0
−10
PCA 2, frac Var = 0.11
−10
0
10
20
30
PCA 1, frac Var = 0.2

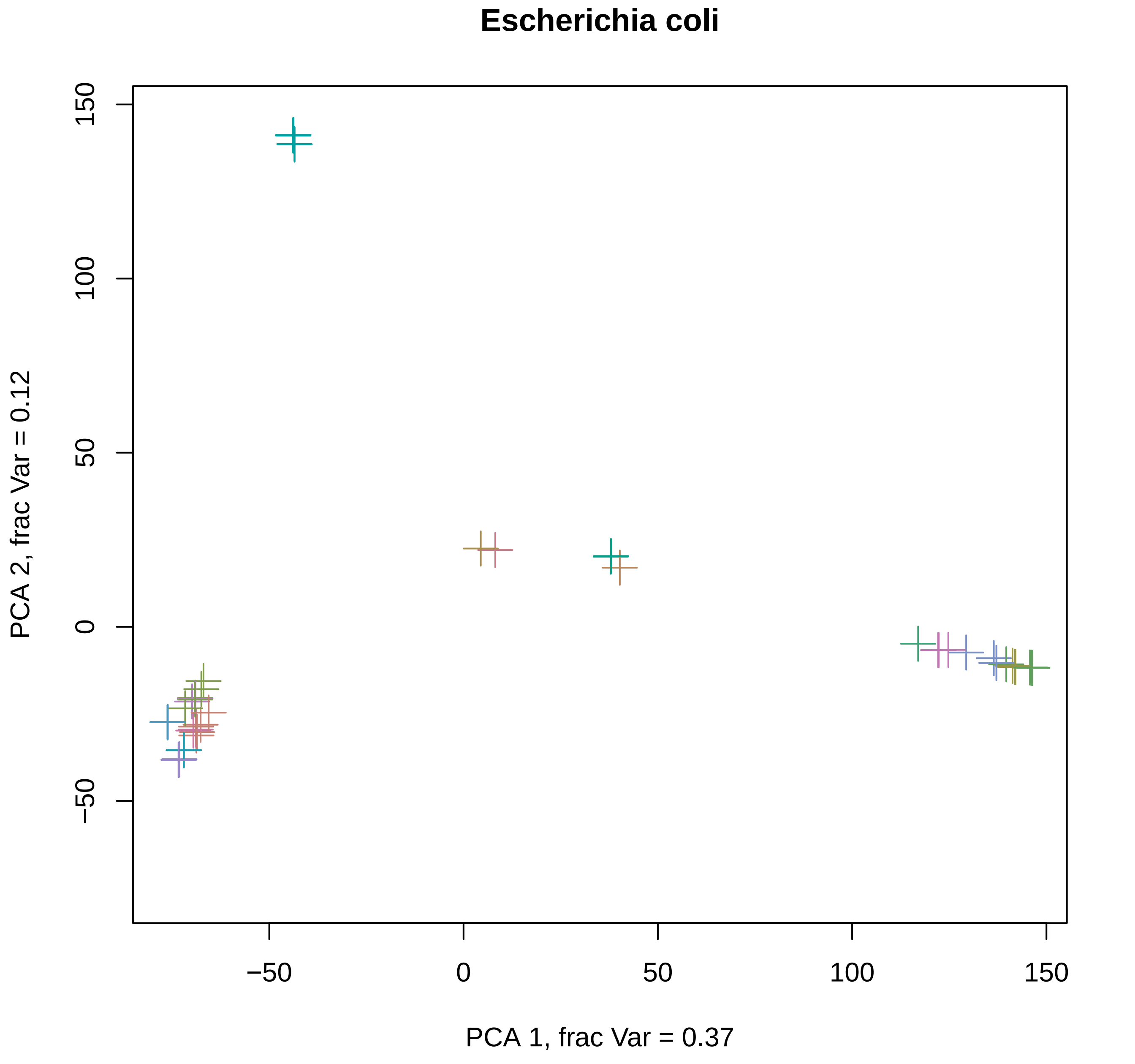

Escherichia coli
PCA 2, frac Var = 0.12
150
100
50
0
−50
−50
0
50
100
150
PCA 1, frac Var = 0.37

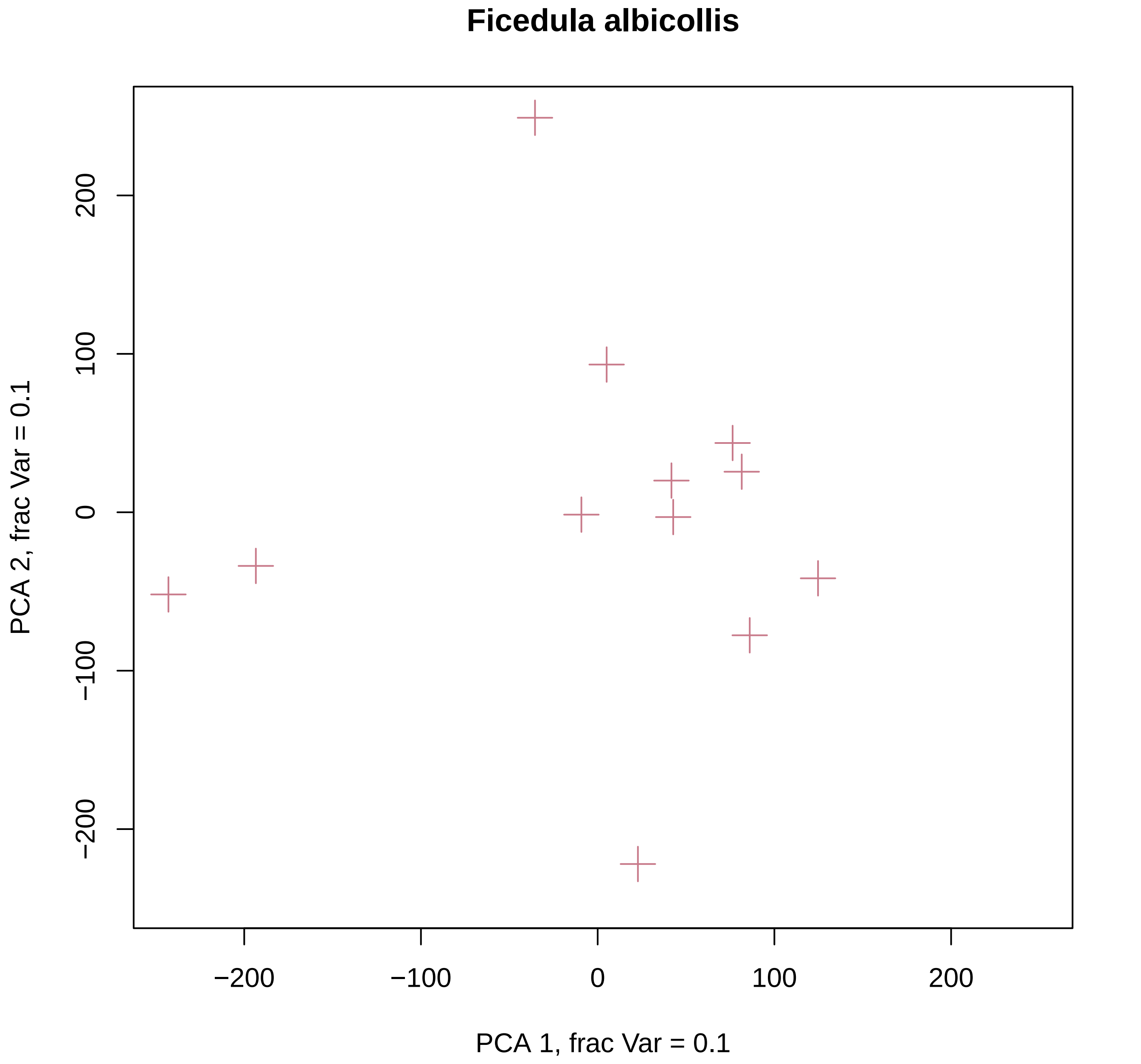

Ficedula albicollis
200
100
0
−100
−200
PCA 2, frac Var = 0.1
−200
−100
0
100
200
PCA 1, frac Var = 0.1

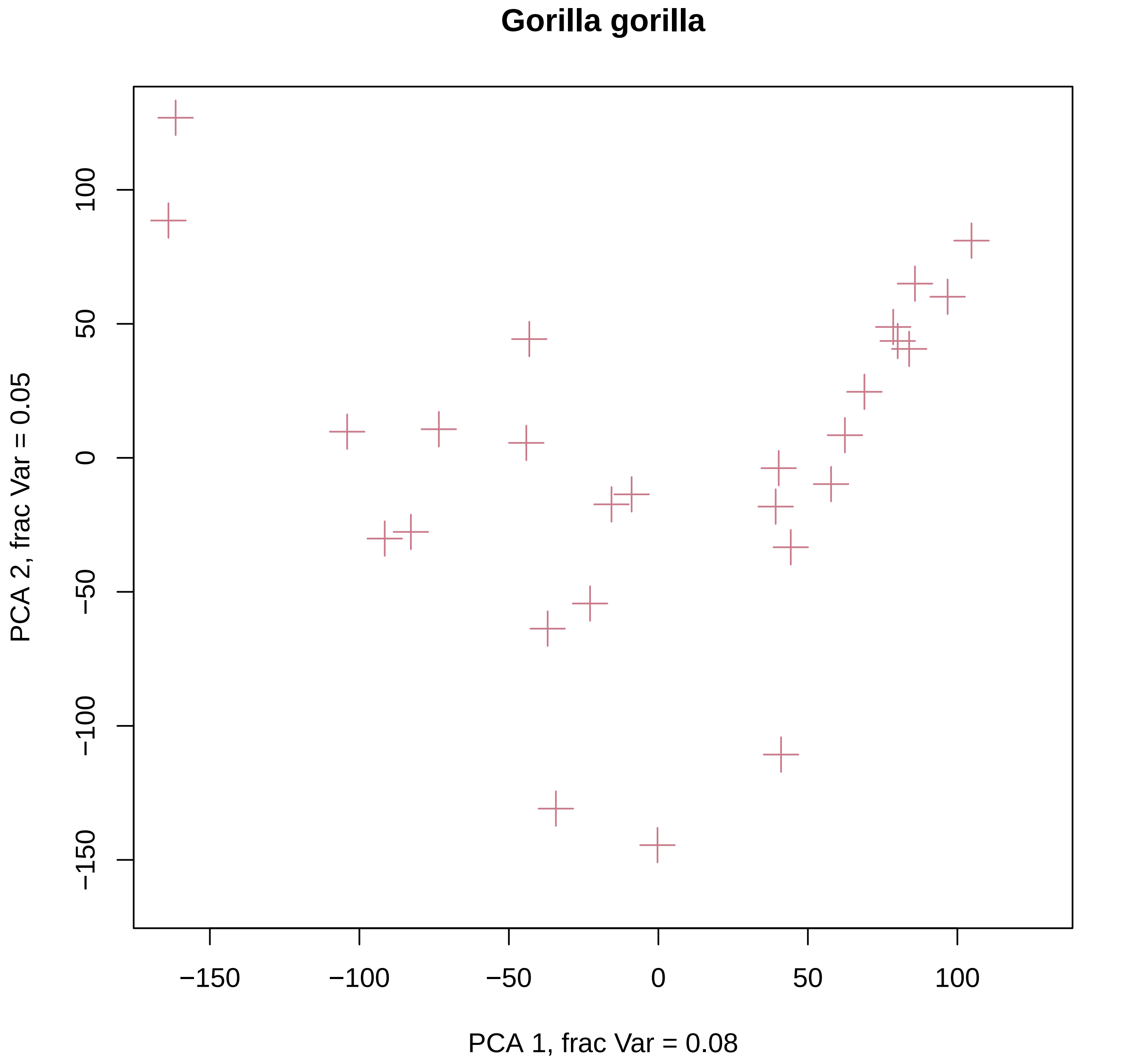
Gorilla gorilla
PCA 1, frac Var = 0.08
PCA 2, frac Var = 0.05
−150
−100
−50
0
50
100

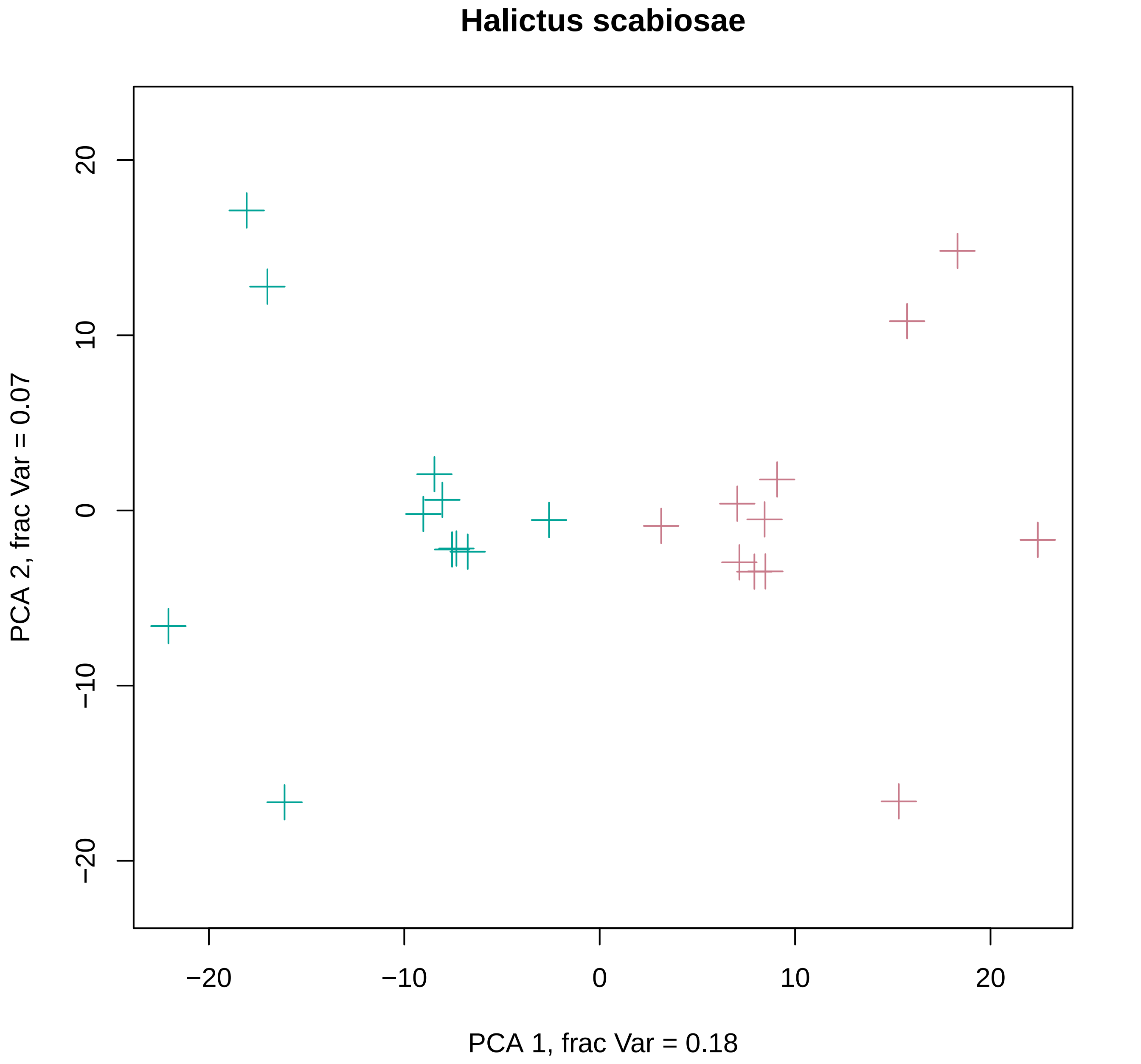
Halictus scabiosae
PCA 1, frac Var = 0.18
PCA 2, frac Var = 0.07
−20
−10
0
10
20
−20
−10
0
10
20

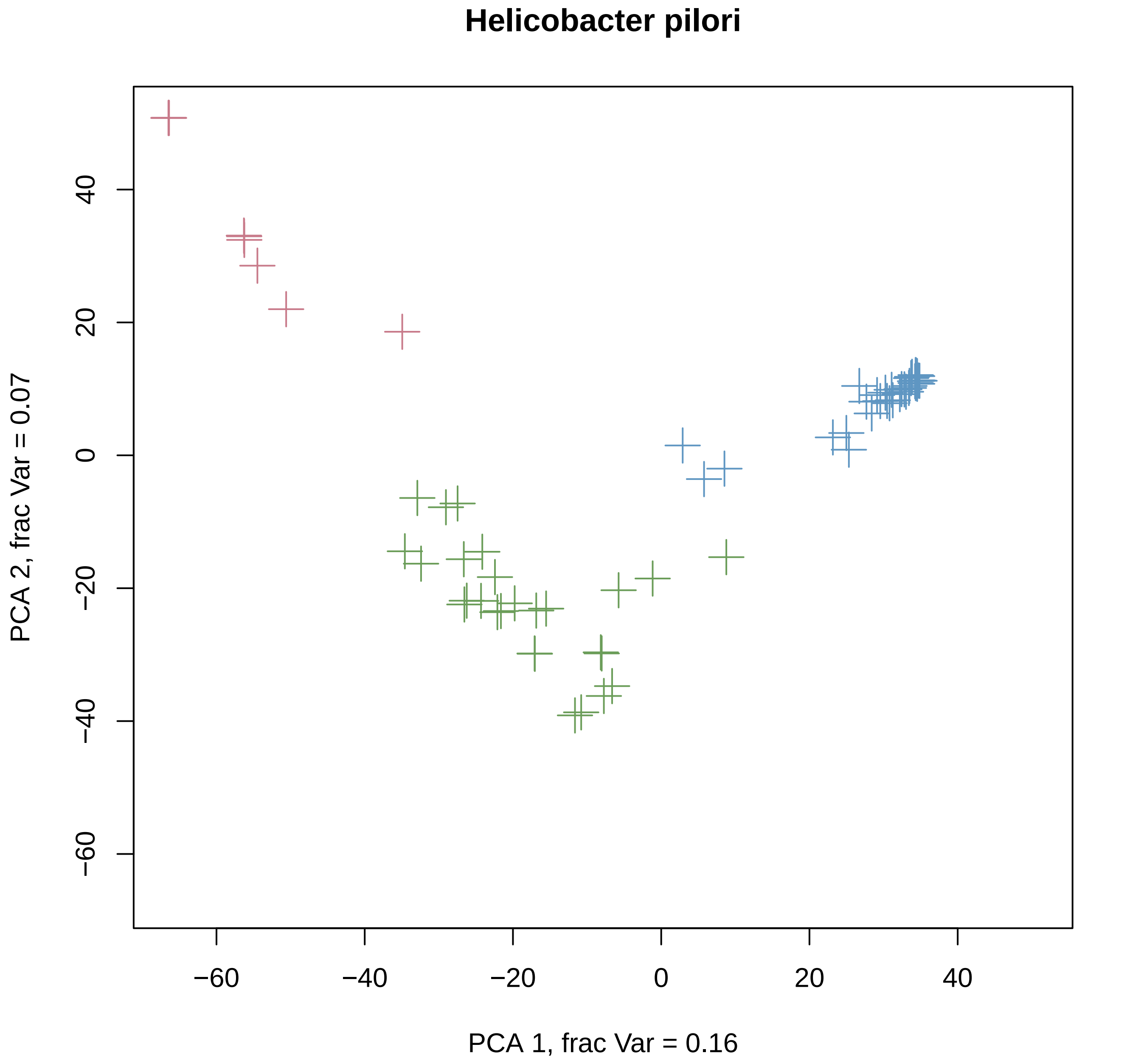
Helicobacter pilori
PCA 2, frac Var = 0.07
PCA 1, frac Var = 0.16
40
20
0
−20
−40
−60
−60
−40
−20
0
20
40

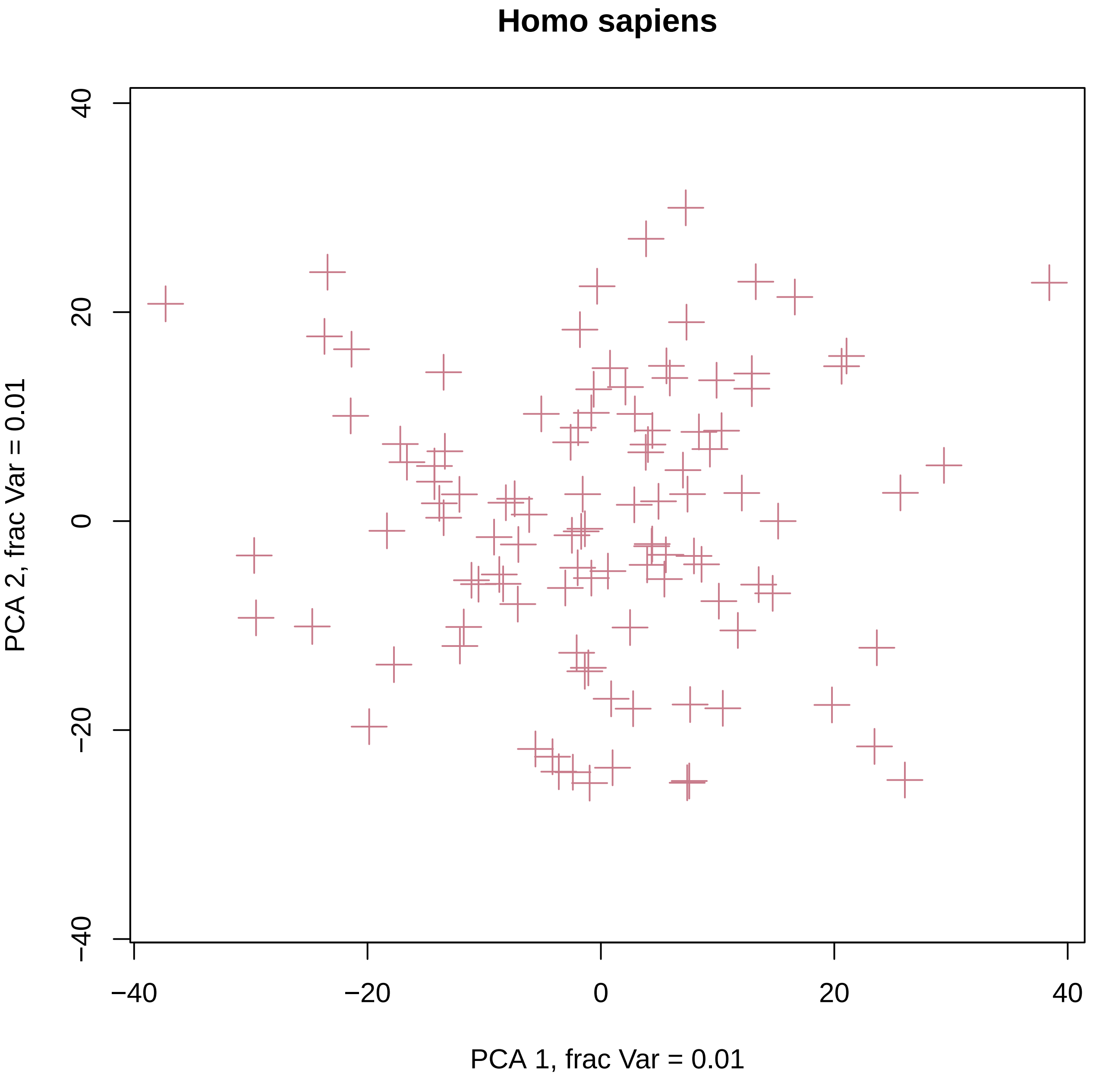

Homo sapiens
PCA 2, frac Var = 0.01
PCA 1, frac Var = 0.01
−40
−20
0
20
40
−40
−20
0
20
40

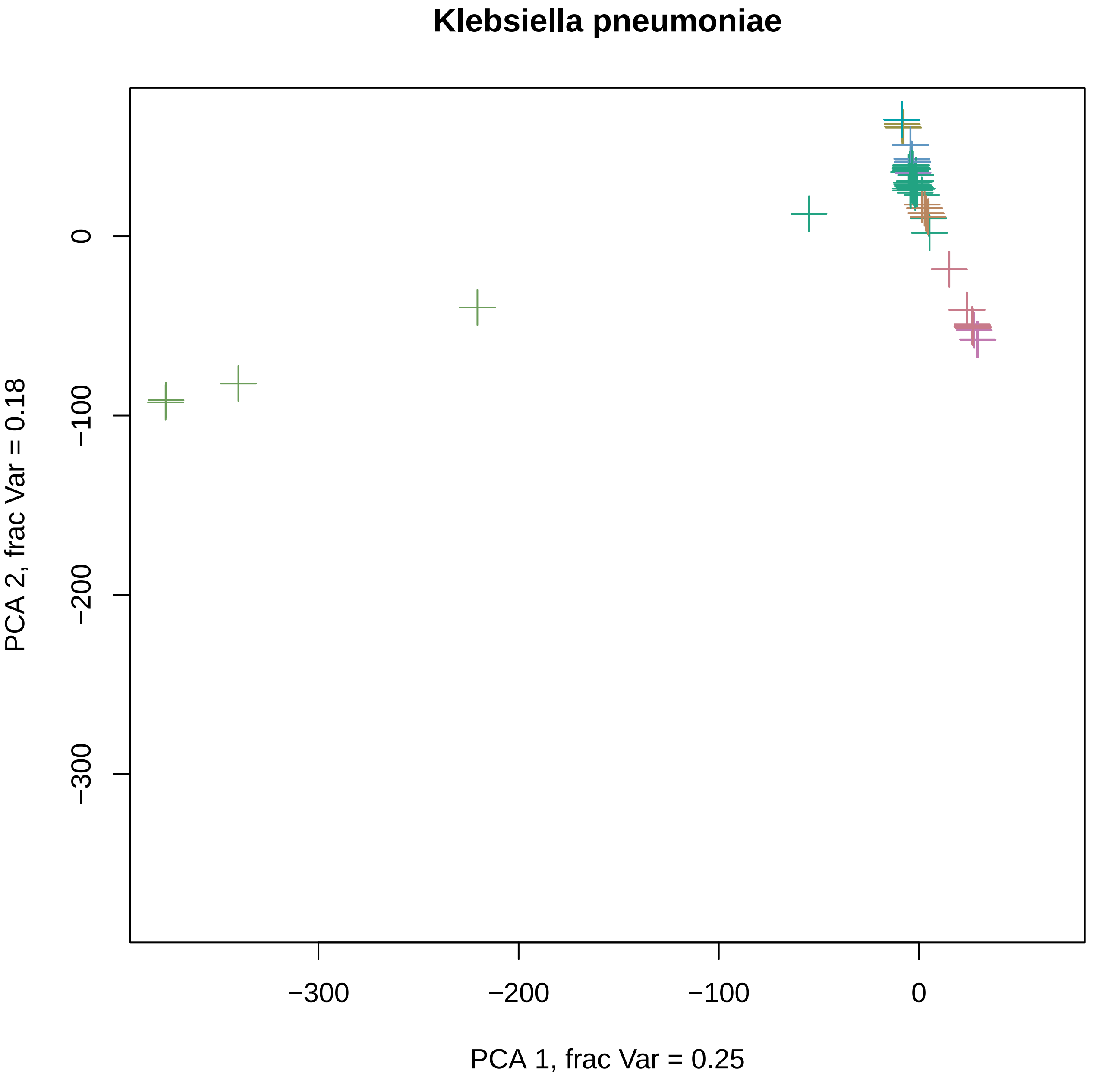
Klebsiella pneumoniae
PCA 1, frac Var = 0.25
PCA 2, frac Var = 0.18
−300
−200
−100
0
0
−100
−200
−300

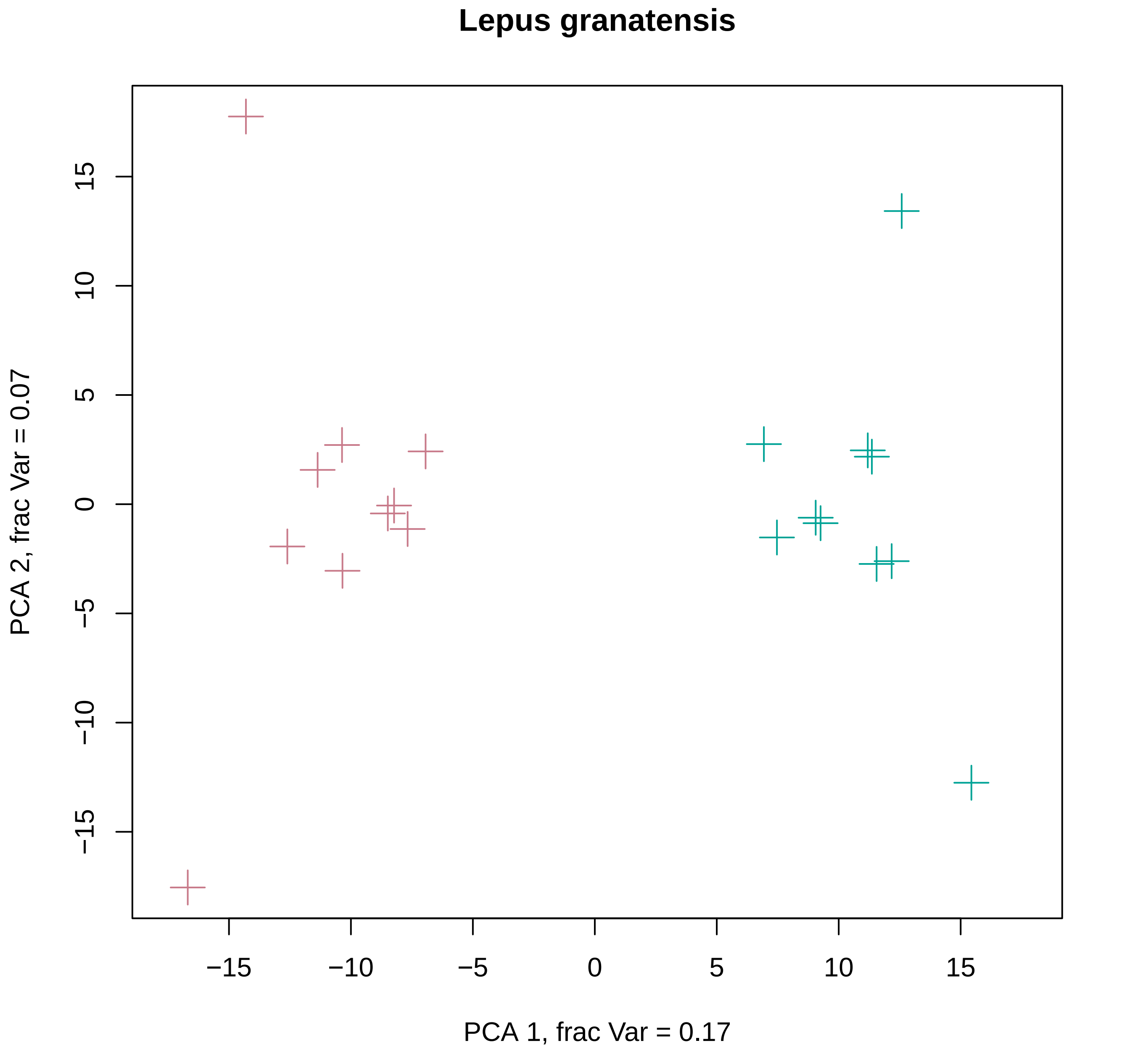
Lepus granatensis
PCA 1, frac Var = 0.17
PCA 2, frac Var = 0.07
−15
−10
−5
0
5
10
15

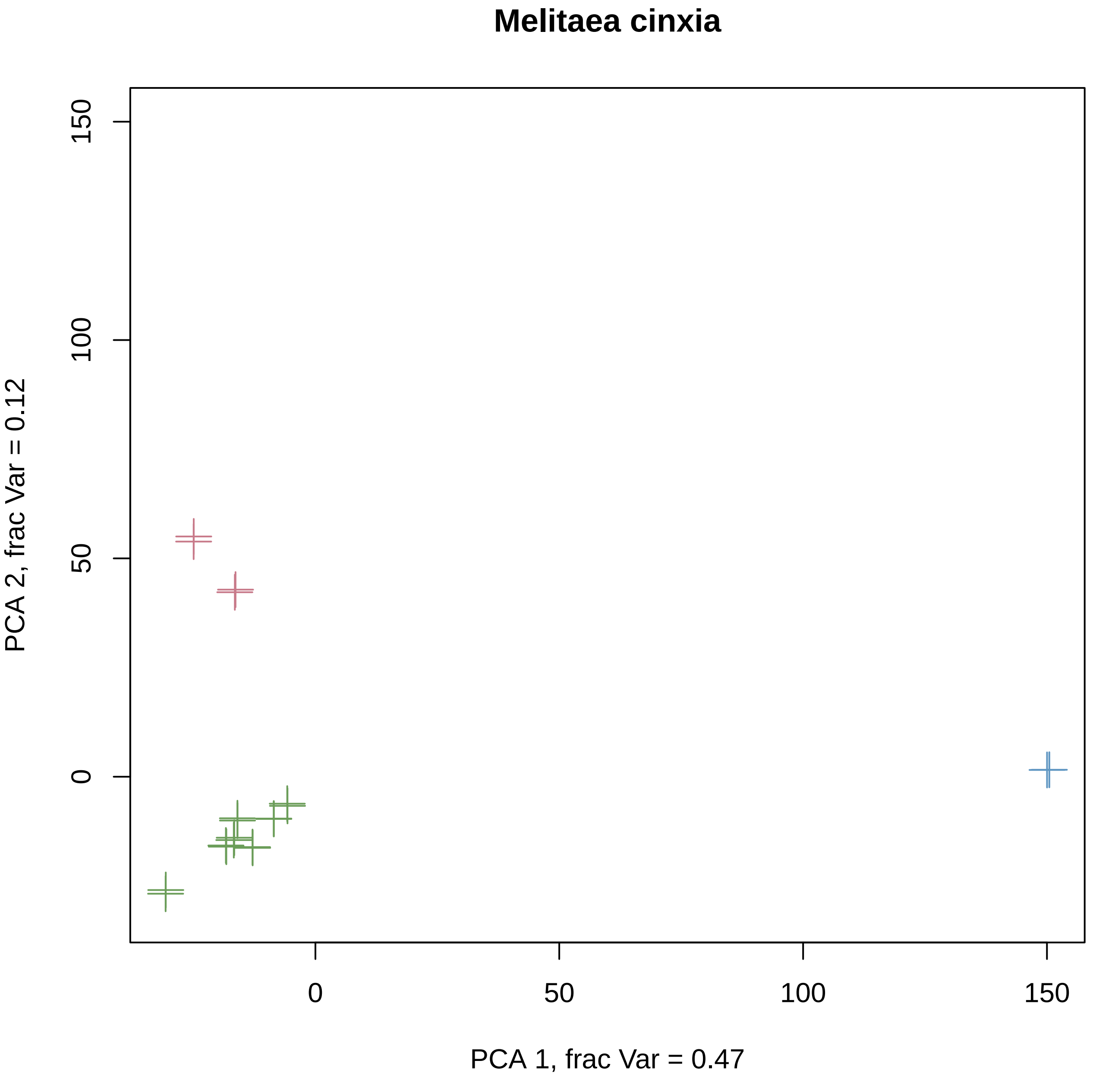
Melitaea cinxia
PCA 1, frac Var = 0.47
PCA 2, frac Var = 0.12
0
50
100
150
0
50
100
150

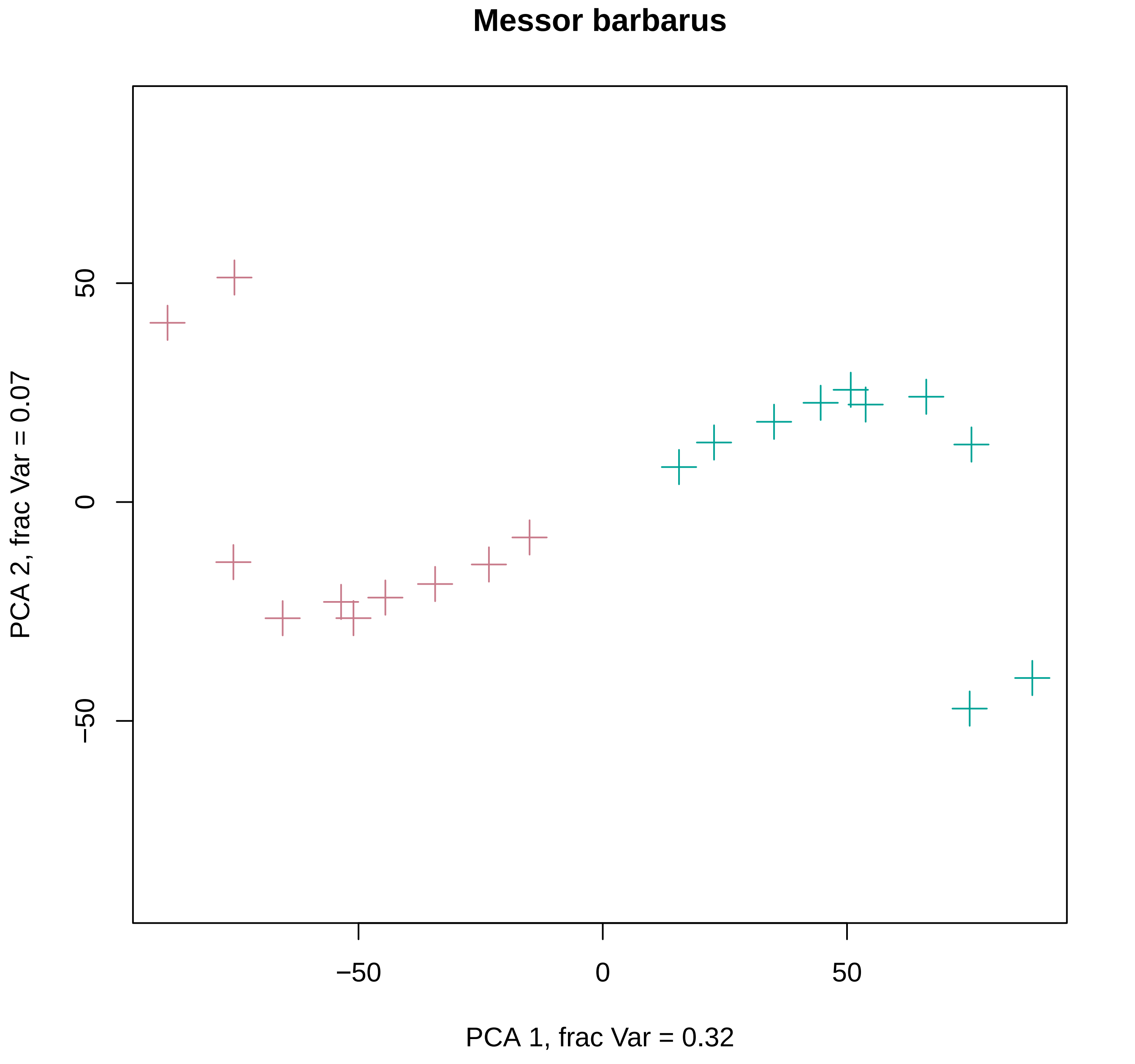

Messor barbarus
50
0
−50
−50
0
50
PCA 1, frac Var = 0.32
PCA 2, frac Var = 0.07

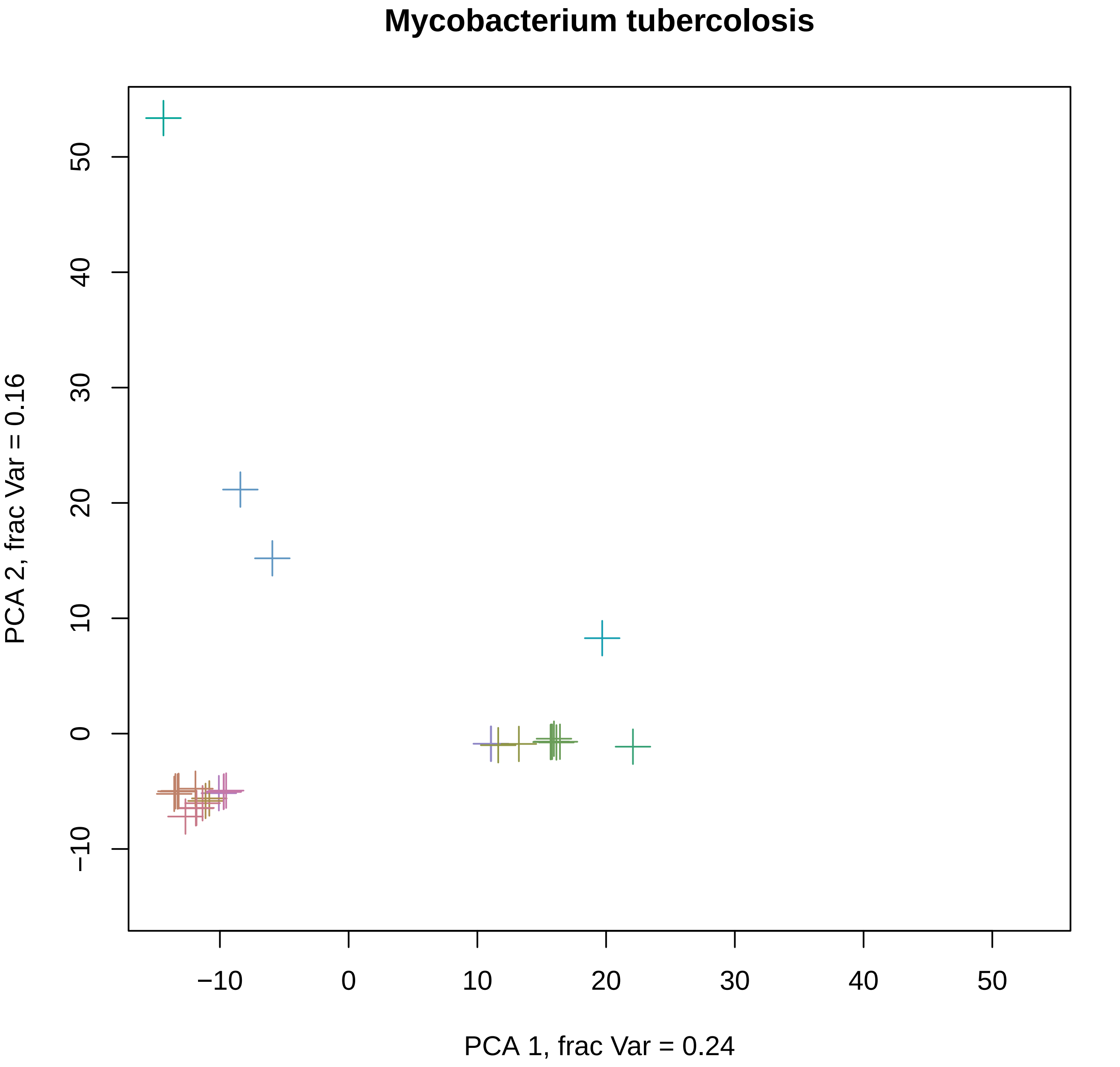

Mycobacterium tubercolosis
PCA 1, frac Var = 0.24
PCA 2, frac Var = 0.16
−10
0
10
20
30
40
50

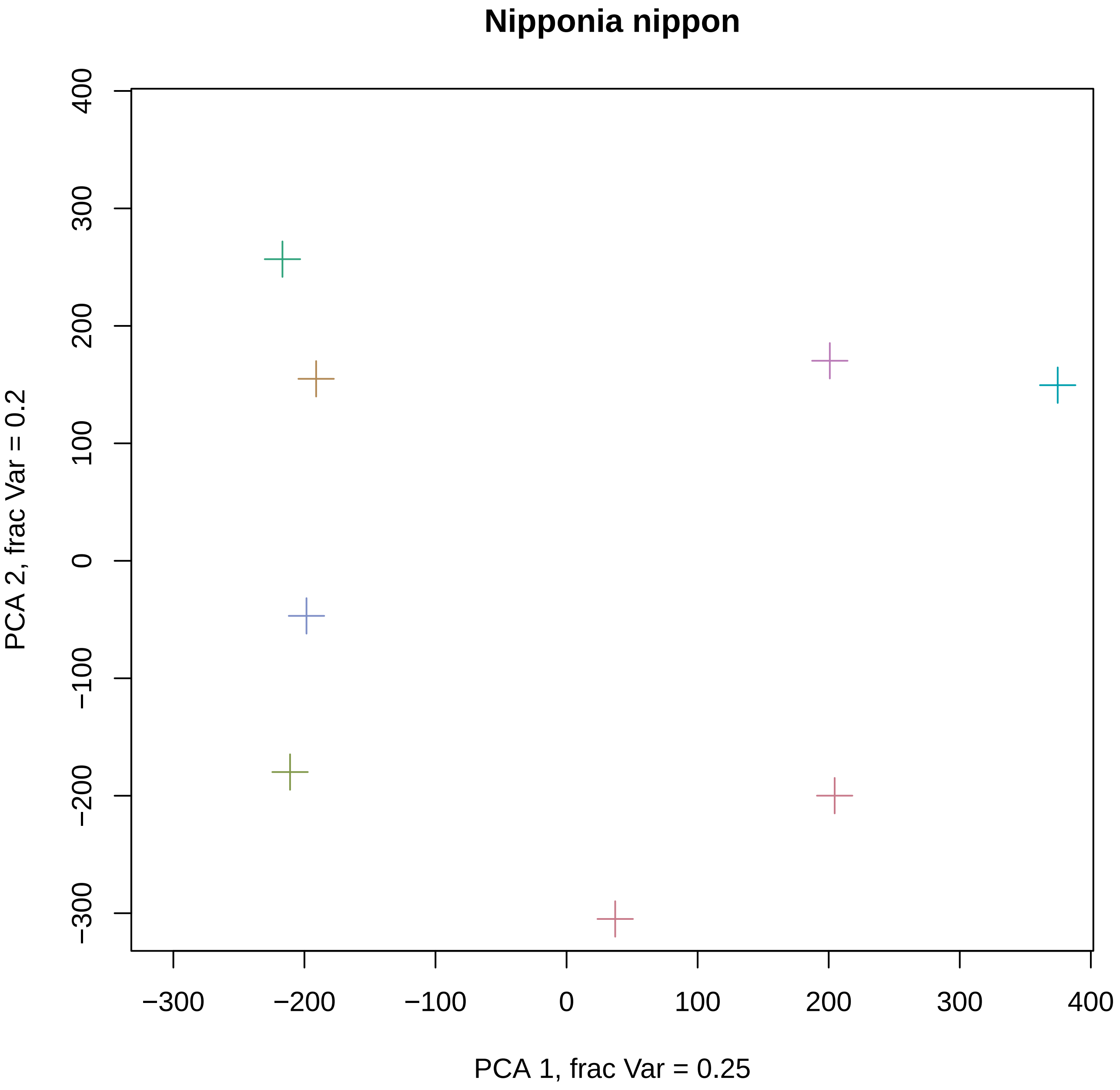
Nipponia nippon
PCA 1, frac Var = 0.25
PCA 2, frac Var = 0.2
−300
−200
−100
0
100
200
300
400

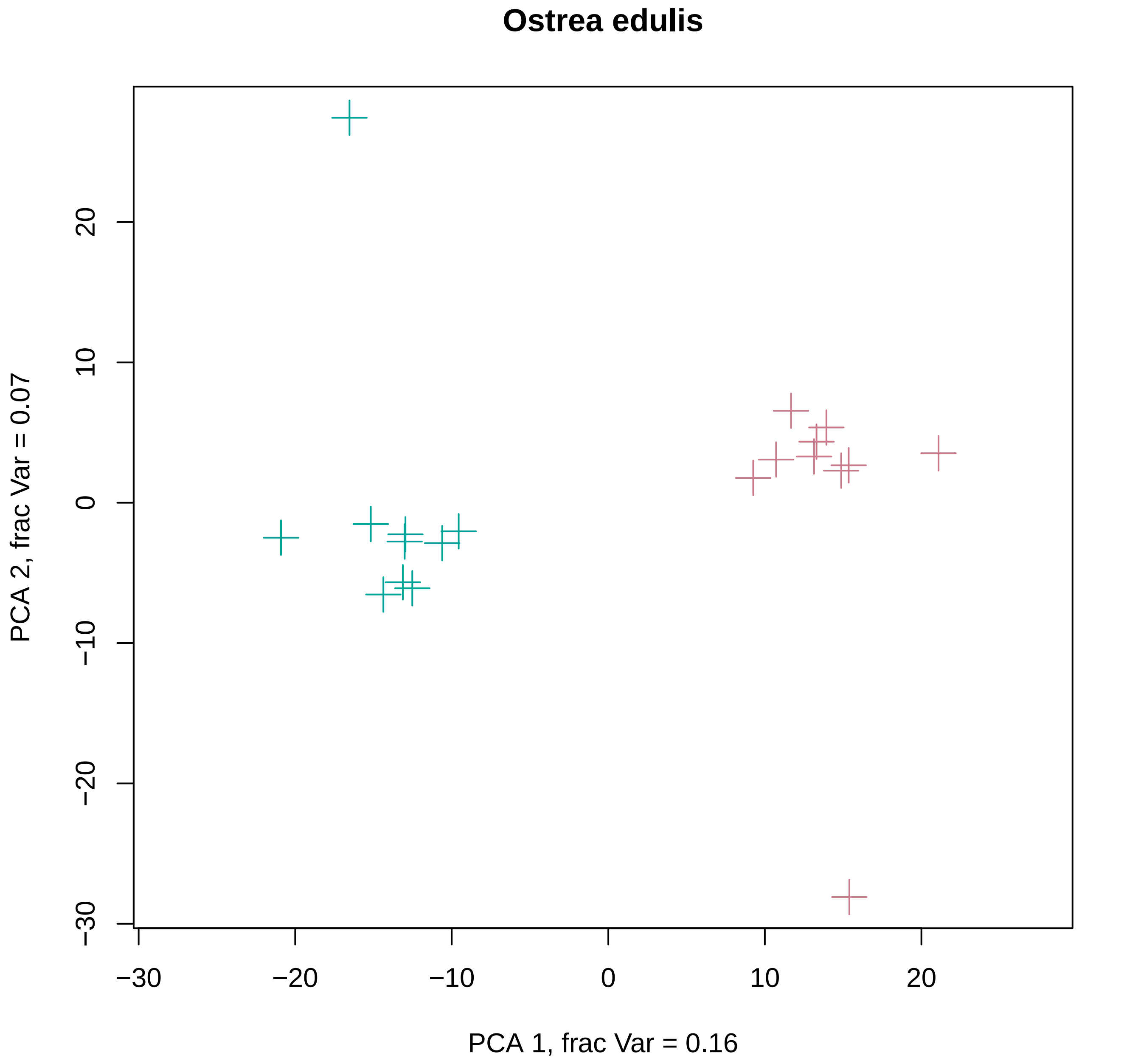

Ostrea edulis
PCA 1, frac Var = 0.16
PCA 2, frac Var = 0.07
−30
−20
−10
0
10
20

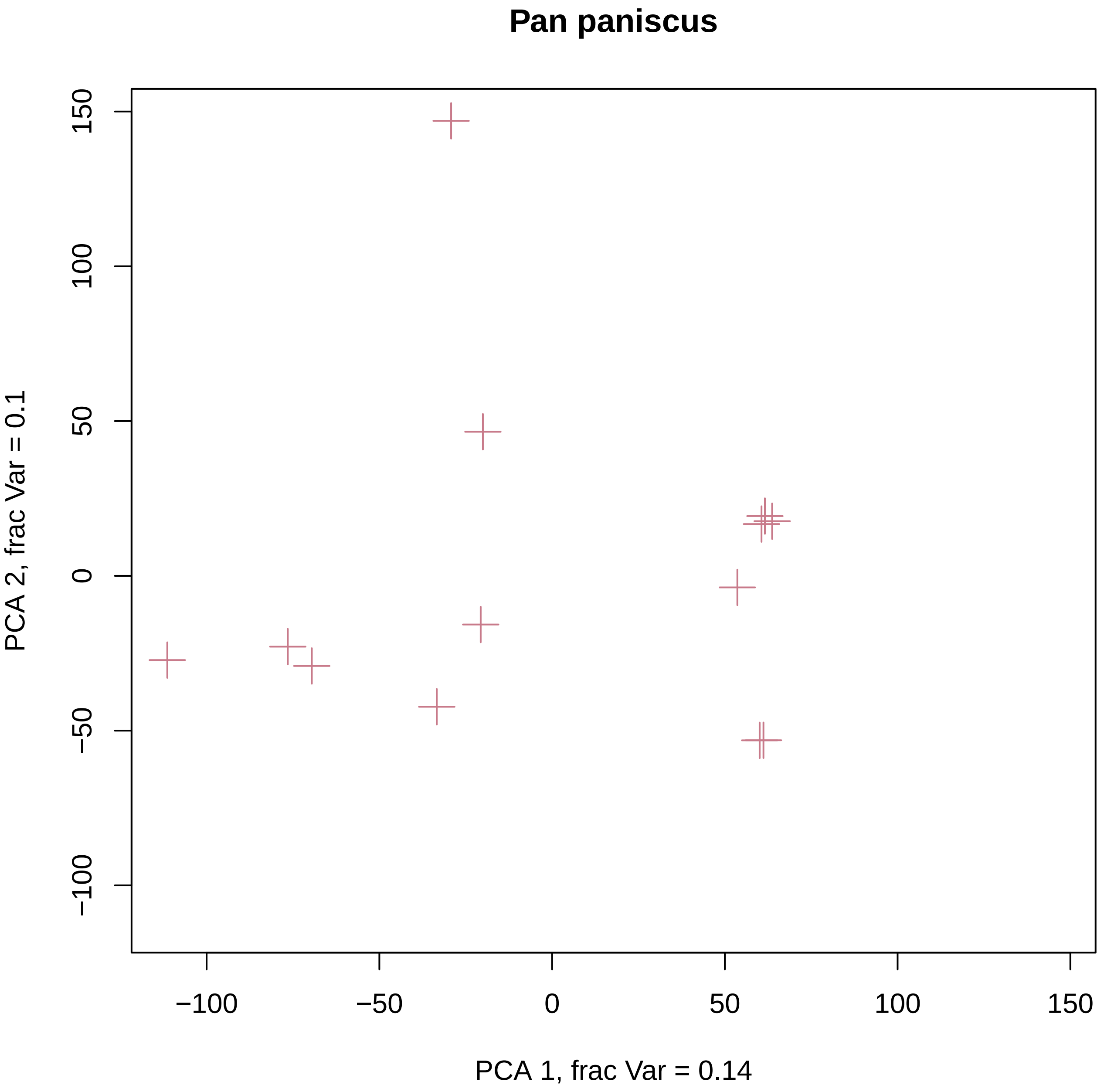
Pan paniscus
150
100
50
0
−50
−100
PCA 2, frac Var = 0.1
−100
−50
0
50
100
150
PCA 1, frac Var = 0.14

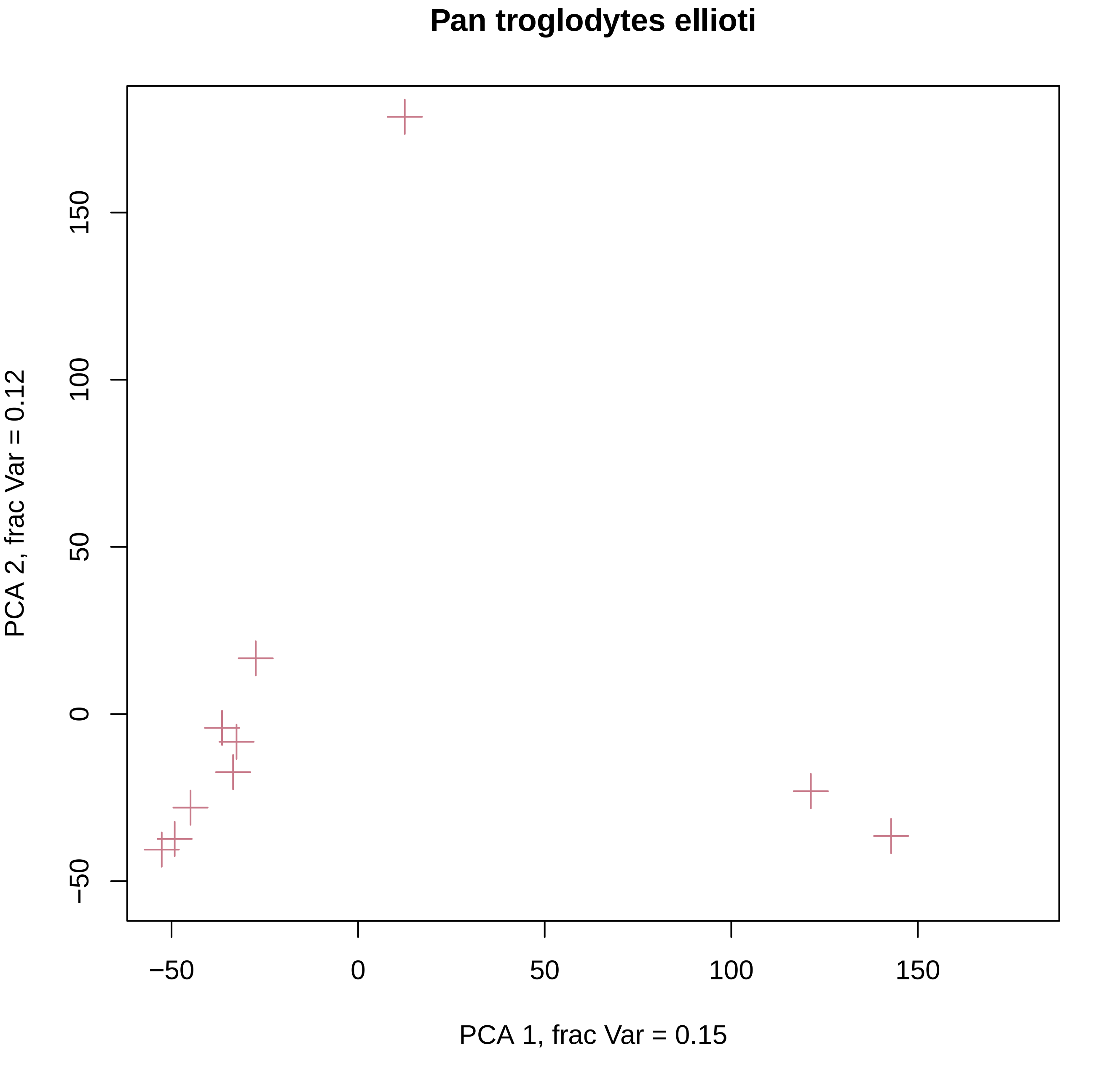

Pan troglodytes ellioti
PCA 1, frac Var = 0.15
PCA 2, frac Var = 0.12
−50
0
50
100
150
−50
0
50
100
150

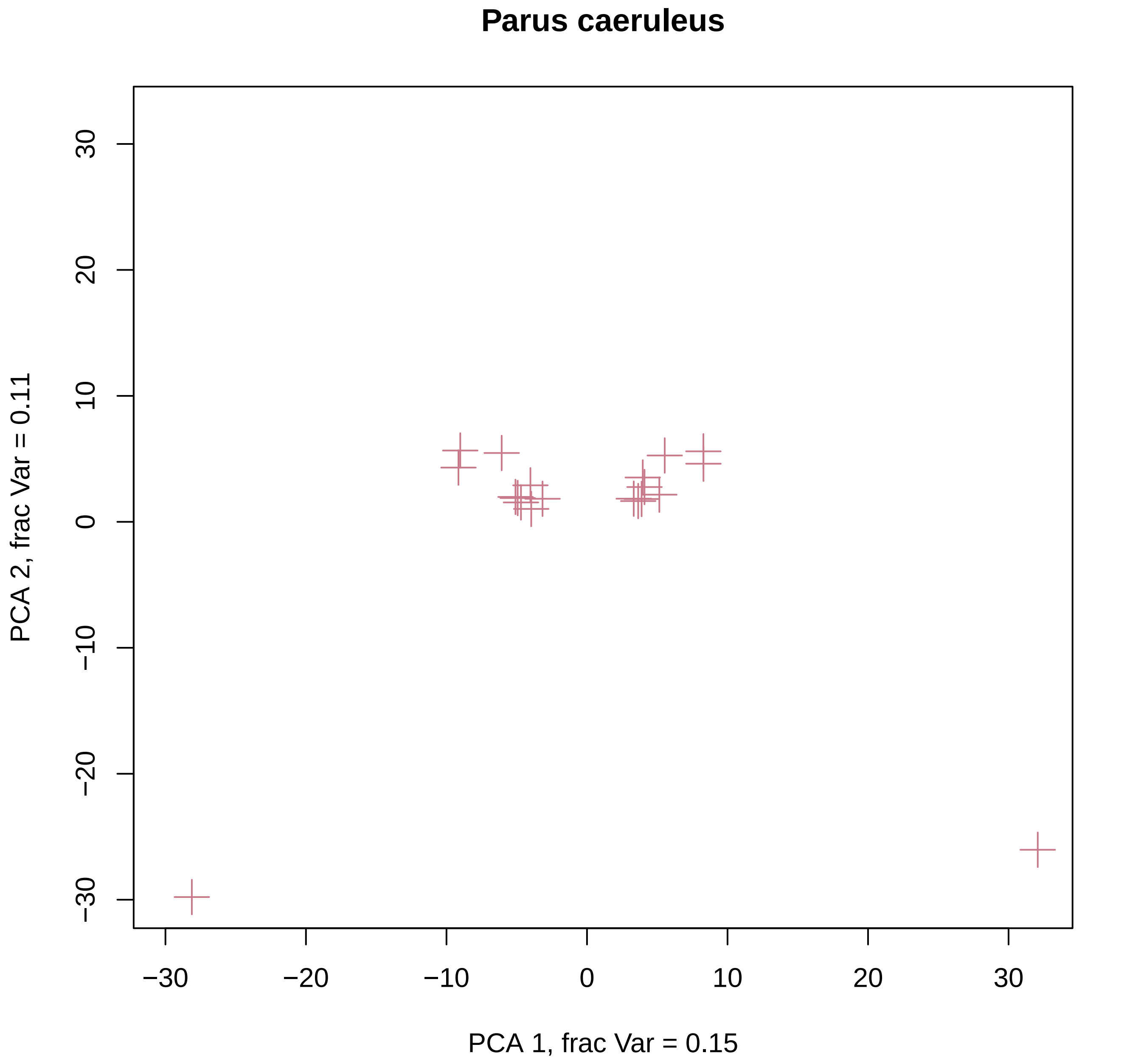
Parus caeruleus
PCA 2, frac Var = 0.11
PCA 1, frac Var = 0.15
30
20
10
0
−10
−20
−30
−30
−20
−10
0
10
20
30

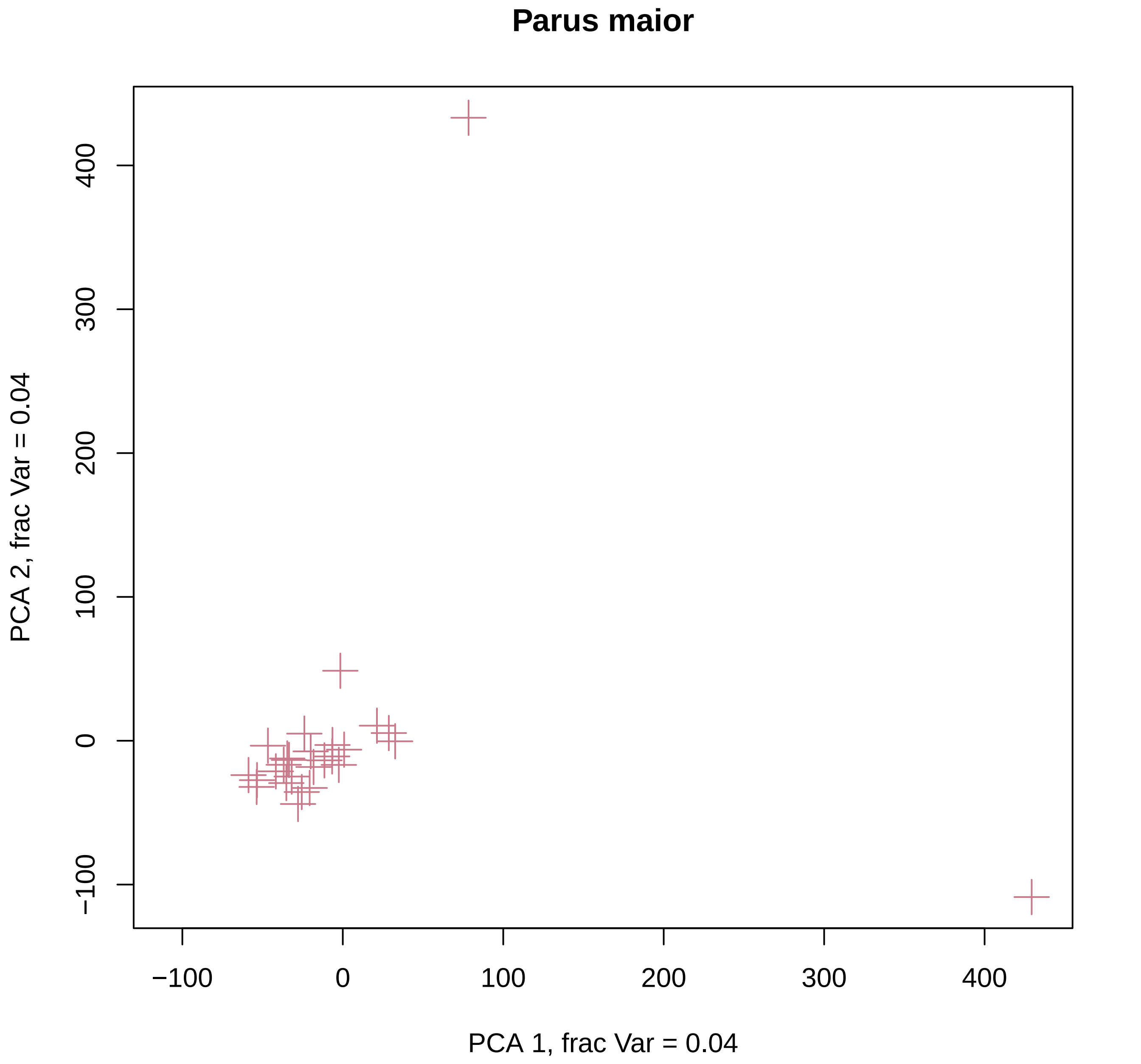
Parus maior
PCA 1, frac Var = 0.04
PCA 2, frac Var = 0.04
−100
0
100
200
300
400
−100
0
100
200
300
400

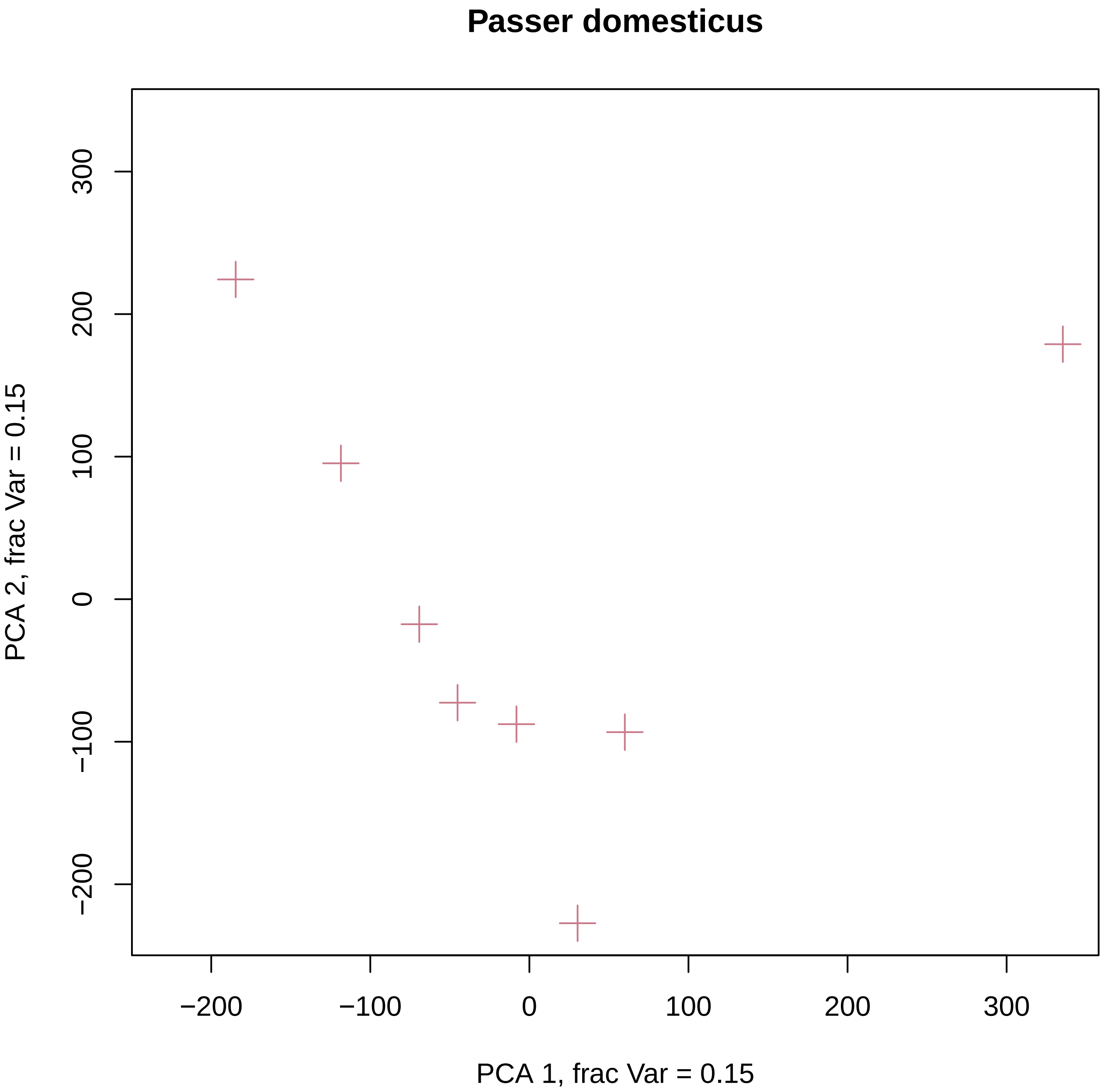
Passer domesticus
PCA 1, frac Var = 0.15
PCA 2, frac Var = 0.15
−200
−100
0
100
200
300
−200
−100
0
100
200
300

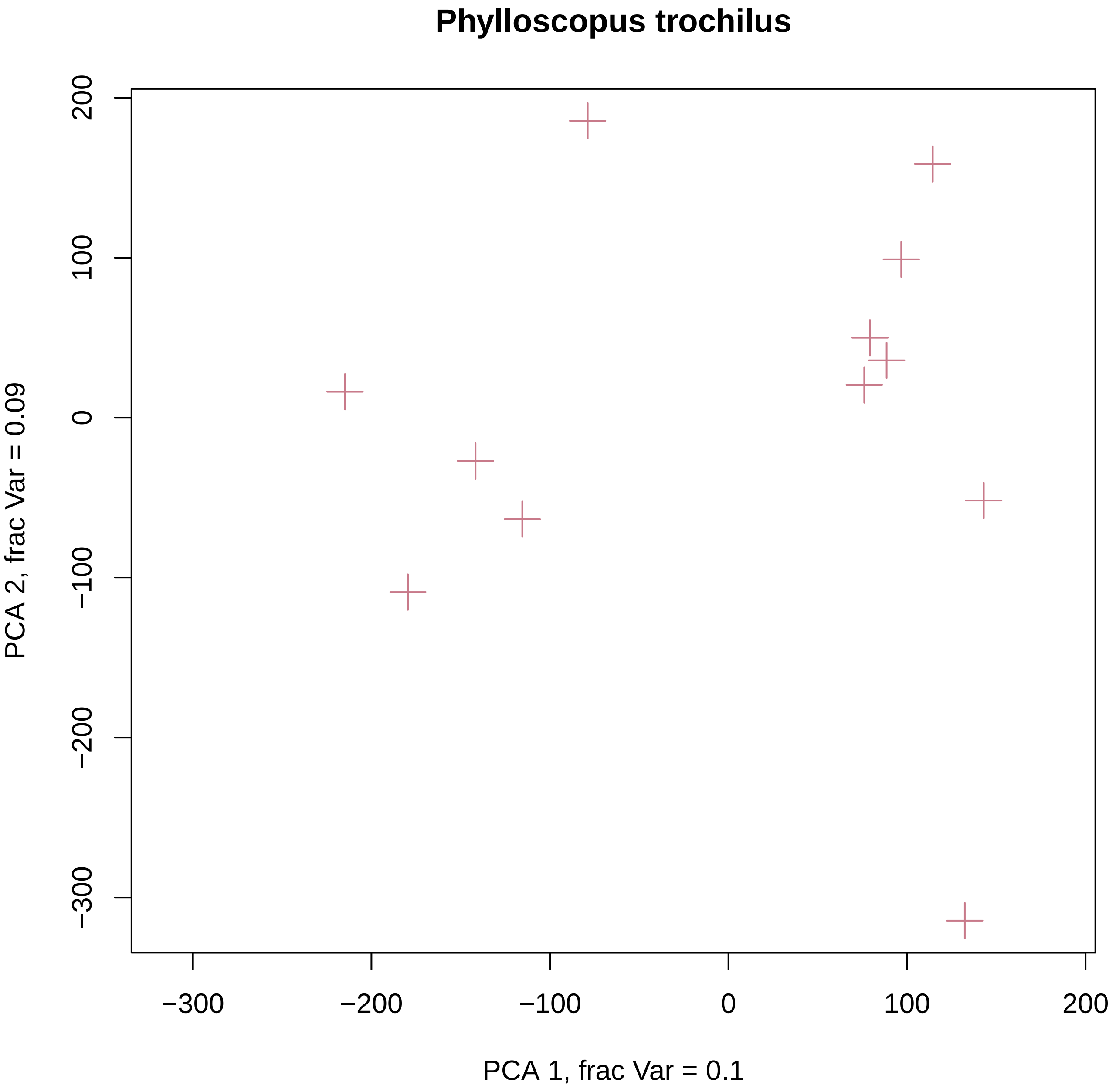
Phylloscopus trochilus
PCA 1, frac Var = 0.1
PCA 2, frac Var = 0.09
−300
−200
−100
0
100
200

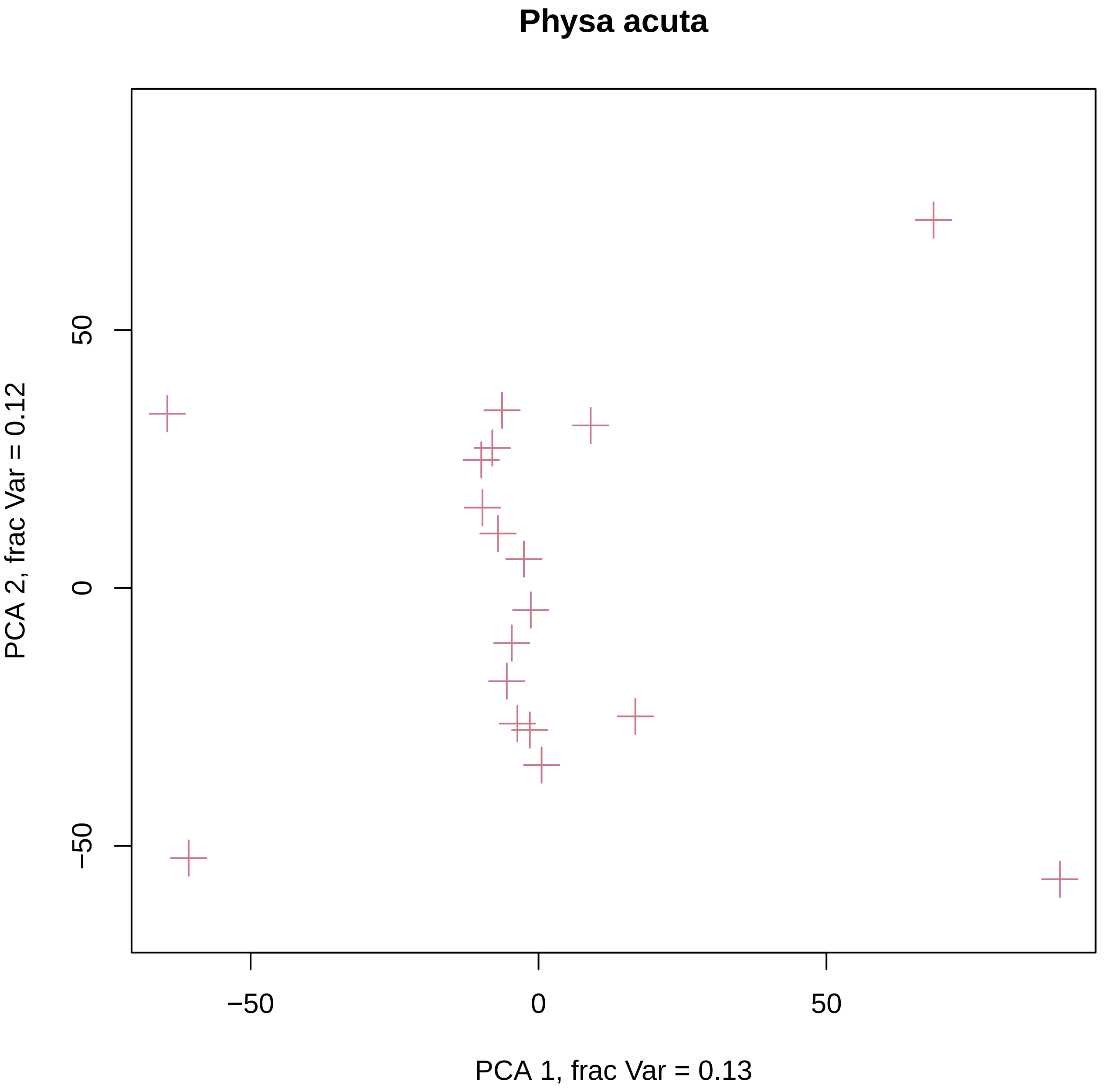
Physa acuta
PCA 1, frac Var = 0.13
PCA 2, frac Var = 0.12
−50
0
50
−50
0
50

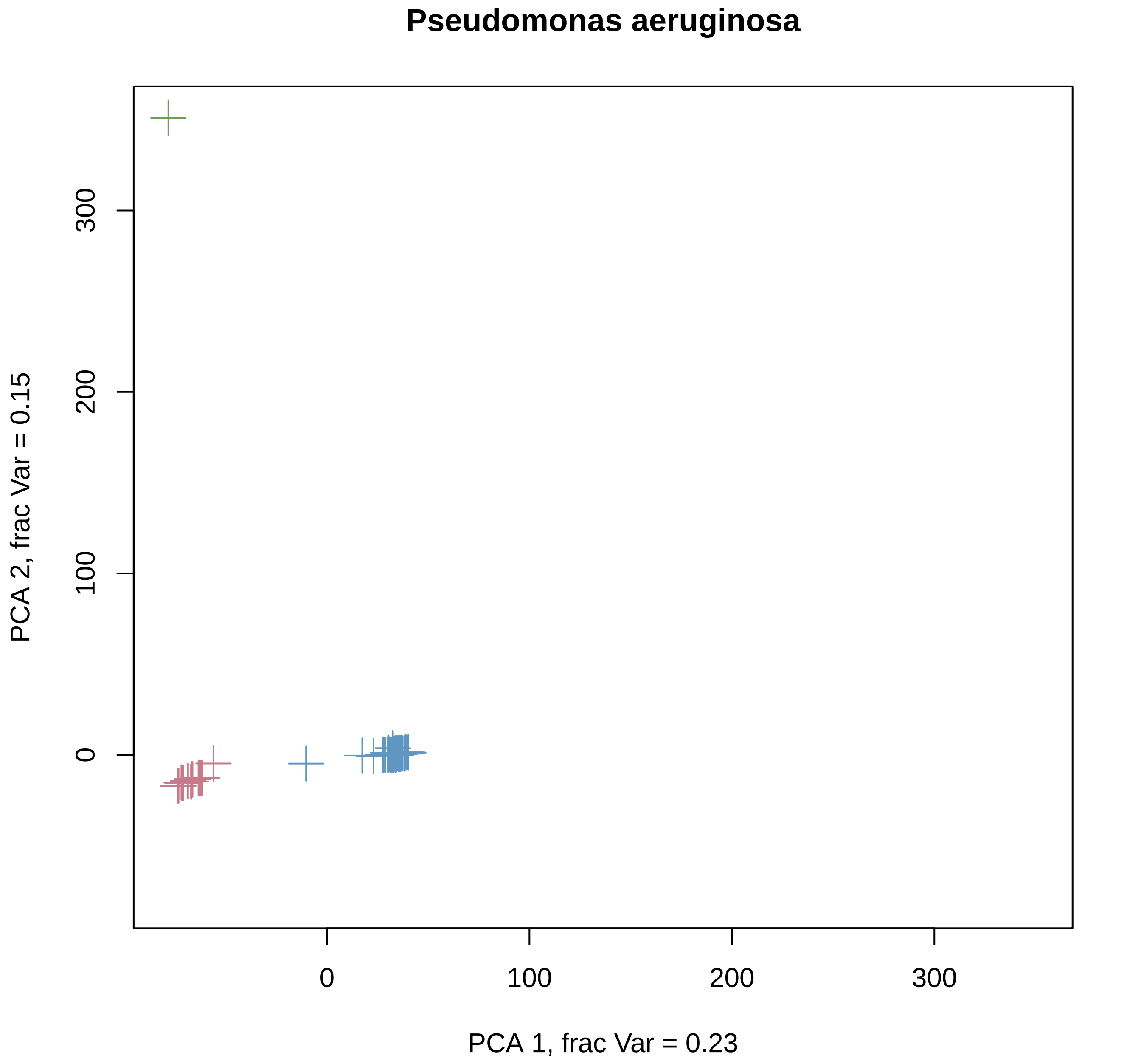
Pseudomonas aeruginosa
PCA 2, frac Var = 0.15
300
200
100
0
0
100
200
300
PCA 1, frac Var = 0.23

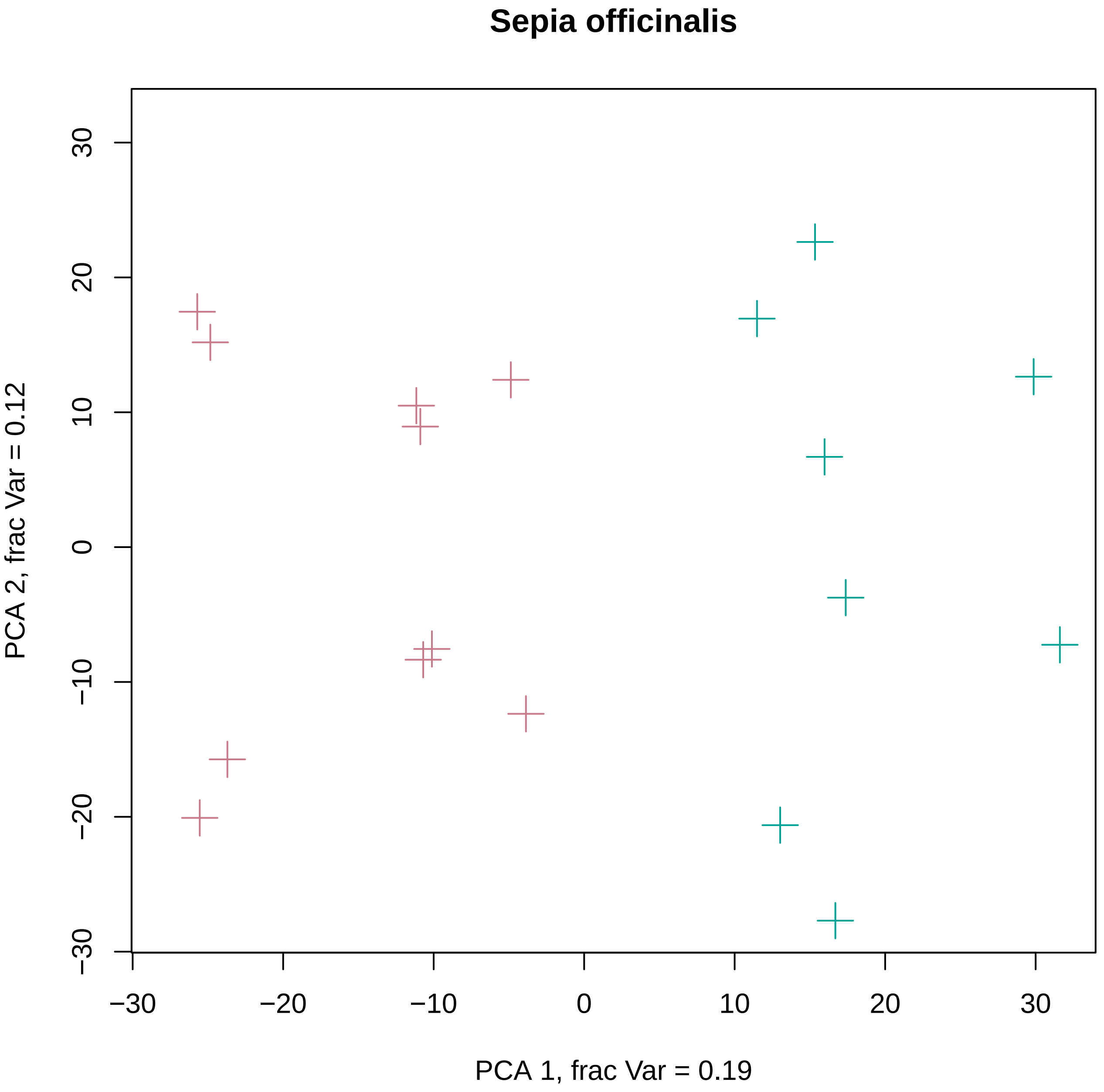

Sepia officinalis
PCA 1, frac Var = 0.19
PCA 2, frac Var = 0.12
−30
−20
−10
0
10
20
30

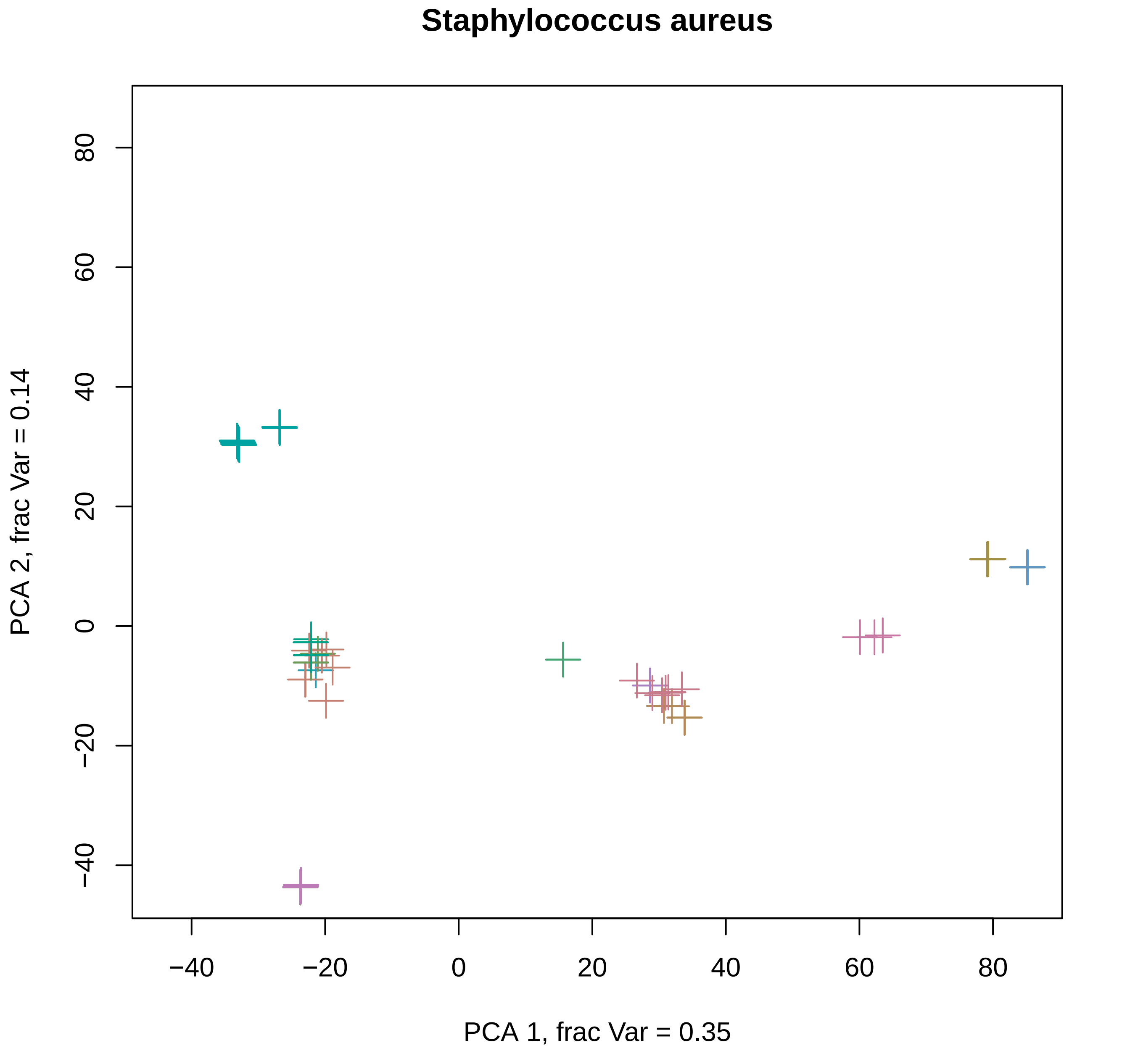

Staphylococcus aureus
PCA 2, frac Var = 0.14
PCA 1, frac Var = 0.35
−40
−20
0
20
40
60
80

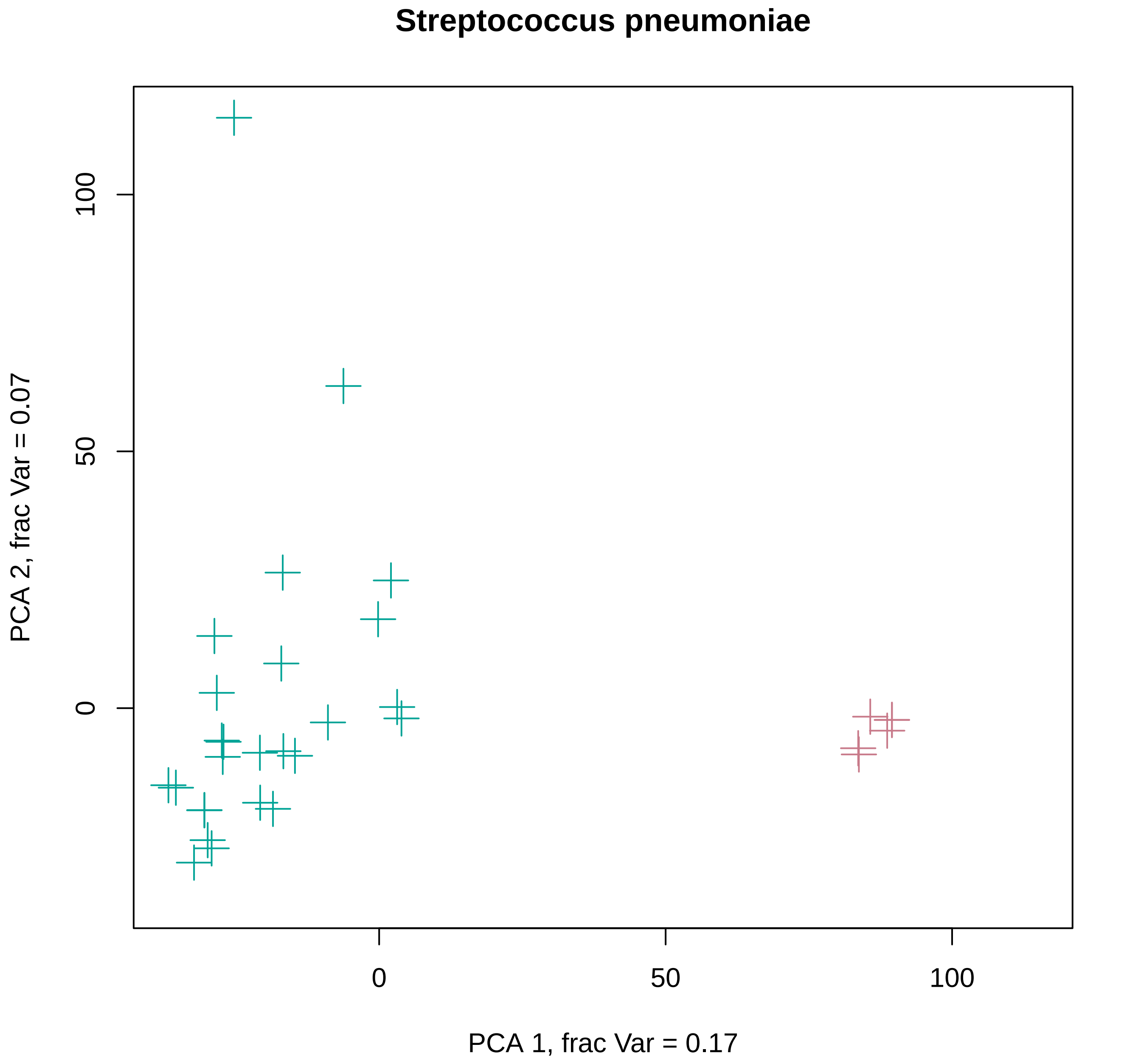

Streptococcus pneumoniae
PCA 2, frac Var = 0.07
100
50
0
0
50
100
PCA 1, frac Var = 0.17

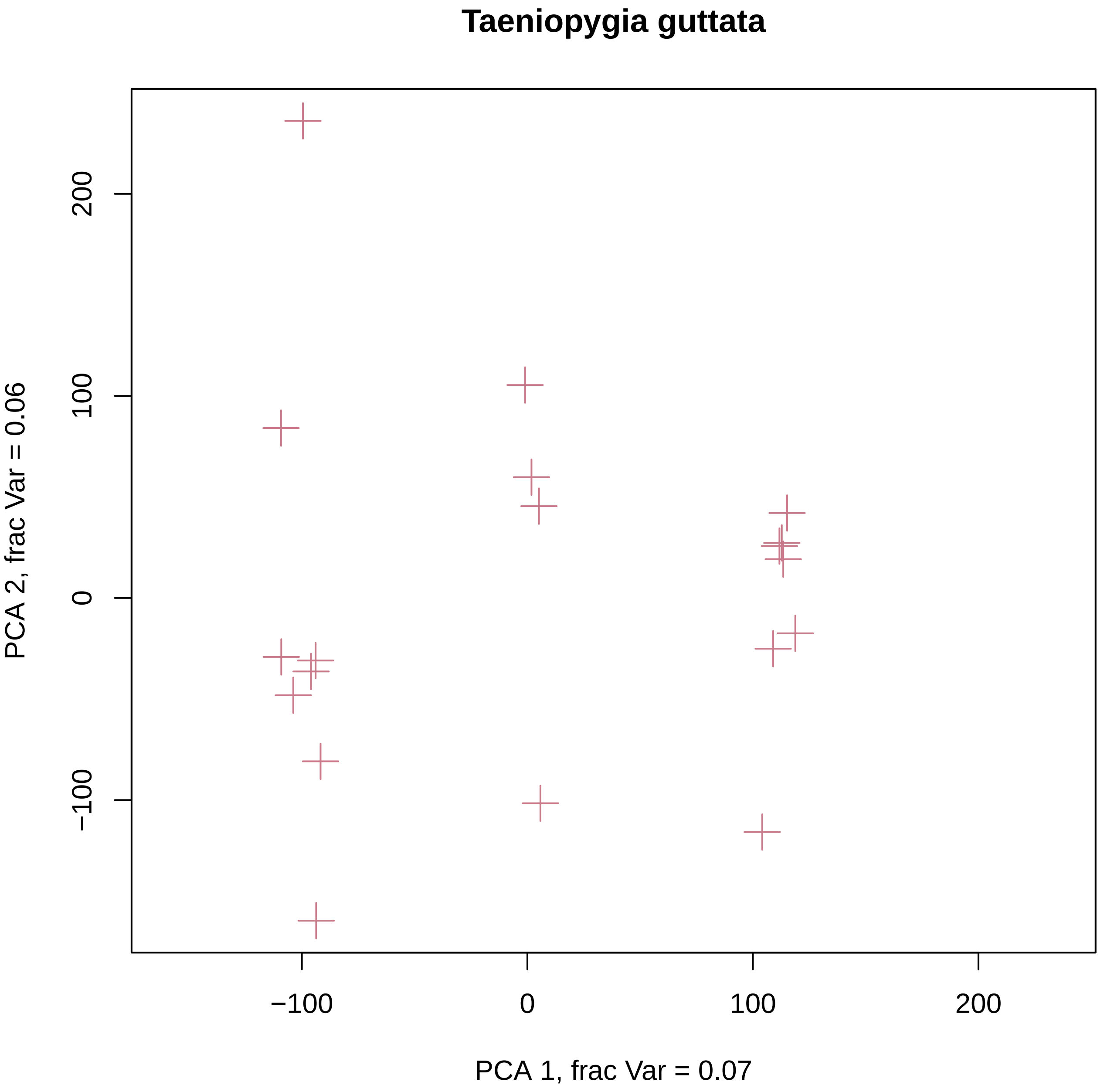

Taeniopygia guttata
200
100
0
−100
−100
0
100
200
PCA 1, frac Var = 0.07
PCA 2, frac Var = 0.06

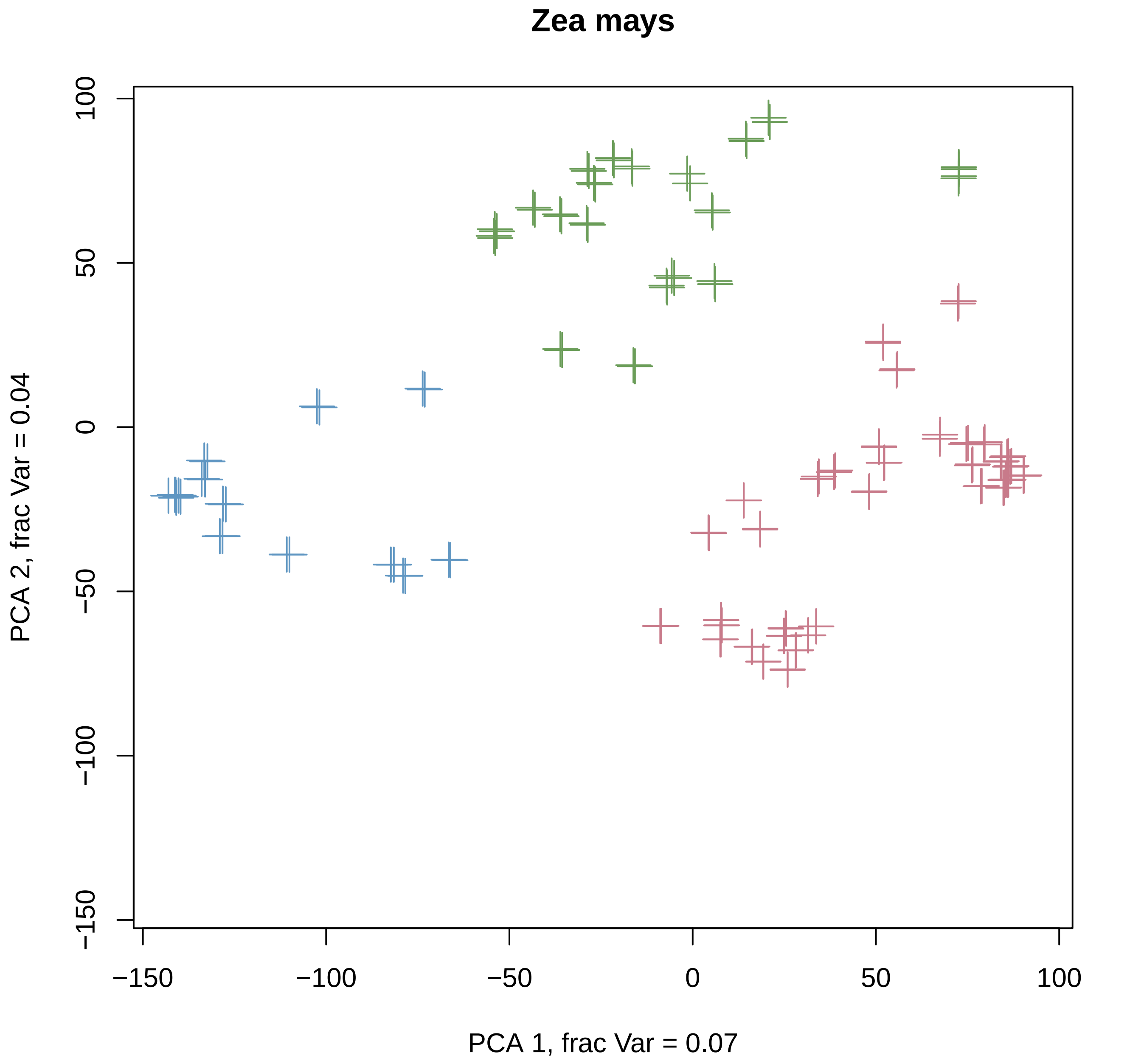
Zea mays
PCA 1, frac Var = 0.07
PCA 2, frac Var = 0.04
−150
−100
−50
0
50
100
