# Supplementary material for: Interpreting the pervasive observation of U-shaped Site Frequency Spectra
Source: PLoS Genet. 2023 Mar 23;19(3):e1010677. doi: 10.1371/journal.pgen.1010677 (PMC10072462; doi:10.1371/journal.pgen.1010677)
Supplement: S5 Fig — (PDF) [file pgen.1010677.s006.pdf]

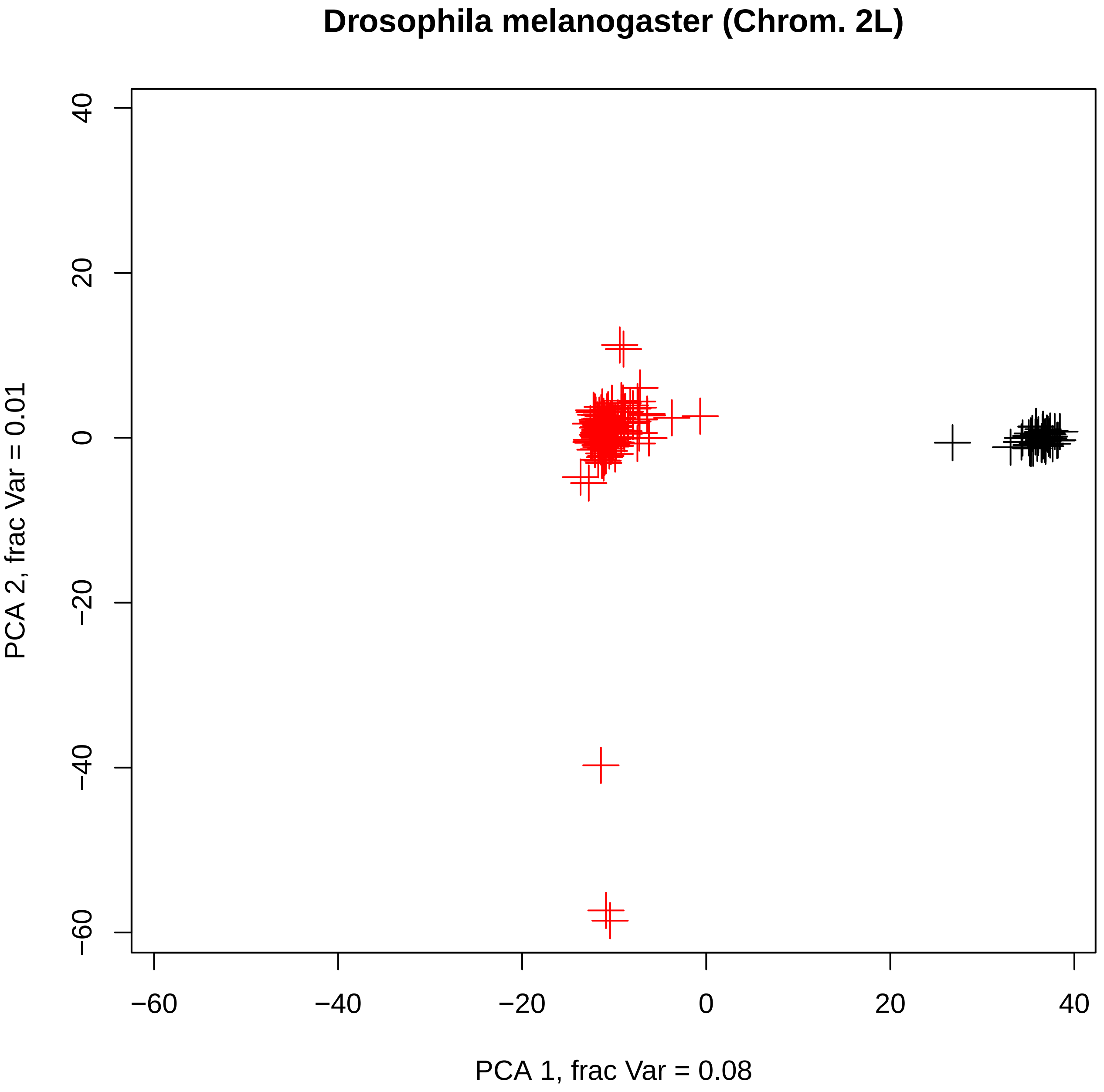

Drosophila melanogaster (Chrom. 2L)
PCA 1, frac Var = 0.08
PCA 2, frac Var = 0.01
−60
−40
−20
0
20
40

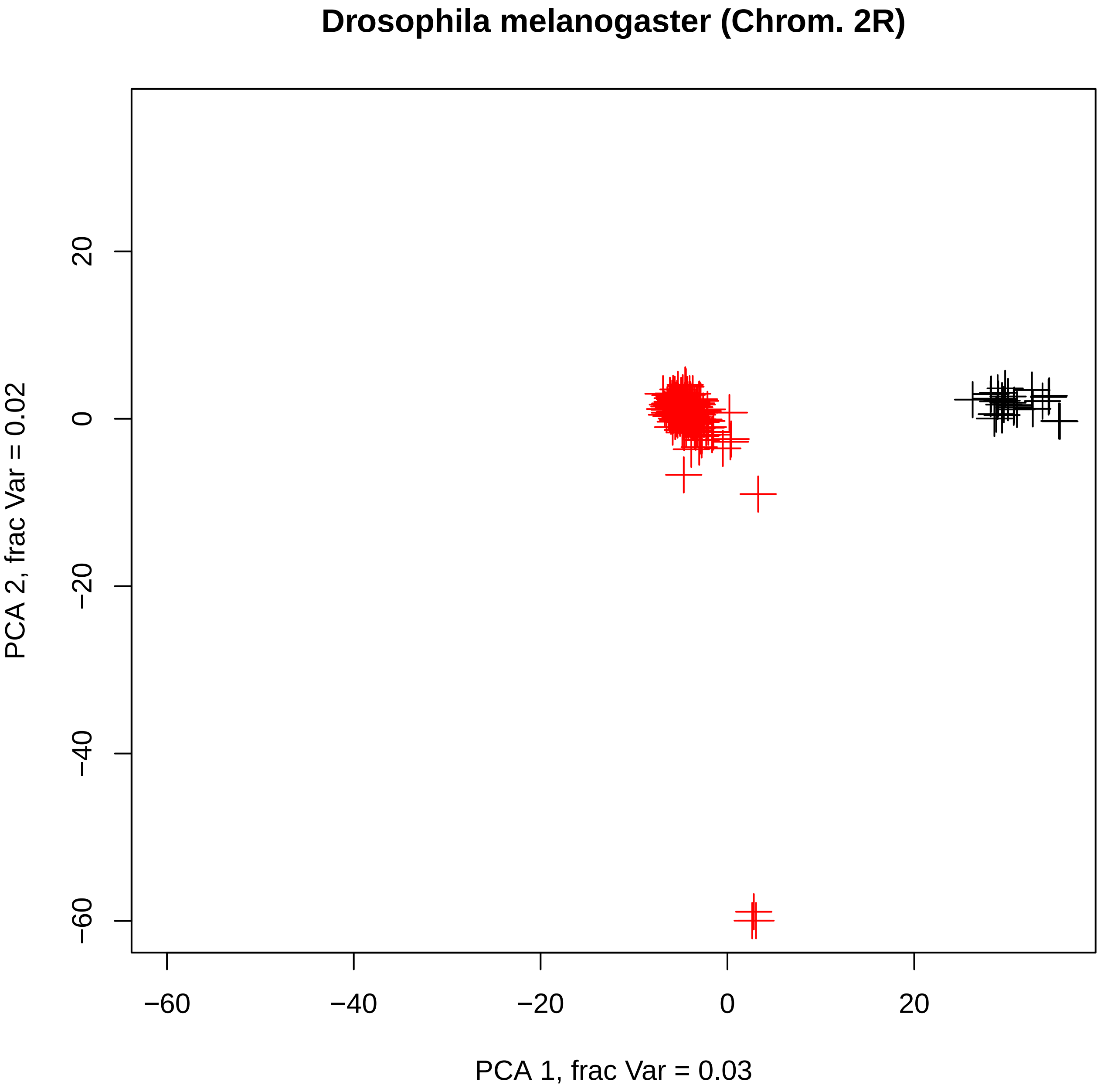

Drosophila melanogaster (Chrom. 2R)
20
0
−20
−40
−60
PCA 2, frac Var = 0.02
−60
−40
−20
0
20
PCA 1, frac Var = 0.03

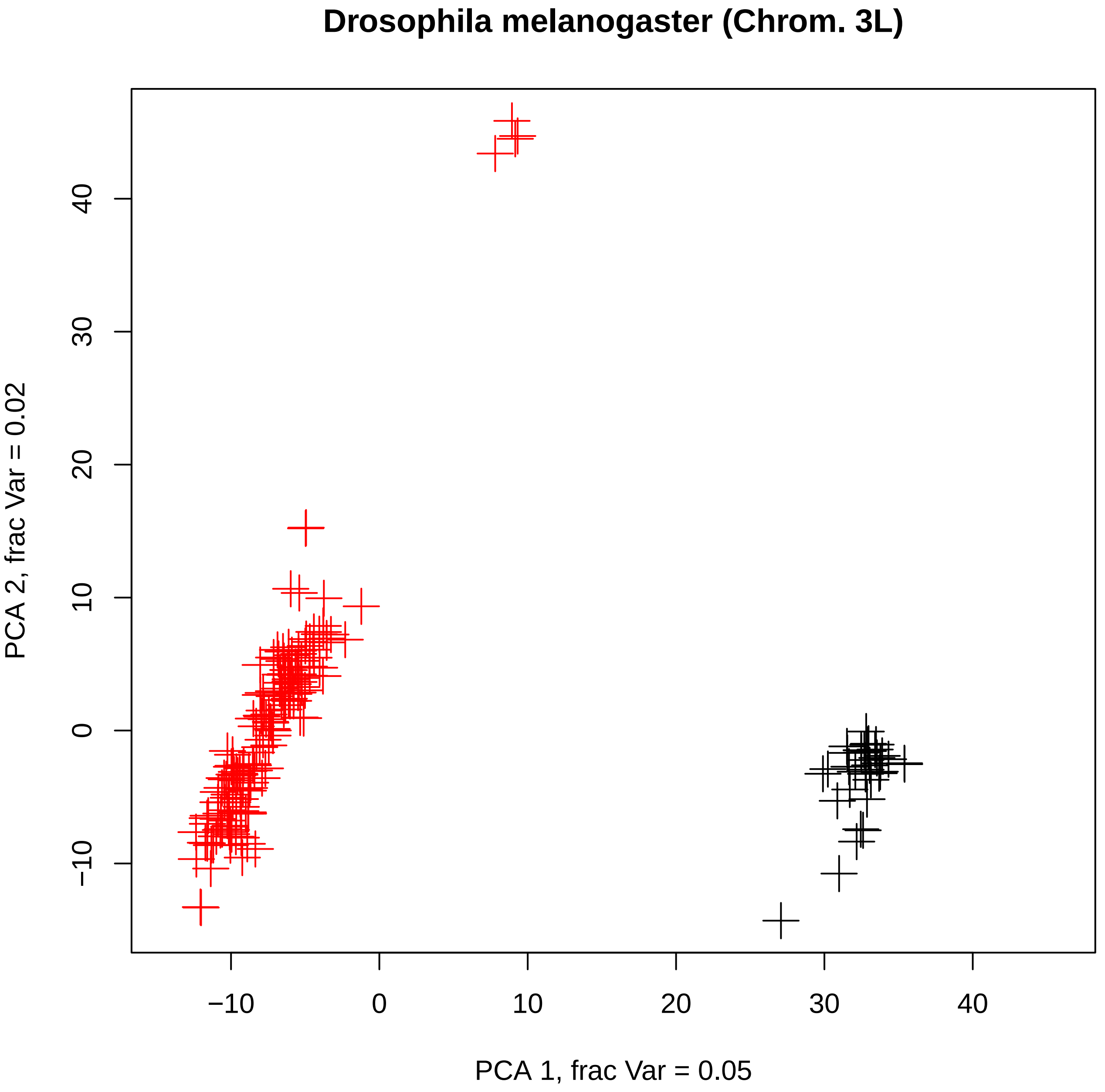
Drosophila melanogaster (Chrom. 3L)
PCA 2, frac Var = 0.02
PCA 1, frac Var = 0.05
−10
0
10
20
30
40

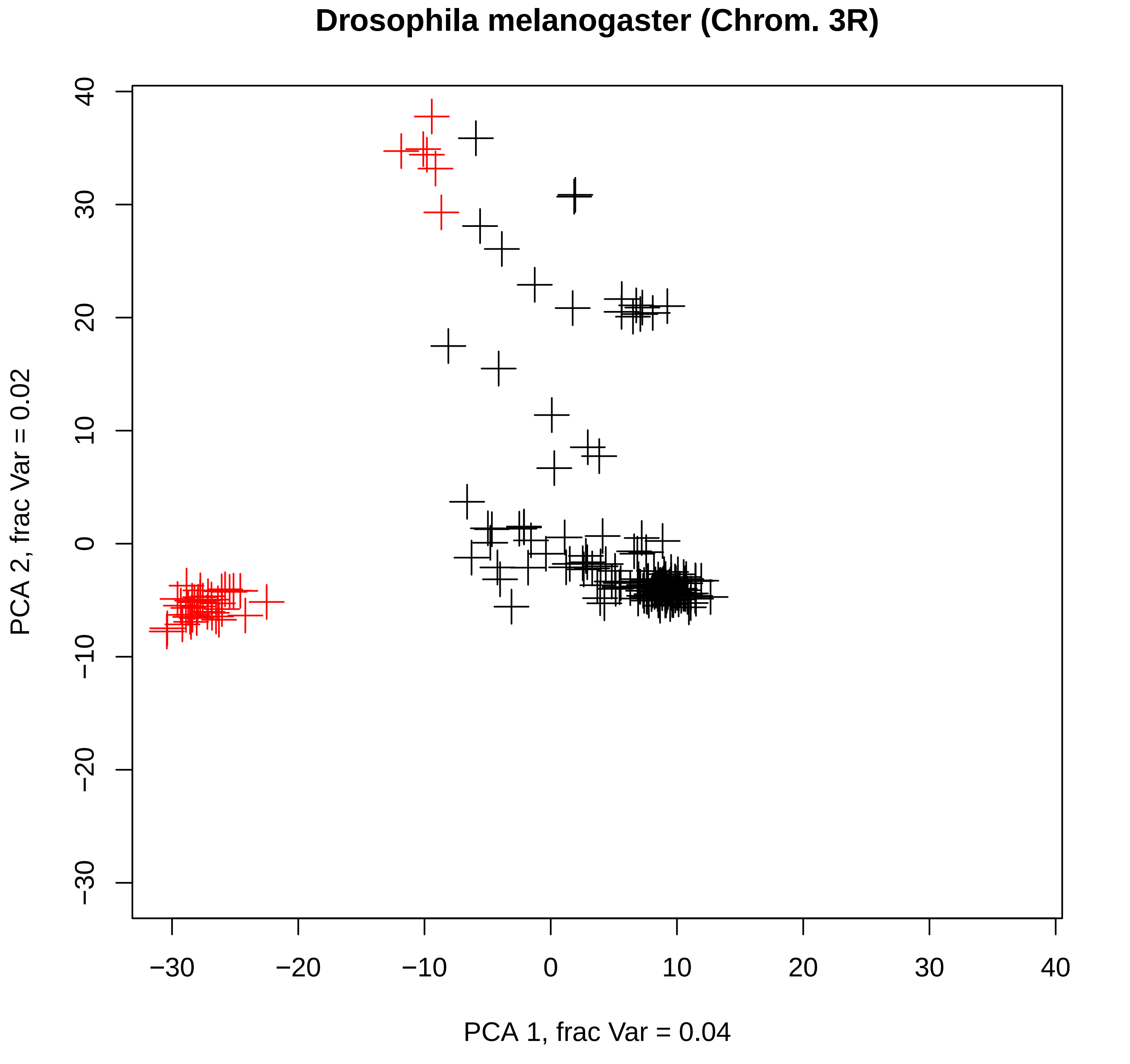
Drosophila melanogaster (Chrom. 3R)
PCA 1, frac Var = 0.04
PCA 2, frac Var = 0.02
−30
−20
−10
0
10
20
30
40
−30
−20
−10
0
10
20
30
40

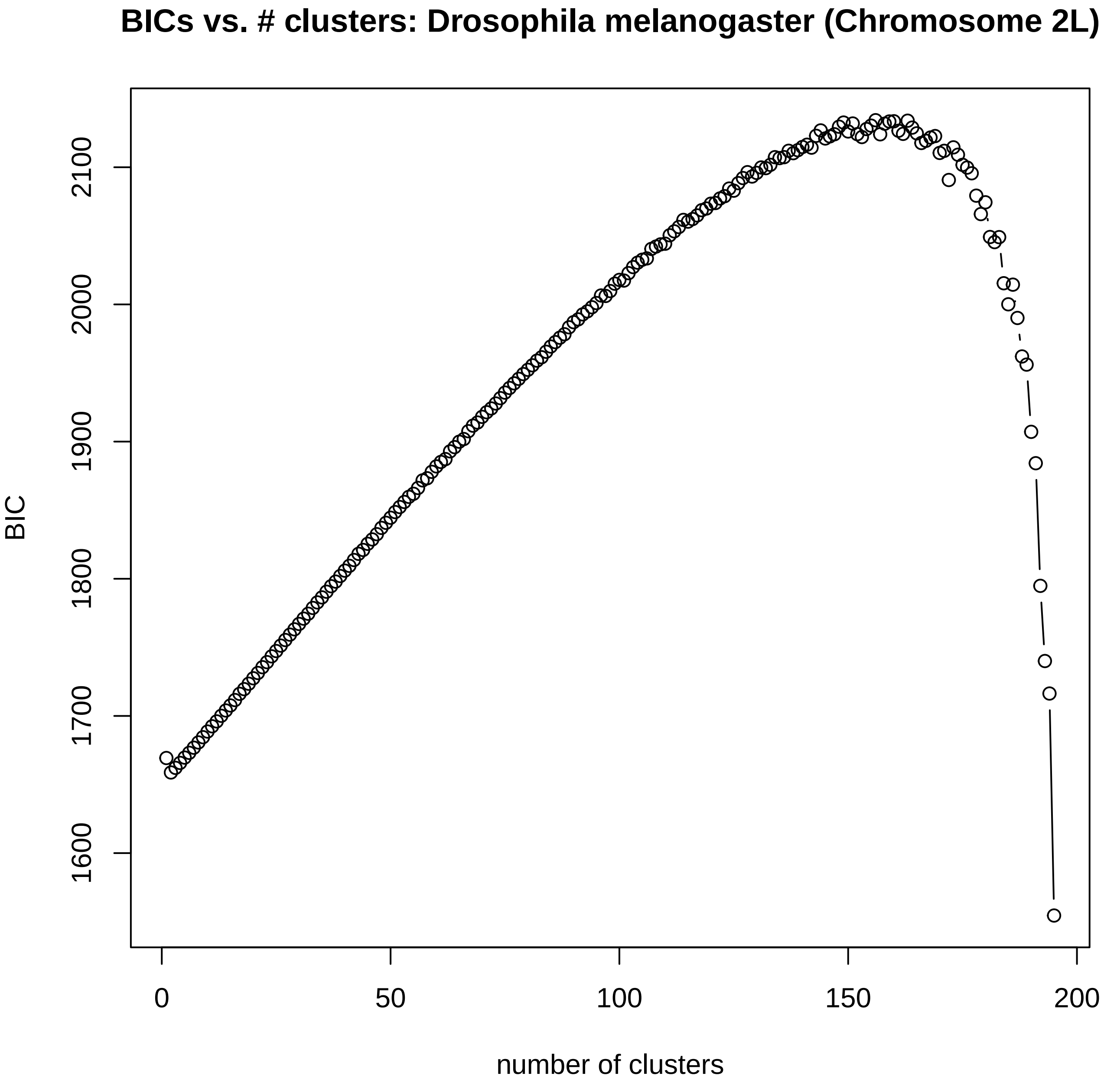
BICs vs. # clusters: Drosophila melanogaster (Chromosome 2L)
BIC
1600
1700
1800
1900
2000
2100
0
50
100
150
200
number of clusters

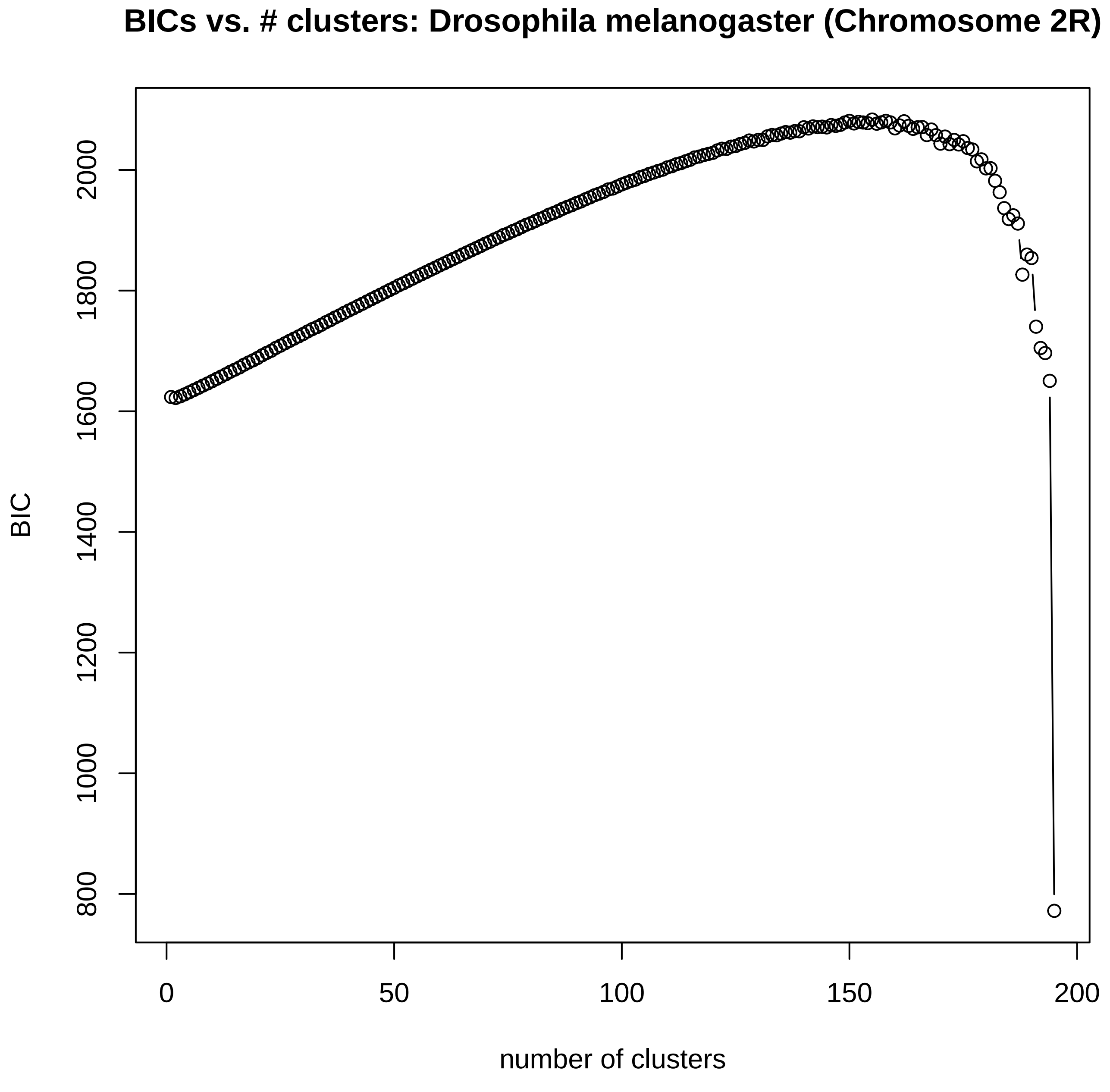

BICs vs. # clusters: Drosophila melanogaster (Chromosome 2R)
BIC
2000
1800
1600
1400
1200
1000
800
0
50
100
150
200
number of clusters

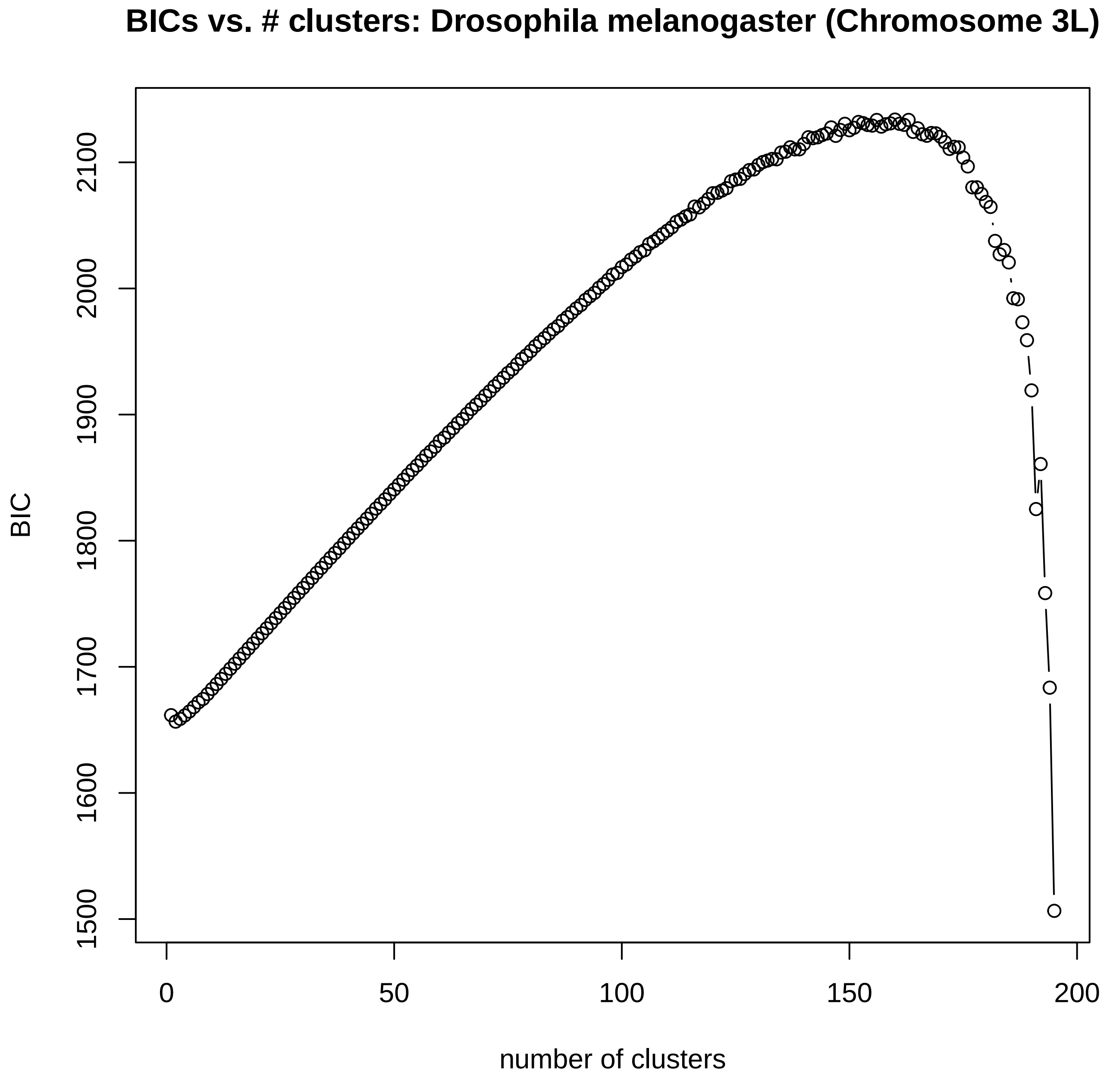

BICs vs. # clusters: Drosophila melanogaster (Chromosome 3L)
BIC
1500
1600
1700
1800
1900
2000
2100
0
50
100
150
200
number of clusters

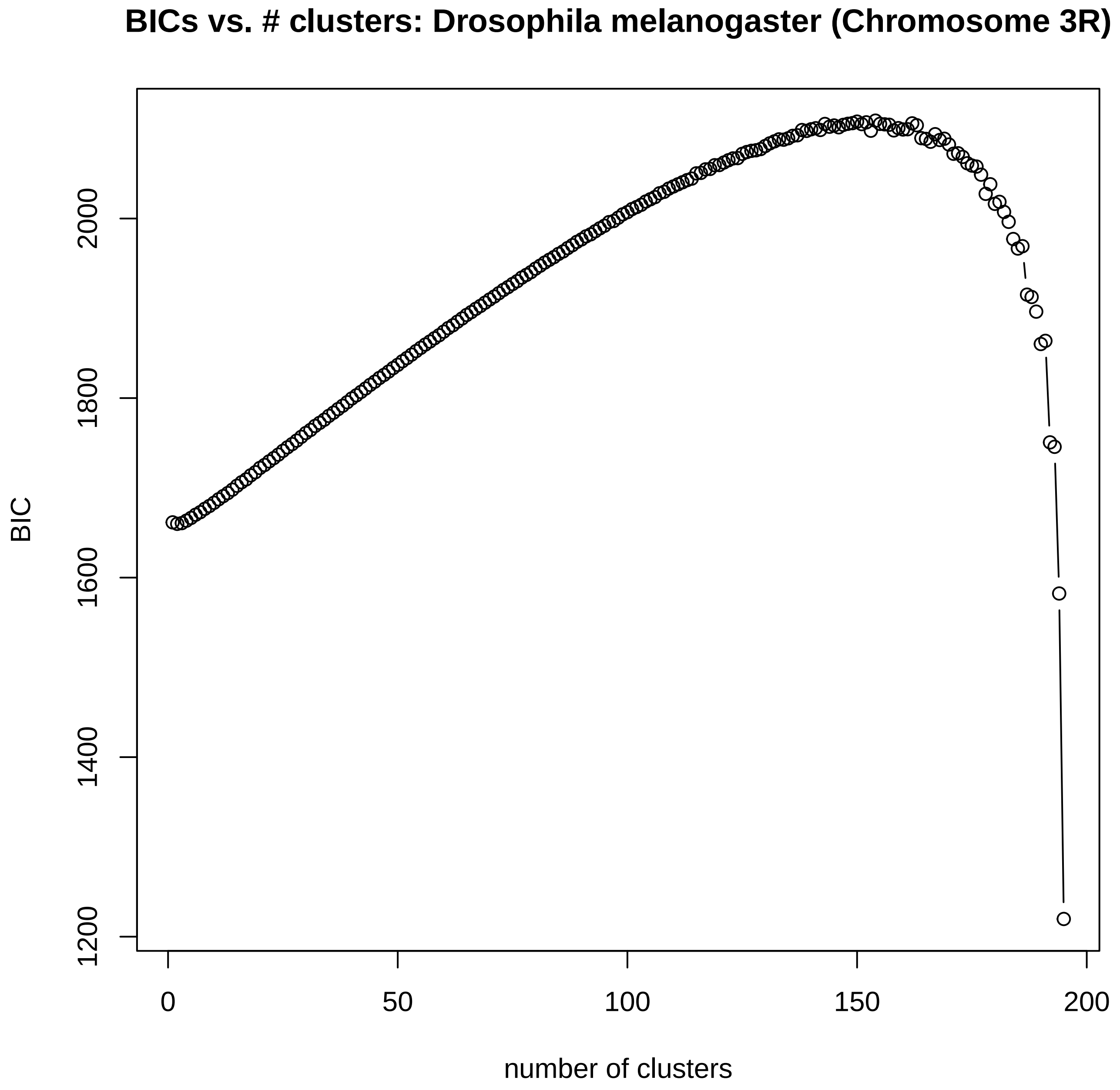
BICs vs. # clusters: Drosophila melanogaster (Chromosome 3R)
BIC
number of clusters
1200
1400
1600
1800
2000
0
50
100
150
200
