# Supplementary material for: Interpreting the pervasive observation of U-shaped Site Frequency Spectra
Source: PLoS Genet. 2023 Mar 23;19(3):e1010677. doi: 10.1371/journal.pgen.1010677 (PMC10072462; doi:10.1371/journal.pgen.1010677)
Supplement: Attachment — Submitted filename: Response_to_reviewers.pdf [file pgen.1010677.s007.pdf]

Letter to the editor

Dear Editor,

Please find here enclosed our revised version of our manuscript entitled "Interpreting the pervasive observation of U-shaped Site Frequency Spectra".

In this version, we have included almost all suggestions of the three reviewers. Most of them have resulted in new sections in the appendix and only minor modifications on the main document. We did so to keep the main document as concise as possible.

All changes in the manuscript are indicated in red.

The three main additions concern : a) a new model taking into account transitions and transversions, 2) an upper-limit quantification of ancestral polymorphisms that could create orientation errors that cannot be detected using the sites of the outgroup that display a third allele, 3) a new section giving basic knowledge of coalescent theory both for Kingman trees and for MMC trees; the section also gives basic intuition on why MMC trees result in U-shaped SFS.

We believe we have also adressed all the points raised by the reviewers. A detailed response of all points is provided below, with our response in blue to ease the reading.

We have also changed the notations in the appendix section A.3 (now A.4) to increase its clarity and ease the presentation of the new transition:traversion model.

Finally, by re-doing extra checks, we have realized that the SFS of two species (*Ciona intestinalis B* and *Corvus cornix*) were previously oriented with an incorrect outgroup. All numbers and discussions regarding these species have been updated. This does not change at all our previous conclusions (and explains why both Cionas were so different).

We believe that our manuscript is now finalized for the readers of PLoS Genetics.

Sincerely yours,

Guillaume Achaz, on behalf of all co-authors

Point by point response

[...] The manuscript was fully evaluated at the editorial level and by independent peer reviewers. All three expert reviewers were enthusiastic about your work, but raised some substantial concerns. Based on the reviews, we will not be able to accept this version of the manuscript, but we would look forward to reviewing a revised version. We cannot, of course, promise publication at that time.

We thank the editor and the reviewers for their enthusiasm about our work. We have substantially revised the manuscript to include almost all reviewer suggestions. Please find here below a point by point response to all comments.

[...] Three issues that have been raised by the reviewers are particularly important and should be addressed with care in the revision. The effects of relaxing the Jukes-Cantor assumption (specifically, transition:transversion bias) should be checked in simulation, as suggested by Reviewer 1, or included in the model (Eq'n 2). The effects of ancestral mis-specification should also be examined (sequencing errors in the outgroup), as should the effects of ancestral variation (SNPs already segregating in the ancestral population). The latter two issues are identified and explained in greater detail by Reviewer 2.

We believe we have addressed, as well as possible, the points listed here. We have developed a new version of the error rate model that explicitly takes into account transitions and transversions. Except for few exceptions, our conclusions regarding the preferential support for an MMC model over a Kingman coalescent is unaltered. We have also tested for potential ancestral polymorphisms that could explain the U-shape patterns and demonstrate that this is not a reasonable explanation for most species as the outgroups are typically phylogenetically too distant. Finally, we have explained why sequencing errors in the outgroup would not obscure our conclusion but contribute to the orientation errors, while keeping the model untouched. These new analyses are explained in details in the Appendix sections and we have added sentences in the main document presenting them in short and pointing to the appendix.

The reviewers also suggest that the manuscript could benefit from a more intuitive explanation, perhaps with some illustrative diagrams, of the differences underlying the Kingman, beta and psi coalescents, and why the latter two, or MMC in general, produce U-shaped site frequency spectra. This would also be important in aiming the manuscript toward the relatively broad audience of PLoS Genetics. [...]

We thank the reviewer for this suggestion. We have now added a new paragraph in the Introduction (paragraph on p2-3) as well as a more detailed hopefully pedagogical section on Kingman and MMC models at the beginning of the Appendix (section A.1).

Point by Point responses to reviewers comments and questions

Reviewer # 1: This study investigated how the empirical allele frequency spectra from many species match the theoretical predictions of multiple merger coalescent (MMC) vs. Kingman's coalescent. The result that MMC better explains the majority of data has a potentially great impact in the investigation of major evolutionary processes governing the pattern of genetic variation in natural populations. I think this study was carefully conducted and clearly presented. I have only one major comment (# 1 below) and other minor comments/suggestions on this manuscript.

We thank the reviewer for their enthusiasm.

1. Using eq. (2) that combines the probability of observing a third allele in the outgroup as a function of the probability of misorientation is a critical step in this study, since a U-shaped spectrum depends sensitively on the frequency of misorientation. However, I wonder the use of this pseudolikelihood function leads to under-estimation of misorientation probability, as the Jukes-Cantor model of DNA sequence evolution was assu med. In many sites where ingroup polymorphisms are due to mutations that are transitions, third alleles in the outgroup arise due to mutations that are transversions. As it is known that transitions occur about five times faster than transversions, then, the probability of double mutations leaving two alleles in the sample can be larger than that leaving three alleles in the sample, while they are assumed p and 2p in the derivation of eq. (2), respectively. Therefore, the inference of less than actual numbers of misorientation, thus overestimation of high frequency derived alleles, probably have been made. I think the effect of violating the Jukes-Cantor assumption can be easily checked in simulation, or eq. (2) can be easily modified to take transition/transversion bias into account.

We thank the reviewer for the constructive comment. Indeed, we also felt that incorporating differential mutation rate for transitions or transversions could impact our results. We have thus developed a expanded version of the inference model where both types of mutations are taken into account. This also requires the 45 datasets to be reanalyzed with two sub-SFS, one for each class of mutations. The new analysis show that, even though the orientation error rates are changed (as there are two errors rates, one for each class), the general conclusions regarding the high support for MMC models remains unchanged for the 45 species (with few exceptions reported in the text). We have added a new paragraph in the Discussion (section "Alternative processes leading to U-shaped SFS, further confounding factors.") in the main document (paragraph p12-13) and described in thorough details the new inference procedure in the Appendix [Section A.4.1] together with the inference results (Tables A.5 and A.6)

2. It will be nice if the authors can provide more illustrations from which the readers with little theoretical background can gain intuitive, biological understanding of differences between beta-, psi-, and Kingman's coalescent. In particular, how beta and psi coalescent disagree with each other is hard to imagine. The best-fitting psi-coalescent spectra might be added to Figure 2. Or, for a given series of Tajima's D or Fay and Wu's H values (statistics that geneticists are more familiar with), best matching beta, psi, and Kingman coalescent spectra could be plotted.

Although it is easy to intuitively understand that any strong inflation in the variance of the number of descendants can result in an MMC and not a Kingman coalescent, the fine differences between SFS of the Psi and Beta coalescent are indeed difficult to grasp on. The individual-based models from which they emerge are detailed in the Method section. We have now added a paragraph in the Introduction (paragraph p2-3) explaining basic differences between Kingman-like trees and both MMCs. As for the differences in the resulting SFS between Ψ and Beta, it is hard to develop a profound understanding of them. We nonetheless would like to mention that we used two MMC models to strengthen our claims that MMC models are better supported by the data than standard Kingman coalescent models, not really to see whether which one fits better. We also would like to restate that we do not think that MMC models are exact representation of the biological reality. We however defend that they provide better approximations of the biological reality than the Kingman coalescent based models (even with demography and orientation errors). We also added an explicit pointer in the main text (second paragraph on page 8) to our Supplementary files 1 and 2 with all plots showing the best fitting Beta, Psi and Kingman models. This should give the reader a meaningful feeling how the models behave and differ.

3. p.12, paragraph starting with line 13. Not all structured populations may not be identified from the PCA analysis. How about a species found in one area but undergoing metapopulation dynamics, i.e. with recurrent extinction-recolonization of multiple patches within the area?

Yes, indeed, we agree that our structure analyses will not identify all subtle or cryptic population structure. However, we do not have a good sense of how we could assess with confidence the presence/absence of unsampled "ghost" demes that can create U-shaped SFS under some parameter space (see Marchi and Excoffier, 2020). We would like to mention that some extinction-recolonisation patterns or range expansions can also lead to actual multiple-merger coalescent genealogies (Taylor, Véber EJP 2009 10.1214/EJP.v14-595; Birzu et al PNAS 2021 https://doi.org/10.1073/pnas.2026746118). Thus, detecting MMC genealogies can also be caused by meta-population dynamics. We have now added a sentence to acknowledge this in the Discussion (second paragraph of p13).

4. From which population(s) was the D. melanogaster sampled? Since fruit fly data is the most abundant one in animals (along with humans), it might be considered to divide the sample into one from a population of relatively stable demographic history and the other from populations of more deviation from simple demography. Likewise, human samples from populations other than Yoruba (with evidence of rapid expansion) might be tested separately. Such tests might help us understand the effect of demography in the mode of coalescent.

The sample is from Zambia population (Africa) [Lack et al., Genetics, 2015]. As for humans, other African populations show the same trend but the SFS from non-African population is more difficult to model with our simple demographic parameter. We agree that looking at different data sets from species to further assess the effect of population structure or other population/metapopulation-centered properties is a necessary further step to infer the main driver(s) for genome-wide genetic diversity, including whether properties leading to multiple merger genealogies are among them. However, we deem this beyond the scope of the current paper, where we want to simply pick reasonable data sets "as is" from publicly available data and assess their properties. We think that species-specific (or maybe similar-species-specifc, e.g. within genus or family) follow-up studies would be the right place for these analyses.

Reviewer # 2: Review of "Interpreting the pervasive observation of U-shaped Site Frequency Spectra" by Fabian Freund, Elise Kerdoncuff, Sebastian Matuszewski, Marguerite Lapierre, Marcel Hildebrandt, Jeffrey D. Jensen, Luca Ferretti, Amaury Lambert, Timothy B. Sackton and Guillaume Achaz:

In this manuscript, the authors investigate whether the SFS, a widely-used summary statistics of genome-wide data, of several different species across the tree of life is better fit by a genealogical model of multiple mergers, rather than the widely used standard model Kingman's coalescent, which only allows binary mergers in the genealogy. The authors use the term U-shaped Site Frequency Spectra, since an excess of high frequency derived variants in the SFS is a tell-tale of multiple merger genealogies, as this cannot be observed under Kingman's coalescent.

To this end, the authors develop an inference framework, to infer the best fitting parameters under Kingman's coalescent, the Psi-coalescent, and the Beta-coalescent, the latter two are two widely used 1-parameter families of multiple merger genealogies, which include Kingman's coalescent at one end of the parameter range. The authors include two nuisance parameters in the framework to account for confounding factors. First, they include a parameter for a model of exponential growth (or decline), since the demographic history of the population does affect the SFS. Secondly, the authors include a parameters that accounts for miss-specification of the ancestral allele. This is indeed crucial, since the excess of derived alleles is an important signal that the author try to infer, which would be heavily affected by the miss-specification.

The authors apply their method to simulated data, to demonstrate that it has power to infer the parameters of the model, and more importantly, correctly distinguish between Kingman's coalescent and multiple merger coalescents. The authors then compile around 45 SFS from different species across the tree of life. They infer the ancestral alleles based on respective outgroup populations, and also use standard approaches to scan for population structure, as this could also affect the SFS in ways that would not be captured by the vanilla Kingman coalescent. The authors can clearly reject population structure for roughly half of their datasets. They then apply their framework to infer the most likely genealogical model for each species, and detect a better support for a multiple merger coalescent in roughly 2/3 of the cases. The authors do discuss that this is not definitive proof of the sweepstake-like reproduction models that are used to motive the Psi- and Beta-coalescent, since there are other biological processes that can lead to multiple merger like genealogies, but they do conclude that in many cases, the genome-wide data is better described by a multiple merger coalescent.

The paper is a worthwhile contribution to understanding the genomic processes that shape genome-wide variation in different species. It demonstrates that in many cases, the standard Kingman coalescent is not the correct model to describe this variation, and future work will have to solidify what the exact mechanisms are in the different species that drive this deviation from the standard model. Compiling the different datasets and analyzing them in the newly introduced modelling framework is an impressive feat, and provides a good resource and reference for future studies. The statistical analysis is sound and convincing.

We thank the reviewer for their enhusiasm.

I do, however, think that the manuscript can be improved to be more accessible to a general audience by more clearly introducing key concepts. In addition, I do think that there are some possible mechanisms that the authors did not address. While I do not think that this will invalidate the conclusions of the study, I do think it would strengthen the arguments. My apologies for the amount of text in this review. I did enjoy reading the article and just have a lot of suggestions.

Specifically:

- The excess of derived alleles is a key signal underlying the inference in this study. This signal would be severely affected, if the ancestral allele is miss-specified. The authors are aware of this and address it by using different outgroups for different species to polarize the segregating variants. In addition, they co-estimate a nuisance parameter for miss-specification probability, where they leverage tri-allelic sites (including outgroup) to refine the estimate. In the model that the authors fit, the miss-specification of ancestral alleles can only result from more than one mutation at the focal site when including the outgroup. I do not think that is the only mechanism that can lead to ancestral

miss-specification. The other most important ones being sequencing error in the outgroup and incomplete-lineage-sorting.

Yes, indeed the reviewer is correct, other causes could explain the orientation errors.

Regarding sequencing error in the outgroup: If only a single sequence for the outgroup is used to polarize the SNPs, sequencing errors could readily affect the inference. In this case, a suitable model for sequencing error would be hard to incorporate, but one could include it in the framework as a single nuisance parameter for error probability. If there are multiple samples in the outgroup, then this problem is likely mitigated. Table A.5 contains a column ”Diallelic outgroup,” which seems to imply that at least for some outgroups, multiple samples are available, but it is not clearly stated in the manuscript whether this is the case. Please either include possible sequencing errors in the model or provide an argument (in the manuscript) why it does not affect the analysis.

Sequencing errors could indeed contribute to the orientation errors, as it would produce both tri-allelic sites and double-mutation convergent sites. Interestingly, sequencing errors in the outgroup will perfectly mimic orientation error and are thus expected to have the exact same effect in Jukes-Cantor setting. Indeed, whether the error stems from a biological mutation or a sequencing error in the outgroup, the effect on the polarization of the SFS is identical. Therefore, sequencing errors would stack on the orientation error, and we could be then tempted to partition them on ”real” mutations and ”sequencing” errors, but it is a difficult task without further information. The framework and the general conclusion supporting MMC over Kingman coalescents will however be untouched. To address the possibility of sequencing errors (both in the ingroup and outgroup), we have added few sentences in the Discussion regarding their potential contribution to the estimated error rate (paragraph p12-13).

Regarding incomplete-lineage-sorting or ancestral variation: If a certain SNP is already segregating in the ancestral population, then the T_{MRCA} is more ancient then the split of the focal population and the outgroup. At these SNPs, the infinite sites model in the focal population is invalid. It is unclear how these SNPs are treated in the analysis presented here. If a single sequence in the outgroup is chosen at such a SNP to polarize the focal sample, this can lead to miss-specification of the ”ancestral” allele. Again, Table A.5 has a column titled ”Diallelic outgroup,” which I figure is related to the issue that I am describing. In some cases, there are sizeable amounts of SNPs in this category. However, it is unclear how or whether these SNPs are used in the analysis, or what the sample size of the outgroups is, which would allow the reader to see in how many cases only a single sequence is used in the outgroup. Please specify in the manuscript whether the ”diallelic outgroup” are indeed SNPs (partially) resulting from incomplete-lineage-sorting. If so, state whether and how these

SNPs are included in the analysis. Incomplete-lineage-sorting should either be included in the modeling framework, or a justification presented, why it is okay to not include it. Also, please provide sample sizes for the outgroup.

Ancestral Polymorphisms would indeed blur the orientation in a non-trivial way. Ancestral polymorphism results from the joint effect of Incomplete Lineage Sorting (ILS) together with mutations on a specific branch (as illustrated in Figure A.3). However, ILS is only a real issue when the outgroup species is phylogenetically close. We have come up with a simple moment based estimation of the probability that ILS can occur in a dataset, given the diversity within and the divergence between ingroup and outgroup. We were then able to propose an estimate of the probability of ILS for each of the 45 datasets. To have problematic sites, we also need mutations on a specific branch that further reduces by an order of magnitude their occurrence. Our simple analysis shows that ILS is typically orders of magnitude less likely to occur than our estimates of orientation error rates (except for 10). Therefore we can safely assume that ILS is not the likely observation for the U-shape pattern and thus that MMC models provide a better explanation. The detailed analysis is described in the Appendix section A.5 and the general conclusions are given in the Discussion (paragrapha p12-13).
Sample size of all outgroup are now reported in Table A.7.

My apologies for the lengthy text and argument, which I hope was not entirely confusing. If I got something mixed up in my head, please tell me.

We think that the reviewer's arguments make perfect sense. We have addressed them as best as we could.

- In the article, the authors introduce the SFS, Kingman's coalescent, and report that this model is often a poor fit to observed U-shapes. The article then transitions to introducing the MMC models, and to the more technical details of the inference procedure. I think that a general audience would appreciate some heuristic explanations why MMCs can produce U-shapes before the inference framework is presented. I assume that there is some literature that exhibits these phenomena, so providing some references would be warranted. In addition, it would be good to insert a paragraph that explicitly states that MMCs can produce a U-shape, and perhaps some heuristical explanation, maybe even a figure or sketch comparing some theoretical spectra under MMCs and Kingman.

As explained below, we have added a paragraph in the Introduction attempting to provide simple explanation of how MMCs emerge and why they result in U-shaped SFS (paragraph p2-3). We have also included a more general description of Kingman and MMC models together with an illustration as the section A.1 of the Appendix.

In the discussion and Appendix A.11, the authors present arguments, why Kingman's coalescent cannot produce the U-shape, even under arbitrary non-extreme demographic models. I think this arguments is a very good addition to the article. However, as a reader, I am now especially intrigued: Following the line of reasoning presented in A.11: What feature of MMCs leads to the U-shape? Do maybe all MMCs that don't have a Kingman component necessarily produce a U-shape? While a complete exposition is probably beyond the scope of the current manuscript, it would be good to provide some heuristics to the reader as to what mechanisms cause U-shapes, or provide references. It seems to me that MMCs have high (higher than Kingman) probabilities of unbalanced trees, and that can cause U-shapes. But I might be completely wrong.

Yes, the reviewer is correct in stating that tree imbalance is more likely in MMC models. This explains why there is an overabundance of high frequency variants. We hope that the addition of the new paragaph in the Introduction and the new A.1 section of the appendix will help regarding these interrogations.

- In their model selection framework, the author fit a single-parameter model of exponential growth (decline). While I do agree that this flexibility likely captures most of the relevant effects of the demographic history, I do think it would be good to add a few sentences in the discussion speculating on how the results of the study would be affected, if a more detailed demographic model would have been fit to the data.

The framework can indeed be used with any arbitrary non-extreme demography for which the likelihood can be computed. This would be of special interest for studies interested in demographical inferences. Likely increasing the parameters for demography will improve the fit. We have added two sentences regarding this at the end of the Discussion (Conclusion, first paragraph, p14).

- p.4, l.13: I find this population growth model a bit strange, but I appreciate that it is the most convenient way to introduce all parameters correctly. Could the authors add a sentence here to be explicit about the fact that under the "non-extreme" demography assumptions, the fact that one individual has $U + G$ offspring does not lead to multiple mergers for $U = 2$, and does not change the distribution of the multiple mergers in the MMC models?

We have added a sentence at the end of the description of the modified Moran models (last paragraph p4).

- In Section 2.2 and throughout the manuscript, the authors refer to the statistics for model comparison as "Bayes factors". I think this is a bit misleading, and should be changed. Bayes factors usually incorporate prior probabilities

for the models being compared, and the authors never introduce such priors. The statistics they define are simply differences of log-likelihoods and thus most closely resemble likelihood-ratio statistics. One could argue that the statistics are Bayes factors with a uniform model prior, but I think this language should be avoided altogether. These statistics are comparing raw (composite) likelihoods, and the respective thresholds are shown to be well calibrated in the simulation study. Also, please add a sentence at the at of the second to last paragraph in Section 2.2, that the threshold log(-10) will be justified in the simulation study.

The reviewer is indeed correct in stating that this can be named log-Likelihood Ratios or Bayes Factors. Classical usage of Bayes Factor also encompasses LR (and we think this is also commonly understood in this way, see e.g. Wikipedia page on Bayes Factor). Here the two MMC models are not nested and the Likelihood are approximate, so we decided to avoid LRT and to use this arbitrary "Bayes Factor" of +/- log(10) to decide which model has a better fit. The power of this approach was, as pointed by the reviewer, evaluated by convincing simulations. We have added a sentence acknowledging that both BF and log-LR are meaningful and can be used (paragraph p5-6). We have further added the required sentence in Section 2.2 while introducing BF.

- In the first paragraph of Section 2.3, the authors state that "the collected SFS come from public data or private communications." This cannot be accepted for publication. All the datasets to re-create the study have to be accessible to the reader. The authors either have to provide the original data source, or provide unpublished datasets explicitly and include a description of how they were generated. Table A.5 provides citations for all but one dataset (Please explain what "RefSeq" means). Thus, it appears that most datasets do have an original source, and the "private communication" statement is not necessary? If the datasets were significantly altered from the original source, then the procedure should be provided explicitly. A more detailed description of the outgroup and citations for the outgroup are also needed, or a reference to the original publication of the dataset, if the outgroup is described there.

Sorry, this was an outdated sentence from an preliminary version. We have removed "private communication" and the RefSeq was completed by a proper reference.

- Figure 1 (and most other plots showing estimation error): It is somewhat difficult to follow these plots, as the spacing of the grid causes gaps in the distribution of points. A possible improvement could be to indicate the grid density on the y-axis of each plot, so it is easily visually accessible. Also, an alternative to indicating the number of replicates with a certain value by the size of the circle, could be violinplots or histograms.

We have tried hard to improve the figure using violin plots and histograms

but the outcome was way less clear, so we have decided to keep the figures as they are. The discretisation is indeed due to the grid approach, which for us seemed clear enough from this representation. We nonetheless add a sentence in the legend of Figure 2.

- Section 3.2, 3rd paragraph: Please provide a reference for Cramer's V and a reference for choosing the goodness-of-fit labels for certain ranges of the value. Please explain why it is necessary to account for different sample sizes across species. The value is computed for each species separately, so it seems that correcting for different sample sizes is not necessary.

The Cramer's V is properly referenced in section A.6. The sample size sets the number of bin sizes and thus the degree of freedom of a χ^2. As likelihoods here are only approximate and that bins are not really independent, we chose to not use the chi-square distribution but rather use a chi-square like metric that correct for bin sizes and number of sites. We have designed ourselves the categories empirically; this is now more clearly stated in the main text (second paragraph of p9).

- Figure 2: Please add a label ($i\xi_i$) to the y-axis of the displayed spectra. It is specified in the caption, but it would be good to have it readily visible in the plots. Please replace the first sentence of the caption by "Estimates of α by species." It might be worth pointing out that, since the estimate of α for E. coli is 2, the uptick in the spectrum has to come exclusively from the allele miss-specification.

Done

- Equation (4): I do think it is very helpful to include the argument of monotonic SFSs under any time-shifted Kingman coalescent. However, while the infinite sample size expression is helpful, I think it should be complemented with an argument for finite sample sizes (here or in A.11), since some sample sizes presented in the data analysis are rather small. There is no guarantee that, if a property holds for the limit, it also holds for each element of the converging sequence (not in general, that is).

We have added a reference in the main text (third paragraph of p12) and in the Appendix A.13 to Sargsyan and Wakeley (2008), where the finite/small n case is discussed (with the same conclusion of SFS being monotonic under Kingman + demograpic changes).

Minor comments:

- p.2, l. 13: ... N the effective population size, ...

- p.2, l.-13: ... number of offspring per individual has variance on the order of the total population size.

- p.3, l.5: In this study, we collected SFS from 45 species...

- p.6, l.2: We first demonstrate the power ..., and then apply it to ...

- p.6, l.7: ... for each parameter combination, choosing different values for the coalescent parameter, ...

- Table 1, caption, l. 4: The second column shows whether ...

- Table 2: What does MMC/Psi in the model column indicate?

- p.10, l.-6: ... we show that this approach has power to detect the correct MMC model ...

- p.10, l.-3: ... and orientation errors are additionally modeled, although in some cases ...

- p.11, l.-6 to -4: Non-extreme changes mean that the population size changes are on the same order of magnitude. [The current long-winded sentence in the manuscript is hard to follow.]

- p.11, l.-1: ... and a large sample is a linear function of the expected waiting times c_k for the next coalescence of k lineages, with a simple analytic form:

- p.22, l.-10: ... with distribution given in Section 2.1 leading to the ...

- p.24, l.-15: If one of the alleles is the same as the outgroup, ...

- p.24, l.-9: ... considerably longer branch lengths to the outgroup.

- p.24, l.-3: ... can be polarized (displaying one allele equal to the outgroup-allele) is 1-2p.

- p.25, l.-5: ... are independent of all other trees () ...

- Table 1 & Table A.2: Perhaps indicate the maximum in each row with boldface text, so it is readily visible.

- Figure A.6, Caption (a): Replace with "MMC parameter α or Ψ", since the blackslash is used to indicate division.

All the minor comments and typos have been corrected in the text.

Reviewer # 3: The main objective of the article is to compare the empirically observed SFS from 45-genome, associated with different species across the tree of life, to the theoretical SFS expected. To be precise, they compare the undelying genealogy given by a coalescent with multiple merges (MMC), with the neutral null model. The MMC emerged to study populations where individuals can give birth to more than two offsprings. From the mathematical point of view, they are characterized by a measure on the unit interval. For the analysis the author focused in two particular classes: the Beta- and Psi-coalescent. Also the authors, assumed an exponential growth of the population and missorientation of the ancestral allele. To decide which coalescent model fit better, they computed the two log Bayes factors. They present different scenarios from which the MMC fits better compare with the Kingman and exihibts a U-shpaed SFS.

I recommed this paper for publication after a very minor revision because it is very well organized and explain their methodology clearly. More important their results are new and interesting. The Kingman coalescent has been used for awhile as a null model and the authors give a new framework where this is not very well applied. Also, the references are very helpful to see clearly their contributions and present an overview of the state of the matter.

We thank the reviewer for their enthusiasm.

Minor comments.
In Table 2 it is not specified the meaning of MMC/Psi.
P. 3 L. 11 Explain the meaning of pure radiation.
P. 9 L. -19 remove one ”the”
P. 24. L.-6 biallelic.

We have replaced MMC/Psi (an old notation) by simply “MMC” (the one used in the current version) in the Table 2 and corrected the minor comments and typos.
