# Supplementary material for: Interpreting the pervasive observation of U-shaped Site Frequency Spectra
Source: PLoS Genet. 2023 Mar 23;19(3):e1010677. doi: 10.1371/journal.pgen.1010677 (PMC10072462; doi:10.1371/journal.pgen.1010677)
Supplement: Attachment — Submitted filename: answer_to_refs2.pdf [file pgen.1010677.s008.pdf]

Dear editor, dear reviewer,

thank you for reading and commenting on our revised version of the manuscript.

We have implemented nearly all suggested changes (details are listed below, and we explained for one suggestion why we opted to not implement it).
Additionally, we have double-checked our analyses for a small number of data sets where we had found discrepancies between the results of our main parameter estimation and coalescent model selection approach and the newly added alternative version accounting for differences between transition and transversion mutation rates. For two data sets (Zea mays and Drosophila melanogaster), we found discrepancies between our filtering of the sequence data (to extract site frequency spectra including ancestral allele calls) between these inference approaches. We have now aligned the data filtering approaches for the two data sets. This lead to now equal results from both inference methods (results for these two data sets in Tables A.5 and A.6 are thus revised). Additionally, we updated Table A.23 and Table A.9, where minor changes in results prior to this revision were not yet reflected. Apart from this, we only corrected a handful of typos.

All data underlying plots (and tables) as well as simulation, inference and data analysis pipelines (including filtering) are available in the public GitHub repository https://github.com/fabfreund/usfs_mmc.

Point-for-point answer to referee's suggestions:
Referee: I do, however, still feel that Table A.7 (previously A.5) needs to be presented better so that the description of the datasets and the analysis performed is clearer. In most rows, only one reference is given in the column "Source." Does this reference, in all cases, include a description of the outgroup? If not, a reference for the outgroup needs to be added. For M. tuberculosis, the "Source" column only indicates "RefSeq". Please provide a reference here, and if necessary, a date when the database was accessed.

Our answer: We have added all references for the outgroups and database access dates. This can be seen in the pdf of the new version, but it is not fully visible in the pdf with the marked differences between the new version and the previous version.

Referee: Please add a clear description (perhaps in the caption) as to what the column "Diallelic outgroup" refers to. I assume it means number of SNP-sites in the ingroup where the outgroup shows two alleles. This a bit at odds with A. thaliana. Here, the outgroup size is 1, but there are 400,000 such sites. Is this a typo? Or is the outgroup 1 diploid individual that is heterozygous at all these sites?

Our answer: Yes, this is the exact meaning we intended for "Diallelic outgroup". For the diploid and usually outcrossing A. lyrata, the outgroup for A. thaliana, we used a single reference alignment (thus counting one sequence) which comes from PacBio reads as described in the reference given in Table A.7. So from our understanding, these are indeed true polymorphisms in the outgroup, but compressed into a single reference (with marked-up variable sites).

Referee: Moreover, if there are two alleles in the outgroup, then there are 3 possible combinations with the ingroup:
1. Same two alleles in outgroup as in ingroup.
2. One allele shared between in- and outgroup, the other allele different.

3. No allele shared (probably very unlikely and perhaps not observed often).
Please replace the column "diallelic outgroup" by two columns that show the number for 1. and 2. Moreover, for 2., it is clear what the inferred ancestral allele is. However, for 1., which is an ancestral polymorphism, what is the "ancestral" allele that is used in the analysis. How is it determined?

Our answer: This is, in our view, not really necessary: We will always exclude any diploid outgroup positions from further analysis, since we then cannot assume that the ancestral allele is the only present outgroup allele. We added a sentence in the legend clarifying this.

Referee: Minor comments:
- p.2, l.-5: ... recurring ancestor of a substantial fraction of the population … DONE!
- p.2, l.-3: ... the presence of a single ancestor … DONE!
- Section 3.2, third paragraph: Please insert an explicit reference to Appendix A.6 in this paragraph. We added a reference in subsection “MMC is sometimes a good fit”
- p.12, l.-12: ... exact same patterns as natural mutations. DONE!
- p.23, l.6: Let us consider a reproduction model, for instance … DONE!
- p.23, l.11: ... is the (random) tree obtained from tracing back the genealogy … DONE!
- p.23, l.-14: Neutral genetic diversity results from neutral mutation events … We have changed this to omitting both instances of neutral in this sentence. Instead, we added a sentence at the end of this paragraph that explains that mutations are neutral in our models, but for some MMC models there still are included selection effects.
- p.30, l.5: ... compare estimated diversity between in- and outgroups … DONE!
- Supplement 1: The linestyles change between some of the plots. Please have all plots with unified linestyles.

This was actually intended, but not properly explained by us. The best fitting model is the one with the solid, non-dotted line (2 solid lines for Beta and Psi coalescent if the test points towards MMC but is inconclusive towards which model fits significantly better). We added an explanatory legend page as a “title page” to both supplementary files 1 and 2.
- Supplement 1 & 2: I think it would be good to add an indicator in each plot as to what model was chosen as the most likely one. See comment above

Many thanks for the suggestions and the careful reading of our manuscript.

On behalf of all authors,

Fabian Freund (soon at the University of Leicester)
